# BAMBI Is a Prognostic Biomarker Associated with Macrophage Polarization, Glycolysis, and Lipid Metabolism in Hepatocellular Carcinoma

**DOI:** 10.3390/ijms252312713

**Published:** 2024-11-26

**Authors:** Huijie Gao, Cuimin Hu, Qing Wu, Zhongze Fang

**Affiliations:** 1 Department of Pathogen Biology, School of Basic Medical Sciences, Tianjin Medical University, Tianjin 300070, China; gaohuijie@tmu.edu.cn (H.G.);; 2 Department of Toxicology and Sanitary Chemistry, School of Public Health, Tianjin Medical University, Tianjin 300070, China

**Keywords:** HCC, BAMBI, prognosis, macrophage polarization, cell signaling pathways

## Abstract

Hepatocellular carcinoma (HCC) is one of the most common types of cancer worldwide. Affected patients have poor prognoses due to high rates of post-surgical recurrence and metastasis. Bone morphogenetic protein and activin membrane-bound inhibitor (BAMBI) reportedly contributes to the development and progression of various human cancers. Thus far, there have been no comprehensive studies regarding the expression of BAMBI in HCC; similarly, no studies have investigated the prognostic significance of BAMBI and its associated mechanisms in HCC. In this study, we analyzed the expression profiles of BAMBI, along with its contributions to pathological findings, metastasis characteristics, and prognosis, in multiple human cancers. We found that upregulation of BAMBI was associated with poor prognosis in HCC. Next, we explored the associations of BAMBI with multiple cell signaling pathways, immune cells, and immune checkpoints in HCC. The results showed that BAMBI was associated with tumor proliferation, epithelial–mesenchymal transition (EMT) markers, glycolysis, fatty acid biosynthesis and degradation pathways, and immune checkpoint regulation in HCC. In vitro and in vivo experiments showed that BAMBI promoted polarization of M1 macrophages and is linked to the expression of key genes involved in glycolipid metabolism. Furthermore, protein–protein interaction analysis suggested that BAMBI plays multiple roles in HCC by regulating genes in the transforming growth factor (TGF)-β and Wnt signaling pathways. Our findings elucidated that BAMBI is a prognostic biomarker and is associated with macrophage polarization, glycolysis, and lipid metabolism in HCC.

## 1. Introduction

Liver cancer (HCC) is one of the most common fatal malignancies worldwide [1]; it is often diagnosed during the late stages of the disease, leading to a poor prognosis [2]. Despite global efforts to improve the clinical management of HCC, affected patients continue to have poor outcomes due to high rates of post-surgical recurrence and metastasis [3]. Efforts to identify the molecular mechanisms and targets that drive HCC metastasis and progression are expected to improve patient survival. Extensive research has been conducted to elucidate the molecular mechanisms that underlie the development and progression of HCC [4,5,6,7,8,9,10]. For example, the profound impacts of immune cells on HCC development and progression have received increasing attention; the immune evasion abilities of HCC represent a major therapeutic challenge [4,5]. Therefore, many studies have focused on interactions between the immune microenvironment and HCC. Additionally, there is some evidence that glycolysis and lipid metabolism disorders play key roles in the development and progression of HCC [6,8].

Bone morphogenetic protein and activin membrane-bound inhibitor (BAMBI), with the genomic location of human in 10p12.1, is regarded as a transmembrane pseudoreceptor due to its lack of an intracellular protein kinase domain; it functions as an antagonist of transforming growth factor (TGF)-β type 1 receptors (TGF-β1Rs) [11,12]. BAMBI is reportedly linked to the development and progression of various human cancers, including colorectal cancer, HCC, lung cancer, bladder cancer, gastric cancer, and gliomas [13,14,15,16,17]. For example, BAMBI contributes to colorectal and hepatocellular tumorigenesis by interfering with TGF-β-mediated growth arrest [13]. It also promotes macrophage proliferation and differentiation in gliomas [14]. The knockdown of BAMBI suppresses metastasis in gastric cancer cells by inhibiting β-catenin and TGF-β [15]. Our previous studies identified BAMBI as a target of miR-HCC2 and showed that BAMBI is involved in the proliferation, metastasis, and stem cell-like properties of HCC cells (both in vivo and in vitro) by promoting the activity of the Wnt/β-catenin signaling pathway [18,19]. Thus far, there have been no comprehensive studies regarding the expression of BAMBI in HCC; similarly, no studies have investigated the prognostic significance of BAMBI and its associated mechanisms in HCC. In particular, the relationships of BAMBI with tumor immunity and glycolipid metabolism in HCC remain unclear. Therefore, a comprehensive analysis is needed concerning the functions of BAMBI in HCC, along with its possible mechanisms.

In order to explore the importance of the BAMBI gene as a prognostic indicator, we analyzed the expression profiles of BAMBI, along with its contributions to pathological findings, metastasis characteristics, and prognosis in various human cancers. To further deeply analyze the specific molecular mechanisms by which BAMBI promotes the development and progression of HCC, we explored the associations of BAMBI with multiple cell signaling pathways, immune cell infiltration, biomarkers for immune cells, and immune checkpoints in HCC. Furthermore, we constructed a protein–protein interaction network to predict and analyze genes that BAMBI was likely to interact with; we sought to elucidate the mechanisms by which BAMBI contributed to HCC development and progression.

## 2. Results

### 2.1. Pan-Cancer Analysis of BAMBI Expression

**Figure 1:**
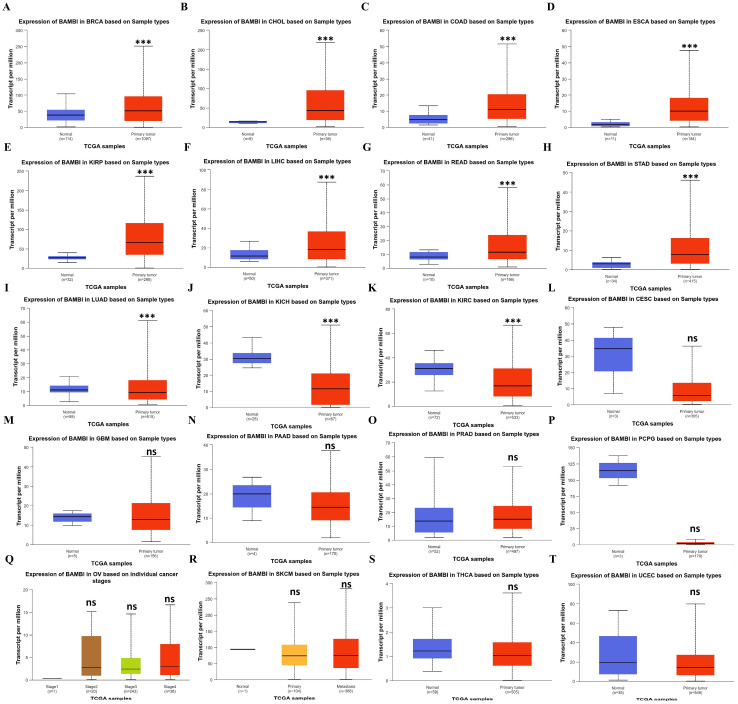
Analysis of BAMBI expression in 20 types of cancers, validated using the UALCAN database. (**A**–**H**) BAMBI expression was increased in BRCA (**A**), CHOL (**B**), COAD (**C**), ESCA (**D**), KIRP (**E**), HCC (**F**), READ (**G**), and STAD (**H**) compared with normal tissues. (**I**–**K**) BAMBI expression was decreased in LUAD (**I**), KICH (**J**), and KIRC (**K**) compared with normal tissues. (**L**–**T**) There were no significant differences in BAMBI expression between cancer and normal tissues in CESC (**L**), GBM (**M**), PAAD (**N**), PRAD (**O**), PCPG (**P**), OV (**Q**), SKCM (**R**), THCA (**S**), or UCEC (**T**). ns, not significant; *** *p* < 0.001.

Since BAMBI is linked to the development and progression of various human cancers, we explored the expression profiles of BAMBI in 24 human cancer types. As shown in Figure S1, analysis of data from The Cancer Genome Atlas (TCGA) revealed that BAMBI was significantly upregulated in eight types of cancers (BRCA, CHOL, COAD, ESCA, KIRP, HCC (LIHC dataset), READ, and STAD) and downregulated in four types of cancers (CESC, KICH, KIRC, and PCPG) compared with the corresponding normal tissues. However, analysis of data from TCGA and the Genotype-Tissue Expression (GTEx) revealed that BAMBI was significantly upregulated in 15 types of cancers (BRCA, CHOL, COAD, DLBC, ESCA, GBM, KIRP, LGG, HCC, PAAD, PRAD, READ, STAD, SKCM, and TGCT) and downregulated in 7 types of cancers (KICH, PCPG, LUAD, LUSC, OV, THCA, and UCEC) compared with normal tissues. Therefore, we validated the expression of BAMBI in each cancer type using the University of ALabama at Birmingham CANcer data analysis portal (UALCAN). As shown in Figure 1A–H, BAMBI expression levels were significantly increased in BRCA, CHOL, COAD, ESCA, KIRP, HCC, READ, and STAD compared with the corresponding normal tissues, and BAMBI expression levels were significantly decreased in LUAD, KICH, and KIRC (Figure 1I–K). However, no significant differences were detected in other cancer types (Figure 1L–T). These results suggest that BAMBI plays an important role in carcinogenesis in 11 cancer types.

### 2.2. BAMBI Is Associated with Prognosis, Pathological Stage, and Nodal Metastasis in Human Cancers

**Figure 2:**
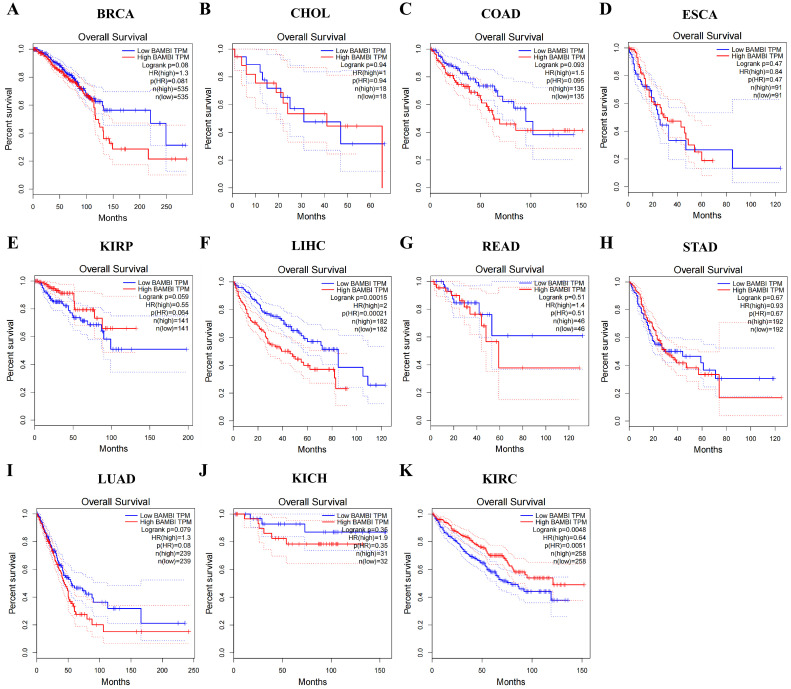
Analysis of overall survival according to BAMBI expression in 11 types of cancers, as determined using the GEPIA database. (**A**–K) Plots of overall survival according to BAMBI expression in BRCA (**A**), CHOL (**B**), COAD (**C**), ESCA (**D**), KIRP (**E**), HCC (**F**), READ (**G**), STAD (**H**), CESC (**I**), KICH (**J**), and KIRC (**K**). *p*-values < 0.05 were considered statistically significant.

To elucidate the contributions of BAMBI to prognosis, pathological stage, and nodal metastasis in BRCA, CHOL, COAD, ESCA, KIRP, HCC, READ, STAD, LUAD, KICH, and KIRC, we analyzed the associations of BAMBI with overall survival, disease-free survival, tumor grade, and nodal metastasis. Regarding overall survival and disease-free survival, higher BAMBI expression was associated with poor prognosis in patients with HCC. In contrast, BAMBI was associated with poor prognosis in patients with KIRC (Figure 2 and Figure S2). No significant associations of BAMBI expression with patient prognosis were detected in the other nine cancer types.

The relationships between BAMBI and pathological stage, tumor grade, and nodal metastasis in HCC and KIRC were examined using gene expression profile interactive analyses. BAMBI significantly correlated with the pathological stage of KIRC (*p* < 0.05) but not HCC (*p* = 0.0523) (Figure S3A,B). BAMBI expression tended to increase as tumor grade increased from 1 to 4 in patients with HCC but decreased as tumor grade increased in patients with KIRC. In patients with tumor grades 1 to 4, BAMBI expression levels were significantly higher in HCC and lower in KIRC compared with the levels in the corresponding normal tissues (Figure S3C,D). The BAMBI expression levels were significantly higher in KIRC tumors grade 4 compared with the levels in KIRC tumor grades 1, 2, and 3 (Figure S3C). However, no significant differences in BAMBI expression were detected among HCC tumor grades (Figure S3D).

The degree of metastasis tended to increase in patients with HCC and decrease in patients with KIRC upon progression from normal tissues to N1 tumors. BAMBI expression levels were higher in N0 and N1 tumors compared with the levels in normal tissues for both KIRC and HCC (Figure S3E,F). Overall, these results demonstrate that high BAMBI expression correlates with poor prognosis and greater nodal metastasis in patients with HCC, whereas the opposite relationship occurs in patients with KIRC.

Our previous study also showed that the expression levels of BAMBI were significantly higher in HCC samples than in the adjacent normal samples, and it could be targeted and upregulated by miR-HCC2 [19]. Further experiments showed that BAMBI contributed to the proliferation, metastasis, and stem cell-like properties of HCC cells through activating Wnt/β-catenin signaling pathway. In general, BAMBI is a potential diagnostic biomarker and an indicator of unfavorable prognosis in patients with HCC.

### 2.3. Correlations Between BAMBI and Cell Signaling Pathways in Patients with HCC

**Figure 3:**
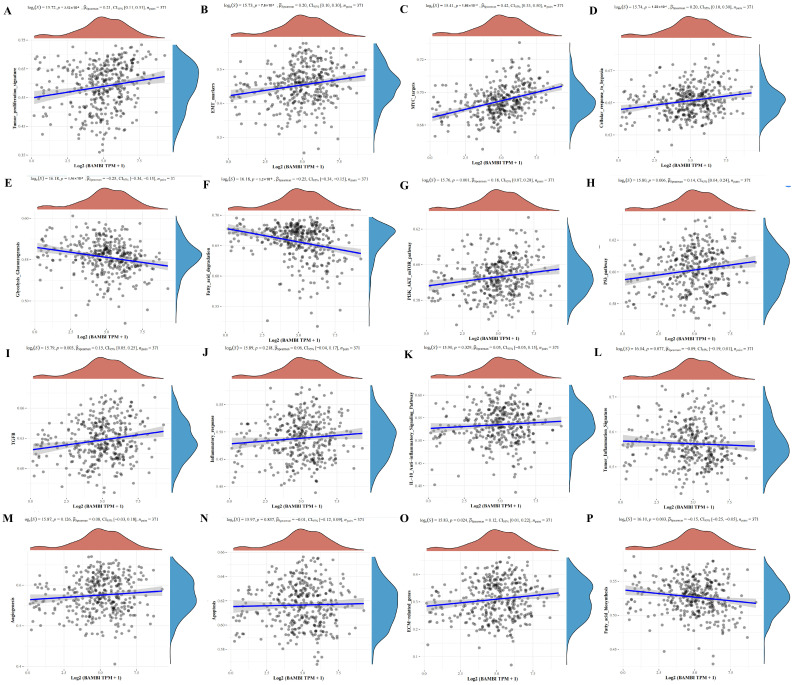
Prediction and analysis of cell signaling pathways in HCC with potential BAMBI involvement. (**A**–**P**) Correlations of BAMBI expression with tumor proliferation (**A**), EMT markers (**B**), MYC targets (**C**), cellular response to hypoxia (**D**), glycolysis/gluconeogenesis (**E**), fatty acid degradation pathways (**F**), PI3K-AKT-mTOR pathway (**G**), p53 pathway (**H**), TGF-β (**I**), inflammatory response (**J**), IL-10 anti-inflammatory signaling pathway (**K**), tumor inflammation (**L**), angiogenesis (**M**), apoptosis (**N**), ECM-related genes (**O**), and fatty acid biosynthesis (**P**). All correlations were analyzed using the GSVA package in R software (version 4.0.3) with the method parameter set to ‘ssgsea’. Relationships of BAMBI expression with cell signaling pathway scores were analyzed by Spearman correlation. *p*-values < 0.05 were considered statistically significant. The density curve on the right shows the distribution trend of the pathway score, while the density curve on the top represents the distribution trend of the expression of BAMBI. The values at the top represent the results of the Spearman correlation analysis, including the *p*-value, correlation coefficient, and correlation calculation method. The blue lines depicted the trends of the correlation.

To identify pathways and mechanisms by which BAMBI influences the development and progression of HCC, we evaluated correlations between BAMBI and 16 cell signaling pathways, including tumor proliferation, tumor inflammation, inflammatory response, interleukin (IL)-10 anti-inflammatory signaling pathway, TGF-β, glycolysis/gluconeogenesis, fatty acid biosynthesis, fatty acid degradation pathways, cellular response to hypoxia, EMT markers, extracellular matrix (ECM)-related genes, angiogenesis, apoptosis, phosphoinositide 3-kinase (PI3K)-protein kinase B (AKT)-mechanistic target of rapamycin (mTOR) pathway, p53 pathway, and MYC targets. As shown in Figure 3, elevated BAMBI expression in HCC positively correlated with tumor proliferation, EMT markers, MYC targets, cellular response to hypoxia, PI3K-AKT-mTOR, p53 pathway, TGF-β pathway and ECM-related genes (Figure 3A–D,G–I,O) and negatively correlated with glycolysis/gluconeogenesis and fatty acid biosynthesis and degradation pathways (Figure 3E,F,P). No significant correlations of BAMBI with the other five signaling pathways were detected (Figure 3J–N).

Massive evidence showed that cancer cells often exhibited heightened glycolytic activity and enhanced lipid metabolism. However, BAMBI as an indicator of unfavorable prognosis negatively correlated with glycolysis/gluconeogenesis and fatty acid degradation pathways. To further explore the relationship between BAMBI with glycolysis and lipid metabolism, we conducted correlation analyses for each of the 62 genes involved in the glycolysis/gluconeogenesis pathway, 17 genes in the fatty acid biosynthesis pathway, and 43 genes in the fatty acid degradation pathway with BAMBI (Supplementary Data S1). Interestingly, the results showed that the majority of genes (25 genes in the glycolysis/gluconeogenesis pathway, 6 genes in the fatty acid biosynthesis pathway, and 8 genes in the fatty acid degradation pathway) showed a positive correlation with BAMBI, while a minority (9 genes in the glycolysis/gluconeogenesis pathway, 2 genes in the fatty acid biosynthesis pathway, and 9 genes in the fatty acid degradation pathway) exhibited a negative correlation.

**Figure 4:**
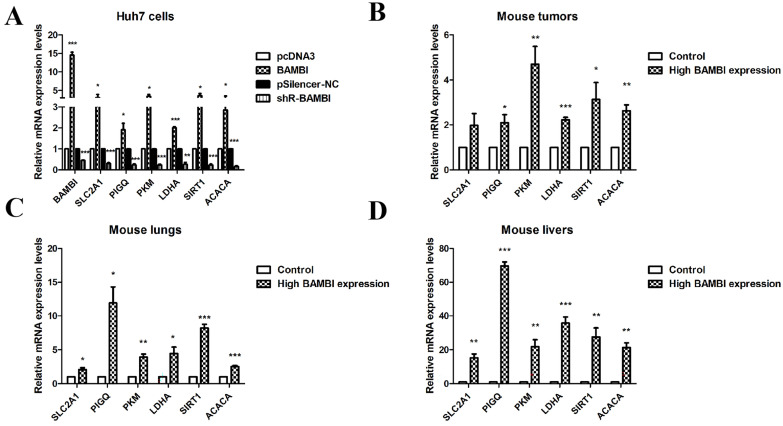
The expression levels of the six key genes in the glycolysis and lipid metabolism pathways in Huh7 cells and mice. (**A**) After overexpression (BAMBI) or knockdown (shR-BAMBI) of BAMBI in Huh7 cells, the mRNA levels of BAMBI, four key genes (SLC2A1, PIGQ, PKM, and LDHA) in the glycolysis pathway and two key genes (SIRT1 and ACACA) in the lipid metabolism pathway were detected. (**B**–**D**) The mRNA levels of BAMBI, SLC2A1, PIGQ, PKM, LDHA, SIRT1, and ACACA in mouse tumors (**B**), lungs (**C**), and livers (**D**). All experiments were performed at least in triplicate. Data are presented as mean ± standard deviation. * *p* < 0.05, ** *p* < 0.01, and *** *p* < 0.001 compared with the corresponding control group (BAMBI vs. pcDNA3 and shR-BAMBI vs. pSilencer-NC for Huh7 cells; high BAMBI expression vs. control for mouse tumors and tissues).

To further confirm the regulatory role of BAMBI on glycolysis and lipid metabolism, RT-qPCR was applied to examine the correlation between BAMBI with four key genes (SLC2A1, PIGQ, PKM, and LDHA) in the glycolysis pathway and two key genes (SIRT1 and ACACA) in the lipid metabolism pathway. Our results showed that overexpression or knockdown of BAMBI increased or decreased the expression levels of SLC2A1, PIGQ, PKM, LDHA, SIRT1, and ACACA in Huh7 cells (Figure 4A). Moreover, mice injected with high-BAMBI-expression Huh7 cells displayed significantly elevated expression levels of those genes in mouse tumors, as well as in metastatic liver and lung tissues, compared to the control group (Figure 4B–D). These results further corroborate our findings, suggesting that BAMBI is involved in the regulation of glycolysis and lipid metabolism in hepatocellular carcinoma (HCC).

### 2.4. BAMBI Is Associated with Immune Cell Infiltration in HCC

**Figure 5:**
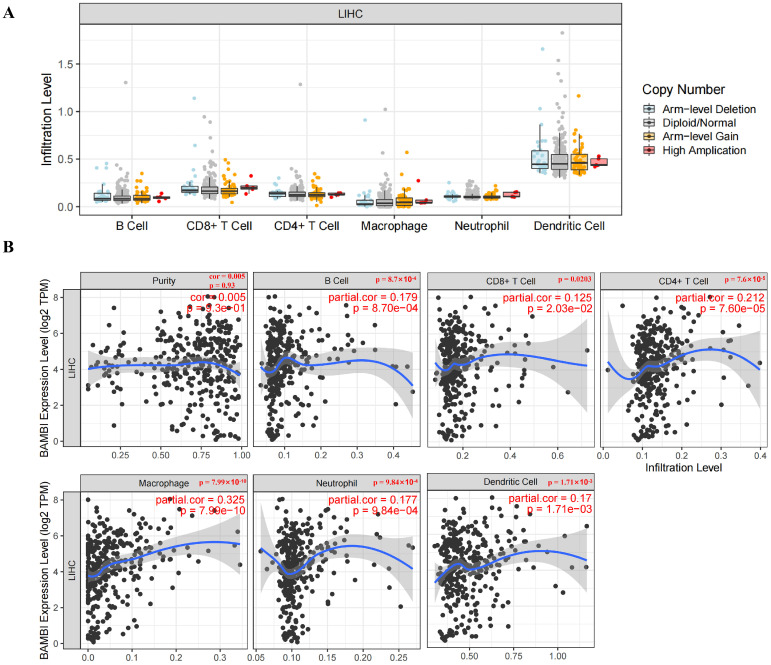
Relationships of BAMBI expression with immune cell infiltration in HCC. (**A**) Effects of different copies of BAMBI on different immune cell infiltration in HCC. (**B**) Correlations of BAMBI expression with the levels of infiltration by B cells, CD8+ T cells, CD4+ T cells, macrophages, neutrophils, and dendritic cells in HCC. The blue lines illustrated the correlation trends between BAMBI and the levels of immune cell infiltration. *p*-values < 0.05 were considered statistically significant.

Immune-associated cells in the tumor microenvironment (TME) play critical roles in the development and progression of HCC. To determine whether BAMBI is associated with tumor immune cell infiltration in HCC, the relationships between BAMBI expression and immune cell infiltration were evaluated. No significant relationships between the level of immune cell infiltration and BAMBI copy numbers were detected in HCC (Figure 5A). However, BAMBI expression levels positively correlated with B-cell, CD8+ T-cell, CD4+ T-cell, macrophage, neutrophil, and dendritic cell infiltration in HCC (Figure 5B).

**Figure 6:**
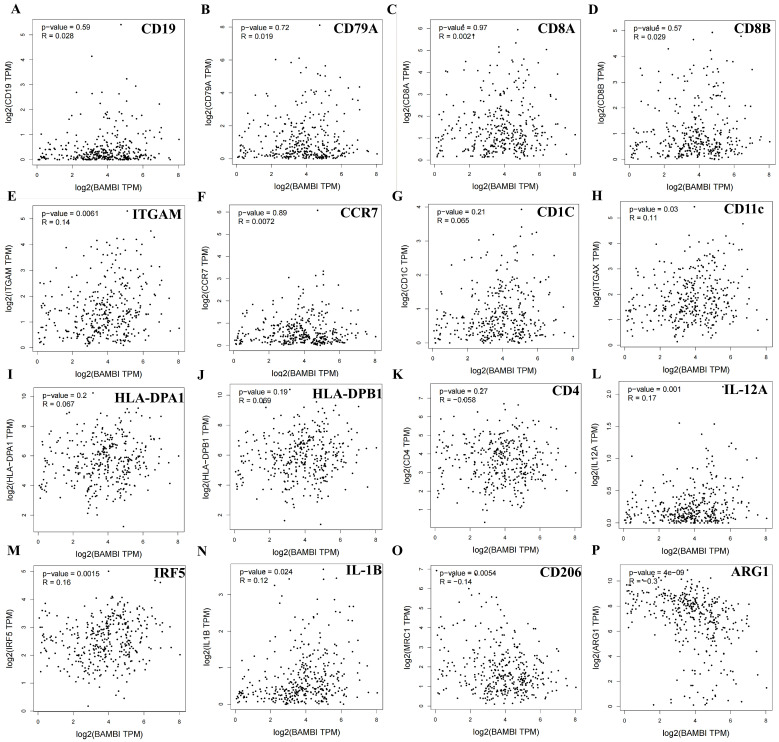
Relationships of BAMBI expression with biomarkers for immune cells in HCC. Correlations of BAMBI expression with the expression levels of biomarkers CD19 (**A**), CD79A (**B**), CD8A (**C**), CD8B (**D**), ITGM (**E**), CCR7 (**F**), CD1C (**G**), ITGAX (**H**), HLA-DPA1 (**I**), HLA-DPB1 (**J**), CD4 (**K**), CD11c (**L**), IRF5 (**M**), IL-12 (**N**), CD206 (**O**), and ARG-1 (**P**) in HCC. *p*-values < 0.05 were considered statistically significant.

To further characterize the role of BAMBI in tumor immunity, the relationships between BAMBI expression levels and the expression levels of biomarkers for B cells (cluster of differentiation [CD]19 and CD79A), CD8+ T cells (CD8A and CD8B), CD4+ T cells (CD4), neutrophils (ITGAM and CCR7), dendritic cells (CD1C, HLA-DPA1, and HLA-DPB1) and macrophages (CD11c, IRF5, and IL-12 for M1 macrophages; CD206 and Arg1 for M2 macrophages) were investigated in HCC. As shown in Figure 6A–K, no significant associations of BAMBI expression levels with biomarkers for B cells, CD8+ T cells, CD4+ T cells, neutrophils, or dendritic cells were detected. However, BAMBI expression levels were significantly and positively associated with CD11c, IRF5, and IL-12 (biomarkers for M1 macrophages; Figure 6L–N) and negatively associated with CD206 and Arg1 (biomarkers for M2 macrophages; Figure 6O,P). These results suggest that BAMBI is probably involved in macrophage infiltration and polarization, which may modulate the TME in HCC.

### 2.5. BAMBI Promotes Polarization of M1 Macrophages

**Figure 7:**
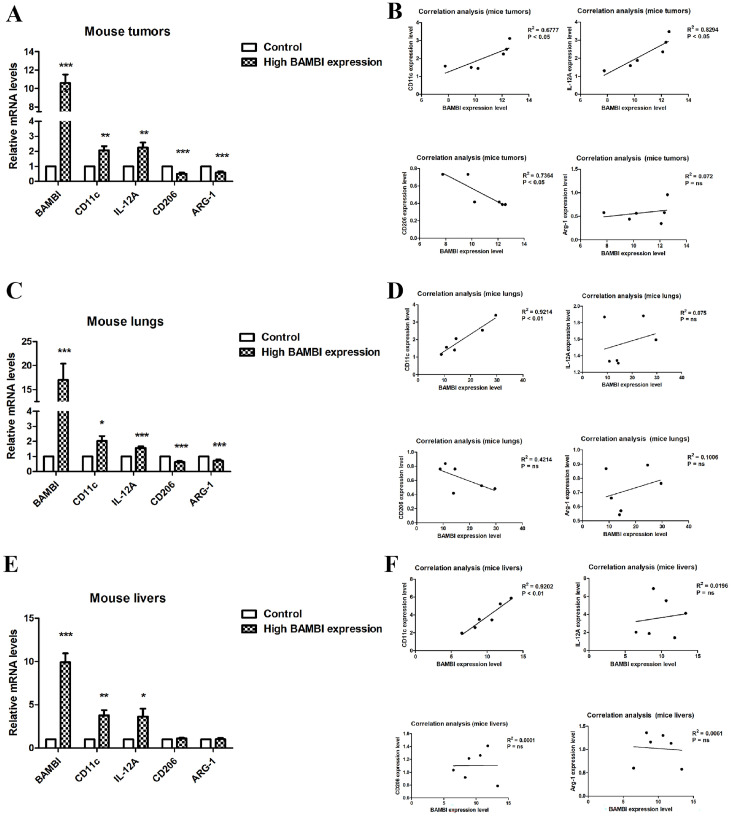
The expression levels and correlation of BAMBI and biomarkers for M1 and M2 macrophages in mouse tumors and tissues. (**A**,**C**,**E**) The RNA expression levels of BAMBI, CD11c, IL-12A, CD206, and ARG-1 in mouse tumors (**A**), lungs (**C**), and livers (**E**). (**B**,**D**,**F**) Correlation analysis between BAMBI expression and the levels of biomarkers for M1 and M2 macrophages in mouse tumors (**B**), lungs (**D**), and livers (**F**). All experiments were performed at least in triplicate. Data are presented as mean ± standard deviation. * *p* < 0.05, ** *p* < 0.01, and *** *p* < 0.001 compared with the control group (high BAMBI expression vs. control).

**Figure 8:**
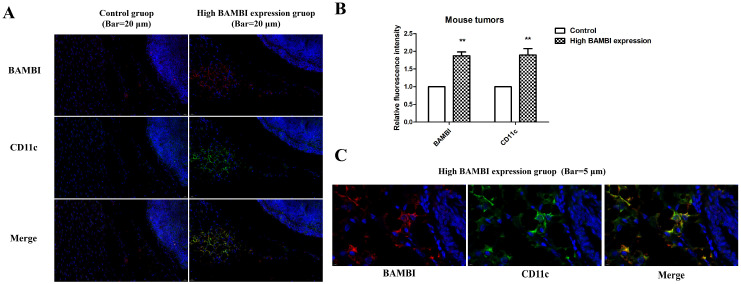
The expression and co-localization of BAMBI with CD11c in mouse tumors were determined by immunofluorescence staining. (**A**) The expression of BAMBI (red) and CD11c (green) in tumors of mice injected with high-BAMBI-expression Huh7 cells (high BAMBI expression) or the control Huh7 cells (Control). Nuclei are stained with DAPI (blue). Scale bar =20 µm. (**B**) Relative fluorescence intensity is expressed as the mean ± SD. **, *p* < 0.01 (high BAMBI expression vs. control). (**C**) The co-localization of BAMBI with CD11c in mouse tumors. Scale bar = 5 µm.

To investigate the role of BAMBI in macrophage infiltration and polarization in HCC, we examined the correlation between BAMBI expression and the levels of biomarkers for M1 and M2 macrophages in mouse tumors from the high-BAMBI-expression group, as well as in metastatic lungs and livers from the high-BAMBI-expression group. As illustrated in Figure 7A,C,E, compared to the control group, M1 markers such as CD11c and IL-12A were elevated in the tumors, lungs, and livers of mice with high BAMBI expression, while M2 markers (CD206 and ARG-1) were decreased in the tumors and tissues of these mice. Correlation analysis revealed that BAMBI positively correlated with CD11c and IL-12A (M1 markers) in mouse tumors, whereas it negatively correlated with CD206 (M2 marker) in mouse tumors (Figure 7B). In mouse lungs and livers, BAMBI positively correlated with CD11c, but no significant correlation was observed between BAMBI and other markers (Figure 7D,F). These findings suggest that BAMBI is probably involved in the positive regulation of M1 macrophage polarization but not in the process of M2 macrophage polarization. Furthermore, the immunofluorescence staining was applied to verify the expression and co-localization of BAMBI with CD11c in mouse tumors, in which CD11c was marked green, BAMBI was marked red, and DAPI was marked blue (Figure 8A,C). Results indicated that the expression levels of CD11c were higher in tumors from the high-BAMBI-expression group compared to the control group (Figure 8A,B). In addition, BAMBI was also found to be co-expressed on CD11c-positive cells in mouse tumors (Figure 8C).

Given that BAMBI may play different roles in the polarization of M1 and M2 macrophages at different stages of HCC. The correlation between the expression of BAMBI and M1 or M2 markers in HCC patients in different stages was analyzed. As shown in Figure S4, in HCC samples with T1 and T2 stages, BAMBI positively related to M1 markers while negatively related to M2 markers. However, in HCC samples with T3 and T4 stages, the significant positive or negative correlations between BAMBI and M1 or M2 markers disappeared. These results suggest that the polarization of macrophages by BAMBI may occur in the early stage of HCC rather than in the late stage.

**Figure 9:**
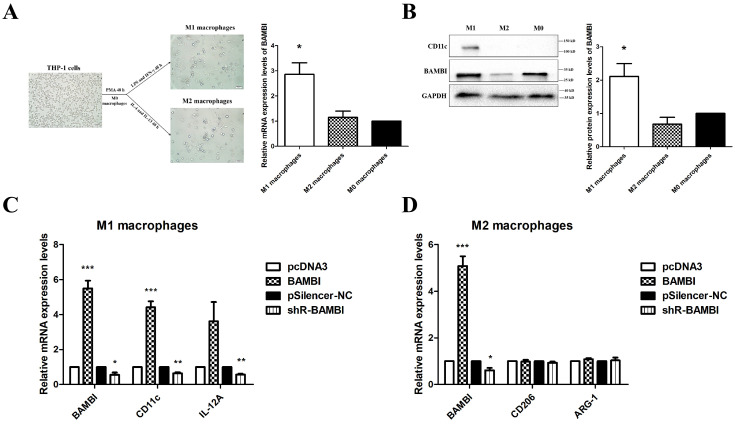
BAMBI contributed to M1 polarization of macrophages in vitro. (**A**,**B**) The mRNA (**A**) and the protein (**B**) expression levels of BAMBI and CD11c in M1, M2, and M0 macrophages. The samples were derived from the same experiment and the blots were processed in parallel. Data are presented as mean ± standard deviation. * *p* < 0.05, compared with M0 macrophages (M1 or M2 macrophages vs. M0 macrophages). (**C**,**D**) The differentiation of M1 and M2 macrophages was induced 48 h after overexpression or knockdown of BAMBI in THP-1 cells. The RNA expression levels of BAMBI, CD11c, and IL-12A in M1 macrophages (**C**) or BAMBI, CD206, and ARG-1 in M2 macrophages (**D**) were detected by RT-qPCR. All experiments were performed at least in triplicate. Data are presented as mean ± standard deviation. * *p* < 0.05, ** *p* < 0.01, and *** *p* < 0.001 compared with the corresponding control group (BAMBI vs. pcDNA3 and shR-BAMBI vs. pSilencer-NC).

To further investigate whether BAMBI influences macrophage polarization, we evaluated the expression levels of BAMBI in undifferentiated THP-1 cells (M0 macrophages), THP-1-derived M1 macrophages, and THP-1-derived M2 macrophages. When compared to M0 macrophages, higher BAMBI expression was observed in M1 macrophages, while no significant difference was observed in M2 macrophages (Figure 9A,B). Overexpression or knockdown of BAMBI in THP-1 cells resulted in increased or decreased expression levels of M1 macrophage markers following differentiation, while no significant difference was observed in the expression of M2 markers (Figure 9C,D). These results demonstrate that BAMBI promotes M1 polarization of macrophages both in vitro and in vivo.

### 2.6. Relationship Between BAMBI and Immune Checkpoints in HCC

**Figure 10:**
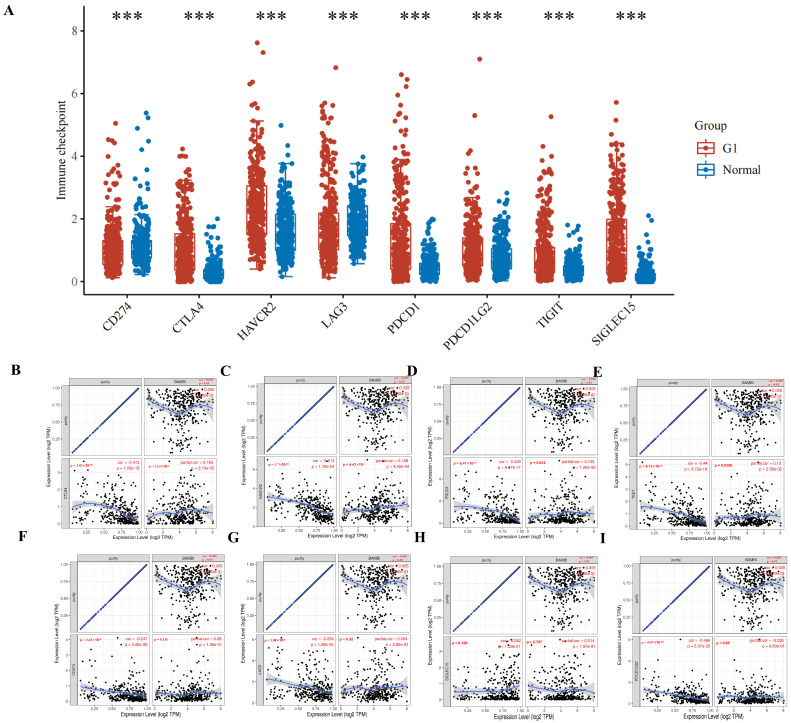
Relationships of BAMBI expression with immune checkpoints in HCC. (**A**) Distribution of immune checkpoint molecules expressed in HCC tissues and normal tissues. (**B**–**I**) Associations of BAMBI with CTLA–4 (**B**), HAVCR2 (**C**), PDCD1 (**D**), TIGIT (**E**), CD274 (**F**), LAG3 (**G**), SIGLEC15 (**H**), and PDCD1LG2 (**I**) in HCC. The blue lines depicted the trends of the correlation. *p*-values < 0.05 were considered statistically significant; *** *p* < 0.001.

Effective immunotherapy reportedly requires adequate immune cell infiltration in the TME and sufficient expression of immune checkpoint molecules [20]. BAMBI plays an oncogenic role in HCC and may modulate tumor immunity. Thus, the relationships between BAMBI and immune checkpoint molecules were evaluated in HCC. CD274, cytotoxic T lymphocyte-associated antigen 4 (CTLA-4), HAVCR2, LAG3, PDCD1, PDCD1LG2, TIGIT, and SIGLEC15 are important immune checkpoint molecules in HCC. As shown in Figure 10A, these molecules were expressed at significantly higher levels in HCC compared with the expression levels in normal tissues. Significant positive correlations between BAMBI expression and CTLA-4, HAVCR2, PDCD1, and TIGIT levels were detected in HCC, which was adjusted by purity based on a TIMER analysis (Figure 10B–E). However, no significant correlations between BAMBI expression and the other four immune checkpoint molecules were detected (Figure 10F–I). These results suggest that tumor immune escape tends to contribute to BAMBI-mediated carcinogenesis in HCC.

### 2.7. Prediction and Analysis of Genes That Interact with BAMBI

A protein–protein interaction network was constructed using the STRING database to identify genes that may interact with BAMBI. As depicted in Figure S5, the protein–protein interaction network for BAMBI consisted of 21 nodes and 70 edges with high-confidence interactions (interaction score > 0.7). The most connected genes with BAMBI with the top ten highest confidence included SMAD7, TGFBR1, BMPR1A, BMPR1B, ACVR1, FZD4, FZD5, FZD9, DVL2, and LRP6. These genes can form a module. Furthermore, the correlations between BAMBI and the 10 central genes in HCC tumor tissues were determined, as demonstrated in Figure S6A–J. BAMBI significantly correlated with SMAD7, ACVR1, BMPR1A, DVL2, TGFBR1, and FZD9, which are key modulators in the TGF-β and Wnt signaling pathways. These results suggest that BAMBI may play multiple roles in HCC by regulating genes in the TGF-β and Wnt signaling pathways.

## 3. Discussion

HCC is among the most common and deadliest types of cancer worldwide [21]. Numerous studies have explored the molecular mechanisms underlying the development and progression of HCC, with the goal of developing effective therapeutic strategies [22]. There is substantial evidence that the TME plays an important role in HCC by promoting tumor growth, invasion, and metastasis [23,24]. The TME, a complex and dynamic ecosystem surrounding cancer cells, consists of cancer-associated fibroblasts, immune cells, blood vessels, and ECM components [4,25]. Infiltrating immune cells such as tumor-associated macrophages (TAMs) are key components of the TME.

TAMs are recruited to the TME by tumor cells and other inflammatory mediators. Macrophages exhibit both pro-tumor and anti-tumor effects, depending on their polarization state and the specific context of the TME [26,27]. TAMs, a subset of white blood cells, are recruited to the TME by tumor cells and other inflammatory mediators [26,27]. TAMs can be divided into M1 and M2 subtypes [28,29]. M1 macrophages produce pro-inflammatory cytokines (e.g., tumor necrosis factor-α and IL-6) and induce anti-tumor immune responses [30]. In addition, M1 macrophages produce reactive oxygen species and nitric oxide, which can directly kill tumor cells or indirectly cause tumor cell death by activating other immune cells. Conversely, M2 macrophages produce anti-inflammatory cytokines (e.g., IL-10 and TGF-β) and promote tumor growth and metastasis. For example, M2 macrophages produce arginase-1 (Arg1), which inhibits M1 macrophage activity and promotes tumor immune evasion [31,32]. High numbers of TAMs are often found in the TME of HCC. These TAMs promote tumor growth and survival through several mechanisms, including provision of nutrients, induction of angiogenesis, remodeling of the ECM, facilitation of immune evasion, and promotion of EMT [33,34]. Therefore, cancer therapies targeting TAMs in the TME have emerged as promising treatment strategies.

Metabolic reprogramming is a hallmark of cancer that contributes to tumorigenesis and disease progression [35]. Cancer cells rewire metabolic pathways to meet their needs for adenosine triphosphate production, biomass generation, and redox balance. Glycolysis is a metabolic pathway that converts glucose into pyruvate, even in the absence of oxygen [36]. This conversion is essential for cells to maintain readily accessible energy; it is particularly important for rapidly dividing cells, such as cancer cells [37,38]. Cancer cells often exhibit increased glycolysis activity, even in the presence of oxygen; this phenomenon is known as the Warburg effect [39]. HK2, PFKFB3, and PKM2 have been identified as key glycolysis enzymes that participate in glucose metabolism; they can enhance glucose uptake and lactate production, thereby providing a source of energy for cancer cells [40,41]. Additionally, glycolysis can promote tumor growth by providing substrates for macromolecule synthesis and promoting angiogenesis [42].

Lipid metabolism is another important metabolic pathway in HCC [43,44]. Cancer cells often exhibit altered lipid metabolism, which can support tumor growth and survival. For example, cancer cells can increase fatty acid uptake from the bloodstream and then use β-oxidation to extract energy from the acquired fatty acids [45]. Additionally, lipid metabolism can promote tumor growth by providing substrates for the synthesis of membrane components and signaling molecules [43]. Therefore, glycolysis and lipid metabolism both play important roles in the development and progression of HCC. An understanding of these mechanisms is needed to develop effective therapeutic strategies for HCC.

Previous studies showed that BAMBI contributes to the development and progression of multiple cancers [15,16,17]. In this study, we analyzed the expression profiles of BAMBI, along with its contributions to pathological findings, metastasis characteristics, and prognosis in multiple human cancers. The results showed that the expression levels of BAMBI were increased in BRCA, CHOL, COAD, ESCA, KIRP, HCC, READ, and STAD, whereas they were decreased in LUAD, KICH, and KIRC; thus, BAMBI presumably plays important roles in carcinogenesis for these 11 types of cancers. Analyses of the contributions of BAMBI to overall survival, disease-free survival, tumor grade, and nodal metastasis in the 11 types of cancers showed that higher BAMBI expression was associated with poor prognosis and greater nodal metastasis in HCC, suggesting that BAMBI can serve as a diagnostic biomarker and an indicator of unfavorable prognosis in HCC.

Our previous studies revealed that BAMBI, as a target of miR-HCC2, contributed to metastasis and stemness properties in HCC cells by activating the Wnt/β-catenin signaling pathway through the nuclear translocation of β-catenin [18,19]. To better understand the signaling pathways in HCC with BAMBI involvement, we evaluated the correlations of BAMBI with 16 cell signaling pathways in HCC. The results showed that BAMBI was positively correlated with tumor proliferation, EMT markers, MYC targets, and cellular response to hypoxia in HCC; it was negatively correlated with glycolysis/gluconeogenesis and fatty acid degradation pathways. We wondered why BAMBI, as an indicator of poor prognosis in HCC, exhibited negative regulation of glycolipid metabolism pathways. Therefore, we analyzed BAMBI’s correlation with over 100 genes related to glycolipid metabolism individually. Interestingly, we found that BAMBI was positively correlated with the majority of genes in glycolipid metabolism, while only a few genes showed a negative correlation. Further experiments indicated that BAMBI was positively correlated with key genes (SLC2A1, PIGQ, PKM, LDHA, SIRT1, and ACACA) for glycolipid metabolism in Huh7 cells and mouse tumors and tissues. These findings suggest that BAMBI is involved in the regulation of glycolipid metabolism processes in HCC, but its specific regulatory mechanisms still require further experimental exploration.

The liver is the largest peripheral immunomodulatory organ in the human body; it contains the largest populations of resident macrophages (Kupffer cells) and natural killer cells, natural killer T cells, CD8+ T cells, and CD4+ T cells. There is increasing evidence that immune-associated cells in the TME have important roles in the development and progression of HCC [4,5,46]. In the present study, we analyzed the relationships of BAMBI expression levels with infiltration by various immune cells; the results showed that BAMBI expression was positively associated with CD11c, IRF5, and IL-12 (biomarkers for M1 macrophages), whereas it was negatively associated with CD206 and Arg1 (biomarkers for M2 macrophages). To investigate the role of BAMBI in macrophage infiltration and polarization in HCC, we examined the correlation between BAMBI expression and the levels of biomarkers for M1 and M2 macrophages in mice and the effect of BAMBI on the differentiation of M1 and M2 macrophages. The data showed that BAMBI positively correlated with M1 markers in mouse tumors and tissues. Moreover, BAMBI negatively correlated with M2 markers in mouse tumors, but no significant correlation was observed between BAMBI and M2 markers in mouse lungs or livers. Moreover, the immunofluorescence staining results showed that CD11c was increased in tumors from the high-BAMBI-expression group compared to the control group, and BAMBI was co-expressed with CD11c-positive cells in mouse tumors. In vitro, higher BAMBI expression was observed in M1 macrophages compared to M0 macrophages, and overexpression or knockdown of BAMBI in THP-1 cells increased or decreased the levels of M1 markers after differentiation. These findings demonstrate that BAMBI promotes M1 polarization of macrophages both in vitro and in vivo. Moreover, the correlation between the expression of BAMBI and M1 or M2 markers in HCC patients in different stages were analyzed, and the results suggested that the polarization of macrophages by BAMBI may occur in the early stage of HCC rather than in the late stage. These findings suggested that BAMBI contributes to the modulation of macrophage polarization and regulation of the TME in HCC.

Novel drugs targeting immune checkpoint molecules have achieved good cancer treatment outcomes [20,47]. Immune checkpoints are pathways that include stimulatory and inhibitory checkpoint molecules. Inhibitory checkpoint molecules, such as CTLA-4, programmed cell death protein-1 (PD-1), and programmed cell death ligand 1 (PD-L1), can suppress anti-tumor immune responses in solid tumors [20]. BAMBI acts as an oncogene in HCC and likely participates in tumor immunity modulation. Thus, we assessed the correlation of BAMBI with immune checkpoints in HCC. BAMBI positively correlated with CTLA-4, HAVCR2, PDCD1, and TIGIT, which are important immune checkpoint molecules in HCC. Thus, BAMBI may be involved in tumor immune escape in HCC.

We used the STRING database to predict interactions between BAMBI and other proteins in various pathways and identify new targets for HCC treatment. We also sought to elucidate the mechanisms by which BAMBI contributes to tumor growth and progression. The genes most strongly connected with BAMBI were SMAD7, ACVR1, BMPR1A, DVL2, TGFBR1, and FZD9. These genes modulate the TGF-β and Wnt signaling pathways [48,49,50,51,52,53,54]. The TGF-β pathway plays a central role in inflammation, fibrogenesis, and immunomodulation during the development and progression of HCC, and as HCC progresses, TGF-β undergoes a functional switch from being a tumor suppressor to a promoter [55,56,57]. Additionally, the TGF-β pathway is involved with tumor immune evasion and poor responses to cancer immunotherapy [55]. Combinations of TGF-β inhibitors and immune checkpoint inhibitors have been successfully applied to induce complete responses to treatment in mouse cancer models [58,59]. The Wnt signaling pathway plays an important role in the regulation of processes such as cell proliferation, survival, migration, polarization, embryonic development, cell fate specification, and self-renewal of stem cells [60,61]. Dysregulated Wnt signaling contributes to proliferation, migration, and invasion in HCC [62]. Approximately 95% of patients with HCC display aberrant activation of the Wnt signaling pathway [63]. Moreover, recent studies have revealed that the TGF-β and Wnt signaling pathways are both involved in lipid metabolism reprogramming and glycolysis across multiple models [64,65,66,67]. Our results suggest that BAMBI plays complex roles in HCC by regulating genes in the TGF-β and Wnt signaling pathways.

Generally, although BAMBI plays a positive role in promoting M1 macrophage polarization, its function as an oncogene cannot be overlooked. Understanding the dual role of BAMBI in tumor biology is crucial for developing new treatment strategies and improving patient outcomes.

## 4. Materials and Methods

### 4.1. Data Acquisition, Processing, and Analysis

The mRNA expression data of the following 33 cancers were downloaded from The Cancer Genome Atlas (TCGA) database (https://genome-cancer.ucsc.edu/, accessed on 12 January 2024): adrenocortical carcinoma (ACC), bladder urothelial carcinoma (BLCA), breast invasive carcinoma (BRCA), cervical squamous cell carcinoma and endocervical adenocarcinoma (CESC), cholangiocarcinoma (CHOL), colon adenocarcinoma (COAD), lymphoid neoplasm diffuse large B-cell lymphoma (DLBC), esophageal carcinoma (ESCA), glioblastoma multiforme (GBM), head and neck squamous cell carcinoma (HNSC), kidney chromophobe (KICH), kidney renal clear cell carcinoma (KIRC), kidney renal papillary cell carcinoma (KIRP), acute myeloid leukemia (LAML), brain lower-grade glioma (LGG), liver hepatocellular carcinoma (LIHC, also known as HCC), lung adenocarcinoma (LUAD), lung squamous cell carcinoma (LUSC), mesothelioma (MESO), ovarian serous cystadenocarcinoma (OV), pancreatic adenocarcinoma (PAAD), pheochromocytoma and paraganglioma (PCPG), prostate adenocarcinoma (PRAD), rectal adenocarcinoma (READ), sarcoma (SARC), skin cutaneous melanoma (SKCM), stomach adenocarcinoma (STAD), testicular germ cell tumors (TGCT), thyroid carcinoma (THCA), thymoma (THYM), uterine corpus endometrial carcinoma (UCEC), uterine carcinosarcoma (UCS), and uveal melanoma (UVM). Subsequently, the data were normalized and subjected to differential expression analysis focused on BAMBI, using the limma package [68] in R software. *p*-values < 0.05 were considered statistically significant.

### 4.2. Gene Expression Profiling Interactive Analysis (GEPIA) Database Analysis

GEPIA (http://gepia.cancer-pku.cn/, accessed on 12 January, 7 March and 4 November 2024) is an online database for cancer and normal gene expression profiling and interactive analyses based on TCGA and the Genotype-Tissue Expression (GTEx) project data. GEPIA was used to explore BAMBI expression in various types of human cancers (*p*-values < 0.05 were considered statistically significant) [69]. The relationships of overall survival and disease-free survival associated with BAMBI expression in 20 cancers were determined using the GEPIA database, along with the prognostic values of BAMBI expression in HCC. Log-rank *p*-values < 0.05 were considered statistically significant. Additionally, the relationships of BAMBI expression with immune cell biomarkers, immune checkpoints, and candidate interacting genes in HCC were examined using the GEPIA database. |R|-values > 0.1 and *p*-values < 0.05 were used as selection criteria for identifying statistically significant associations.

### 4.3. UALCAN Database Analysis

UALCAN (http://ualcan.path.uab.edu/analysis.html, accessed on 29 November 2023) is an online server for analyzing differential expression in tumor and normal tissues across tumor stages and lymph node metastasis statuses and in relation to other relevant clinical parameters based on TCGA transcriptomic and clinical data [70]. We used the UALCAN database to analyze differences in BAMBI expression among multiple types of cancer tissues and normal tissues, then performed correlation analysis focused on the interactions of BAMBI expression with pathological stage, tumor grade, and lymph node metastasis in KIRC and HCC.

### 4.4. TIMER Database Analysis

TIMER (version 2.0, https://cistrome.shinyapps.io/timer/, accessed on 12 January 2024) is a tool for comprehensive analysis of tumor-infiltrating immune cells [71]. TIMER was utilized to analyze correlations of BAMBI expression with immune cell infiltration and immune checkpoints in HCC. *p*-values < 0.05 were considered statistically significant.

### 4.5. Analysis of the Correlations Between BAMBI and Cell Signaling Pathways in HCC

RNA-sequencing expression (level 3) profiles and corresponding clinical information for HCC were downloaded from the TCGA dataset (https://portal.gdc.cancer.gov/, accessed on 12 January 2024). R software GSVA package was used to analyze, choosing parameter as method = ‘ssgsea’. The correlation between genes and pathway scores was analyzed using Spearman correlation. All the analysis methods and R packages were implemented by R version 4.0.3. *p*-values < 0.05 was considered statistically significant.

### 4.6. STRING Database

STRING (version 12.0, https://cn.string-db.org, accessed on 29 November 2023) is a database containing 24,584,628 proteins from 5090 organisms; it integrates known and predicted interactions among more than 932,000,000 proteins from multiple organisms, including Homo sapiens [72]. The STRING was used to analyze the interacting protein network for BAMBI with the options “Multiple proteins” option and “Homo sapiens”. *p*-values < 0.05 were considered statistically significant, and an interaction score > 0.7 was regarded as a high-confidence interaction.

### 4.7. Cell Cultures and Transfections

Huh7 and THP-1 cells were purchased from The Cell Bank of Type Culture Collection of the Chinese Academy of Sciences and authenticated by short tandem repeat profiling. Huh7 cells were maintained in Dulbecco’s modified Eagle medium (DMEM) (Gibco; Thermo Fisher Scientific, Inc.; Waltham, MA, USA) with 10% fetal bovine serum (Gibco; Thermo Fisher Scientific, Inc.), 20 mM 4-(2-hydroxyethyl)-1-piperazineethanesulfonic acid, 100 units/mL penicillin and 100 g/mL streptomycin. THP-1 cells were maintained in Roswell Park Memorial Institute medium (RPMI 1640, Gibco; Thermo Fisher Scientific, Inc.) containing 10% fetal bovine serum (Gibco; Thermo Fisher Scientific, Inc.), 100 units/mL penicillin, 100 g/mL streptomycin and 50 pM β-mercaptoethanol (Gibco; 31350–010). The cells were incubated at 37 °C in a humidified atmosphere with 5% CO_2_. Advanced DNA RNA Transfection Reagent (ZETA LIFE; San Francisco, CA, USA) was used for all transfections in this study, according to the manufacturer’s protocol. Huh7 cells were collected 48 h after transfection, and cell samples were collected for reverse transcription-quantitative PCR (RT-qPCR) and Western blotting detection.

### 4.8. Vector Construction

The overexpression vector (BAMBI) and the silencing (shR-BAMBI) vector for BAMBI have been constructed as described in our previous study [19]. BAMBI was constructed by the respective coding sequences of BAMBI amplified by PCR from the cDNA of Huh7 cells and then inserted into the pcDNA3 vector between the BamHI and XhoI sites. For shR-BAMBI, the synthesized oligonucleotides were annealed and ligated into the pSilencer2.1/neo vector (pSilencer-NC; Ambion, Thermo Fisher Scientific, Inc.; Waltham, MA, USA) between the BamHI and HindIII sites.

### 4.9. Macrophage Differentiation

THP-1 cells were stimulated with 100 ng/mL phorbol-12-myristate-13-acetate (PMA; cat no. P33390; ACMEC; Shanghai, China) for 48 h and termed as M0 macrophages in this study [73]. For M1 activation, THP1-derived macrophages were further treated with 100 ng/mL lipopolysaccharides (LPS; cat no. AC12037; ACMEC) and 20 ng/mL human IFN-γ (cat no. AC13071; ACMEC) for 48 h, and termed for M1 macrophages. For M2 activation, THP1-derived macrophages were further treated with 20 ng/mL human IL-13 (cat no. HY-P7033; MCE; Monmouth Junction, NJ, USA) and 20 ng/mL IL-4 (cat no. AC13064; ACMEC) for 48 h, and termed for M2 macrophages. Undifferentiated THP-1 cells (M0), as well as M1- and M2-polarized macrophages, were harvested for subsequent RT-qPCR and Western blot analyses. These assays were conducted to determine the expression levels of BAMBI across these three cell types. To assess the impact of BAMBI on macrophage polarization, THP-1 cells were initially transfected with 4 μg of pcDNA3, BAMBI, pSilencer-NC or shR-BAMBI and subsequently incubated for 48 h at 37 °C. Following this incubation period, the cells were treated with PMA for an additional 48 h to induce differentiation before being subjected to M1 or M2 activation protocols and then collected following RT-qPCR and Western blot analyses.

### 4.10. RT-qPCR

Total RNA was extracted from tissue samples and cells using TRIzol reagent (Invitrogen, Carlsbad, CA, USA), according to the manufacturer’s protocols. RNA (2 μg) was reverse transcribed into complementary DNA using Moloney Murine Leukemia Virus (M-MLV) reverse transcriptase (Promega Corporation; Madison, WI, USA) for 60 min at 42 °C, 15 min at 70 °C, and then on ice. The subsequent qPCR amplification was performed using a SYBR Premix Ex Taq™ Kit (Promega Corporation; Wisconsin, USA) as follows: 95 °C for 3 min, followed by 40 cycles of denaturation at 95 °C for 10 s, annealing at 60 °C for 30 s and extension at 72 °C for 30 s. GAPDHs were used as internal controls. Relative mRNA expression was calculated using the 2^−ΔΔCq^ method [74]. Detailed sequences of all primers are presented in Table S1.

### 4.11. Western Blotting

Cellular proteins were extracted using radioimmunoprecipitation assay lysis buffer (Beijing Solarbio Science & Technology Co., Ltd.; Tongzhou Dist., Beijing, China), and protein concentrations were quantified using a BCA protein assay kit (cat no. P0010S; Beyotime Institute of Biotechnology; Haimen, China) according to the manufacturer’s protocols. Equal amounts (10 µg/lane) of protein samples were separated using 10% SDS-PAGE and transferred onto polyvinylidene fluoride membranes. Membranes were blocked with 5% non-fat milk for 1 h at room temperature and incubated at 4 °C with primary antibodies overnight as follows: BAMBI (1:1000; cat no. 680053; Chengdu Zhengneng Biotechnology Co., Ltd.; China), CD11c (1:1000; cat no. R380832; Chengdu Zhengneng Biotechnology Co., Ltd., Chengdu, China), GAPDH (1:5000; cat no. 380626; Chengdu Zhengneng Biotechnology Co., Ltd.). The membranes were washed three times using TBST (0.05% Tween-20) and incubated with horseradish peroxidase (HRP)-conjugated secondary antibodies (1:10,000; cat no. 511203; Chengdu Zhengneng Biotechnology Co., Ltd.) for 1 h at room temperature and visualized using a Pierce ECL Western Blotting kit (Pierce; Thermo Fisher Scientific, Inc., Waltham, MA, USA). The protein band images were quantified with Image J (version 1.48; National Institutes of Health, Bethesda, MD, USA). GAPDH was used as an endogenous control for the normalization of the expression levels of target proteins.

### 4.12. Mouse Experiments

Our previous studies showed that BAMBI was a direct target gene of miR-HCC2, and its overexpression could be induced by miR-HCC2 [19]. After transfection of Huh7 cells with the miR-HCC2 overexpression plasmid (constructed in our previous study) or its control vector, the expression level of BAMBI in Huh7 cells with overexpressed miR-HCC2 was significantly higher than in those transfected with the control vector plasmid. The above two groups in this study are referred to as the high-BAMBI-expression Huh7 cells and the control Huh7 cells, respectively.

Subsequently, 6-week-old (with an average weight of 18 g) or 5-week-old female (with an average weight of 16 g) BALB/c nude mice (Beijing Vital River Laboratory Animal Technology Co., Ltd., Beijing, China) were inoculated subcutaneously or via the tail vein with either high-BAMBI-expression Huh7 cells or the control Huh7 cells (1 × 10^6^ Huh7 cells for the 6-week-old subcutaneously inoculated mice and 1 × 10^5^ Huh7 cells for the 5-week-old tail vein injection mice), as described in our previous study [19]. Mice inoculated with high-BAMBI-expression Huh7 cells and control Huh7 cells were referred to as the high-BAMBI-expression group and the control group in this study. The mice kept under standard laboratory conditions (temperature 22 ± 2 °C; relative humidity 50 ± 10%; 12 h light/dark cycle) with access to food and water ad libitum. The health and behavior of mice were monitored every day. The volume of each tumor in subcutaneously inoculated mice was assessed on once in the first week and then twice a week as follows: Length × width 2 × 1/2. At, 49 days after implantation, mice were sacrificed by cervical dislocation and the tumors were collected for RT-qPCR and immunofluorescence analyses. The mean tumor weight and mean tumor volume in the high-BAMBI-expression group were significantly greater than in the control group [19]. Additionally, the mice with tail vein injections were euthanized by cervical dislocation at 11 weeks post-injection, and their lungs and livers were then harvested for RT-qPCR detection. The injection of high-BAMBI-expression Huh7 cells led to significantly increased tumor incidence in vivo, compared with the injection of control Huh7 cells (two of six mice with high BAMBI expression developed lung and liver metastases, whereas no mice in the control group developed lung or liver metastases). The tumors were fixed with 10% formalin at room temperature for 72 h and embedded in paraffin. The expression levels and co-localization of CD11c and BAMBI were evaluated using immunofluorescence staining. The paraffin embedded sections (3 µm) underwent sequential dewaxing, hydration, antigen retrieval, and blocking. Slides were incubated overnight at 4 °C with primary antibodies against CD11c (conjugated Alexa Fluor^TM^ 488; 1:100; cat no. # 53-0114-82; Invitrogen Co., Ltd.; Waltham, MA, USA) and BAMBI (1:1000; cat no. 680053; Chengdu Zhengneng Biotechnology Co., Ltd.), followed by incubation with the secondary antibody (CY3-labeled goat anti-rabbit, 1:400; cat no. ab150078; Abcam; Cambridge, UK) for 1 h at room temperature. Images of the sections were captured under a light microscope (40× magnification; Olympus Corporation; Tokio, Japan) and analyzed using Caseviewer 2.4 (3DHISTECH, Ltd.; Budapest, Hungary). The fluorescence intensity and area of all the images acquired were measured using Image J (version 1.48; National Institutes of Health), and the mean fluorescence intensity of each image was calculated. Symptoms, including loss of appetite, ≥20% body weight loss, weakness, pain, breathing difficulties, or tumor volume reaching 2000 mm^3^, were set as humane endpoints for the present study. However, no mice were sacrificed before the end of tumor xenograft experiments due to display of any of these symptoms. All studies were performed with approval from the Animal Ethics Committee of Tianjin Medical University (approval no. TMULA-201954).

### 4.13. Statistical Analysis

Statistical analyses in this study were automatically calculated using the online databases mentioned above. *p*-values < 0.05 and log-rank *p*-values < 0.05 were considered statistically significant. GraphPad Prism 5 (GraphPad Software; Dotmatics; La Jolla, CA, USA) was used for statistical analysis. Each experiment was repeated in triplicate. All values were presented as mean ± standard deviation. Differences between the two groups were evaluated using a two-tailed Student’s *t*-test. Gene expression relationships were assessed using Pearson correlation coefficients. *p* < 0.05 was considered to indicate a statistically significant difference.

## 5. Conclusions

Our analysis of expression profiles and prognostic values demonstrated the potential roles of BAMBI in the development and progression of various human cancers. In particular, the upregulation of BAMBI was associated with poor prognosis in HCC. Further analysis revealed that BAMBI was strongly correlated with tumor proliferation, EMT markers, MYC targets, cellular response to hypoxia, glycolysis/gluconeogenesis, fatty acid degradation pathways, as well as regulating immune checkpoints in HCC. In vitro and in vivo experiments showed that BAMBI promoted polarization of M1 macrophages and is linked to the expression of key genes involved in glycolipid metabolism. The analysis of interacting genes suggested that BAMBI plays complex roles in HCC by regulating genes in the TGF-β and Wnt signaling pathways. However, the complex regulatory networks of BAMBI in HCC require further experimental and clinical validation in future studies. Our study has provided a comprehensive analysis of the roles of BAMBI in HCC, along with possible molecular mechanisms underlying its effects. Our findings elucidated that BAMBI was a prognostic biomarker and associated with macrophage polarization, glycolysis, and lipid metabolism in HCC.

## Data Availability

The datasets presented in this study can be found in online repositories. The names of the repository/repositories and accession number(s) can be found in the article/Supplementary Materials.

## Supplementary Materials

The following supporting information can be downloaded at https://www.mdpi.com/article/10.3390/ijms252312713/s1.
