# Supplementary material for: BAMBI Is a Prognostic Biomarker Associated with Macrophage Polarization, Glycolysis, and Lipid Metabolism in Hepatocellular Carcinoma
Source: Int J Mol Sci. 2024 Nov 26;25(23):12713. doi: 10.3390/ijms252312713 (PMC11640931; doi:10.3390/ijms252312713)
Supplement: Supplementary file 1 [file ijms-25-12713-s001.zip › Correlation of BAMBI and 17 genes in the fatty acid biosythesis pathway.pdf]

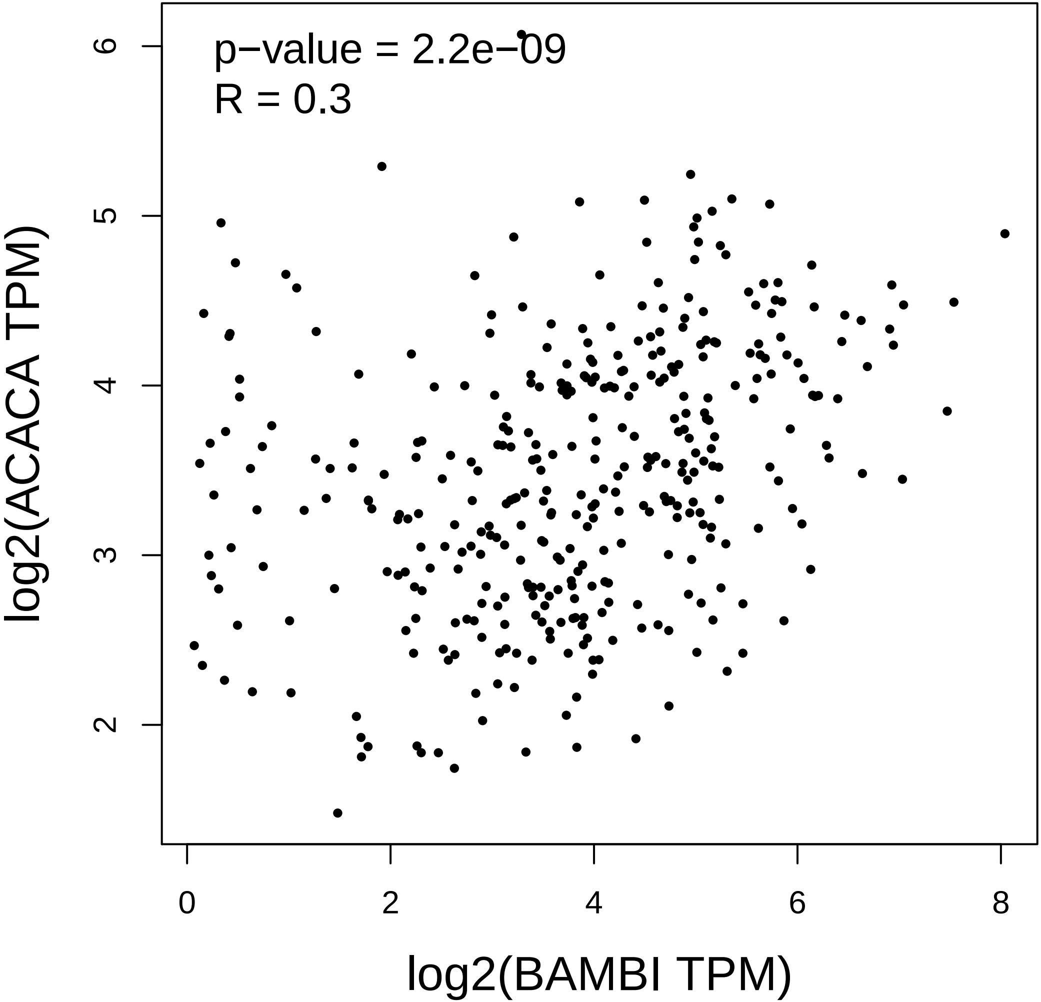

p−value = 2.2e−09
R = 0.3
log2(ACACA TPM)
log2(BAMBI TPM)
0
2
4
6
8
2
3
4
5
6

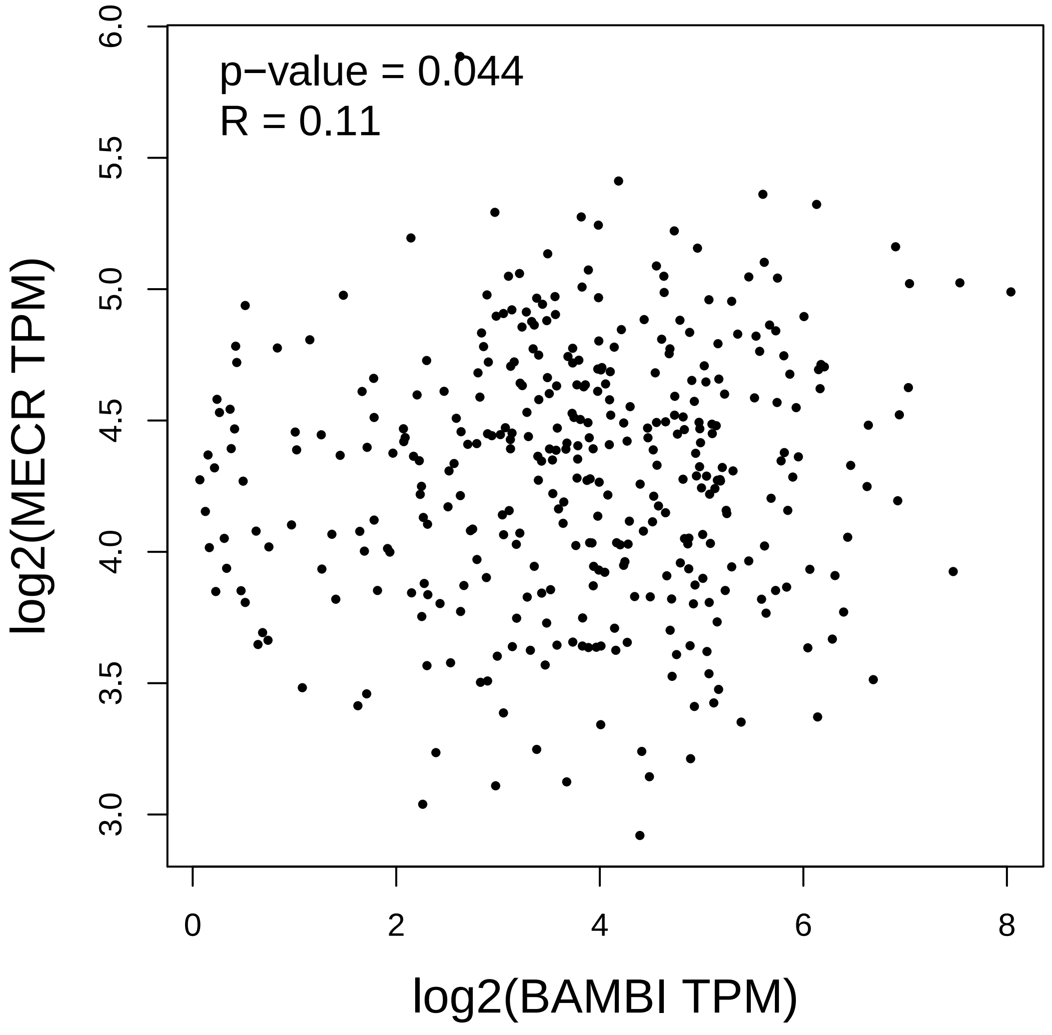
p−value = 0.044
R = 0.11
log2(MECR TPM)
log2(BAMBI TPM)
6.0
5.5
5.0
4.5
4.0
3.5
3.0
0
2
4
6
8

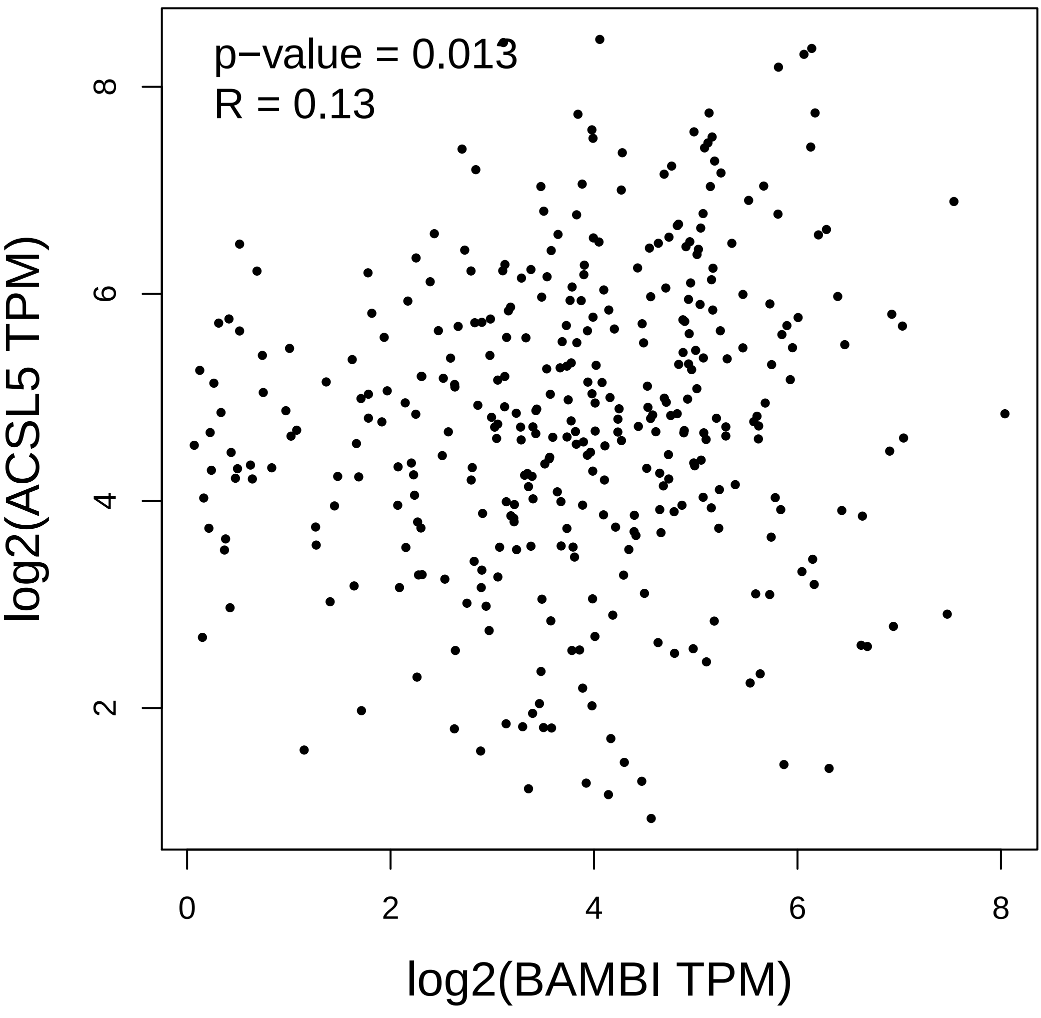
p−value = 0.013
R = 0.13
log2(ACSL5 TPM)
log2(BAMBI TPM)
2
4
6
8
0
2
4
6
8

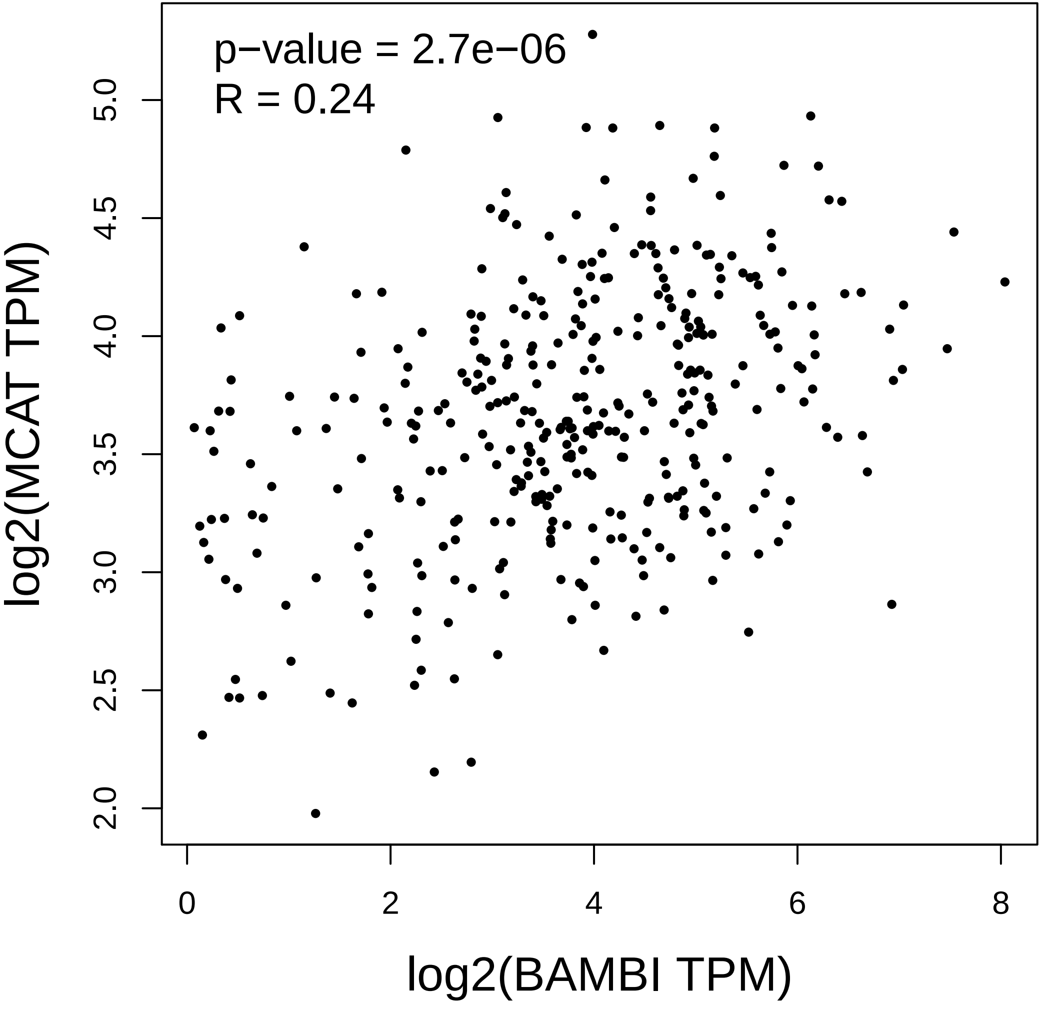
p−value = 2.7e−06
R = 0.24
log2(MCAT TPM)
5.0
4.5
4.0
3.5
3.0
2.5
2.0
0
2
4
6
8
log2(BAMBI TPM)

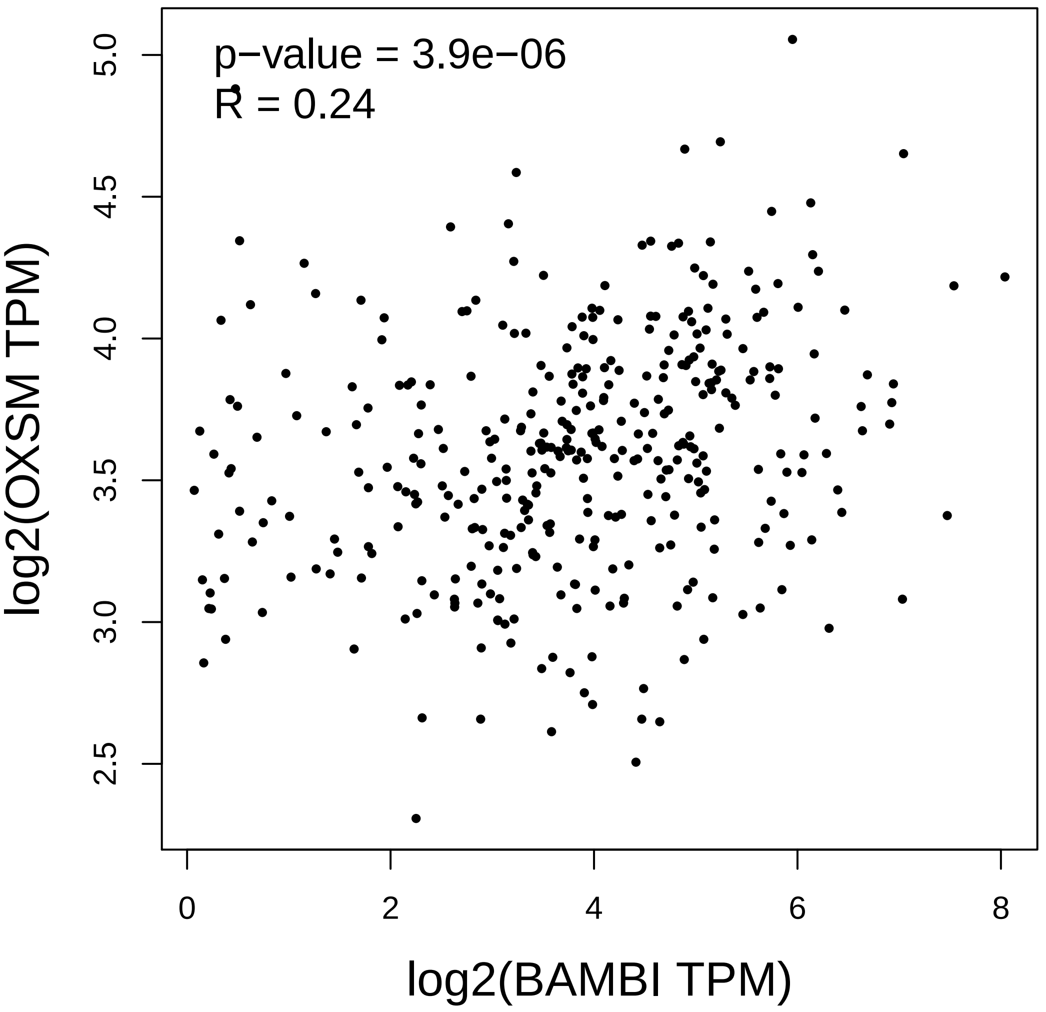
p−value = 3.9e−06
R = 0.24
log2(OXSM TPM)
5.0
4.5
4.0
3.5
3.0
2.5
0
2
4
6
8
log2(BAMBI TPM)

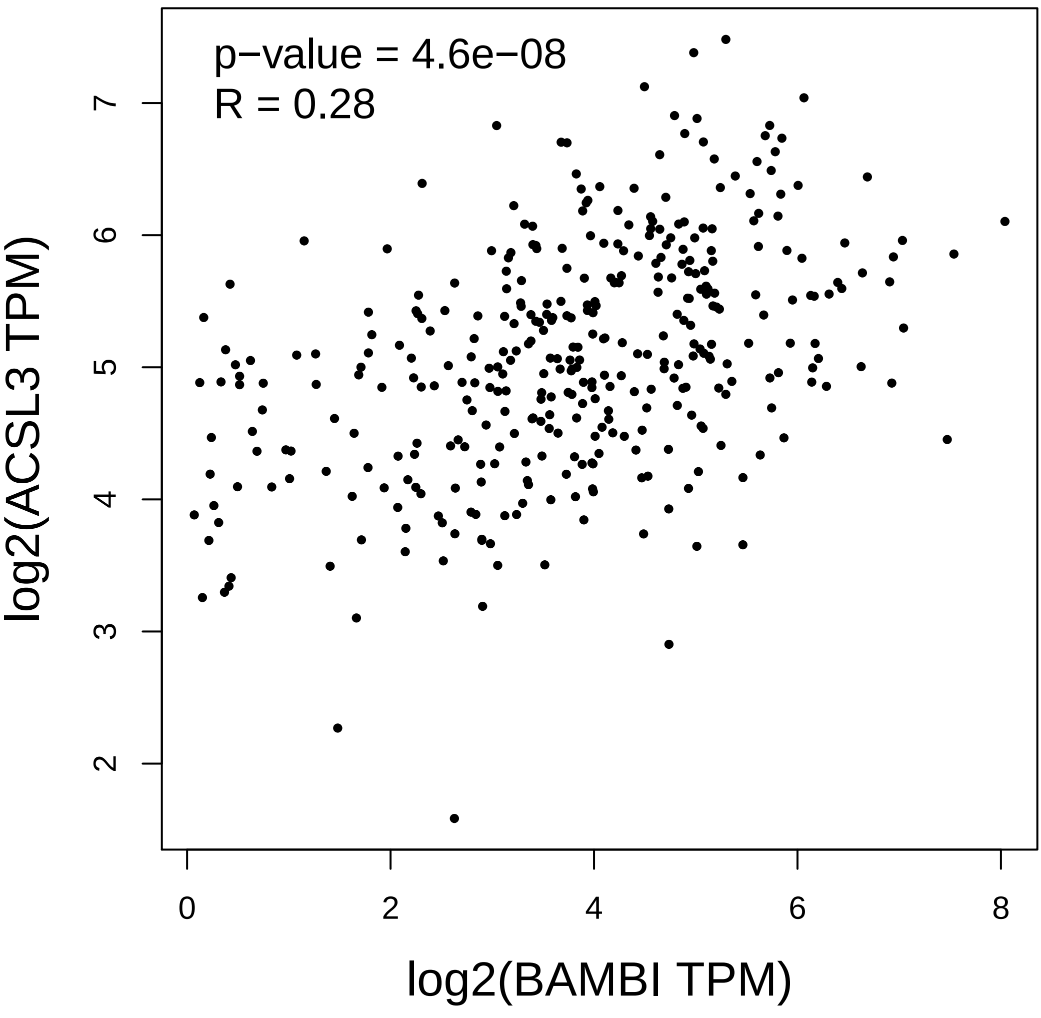

p−value = 4.6e−08
R = 0.28
log2(ACSL3 TPM)
log2(BAMBI TPM)
2
3
4
5
6
7
0
2
4
6
8

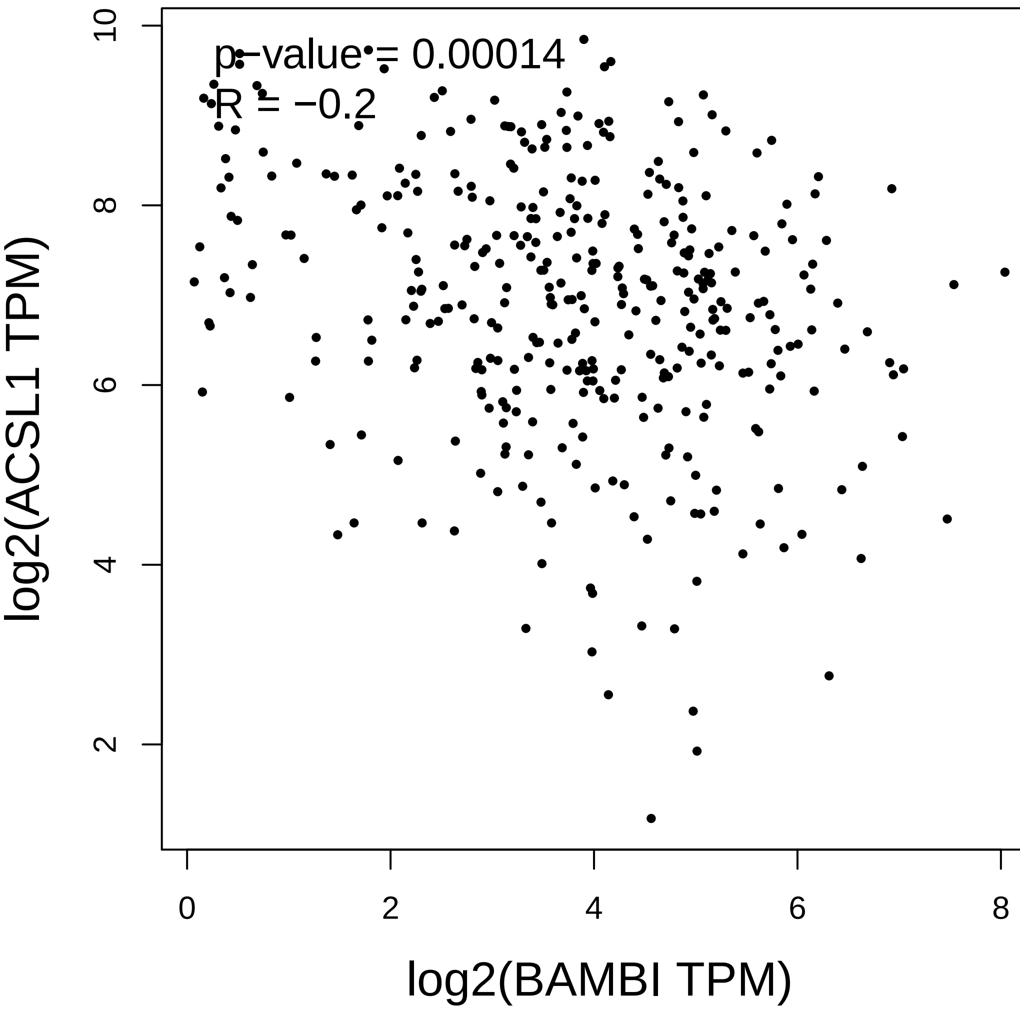

p−value = 0.00014
R = −0.2
log2(ACSL1 TPM)
log2(BAMBI TPM)
2
4
6
8
10
0
2
4
6
8

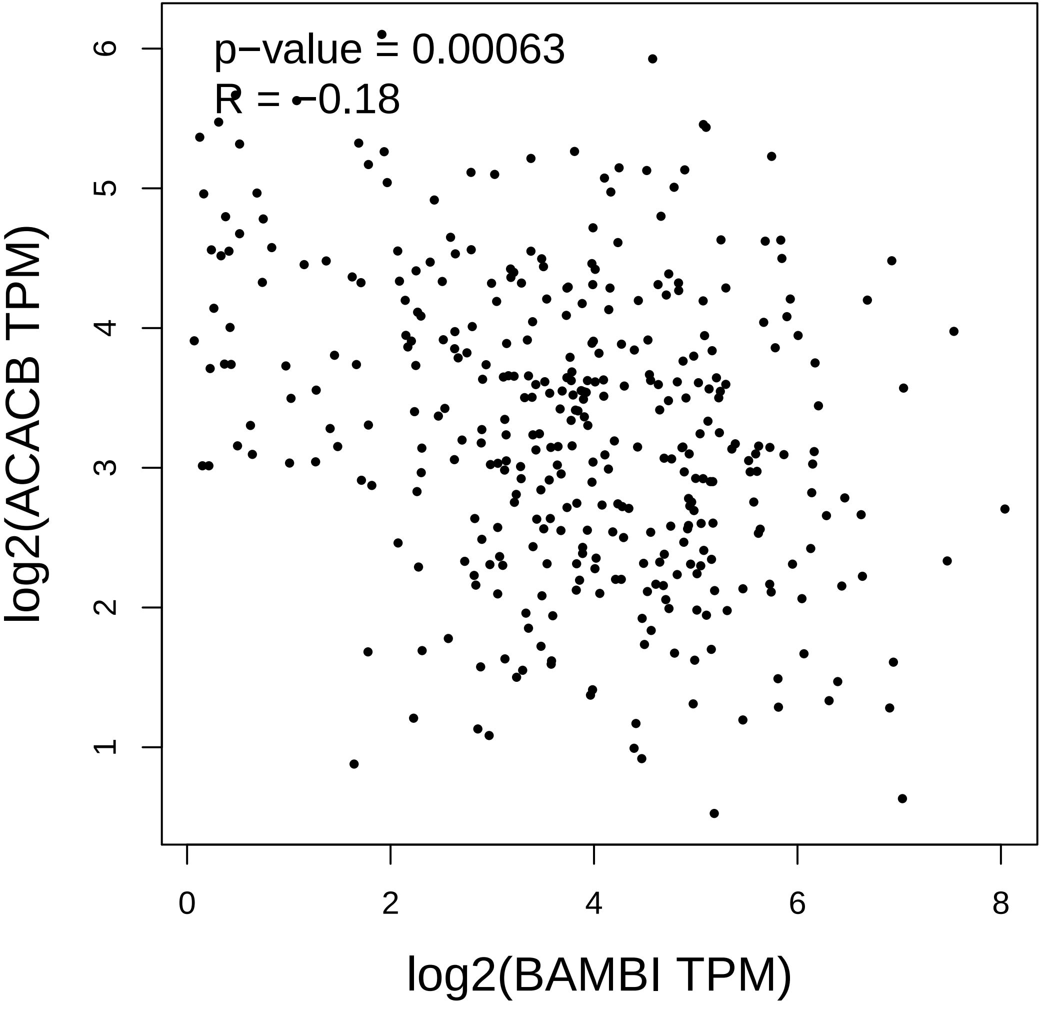
p−value = 0.00063
R = −0.18
log2(ACACB TPM)
1
2
3
4
5
6
log2(BAMBI TPM)
0
2
4
6
8

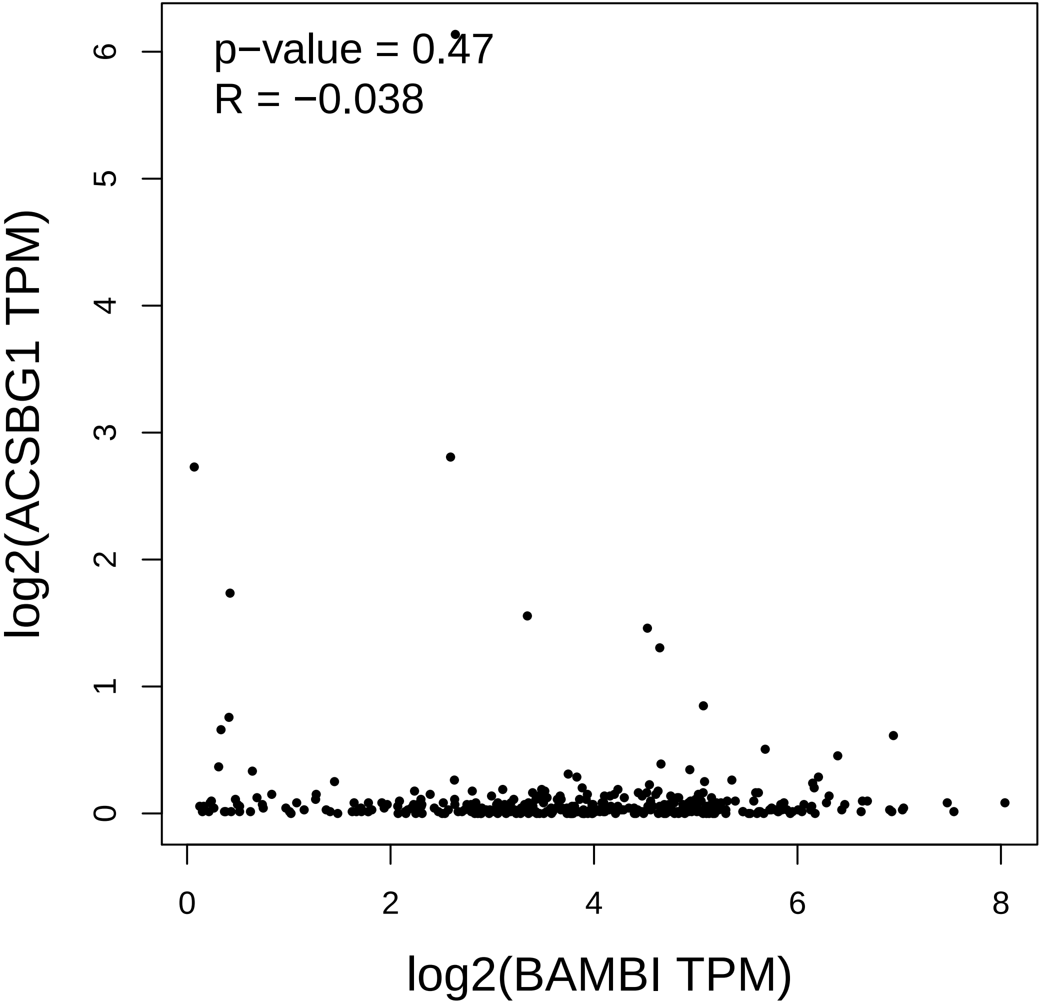
p−value = 0.47
R = −0.038
log2(ACSBG1 TPM)
log2(BAMBI TPM)
0
1
2
3
4
5
6
0
2
4
6
8

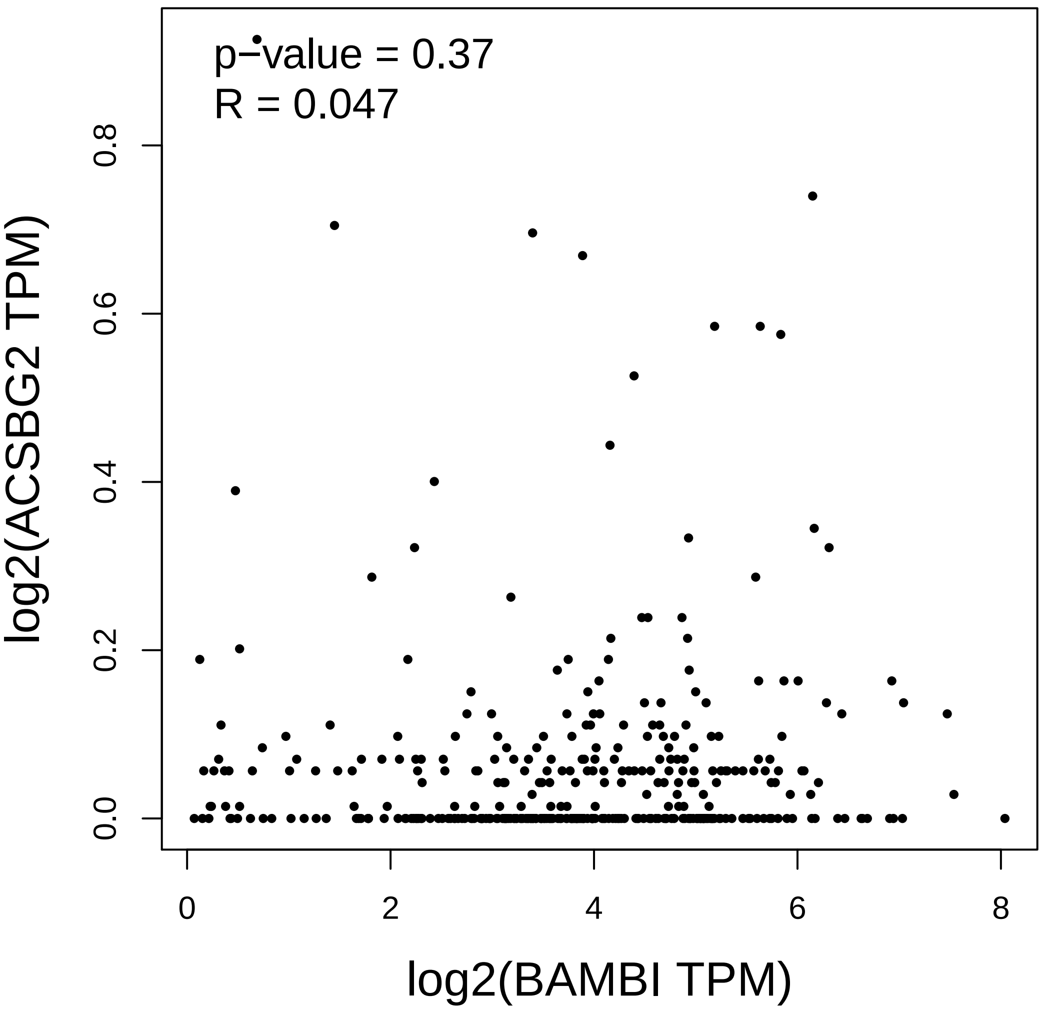
p−value = 0.37
R = 0.047
log2(ACSBG2 TPM)
0.0
0.2
0.4
0.6
0.8
0
2
4
6
8
log2(BAMBI TPM)

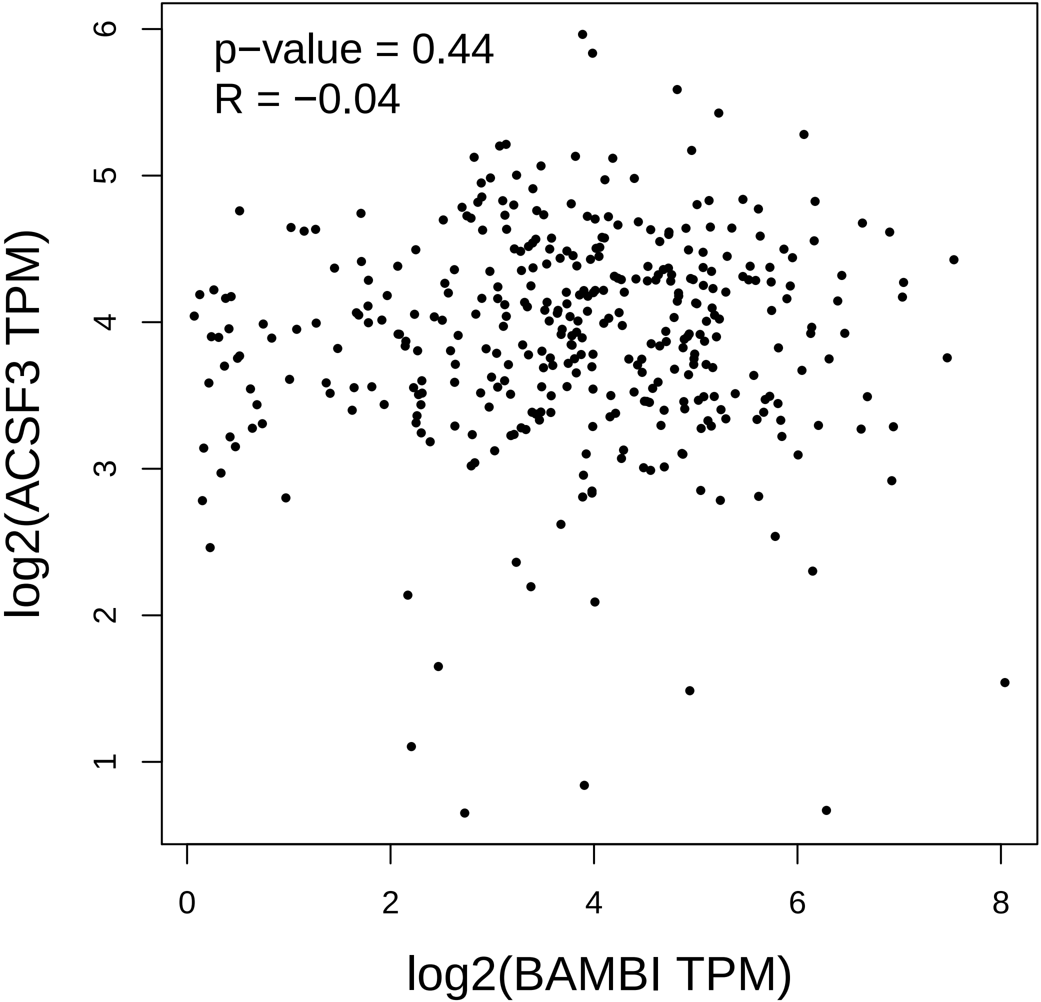

p−value = 0.44
R = −0.04
log2(ACSF3 TPM)
log2(BAMBI TPM)
1
2
3
4
5
6
0
2
4
6
8

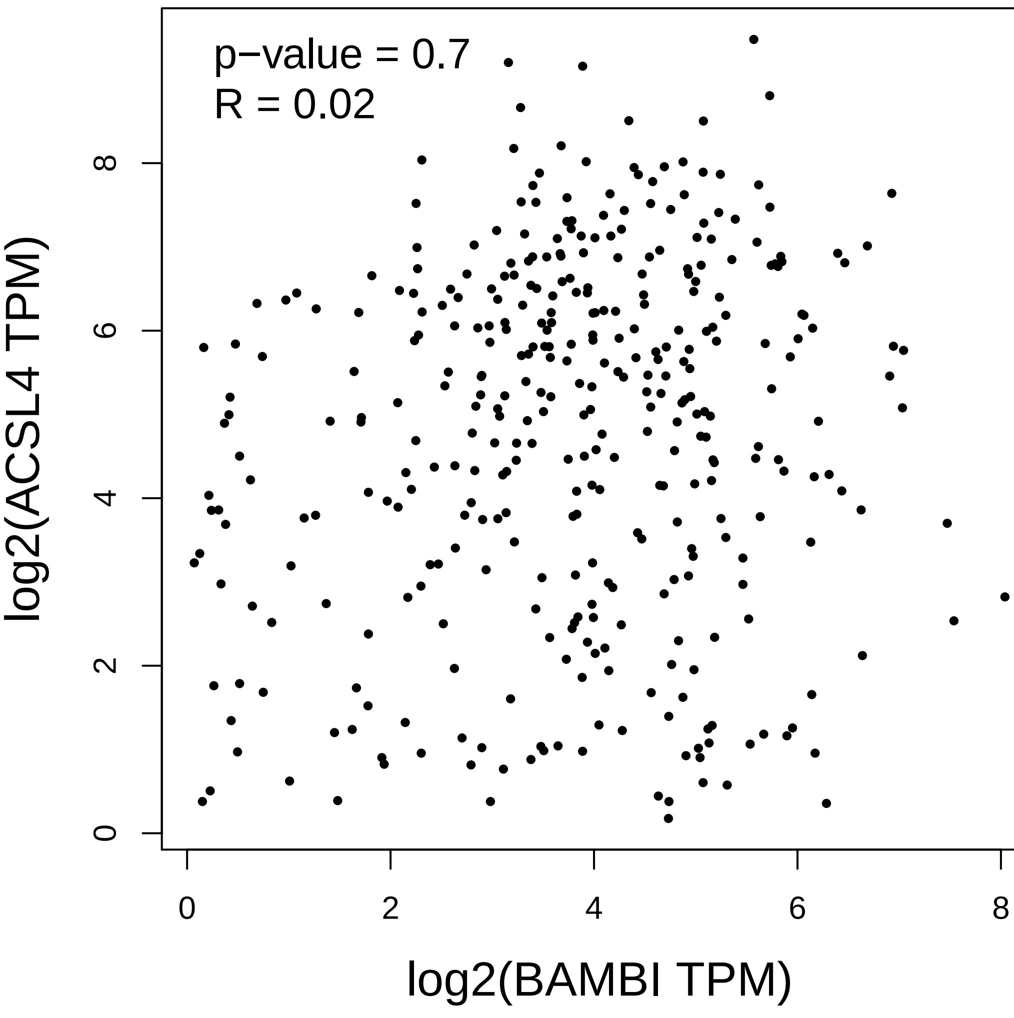

p−value = 0.7
R = 0.02
log2(ACSL4 TPM)
log2(BAMBI TPM)
0
2
4
6
8

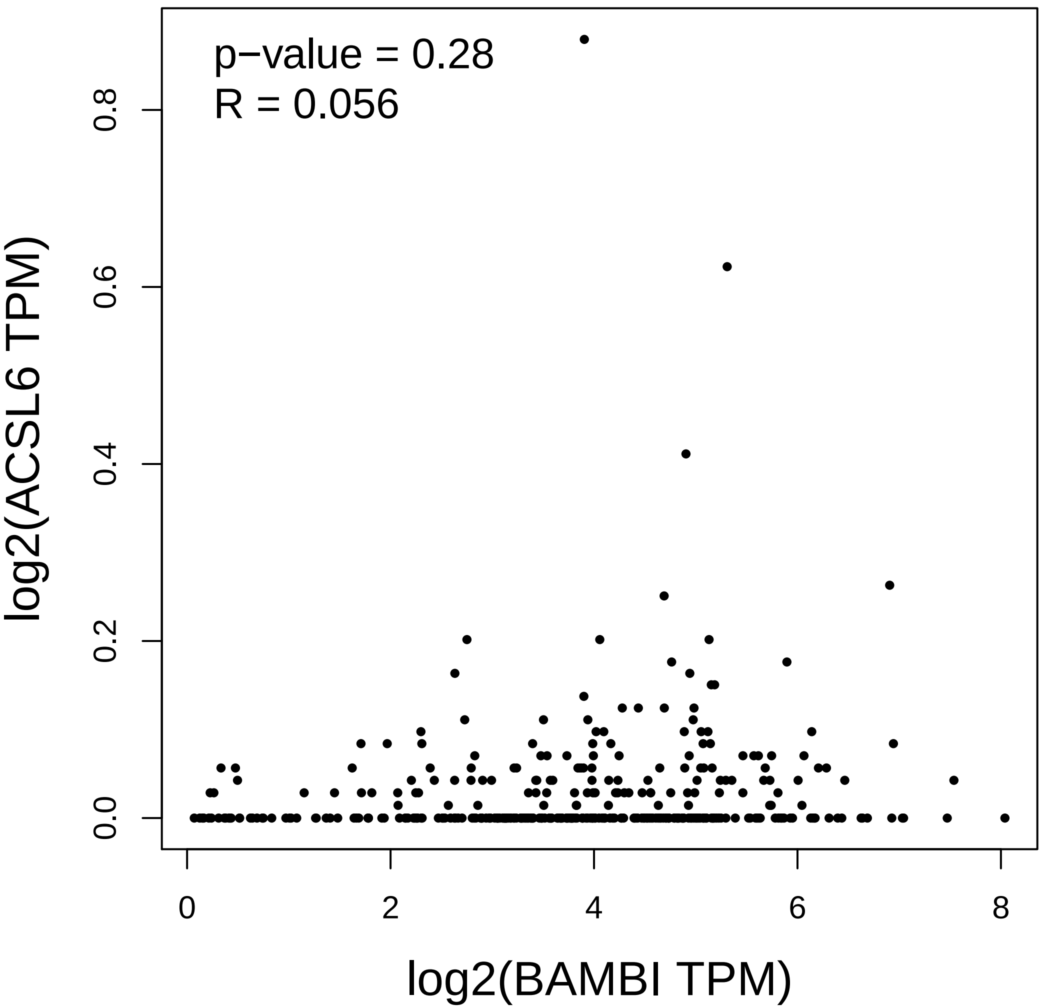
p−value = 0.28
R = 0.056
0.0
0.2
0.4
0.6
0.8
log2(ACSL6 TPM)
0
2
4
6
8
log2(BAMBI TPM)

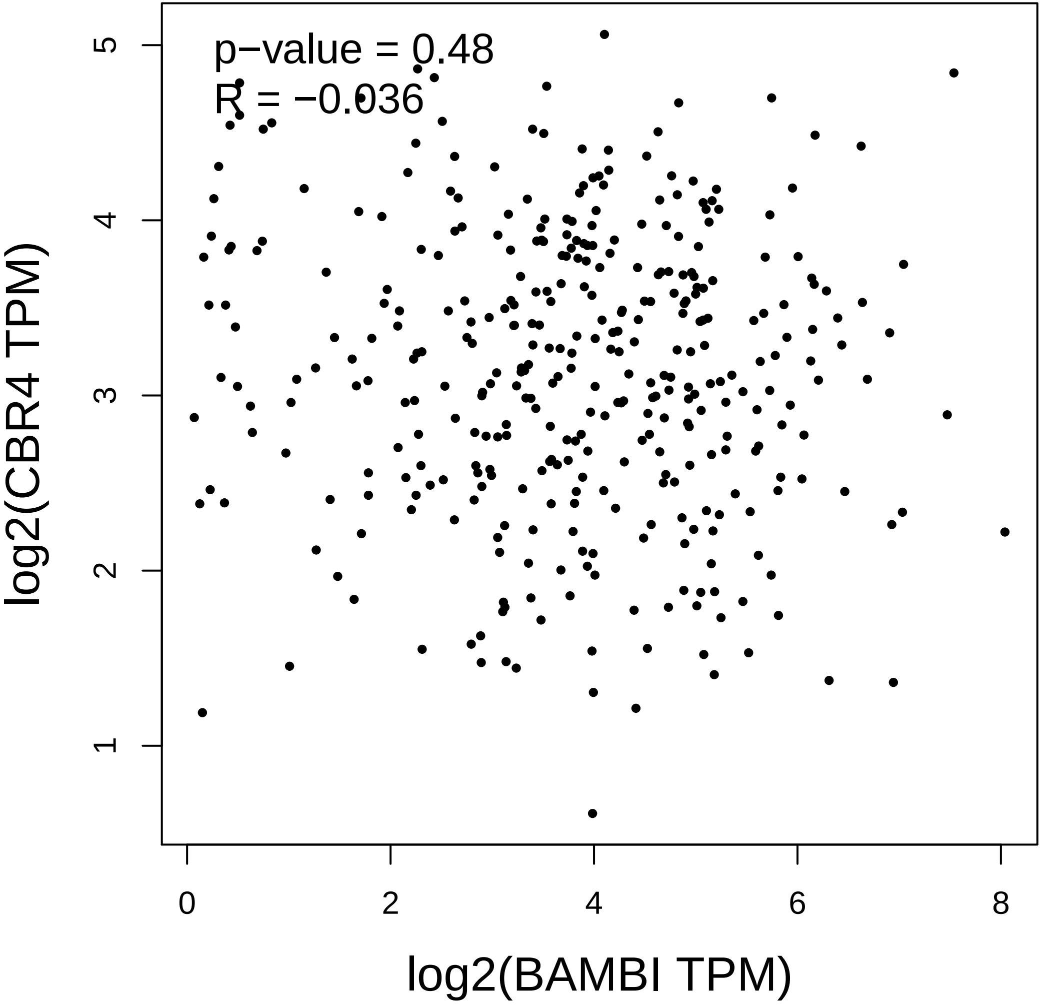
p−value = 0.48
R = −0.036
log2(CBR4 TPM)
log2(BAMBI TPM)
0
2
4
6
8
1
2
3
4
5

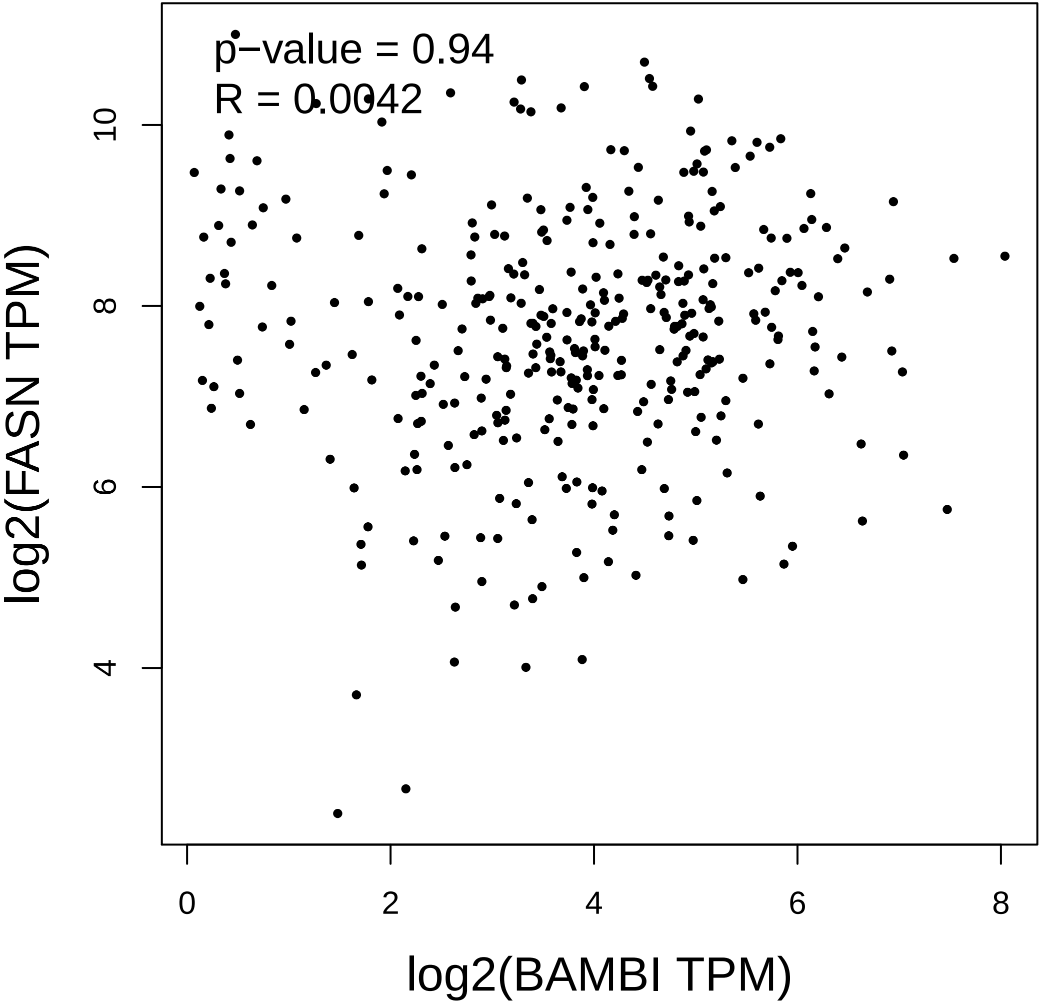
p−value = 0.94
R = 0.0042
log2(FASN TPM)
10
8
6
4
0
2
4
6
8
log2(BAMBI TPM)

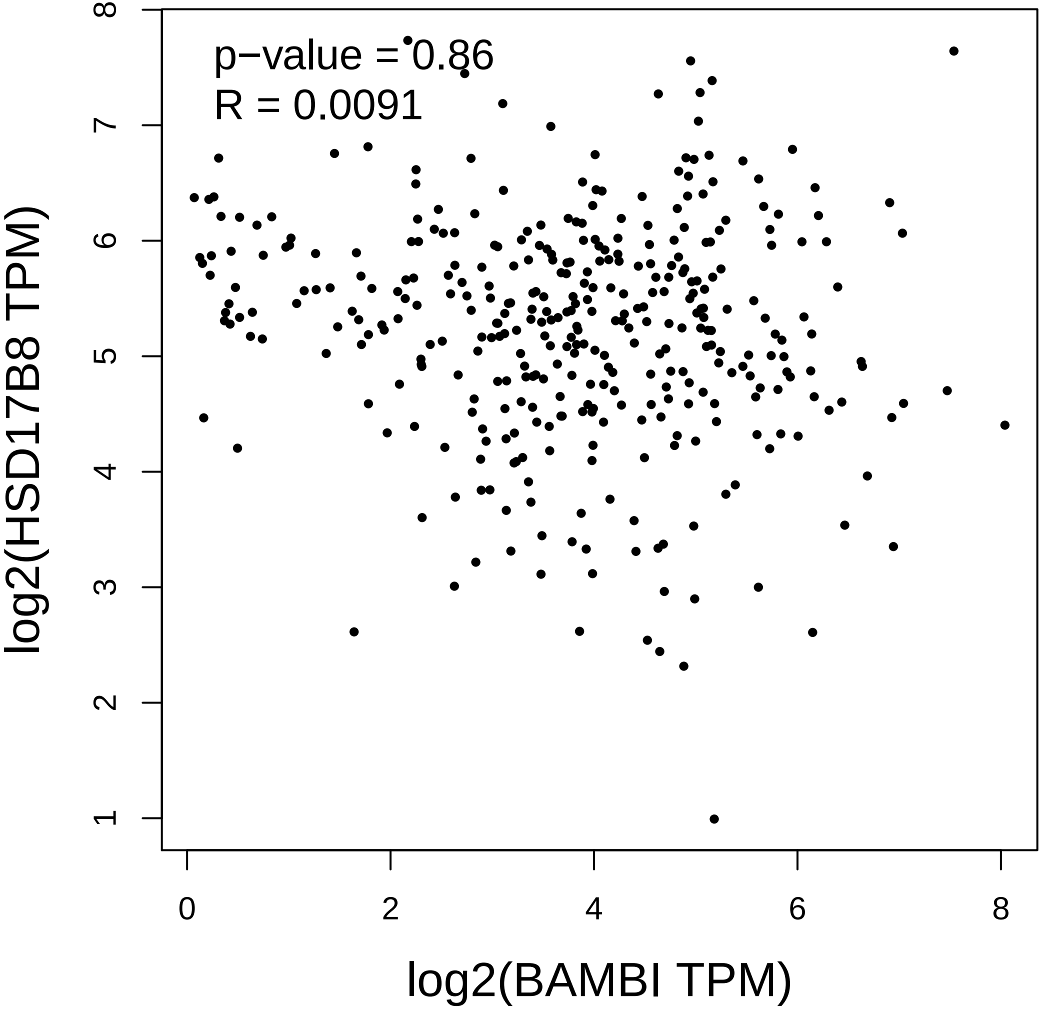
p−value = 0.86
R = 0.0091
log2(HSD17B8 TPM)
log2(BAMBI TPM)
1
2
3
4
5
6
7
8
0
2
4
6
8

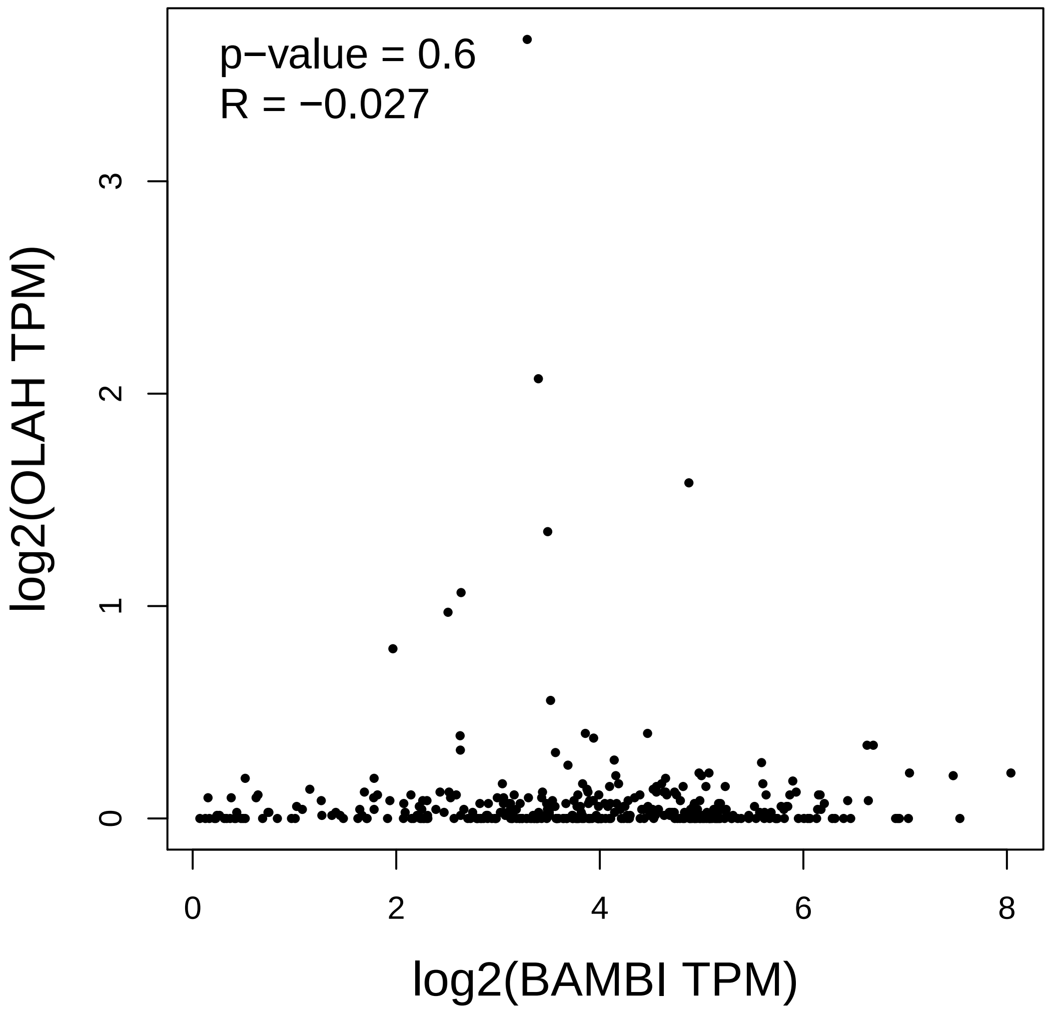
p−value = 0.6
R = −0.027
log2(OLAH TPM)
0
1
2
3
0
2
4
6
8
log2(BAMBI TPM)
