# Supplementary material for: BAMBI Is a Prognostic Biomarker Associated with Macrophage Polarization, Glycolysis, and Lipid Metabolism in Hepatocellular Carcinoma
Source: Int J Mol Sci. 2024 Nov 26;25(23):12713. doi: 10.3390/ijms252312713 (PMC11640931; doi:10.3390/ijms252312713)
Supplement: Supplementary file 1 [file ijms-25-12713-s001.zip › Correlation of BAMBI and 43 genes in the fatty acid degradation pathway.pdf]

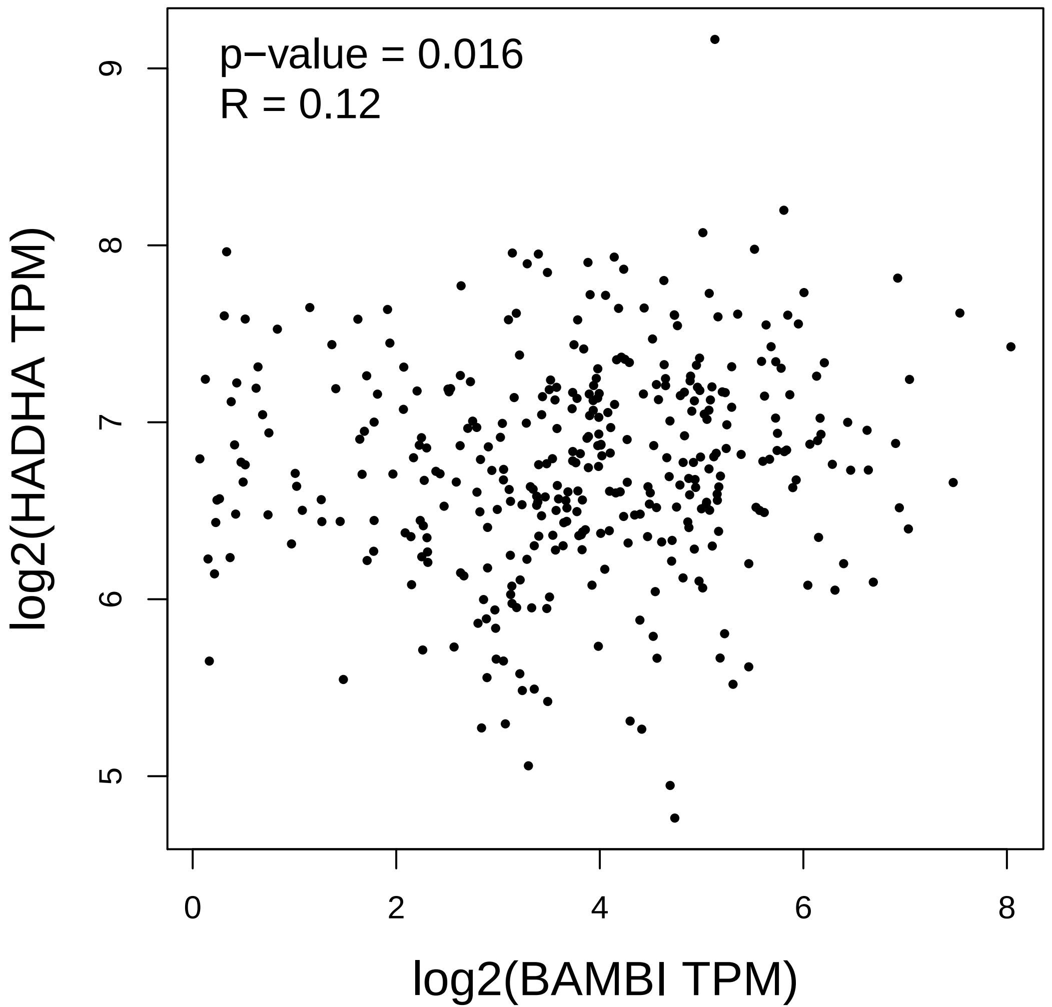

p−value = 0.016
R = 0.12
log2(HADHA TPM)
log2(BAMBI TPM)
5
6
7
8
9
0
2
4
6
8

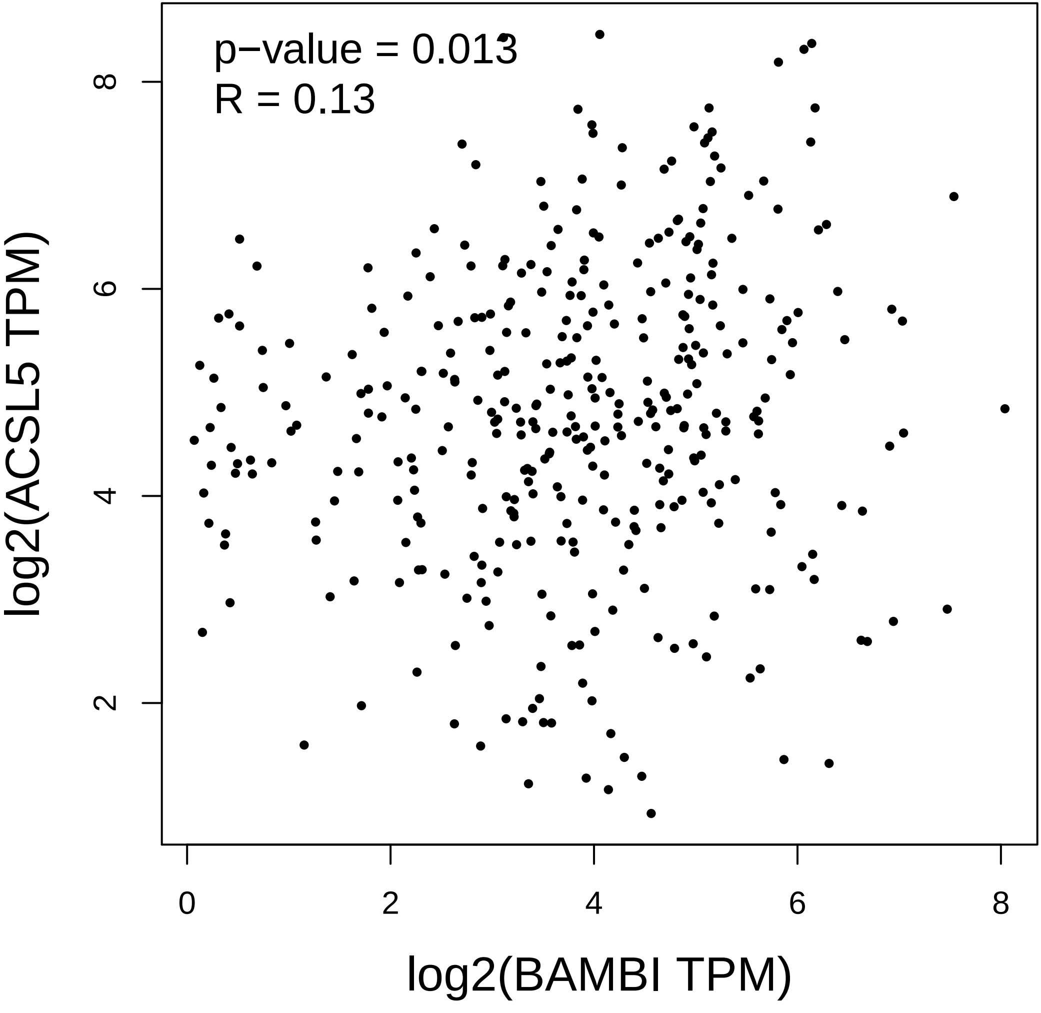

p−value = 0.013
R = 0.13
log2(ACSL5 TPM)
log2(BAMBI TPM)
0
2
4
6
8
2
4
6
8

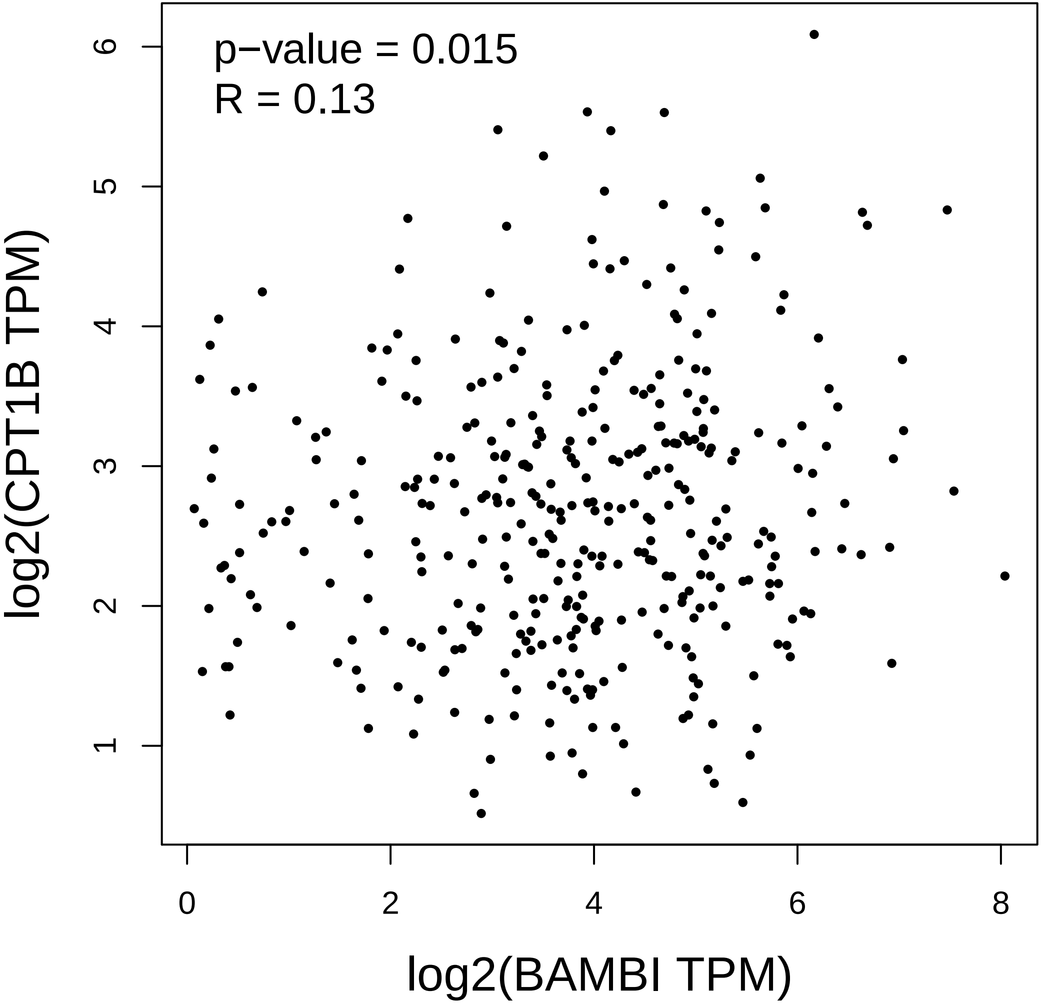
p−value = 0.015
R = 0.13
log2(CPT1B TPM)
log2(BAMBI TPM)
1
2
3
4
5
6
0
2
4
6
8

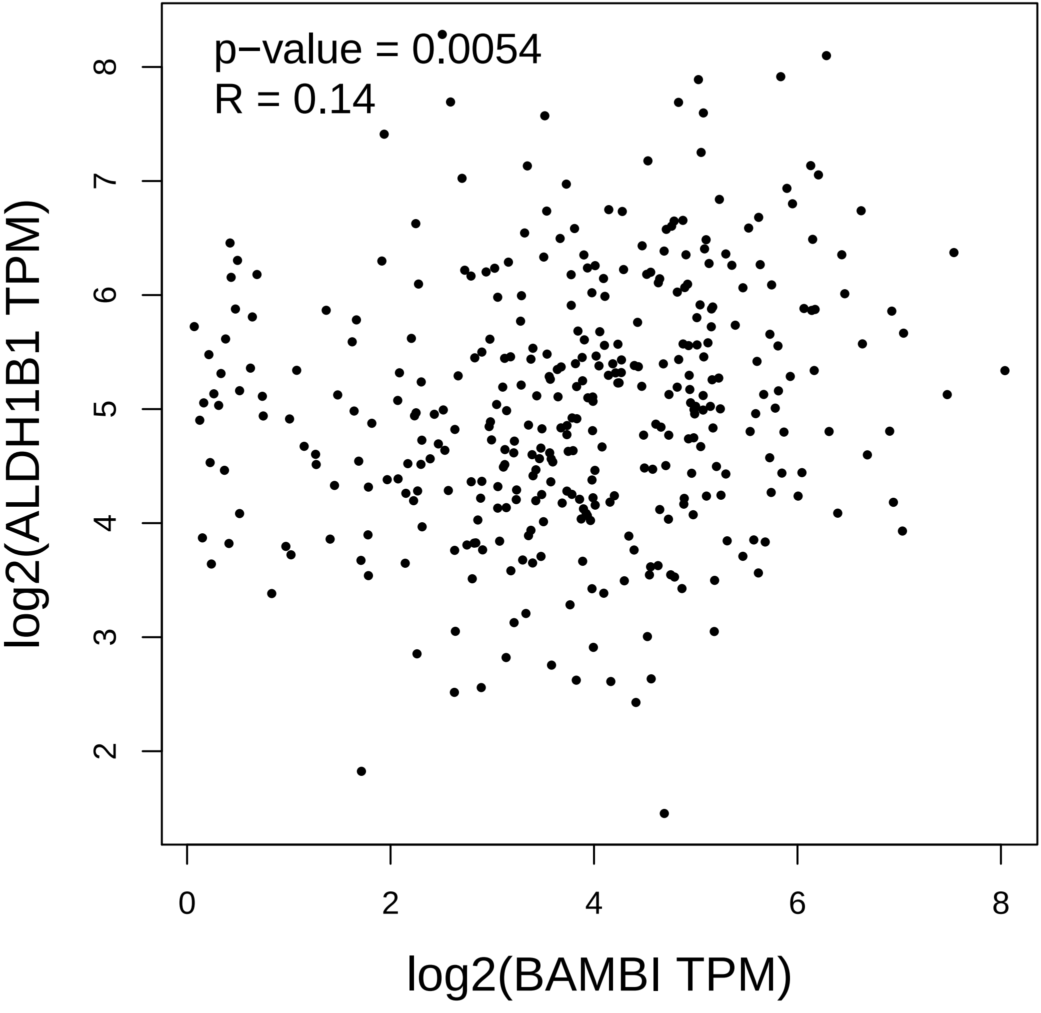
p−value = 0.0054
R = 0.14
log2(ALDH1B1 TPM)
log2(BAMBI TPM)
2
3
4
5
6
7
8
0
2
4
6
8

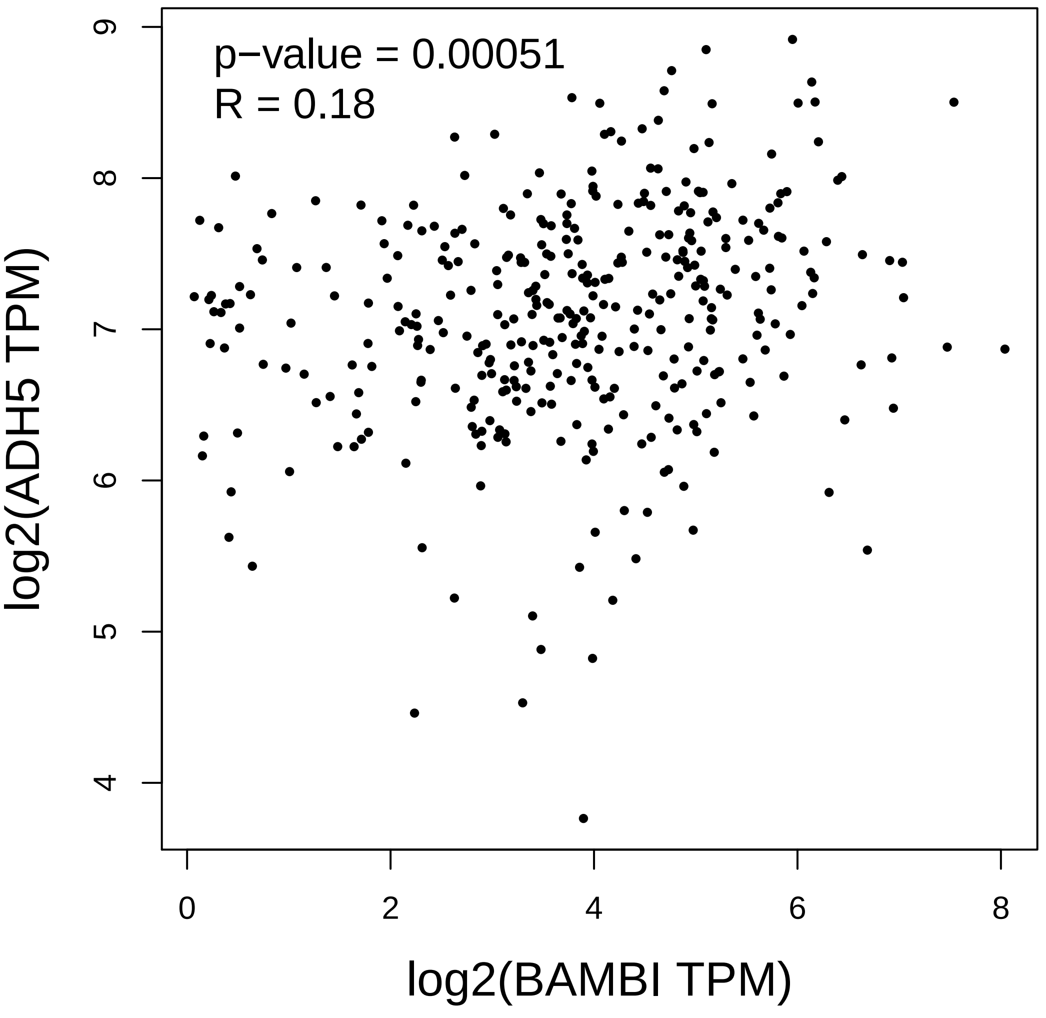
p−value = 0.00051
R = 0.18
log2(ADH5 TPM)
log2(BAMBI TPM)
4
5
6
7
8
9
0
2
4
6
8

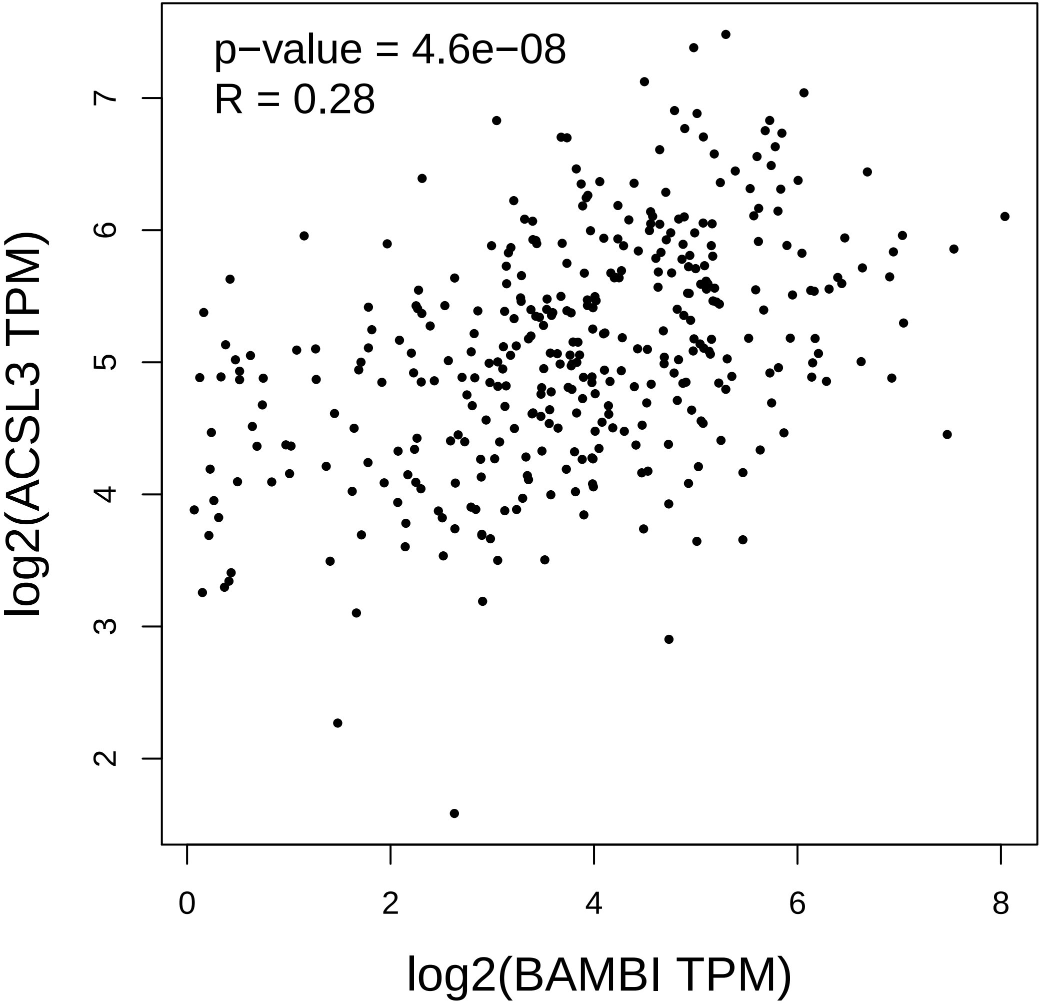

p−value = 4.6e−08
R = 0.28
log2(ACSL3 TPM)
2
3
4
5
6
7
log2(BAMBI TPM)
0
2
4
6
8

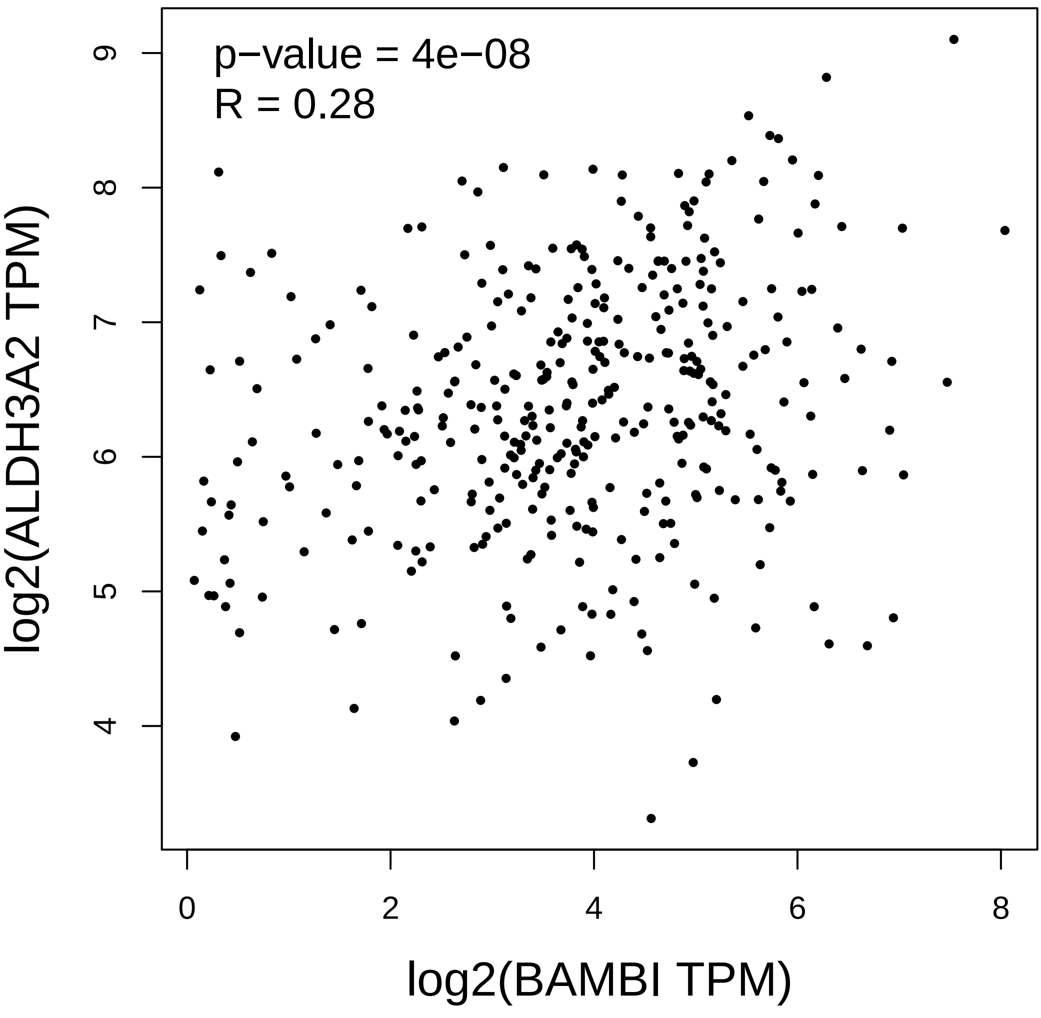
p−value = 4e−08
R = 0.28
log2(ALDH3A2 TPM)
log2(BAMBI TPM)
0
2
4
6
8
4
5
6
7
8
9

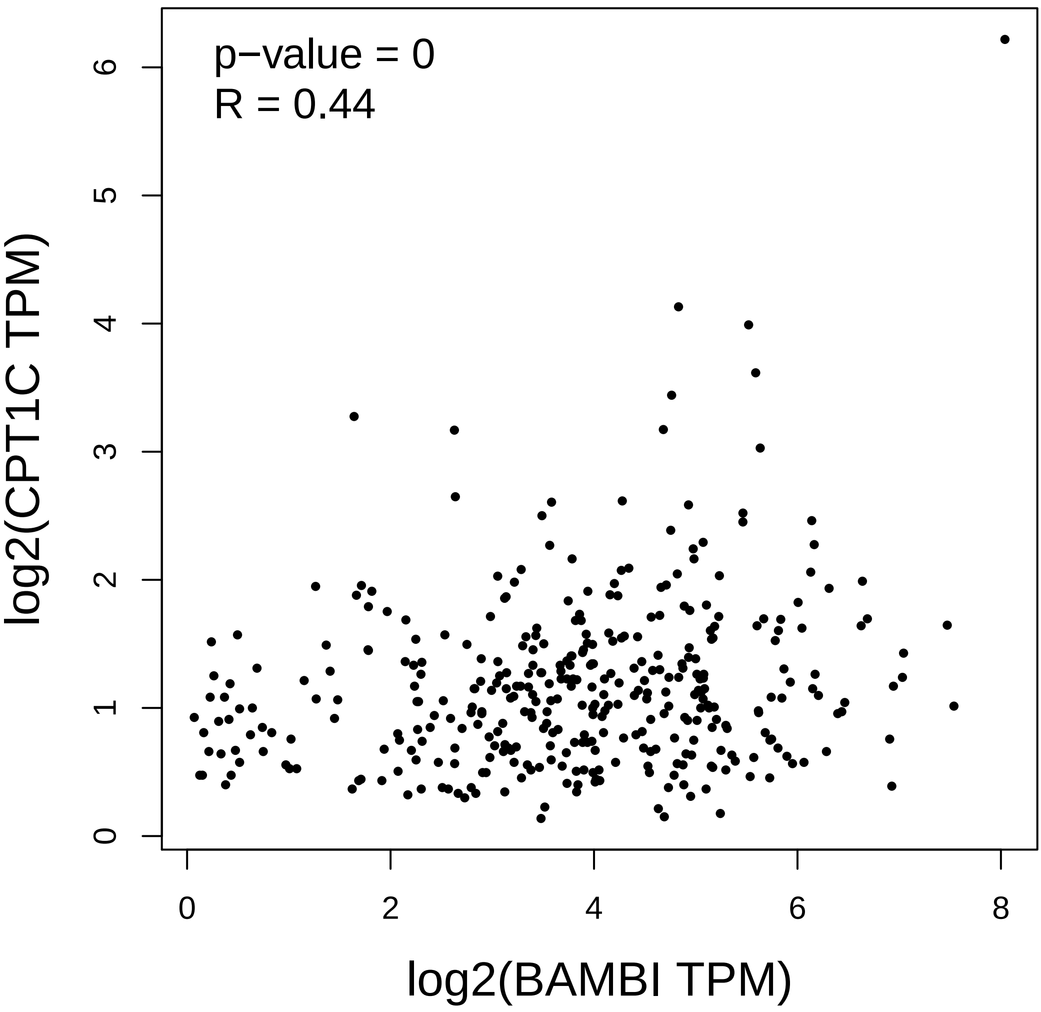

p−value = 0
R = 0.44
log2(CPT1C TPM)
log2(BAMBI TPM)
0
1
2
3
4
5
6
0
2
4
6
8

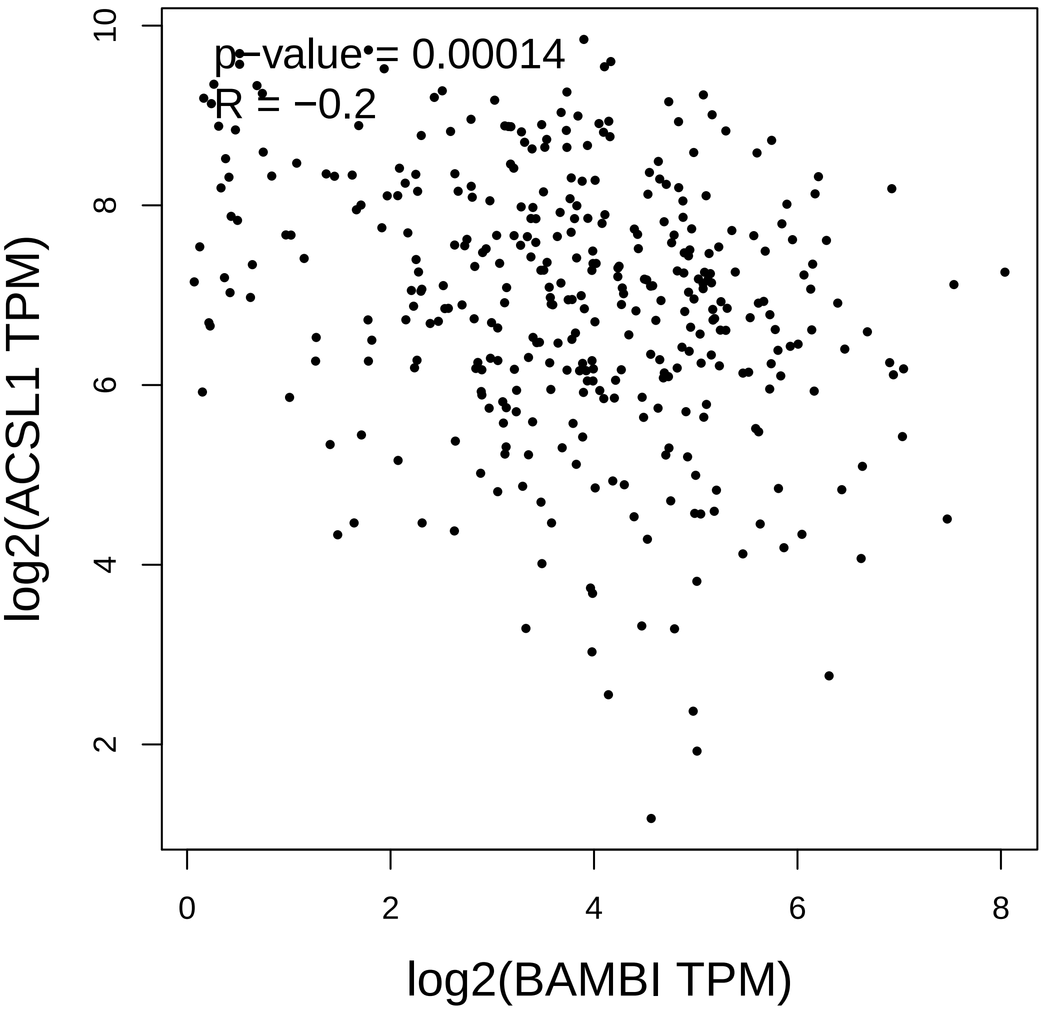
p−value = 0.00014
R = −0.2
log2(ACSL1 TPM)
log2(BAMBI TPM)
0
2
4
6
8
10

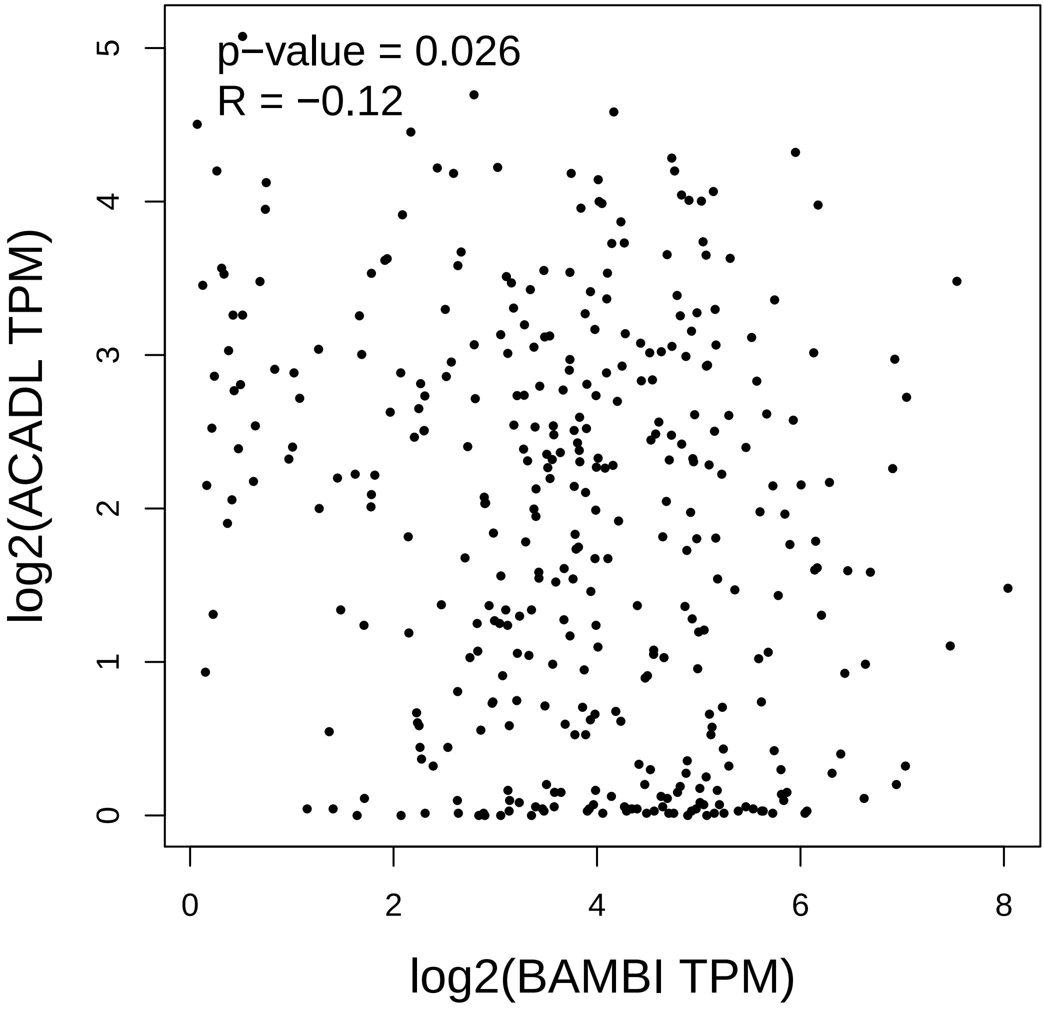
p−value = 0.026
R = −0.12
log2(ACADL TPM)
log2(BAMBI TPM)
0
1
2
3
4
5
0
2
4
6
8

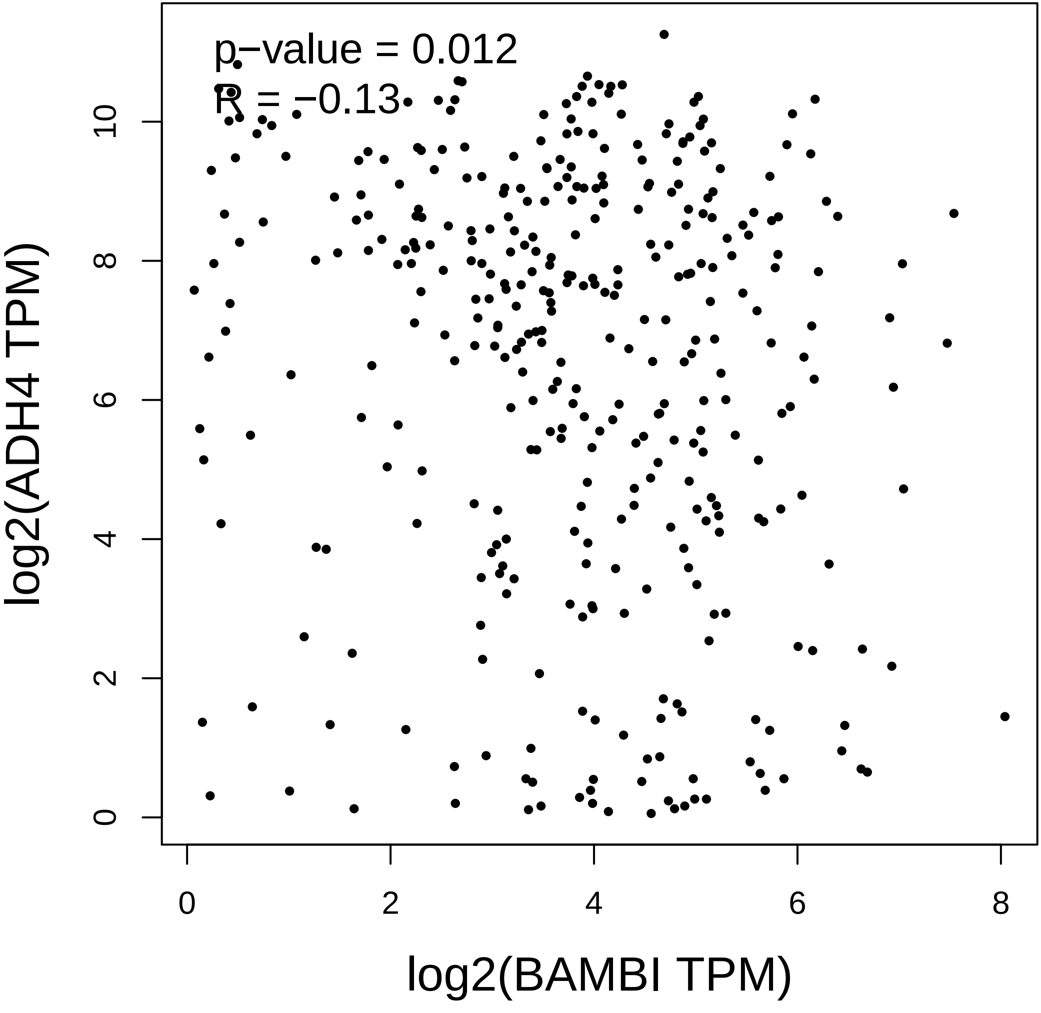

p−value = 0.012
R = −0.13
log2(ADH4 TPM)
0
2
4
6
8
10
0
2
4
6
8
log2(BAMBI TPM)

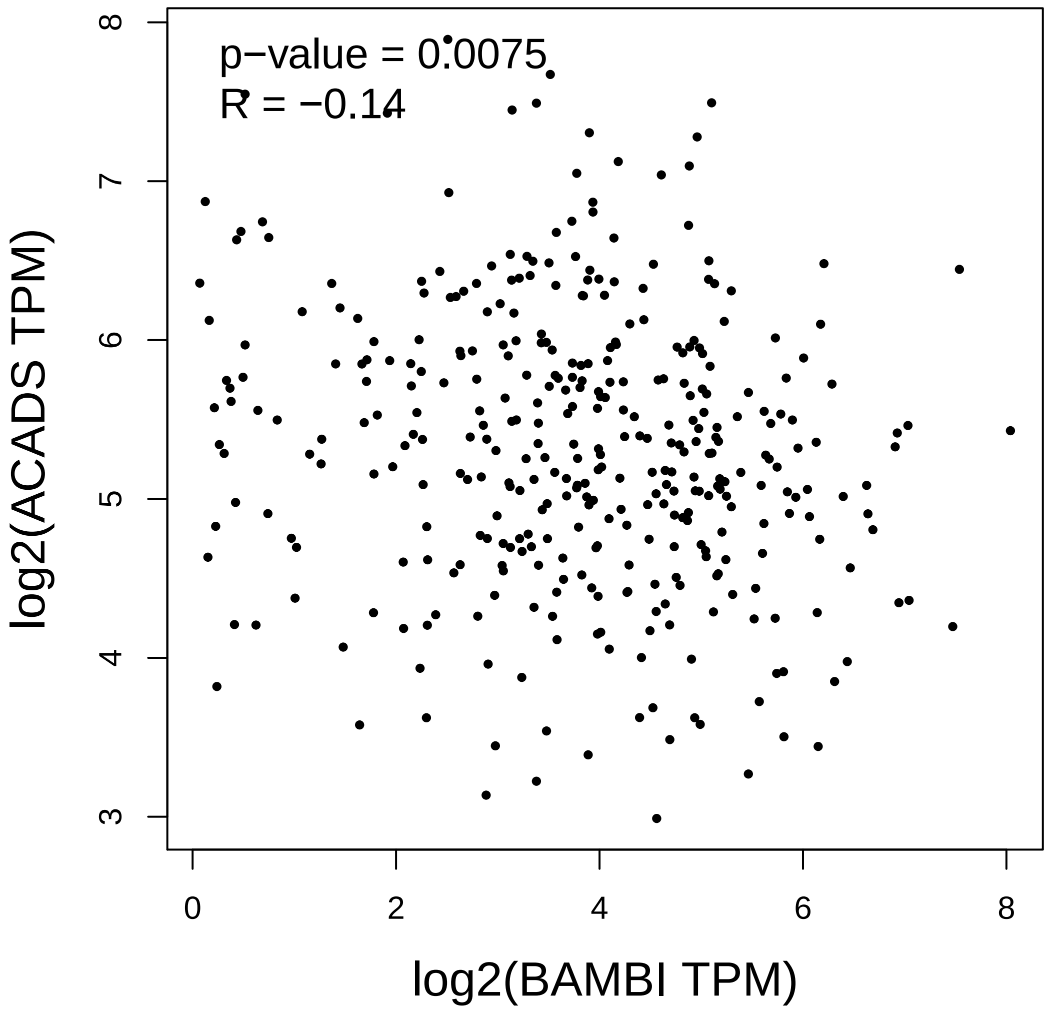
p−value = 0.0075
R = −0.14
log2(ACADS TPM)
log2(BAMBI TPM)
3
4
5
6
7
8
0
2
4
6
8

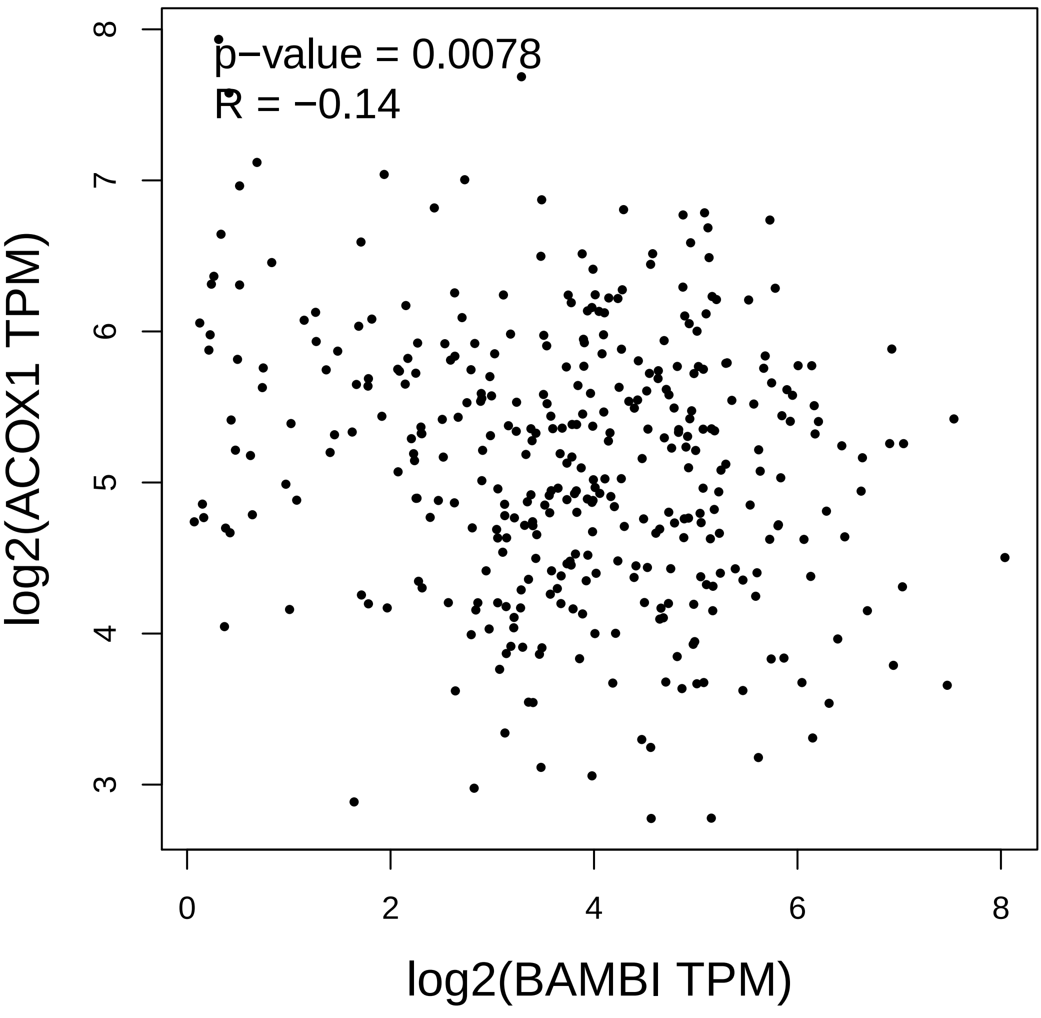
p−value = 0.0078
R = −0.14
log2(ACOX1 TPM)
log2(BAMBI TPM)
3
4
5
6
7
8
0
2
4
6
8

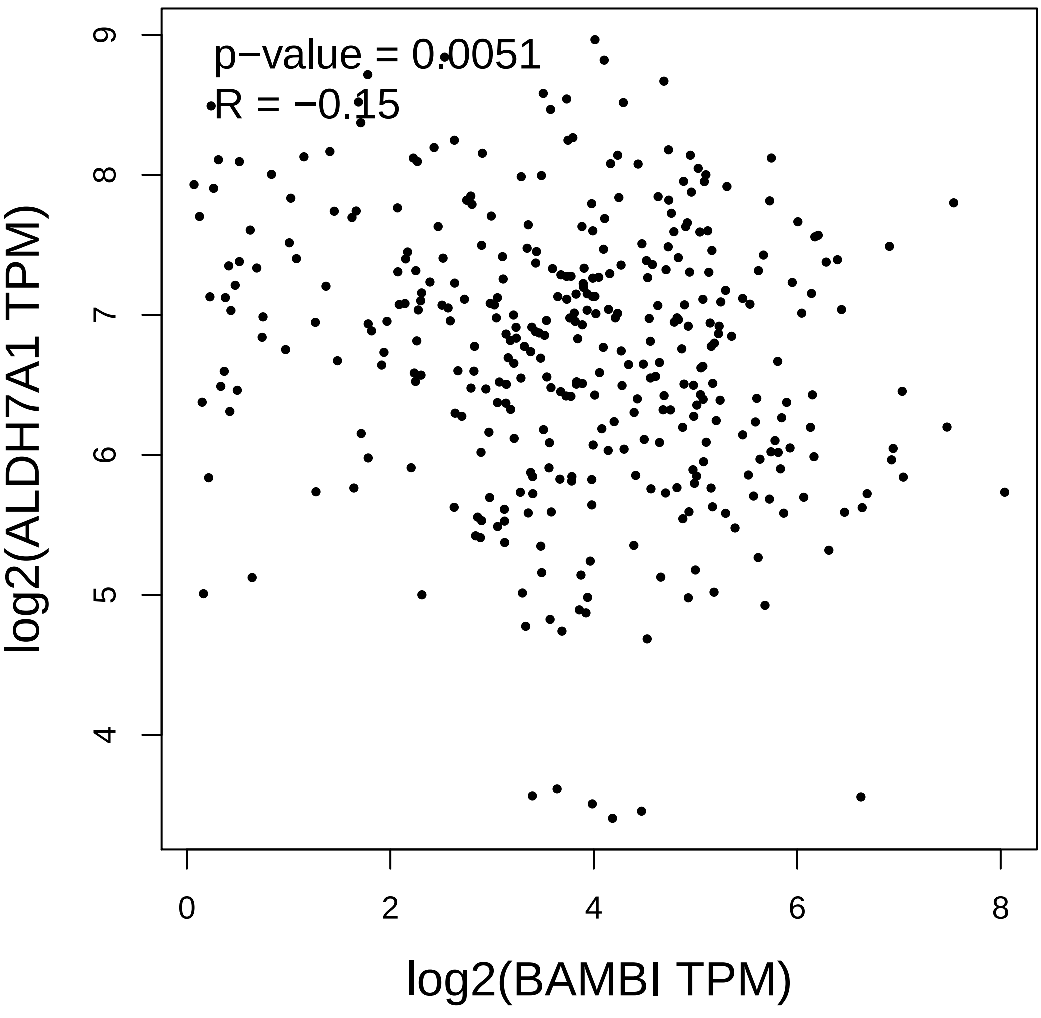
p−value = 0.0051
R = −0.15
log2(ALDH7A1 TPM)
9
8
7
6
5
4
0
2
4
6
8
log2(BAMBI TPM)

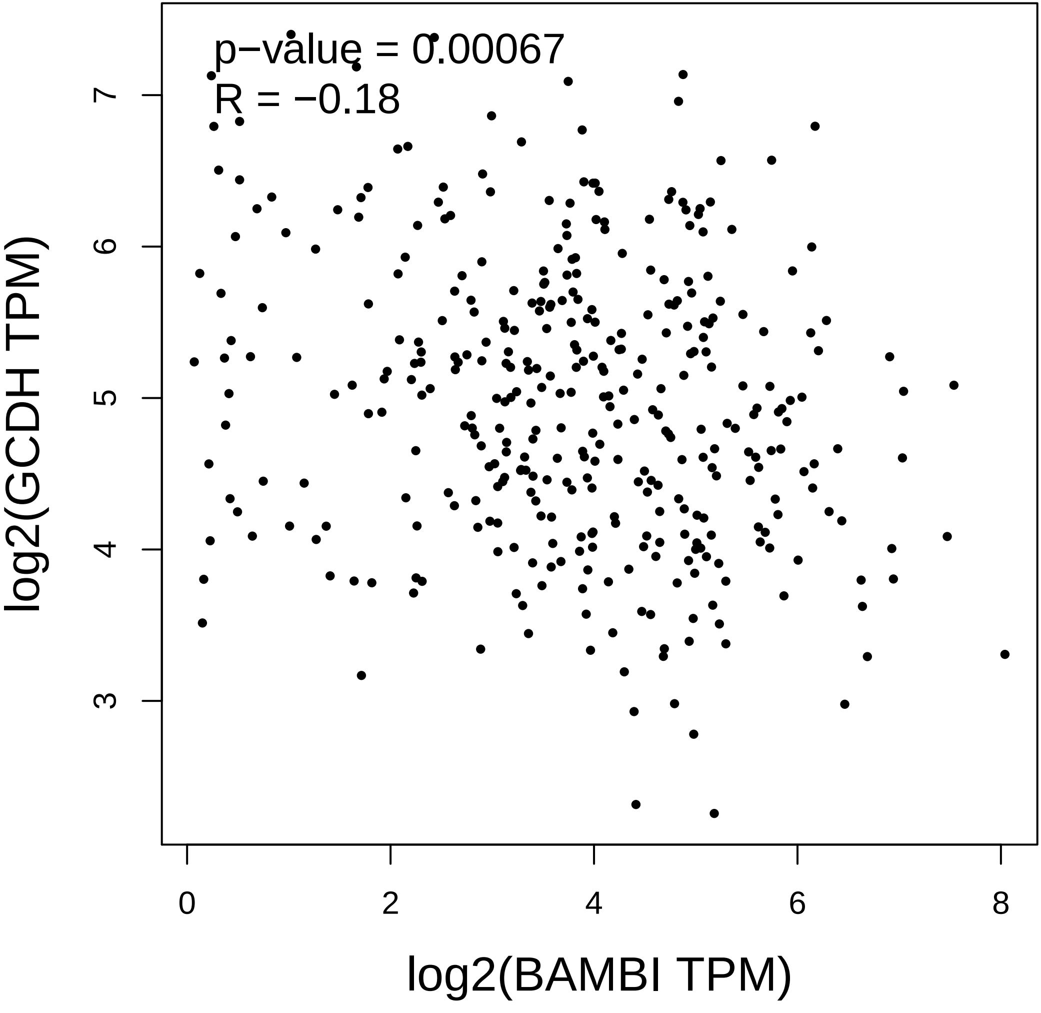

p−value = 0.00067
R = −0.18
log2(GCDH TPM)
3
4
5
6
7
0
2
4
6
8
log2(BAMBI TPM)

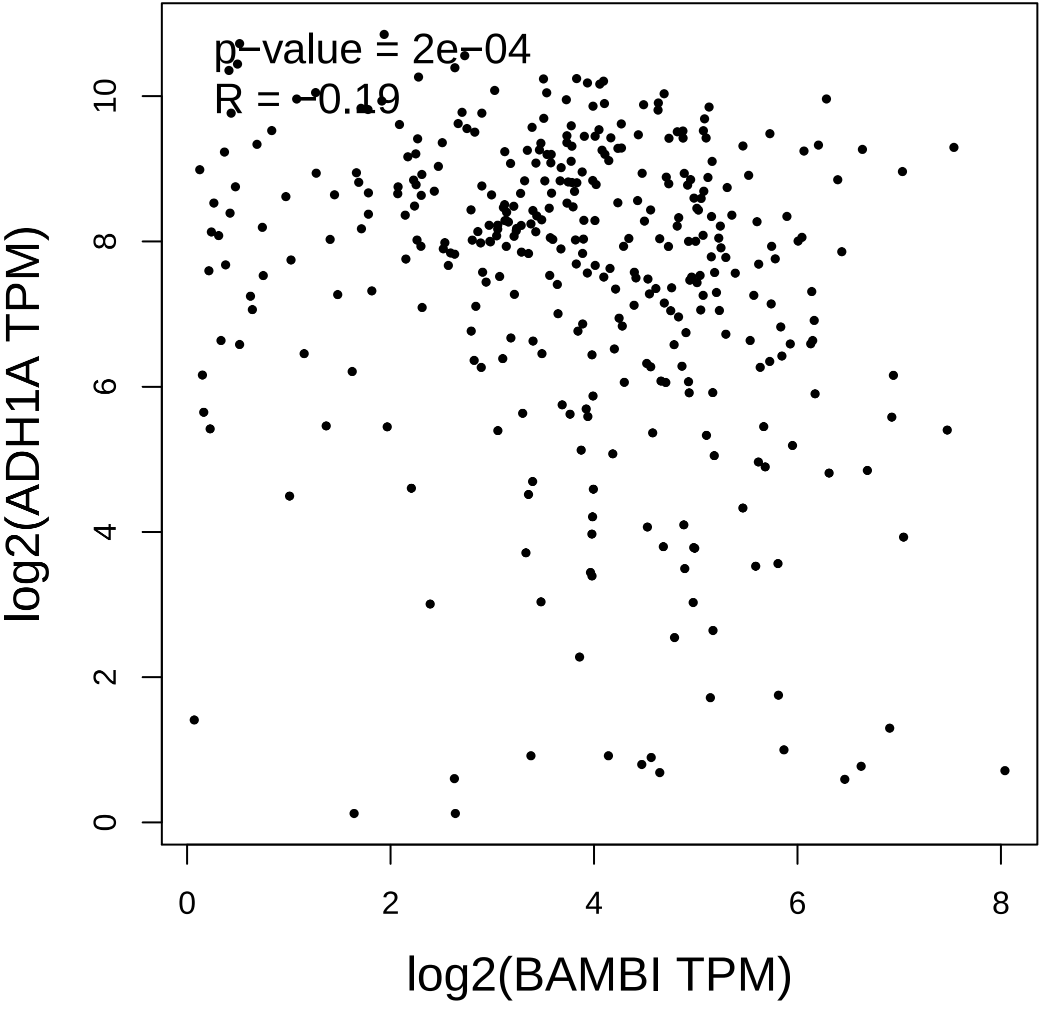
p−value = 2e−04
R = −0.19
log2(ADH1A TPM)
log2(BAMBI TPM)
0
2
4
6
8
10

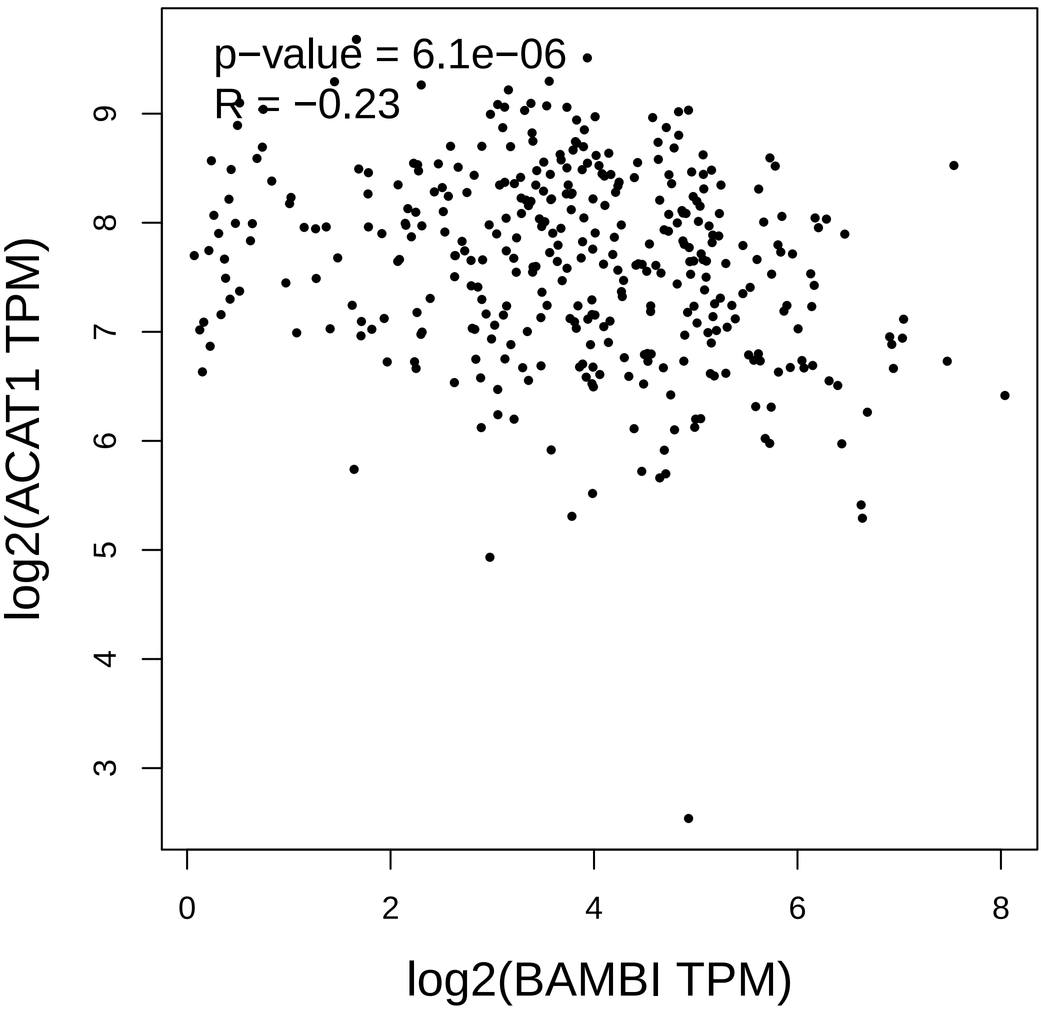

p−value = 6.1e−06
R = −0.23
log2(ACAT1 TPM)
3
4
5
6
7
8
9
log2(BAMBI TPM)
0
2
4
6
8

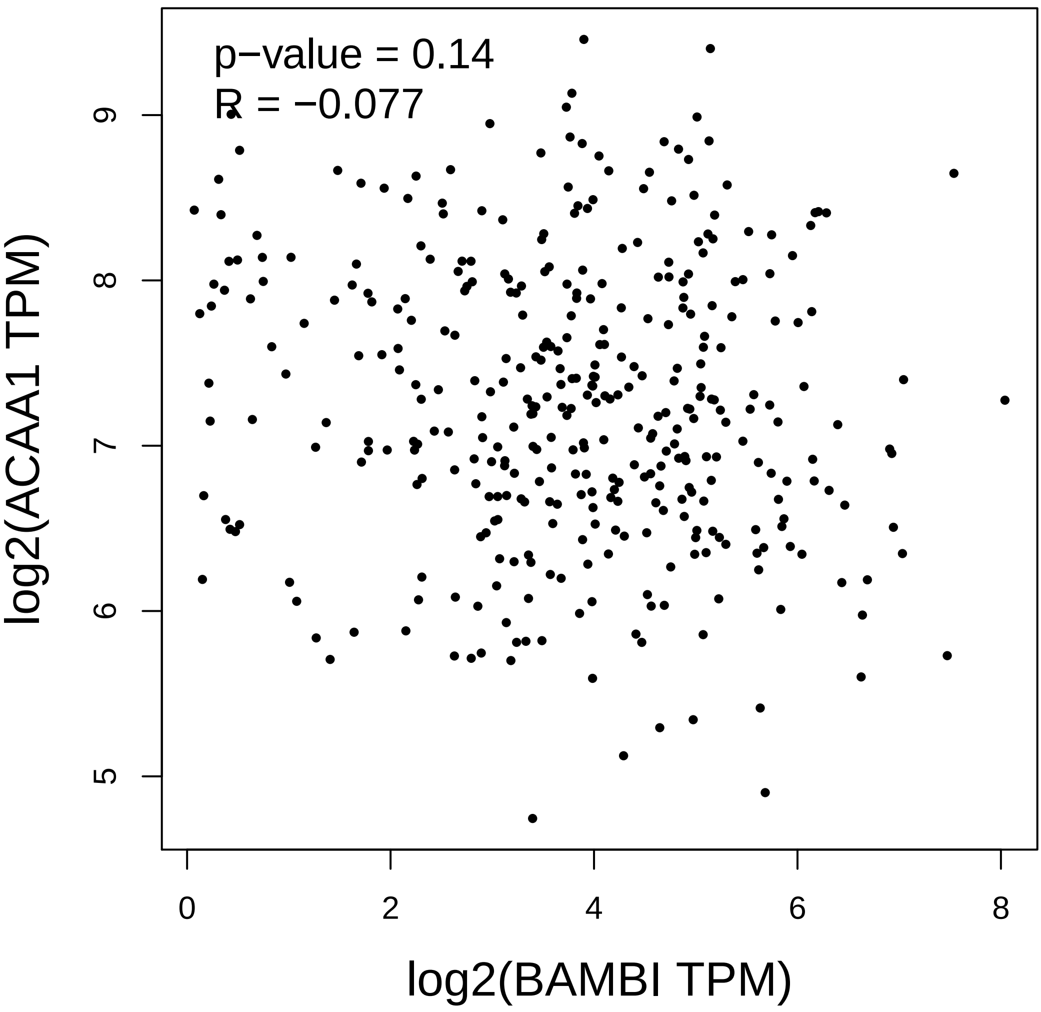

p−value = 0.14
R = −0.077
log2(ACAA1 TPM)
log2(BAMBI TPM)
5
6
7
8
9
0
2
4
6
8

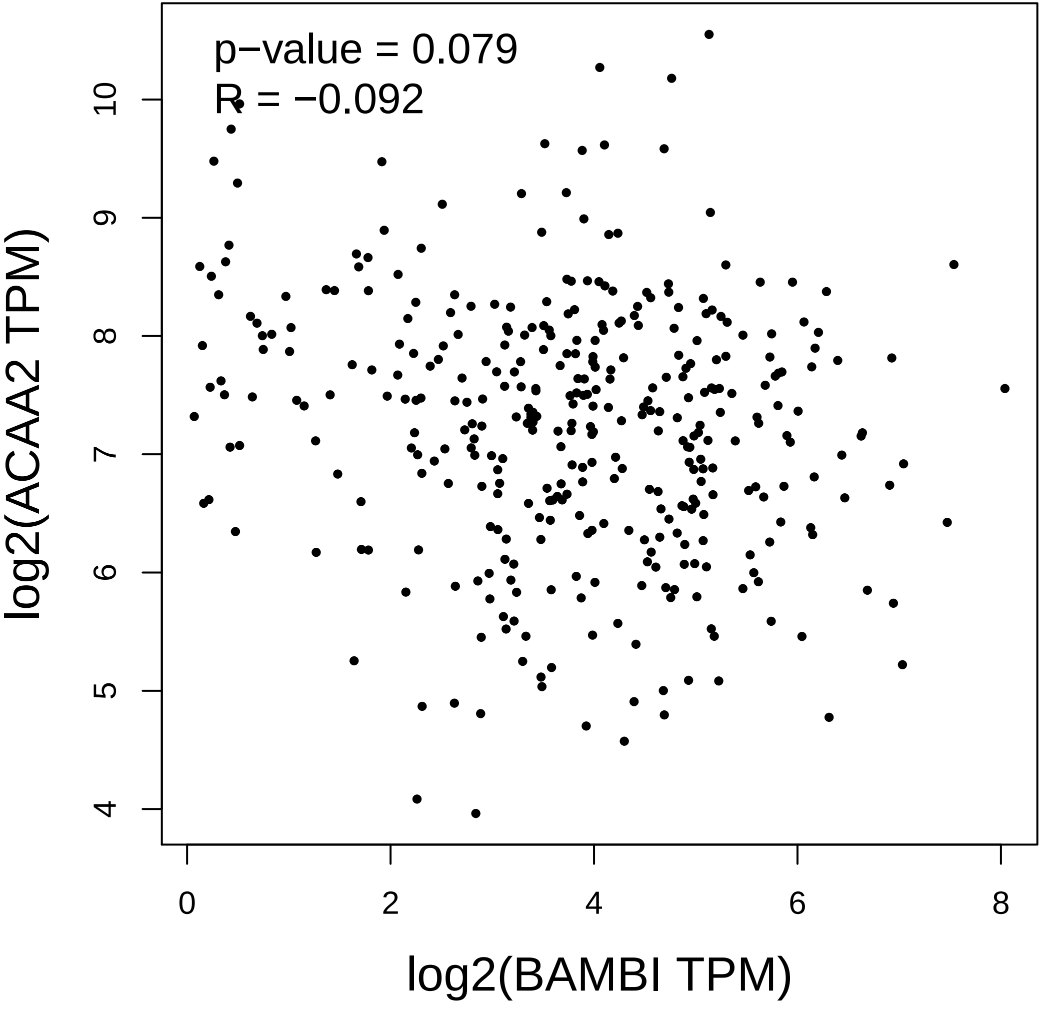

p−value = 0.079
R = −0.092
log2(ACAA2 TPM)
log2(BAMBI TPM)
4
5
6
7
8
9
10
0
2
4
6
8

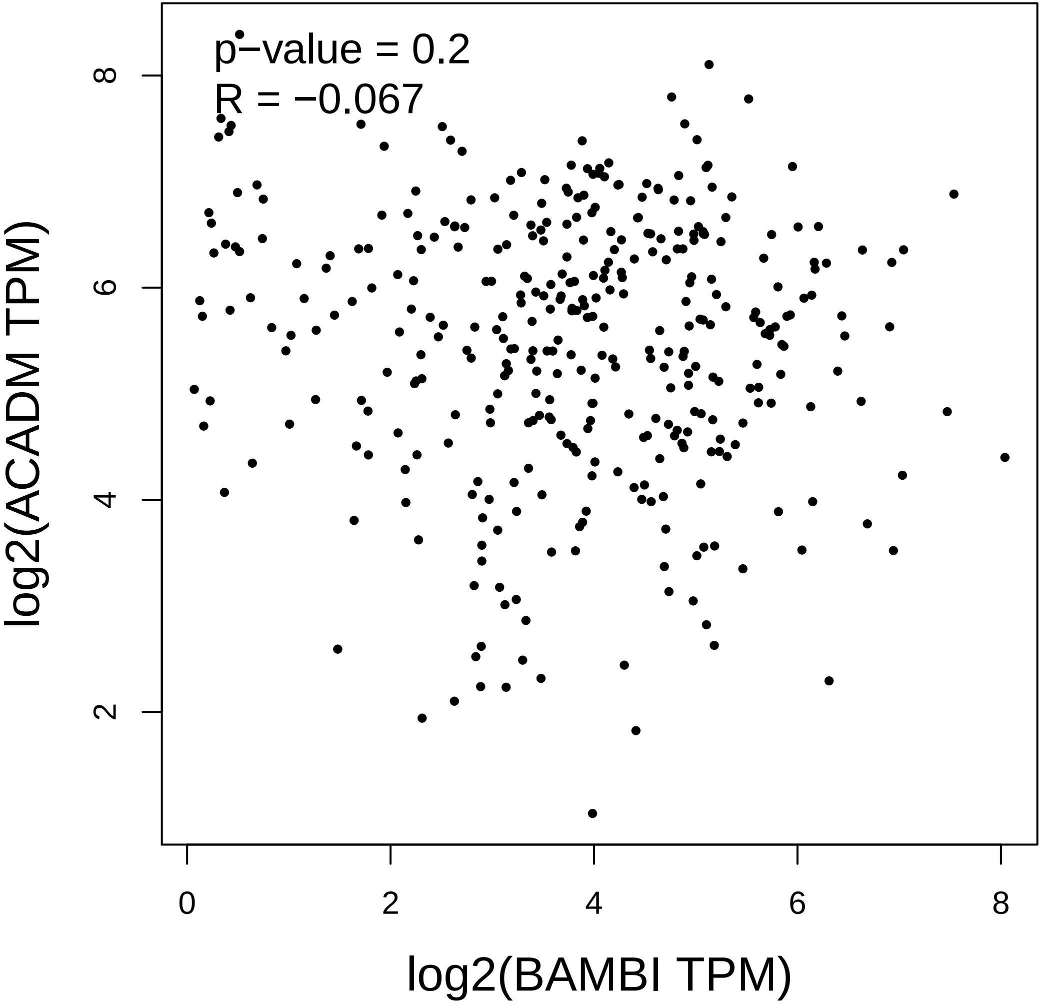

p−value = 0.2
R = −0.067
log2(ACADM TPM)
log2(BAMBI TPM)
0
2
4
6
8
2
4
6
8

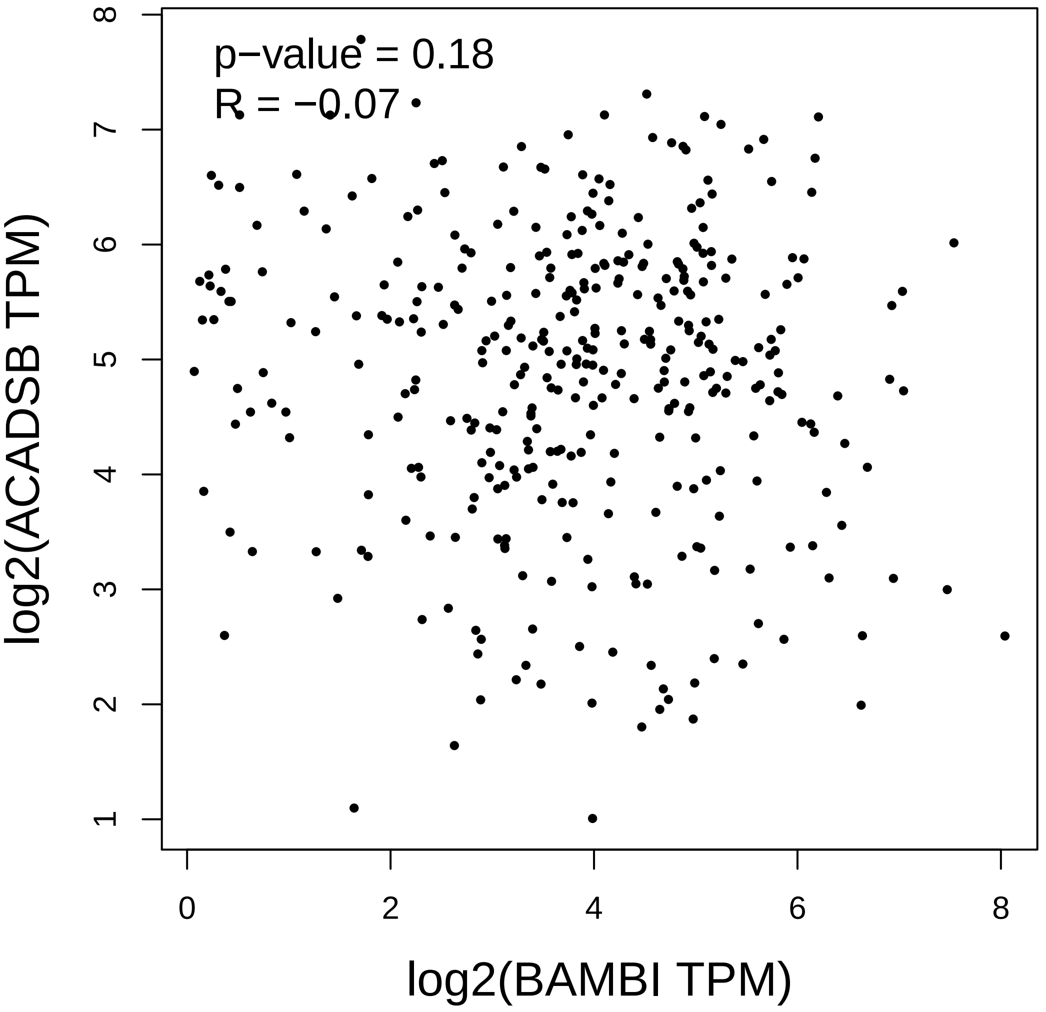

p−value = 0.18
R = −0.07
log2(ACADSB TPM)
log2(BAMBI TPM)
1
2
3
4
5
6
7
8
0
2
4
6
8

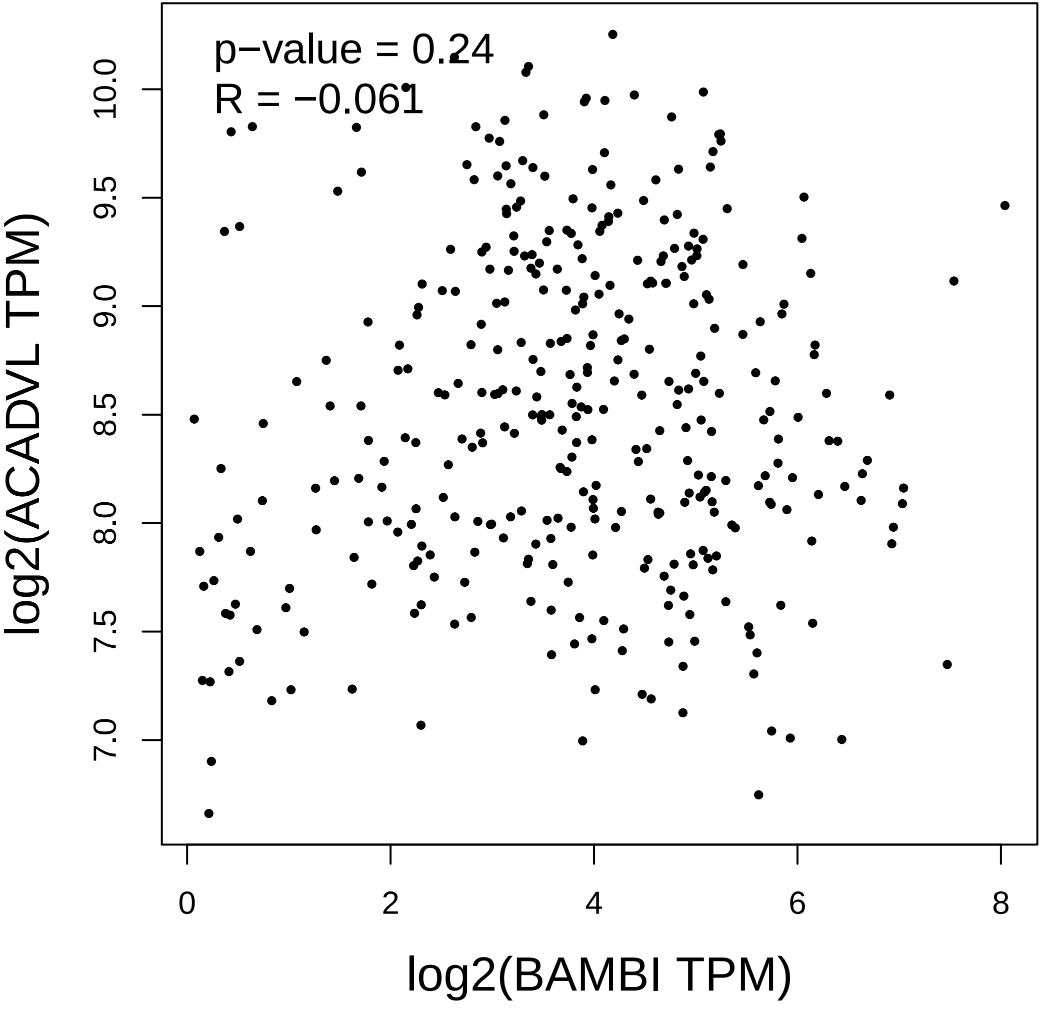

p−value = 0.24
R = −0.061
log2(ACADVL TPM)
10.0
9.5
9.0
8.5
8.0
7.5
7.0
0
2
4
6
8
log2(BAMBI TPM)

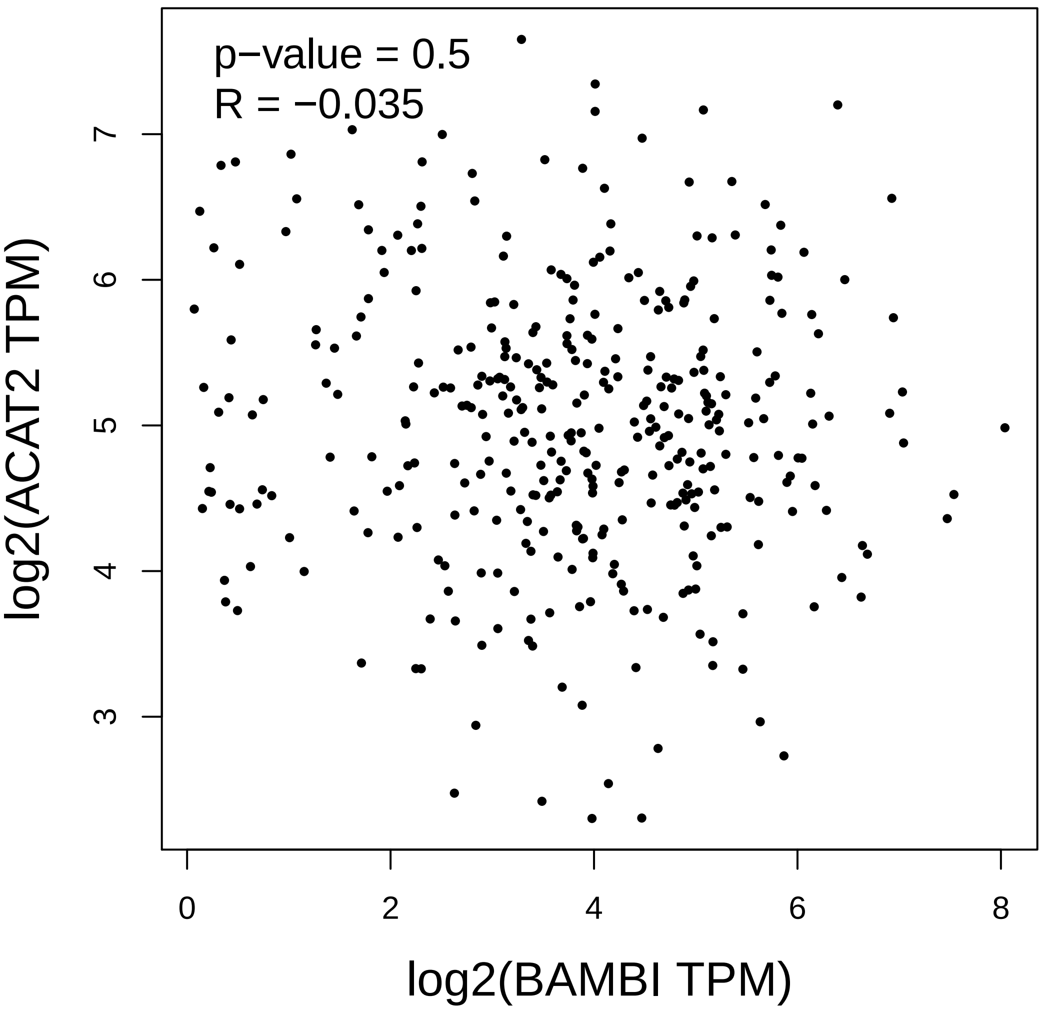
p−value = 0.5
R = −0.035
log2(ACAT2 TPM)
3
4
5
6
7
0
2
4
6
8
log2(BAMBI TPM)

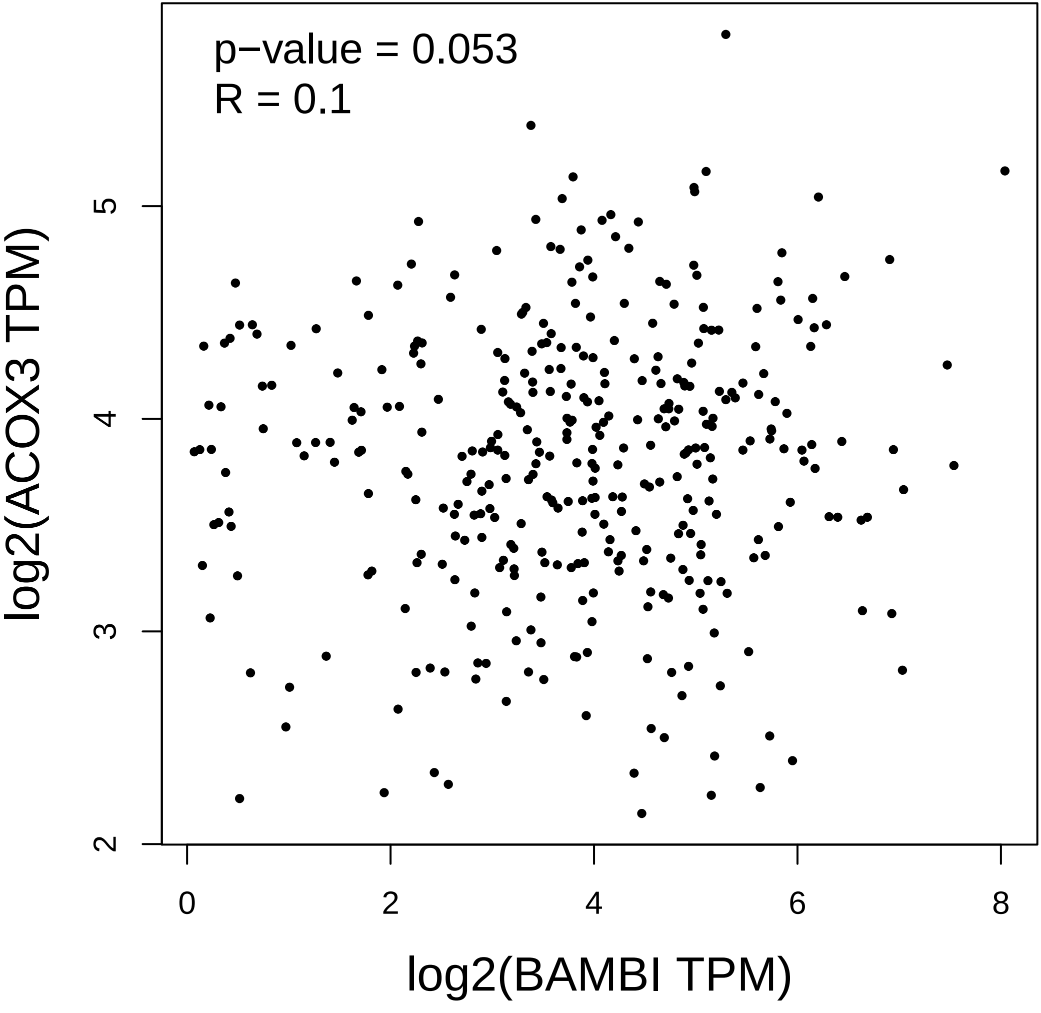

p−value = 0.053
R = 0.1
log2(ACOX3 TPM)
2
3
4
5
0
2
4
6
8
log2(BAMBI TPM)

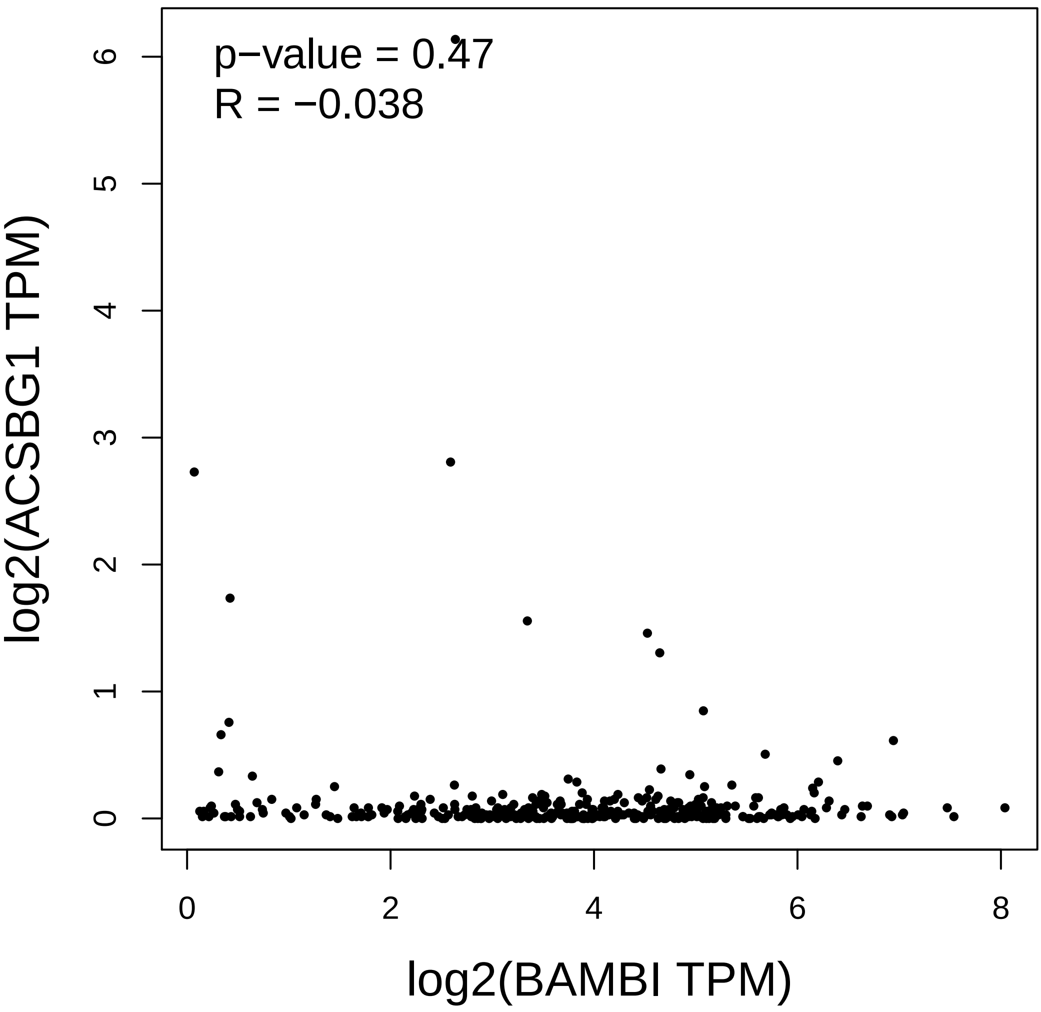
p−value = 0.47
R = −0.038
log2(ACSBG1 TPM)
log2(BAMBI TPM)
0
1
2
3
4
5
6
0
2
4
6
8

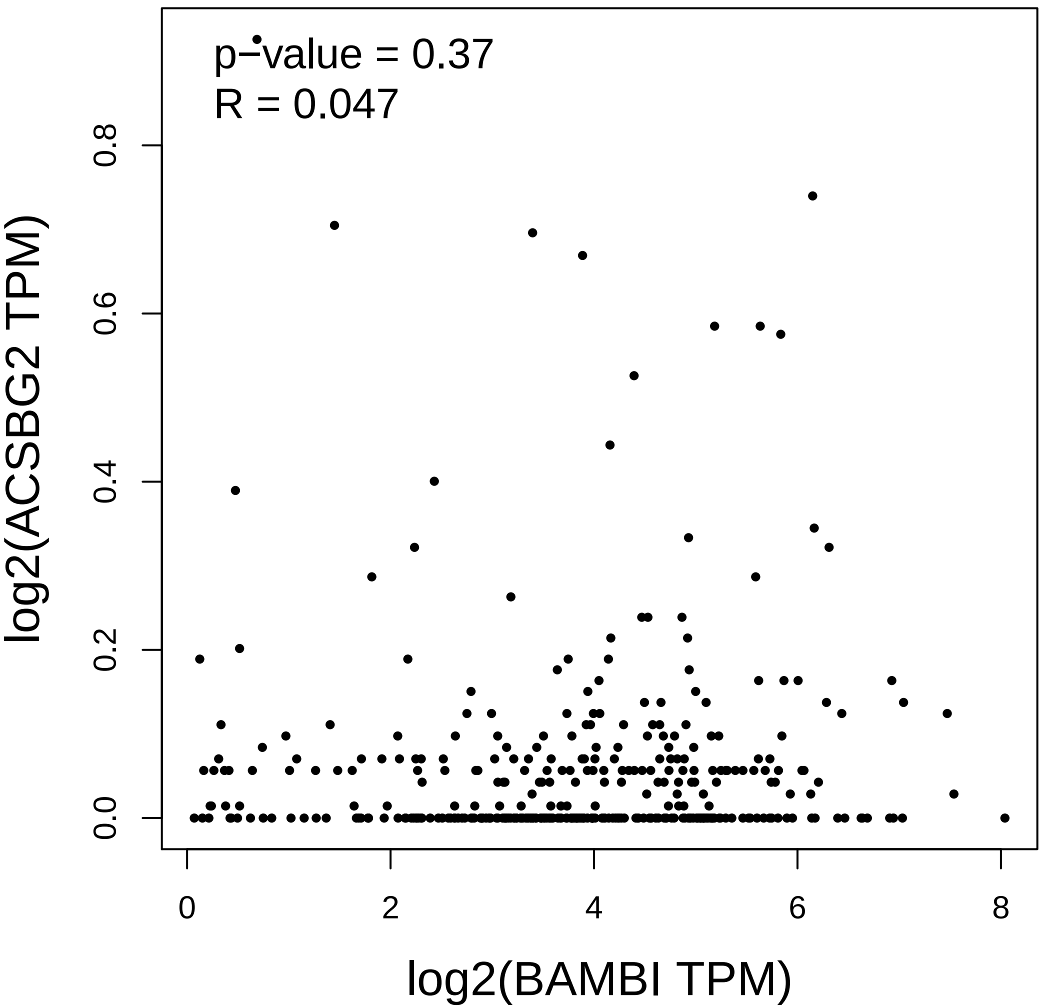

p−value = 0.37
R = 0.047
log2(ACSBG2 TPM)
0.0
0.2
0.4
0.6
0.8
0
2
4
6
8
log2(BAMBI TPM)

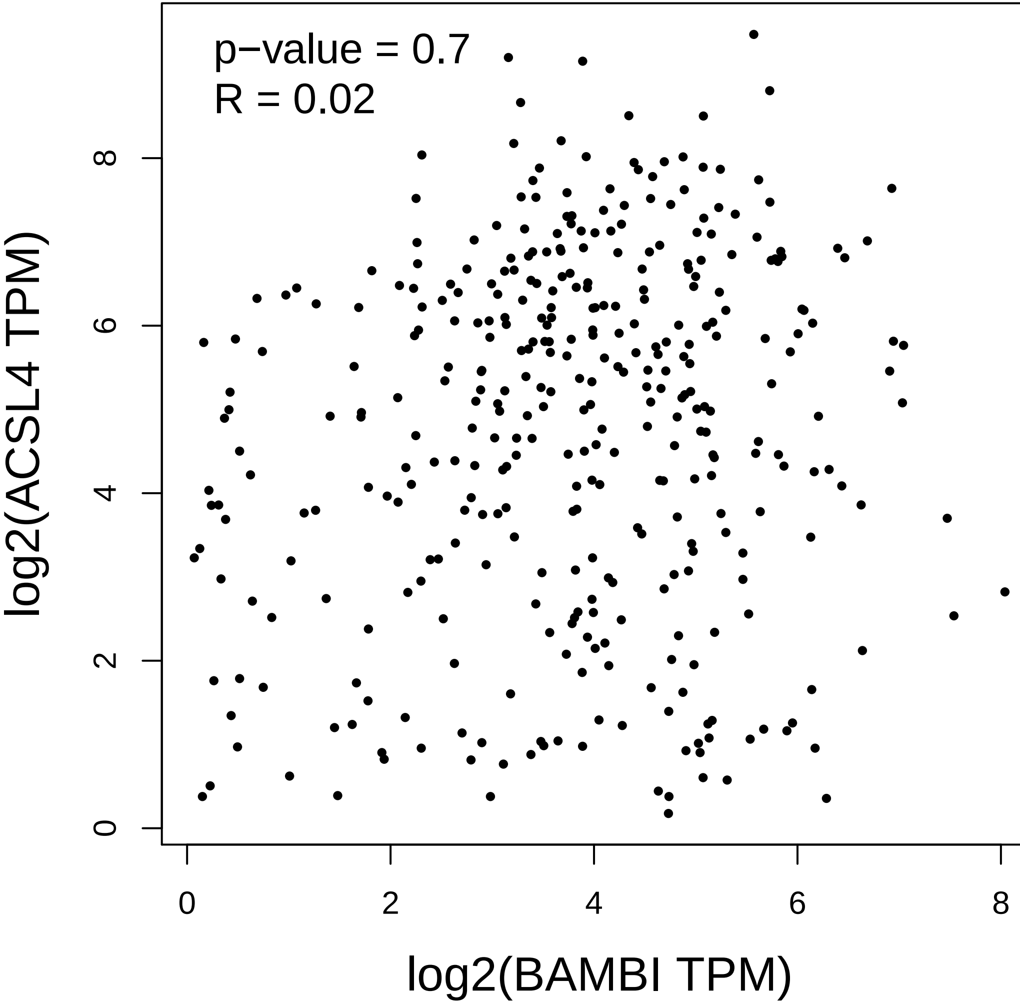
p−value = 0.7
R = 0.02
log2(ACSL4 TPM)
0
2
4
6
8
log2(BAMBI TPM)
0
2
4
6
8

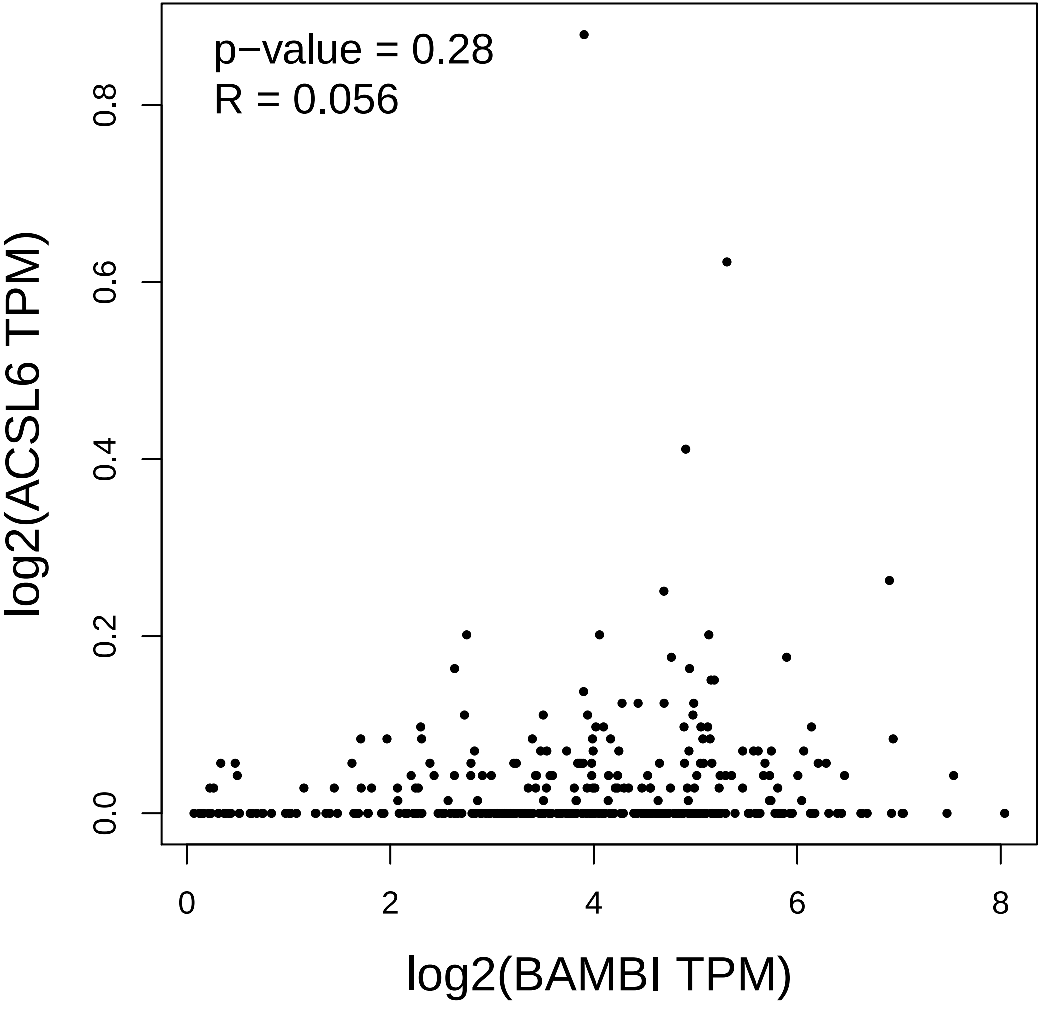

p−value = 0.28
R = 0.056
log2(ACSL6 TPM)
0.0
0.2
0.4
0.6
0.8
0
2
4
6
8
log2(BAMBI TPM)

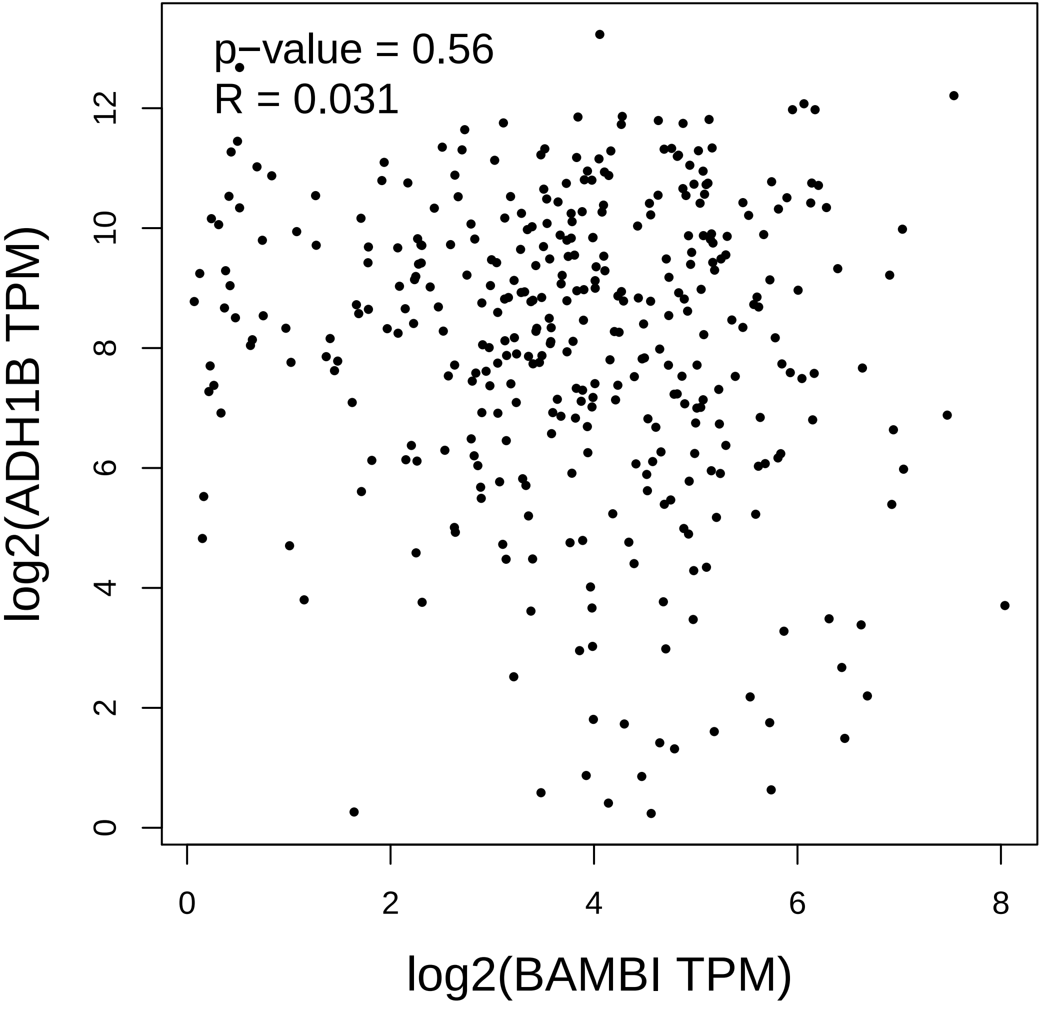
p−value = 0.56
R = 0.031
log2(ADH1B TPM)
log2(BAMBI TPM)
0
2
4
6
8
10
12

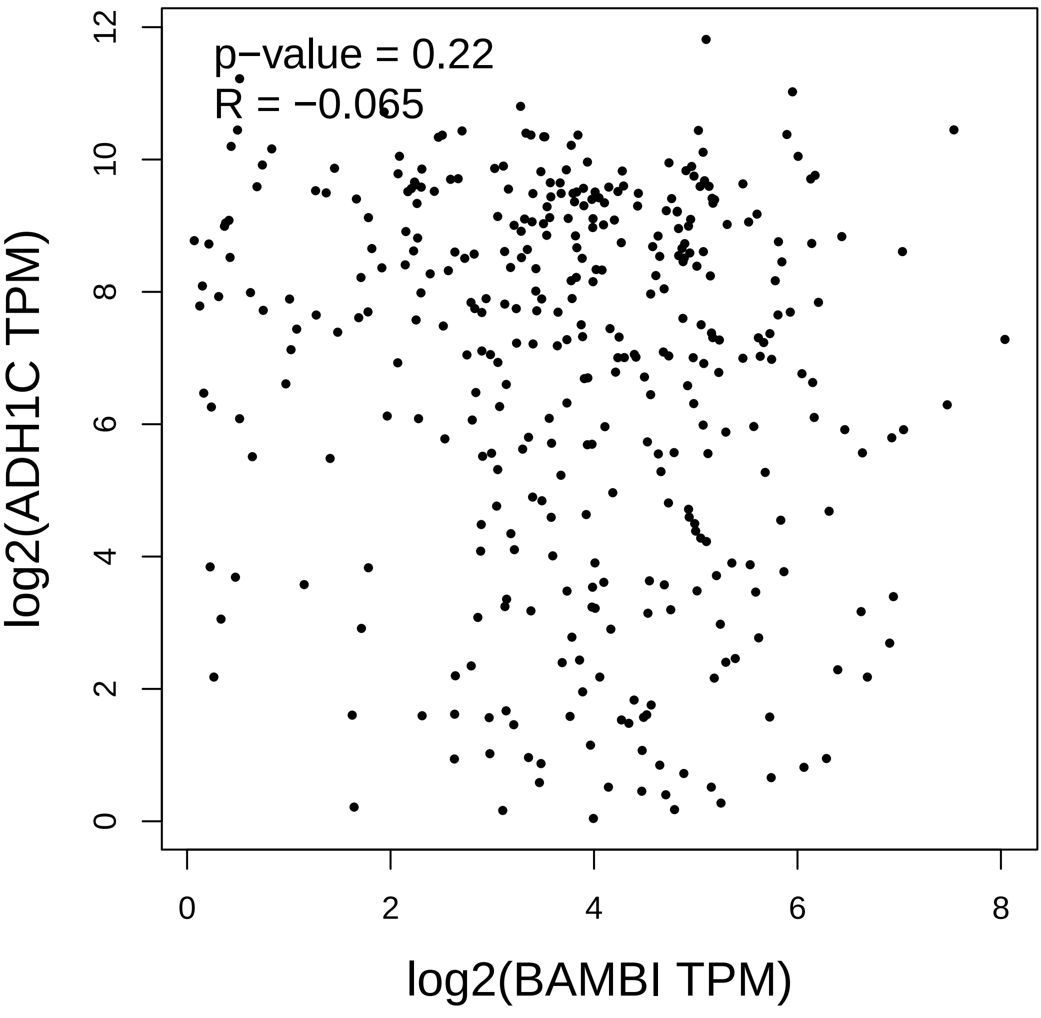

p−value = 0.22
R = −0.065
log2(ADH1C TPM)
log2(BAMBI TPM)
0
2
4
6
8
10
12

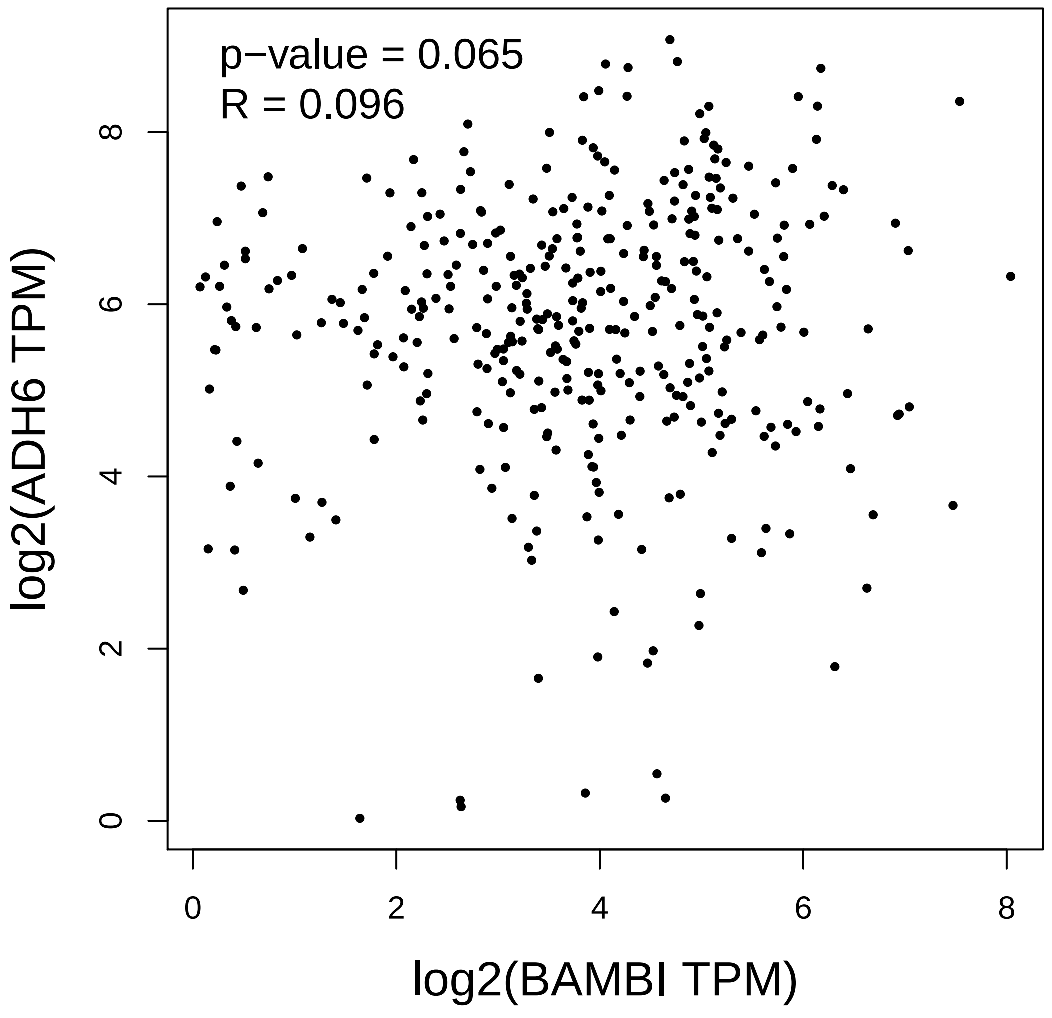
p−value = 0.065
R = 0.096
log2(ADH6 TPM)
0
2
4
6
8
log2(BAMBI TPM)
0
2
4
6
8

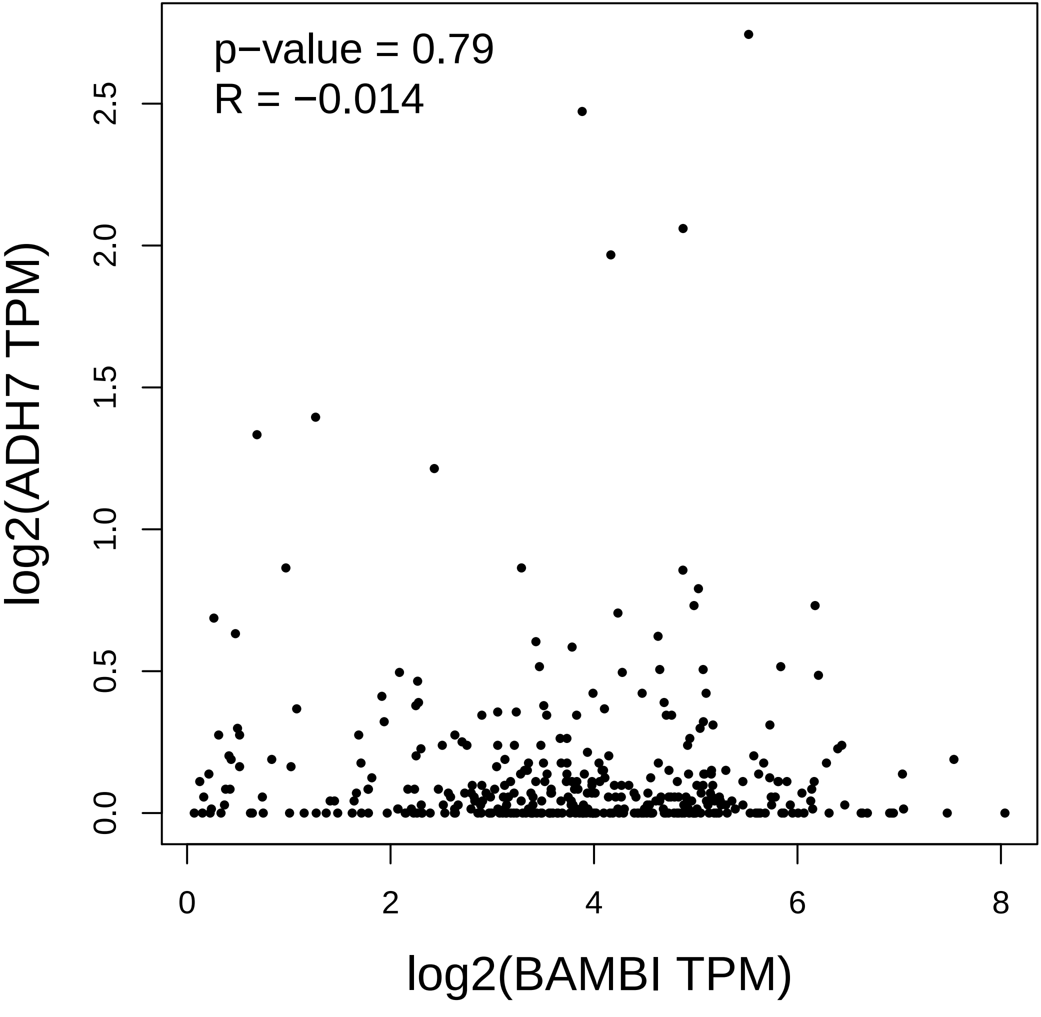
p−value = 0.79
R = −0.014
log2(ADH7 TPM)
log2(BAMBI TPM)
0.0
0.5
1.0
1.5
2.0
2.5
0
2
4
6
8

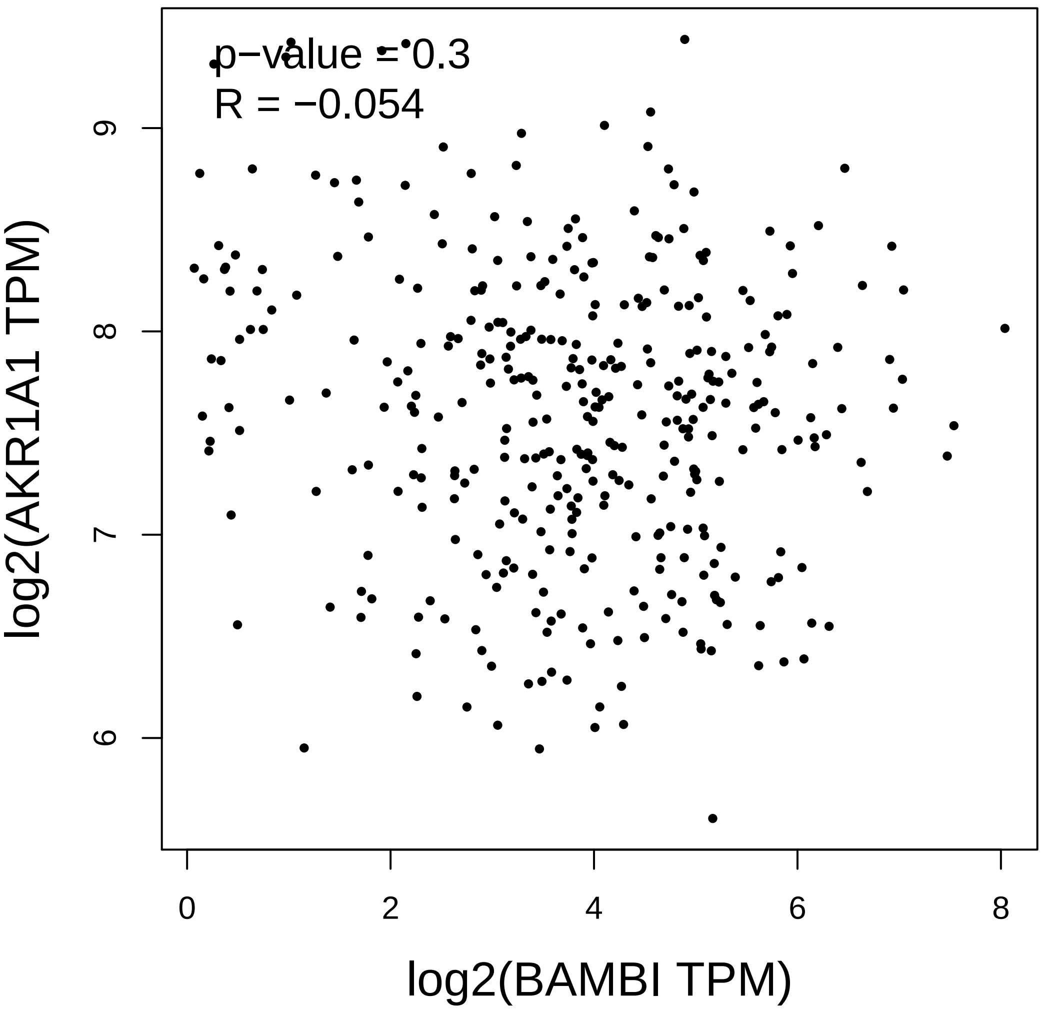
p−value = 0.3
R = −0.054
9
8
7
6
log2(AKR1A1 TPM)
0
2
4
6
8
log2(BAMBI TPM)

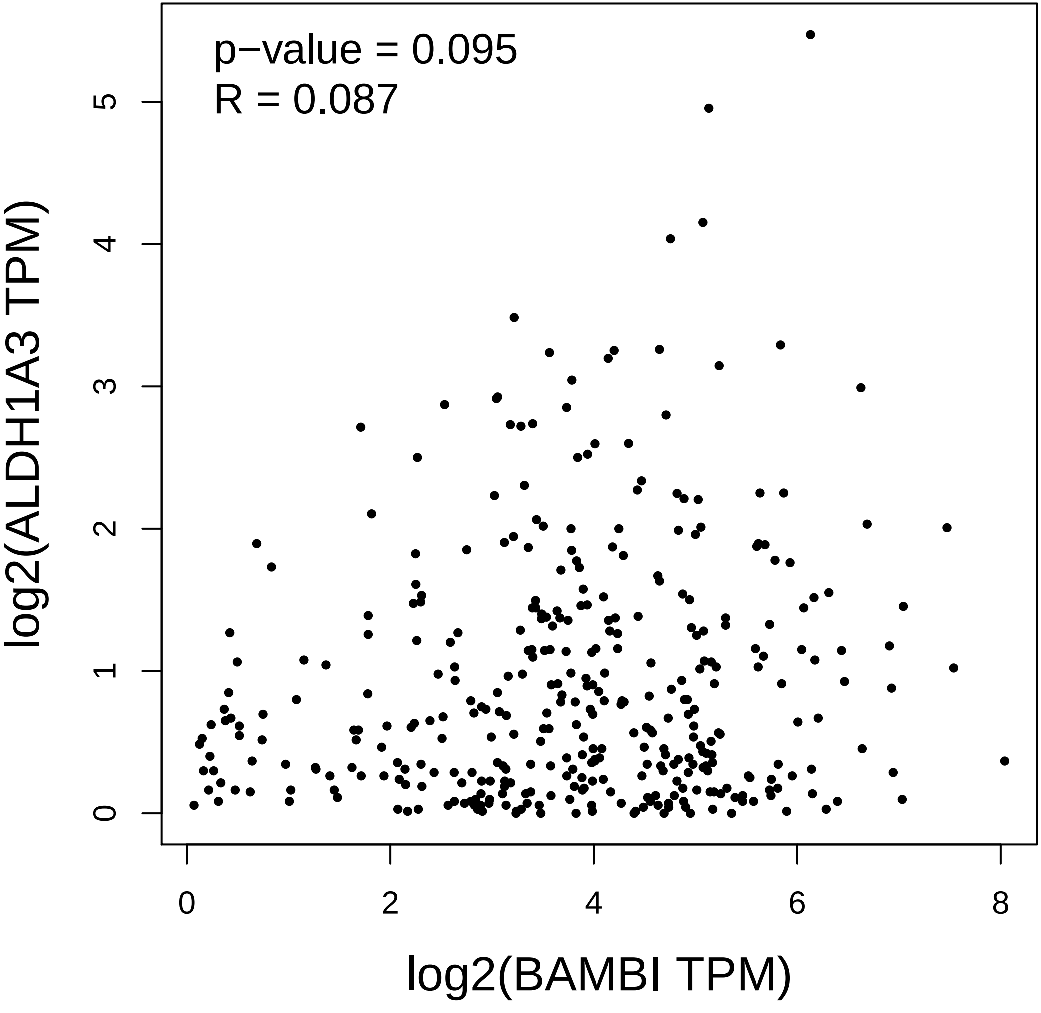
p−value = 0.095
R = 0.087
log2(ALDH1A3 TPM)
0
1
2
3
4
5
0
2
4
6
8
log2(BAMBI TPM)

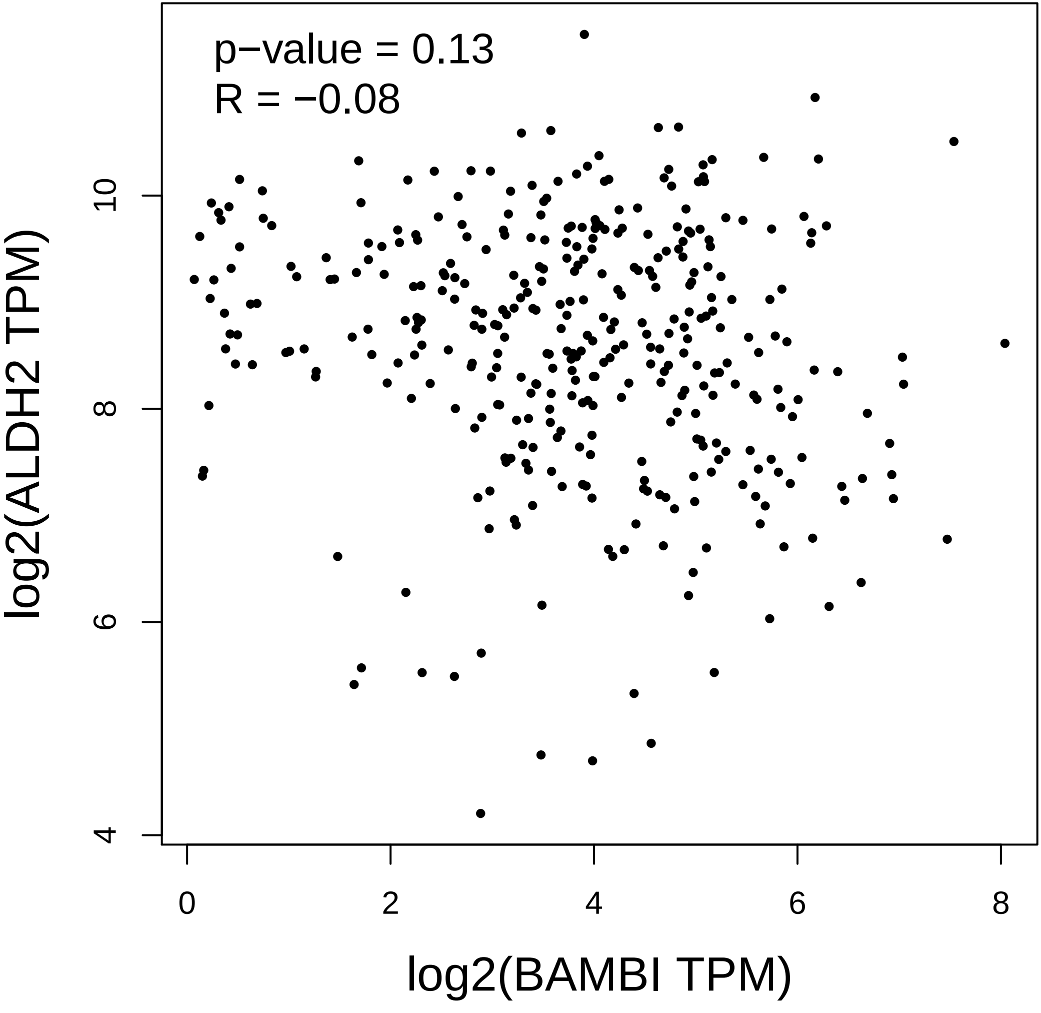
p−value = 0.13
R = −0.08
log2(ALDH2 TPM)
4
6
8
10
0
2
4
6
8
log2(BAMBI TPM)

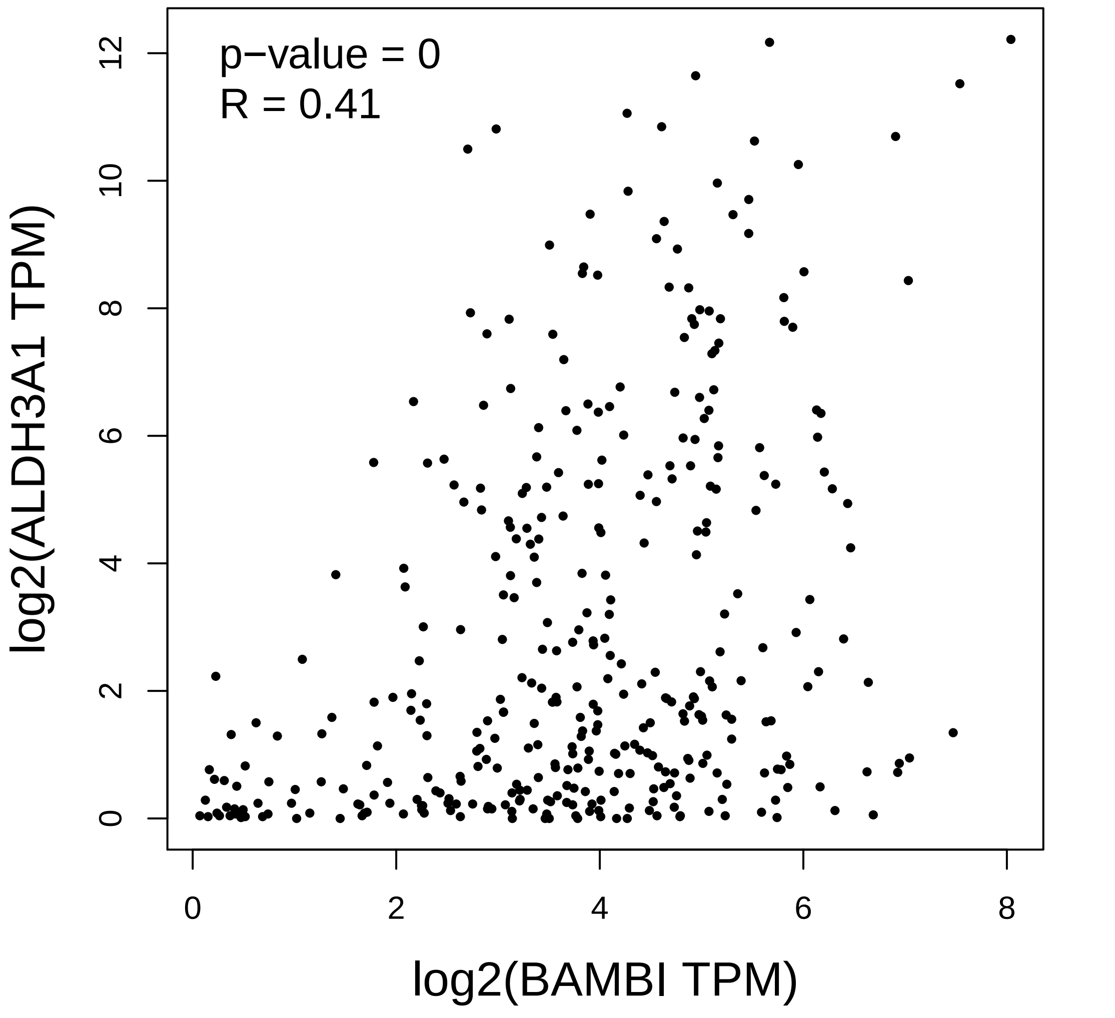
p−value = 0
R = 0.41
log2(ALDH3A1 TPM)
log2(BAMBI TPM)
0
2
4
6
8
10
12

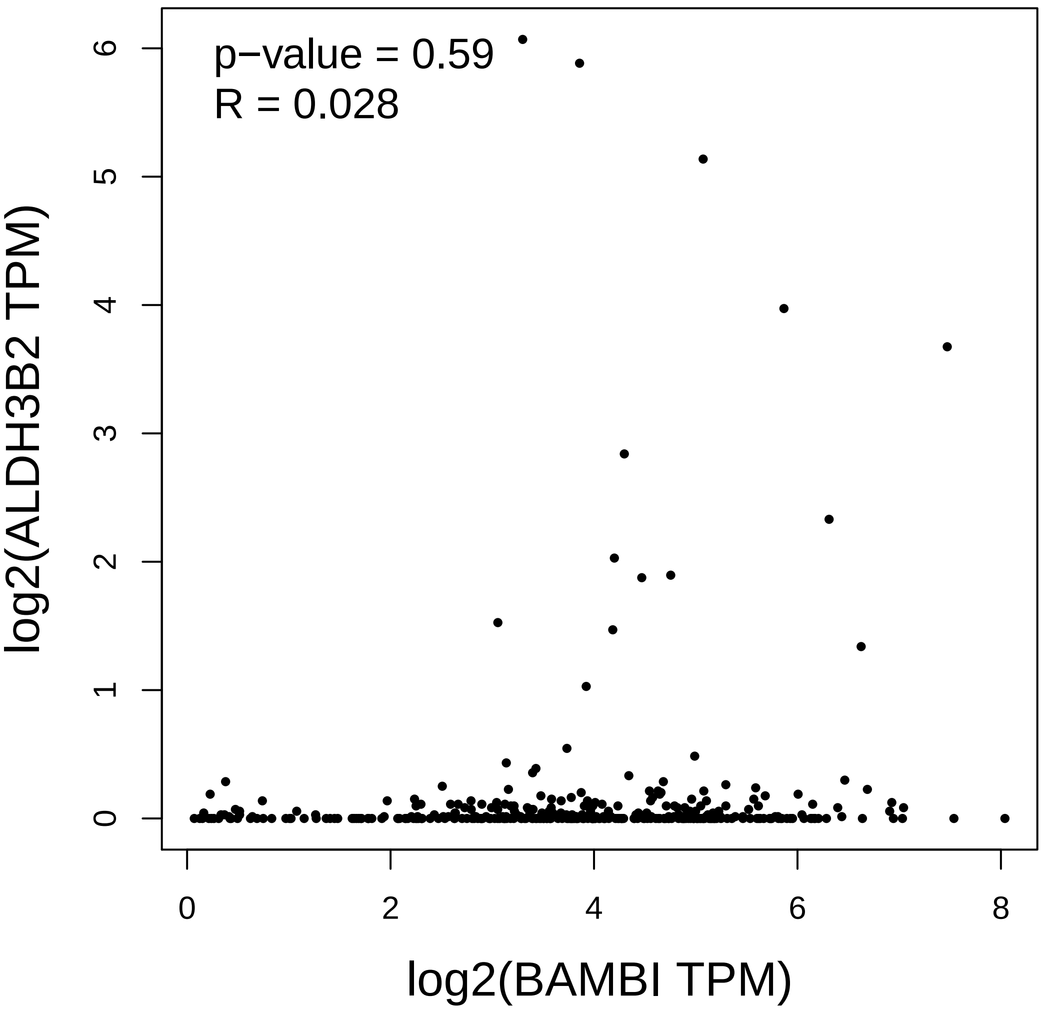
p−value = 0.59
R = 0.028
log2(ALDH3B2 TPM)
log2(BAMBI TPM)
0
1
2
3
4
5
6
0
2
4
6
8

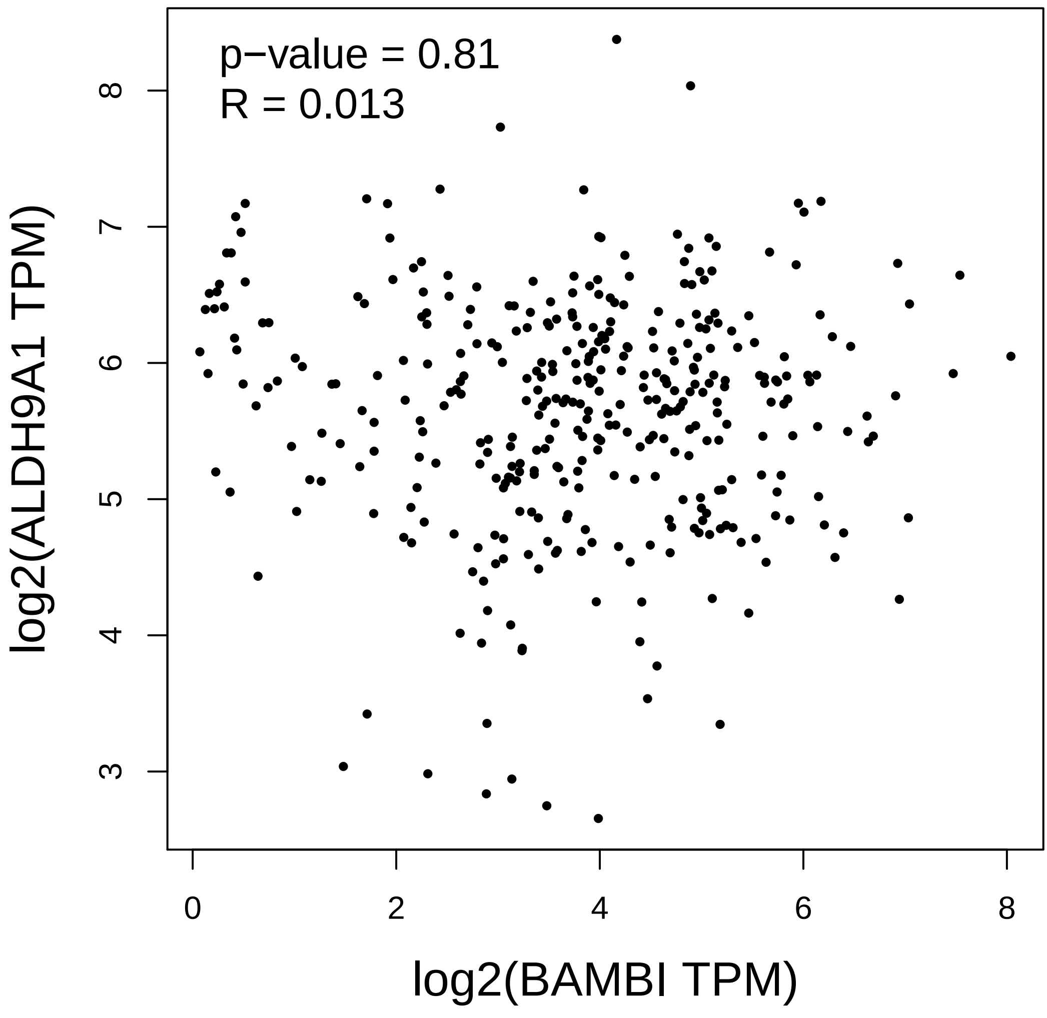
p−value = 0.81
R = 0.013
log2(ALDH9A1 TPM)
3
4
5
6
7
8
0
2
4
6
8
log2(BAMBI TPM)

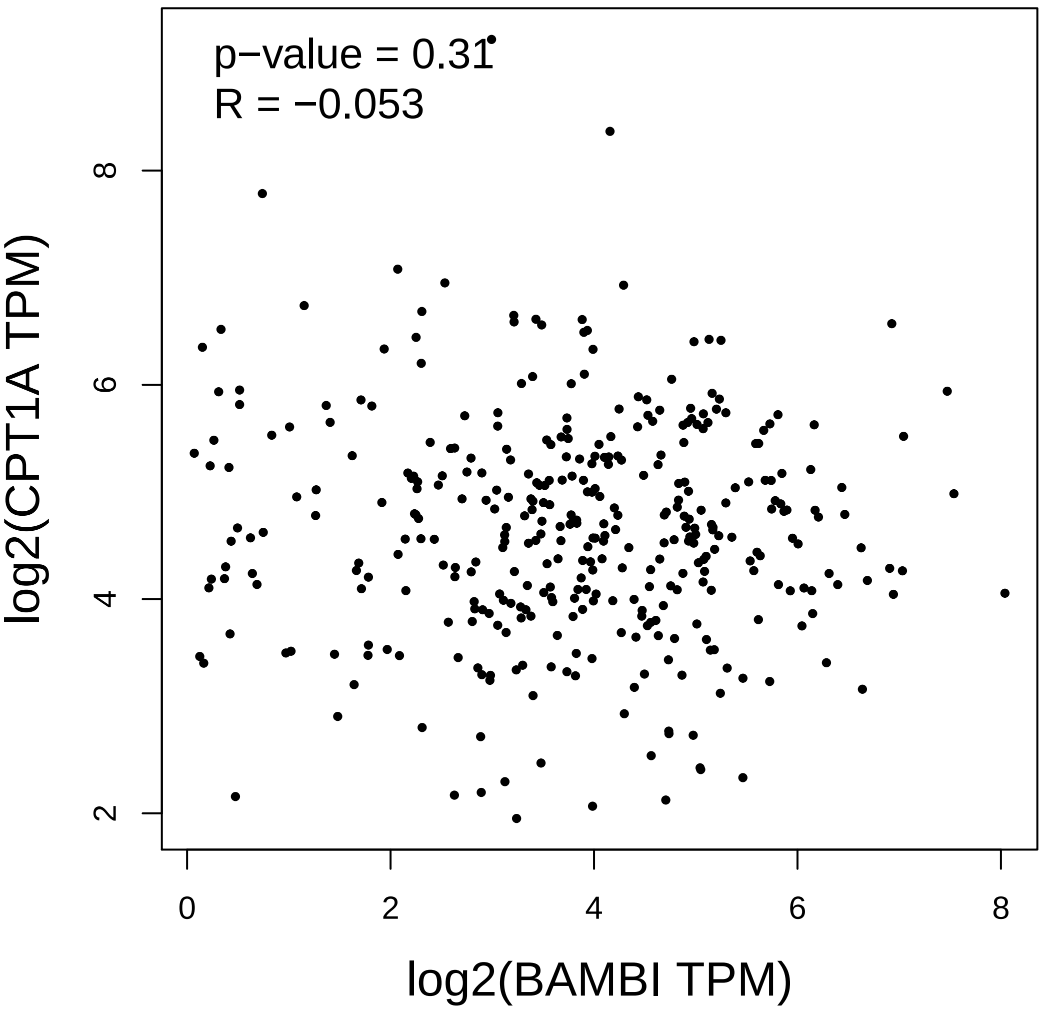
p−value = 0.31
R = −0.053
8
6
4
2
log2(CPT1A TPM)
0
2
4
6
8
log2(BAMBI TPM)

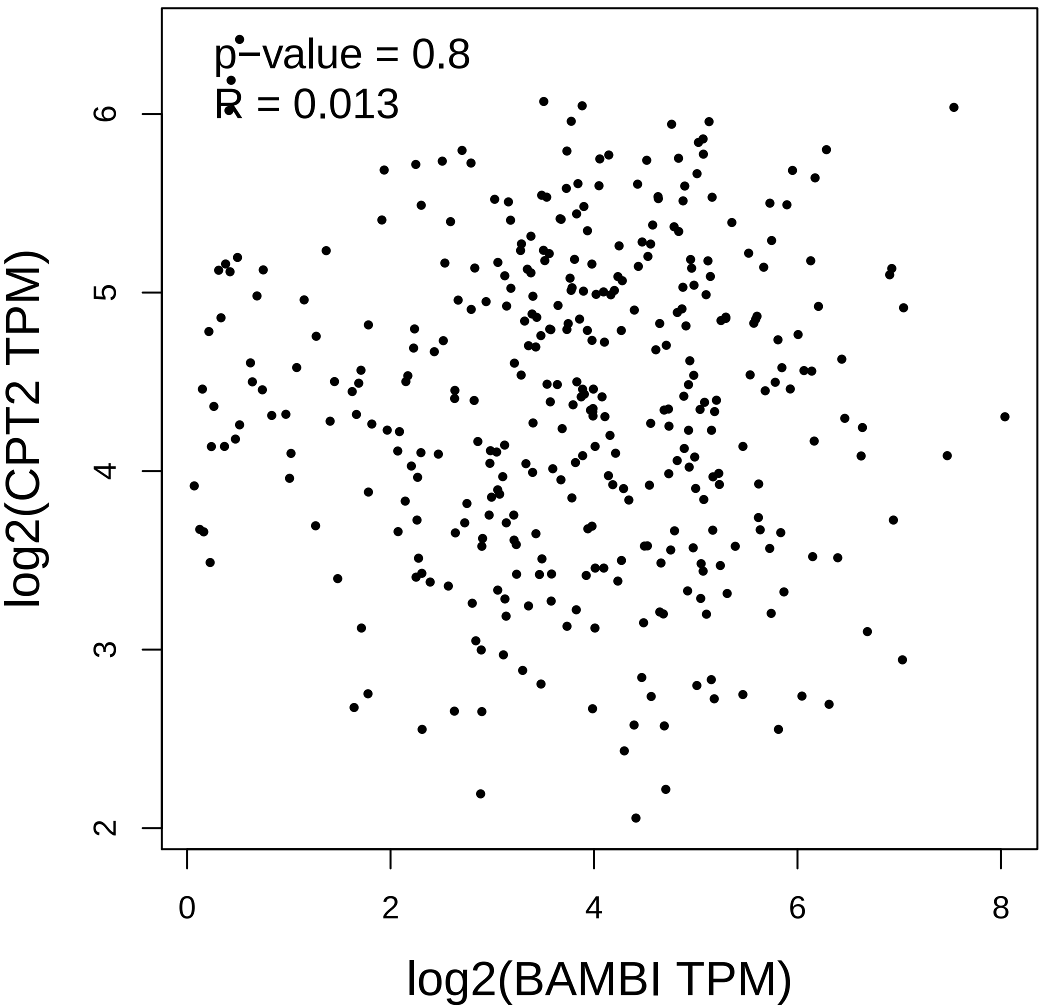
p−value = 0.8
R = 0.013
log2(CPT2 TPM)
log2(BAMBI TPM)
0
2
4
6
8
2
3
4
5
6

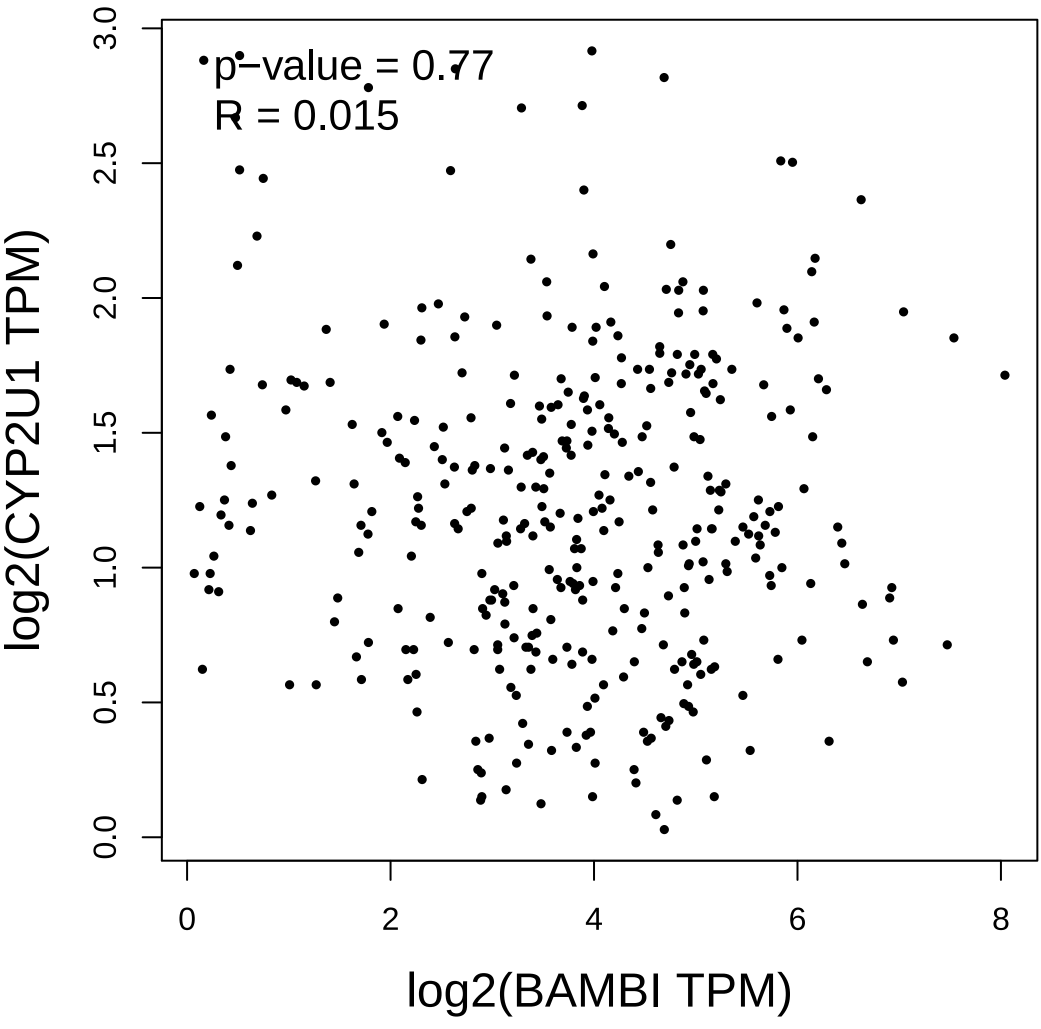

p−value = 0.77
R = 0.015
log2(CYP2U1 TPM)
0.0
0.5
1.0
1.5
2.0
2.5
3.0
0
2
4
6
8
log2(BAMBI TPM)

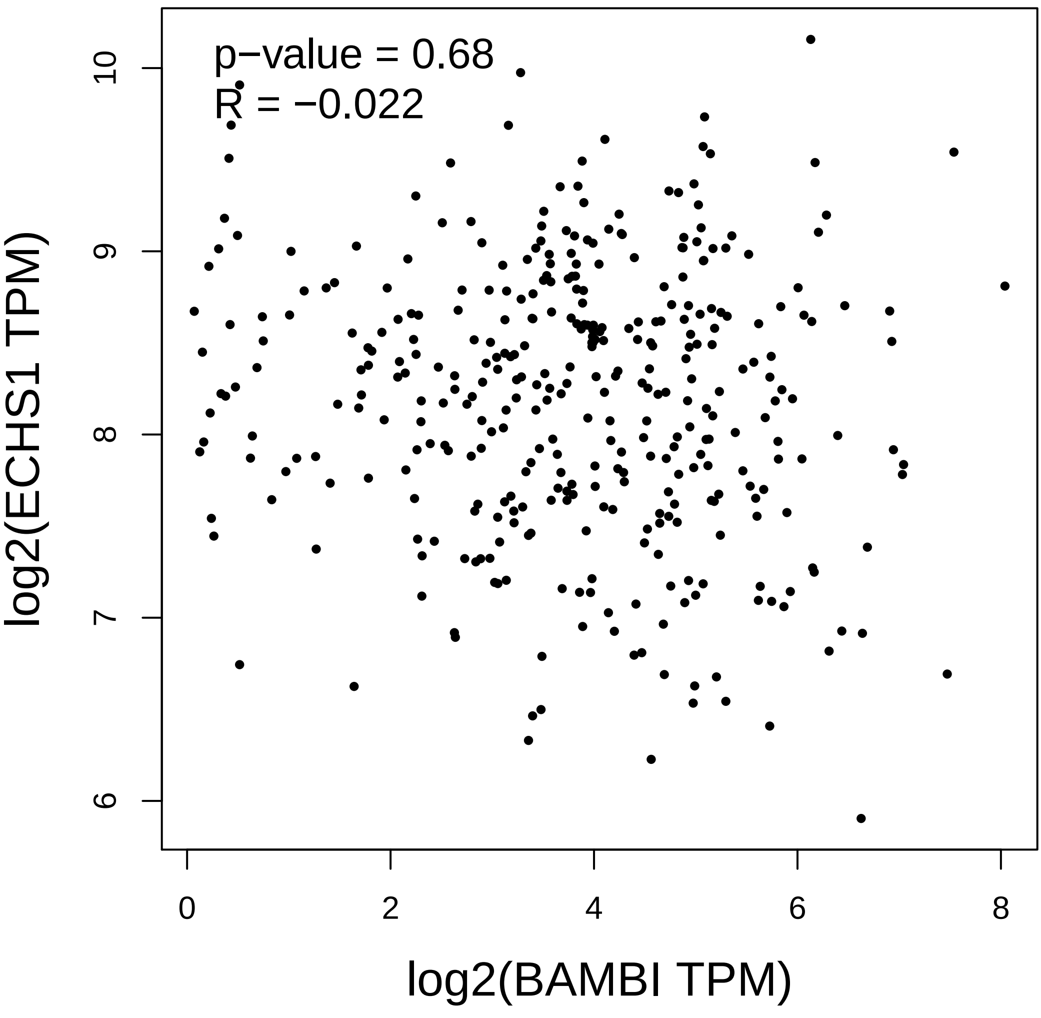
p−value = 0.68
R = −0.022
log2(ECHS1 TPM)
log2(BAMBI TPM)
10
9
8
7
6
0
2
4
6
8

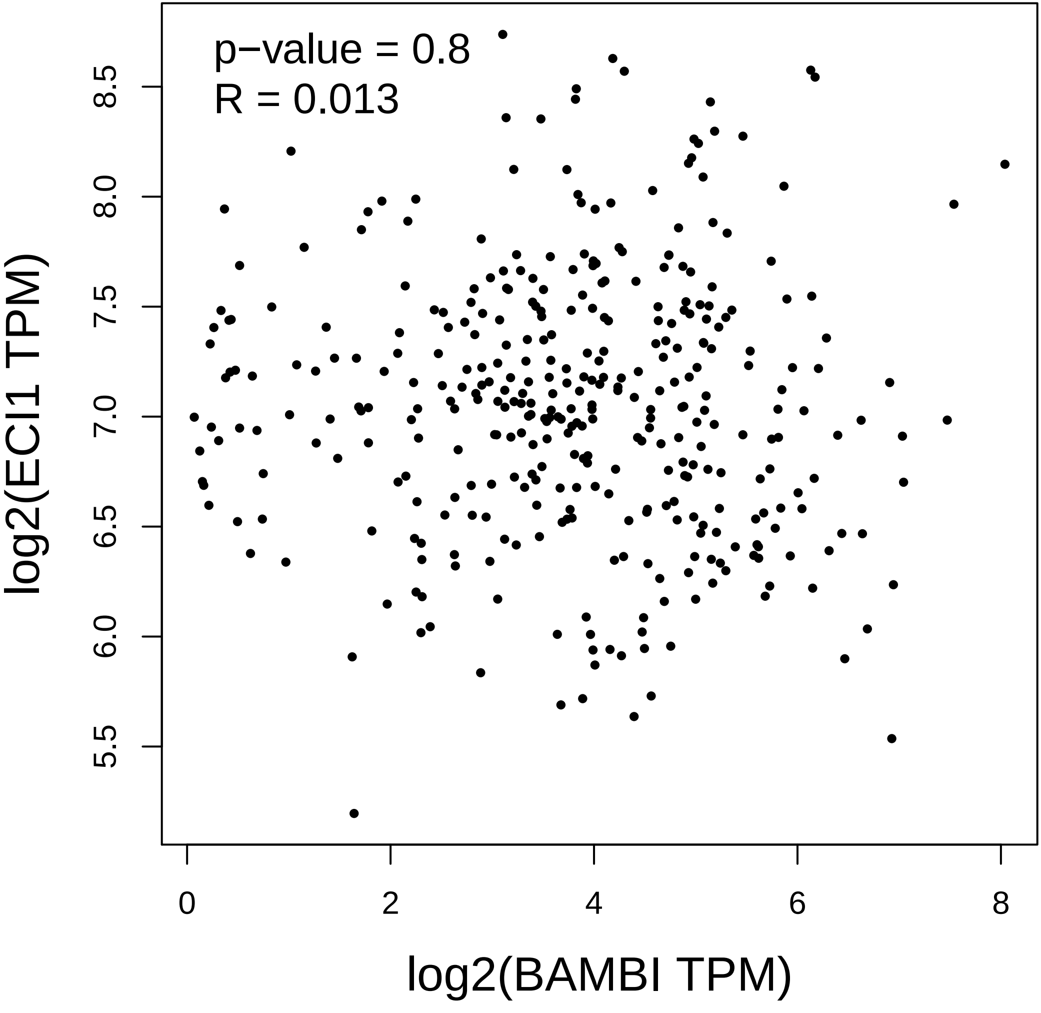

p−value = 0.8
R = 0.013
log2(ECI1 TPM)
log2(BAMBI TPM)
5.5
6.0
6.5
7.0
7.5
8.0
8.5
0
2
4
6
8

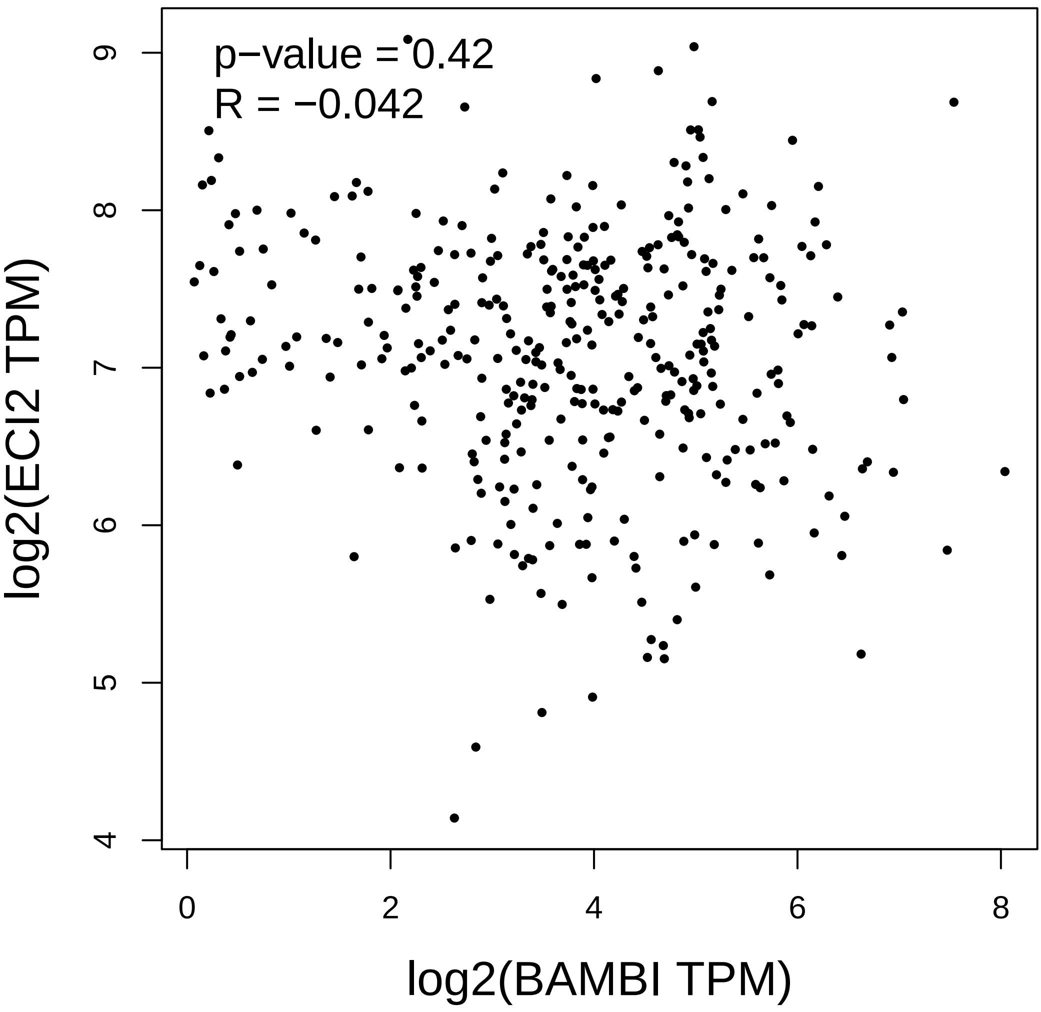

p−value = 0.42
R = −0.042
9
8
7
6
5
4
0
2
4
6
8
log2(ECI2 TPM)
log2(BAMBI TPM)

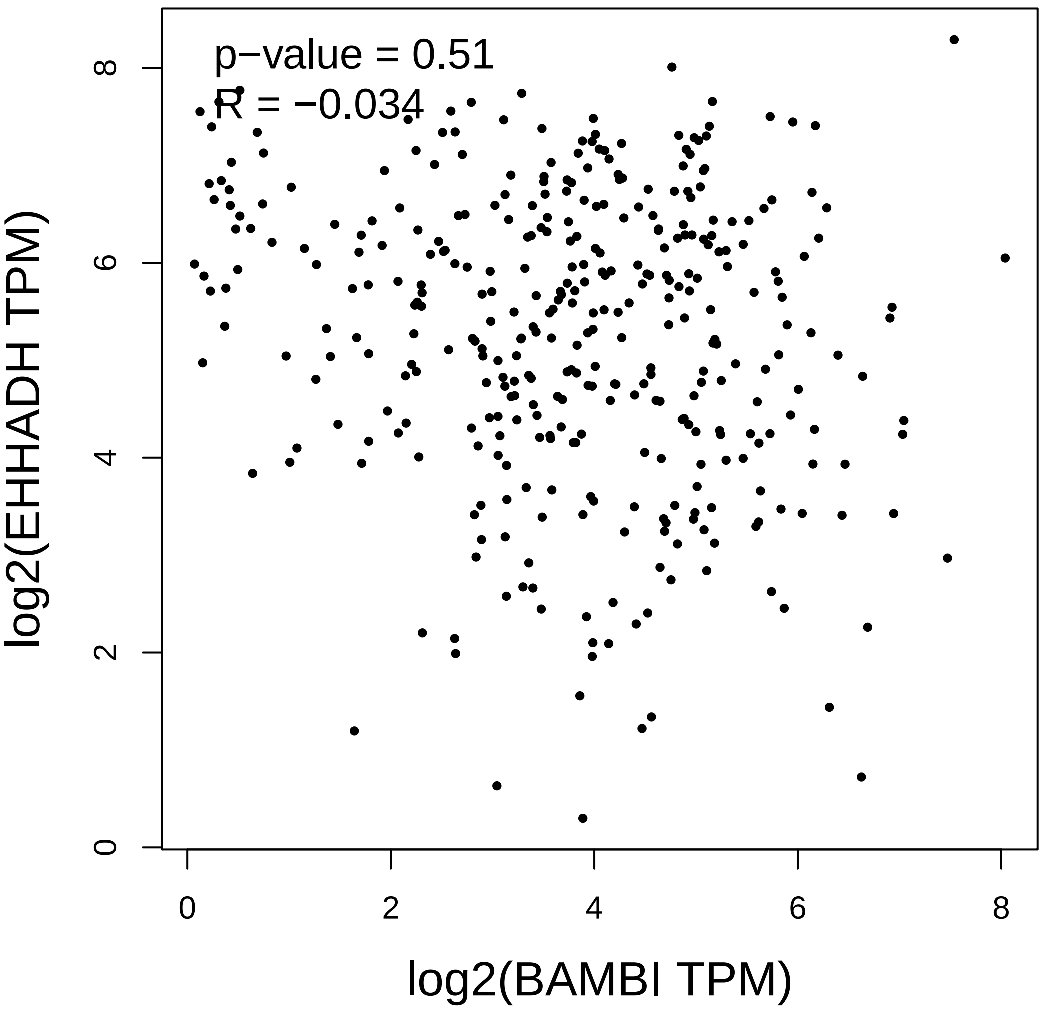
p−value = 0.51
R = −0.034
log2(EHHADH TPM)
log2(BAMBI TPM)
0
2
4
6
8

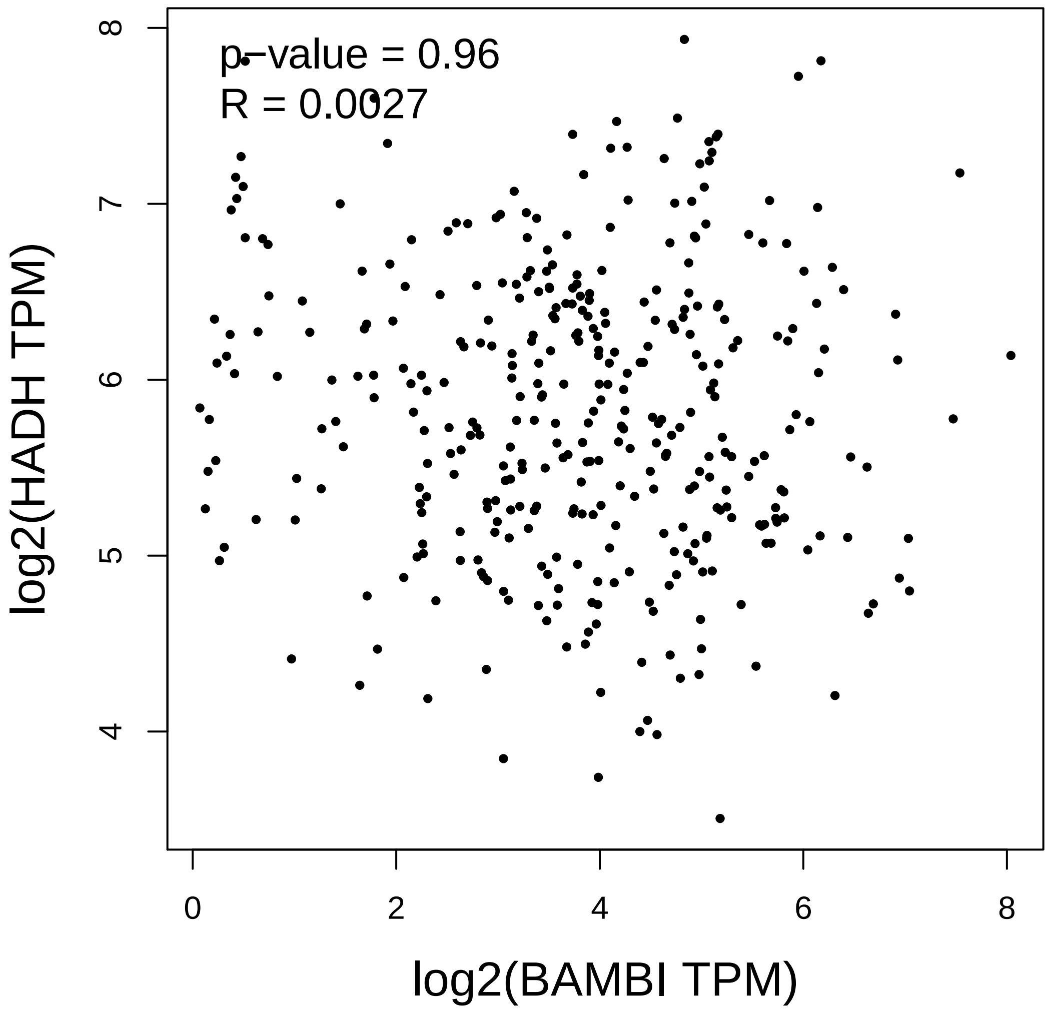
p−value = 0.96
R = 0.0027
log2(HADH TPM)
log2(BAMBI TPM)
0
2
4
6
8
4
5
6
7
8

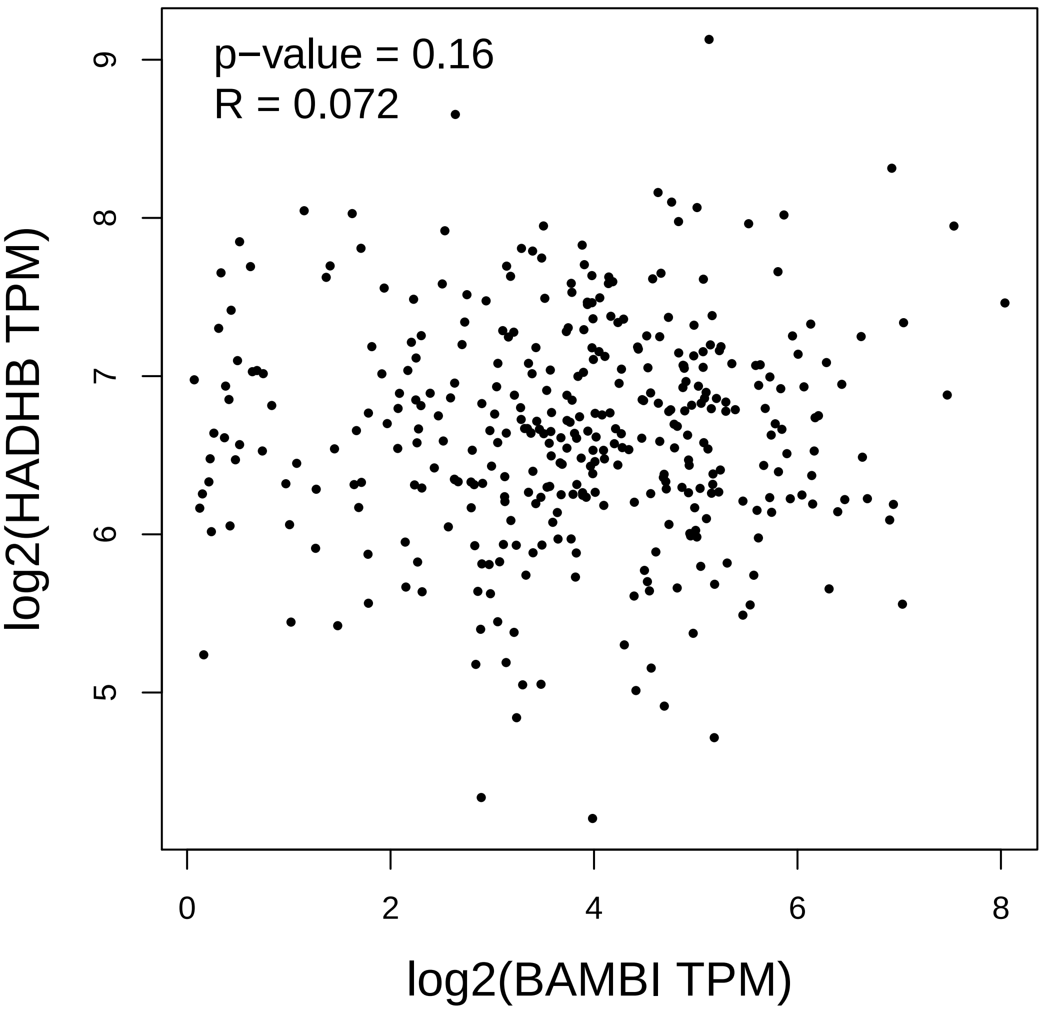
p−value = 0.16
R = 0.072
log2(HADHB TPM)
log2(BAMBI TPM)
5
6
7
8
9
0
2
4
6
8
