# Supplementary material for: BAMBI Is a Prognostic Biomarker Associated with Macrophage Polarization, Glycolysis, and Lipid Metabolism in Hepatocellular Carcinoma
Source: Int J Mol Sci. 2024 Nov 26;25(23):12713. doi: 10.3390/ijms252312713 (PMC11640931; doi:10.3390/ijms252312713)
Supplement: Supplementary file 1 [file ijms-25-12713-s001.zip › Correlation of BAMBI and 62 genes in the glycolysis gluconeogenesis pathway.pdf]

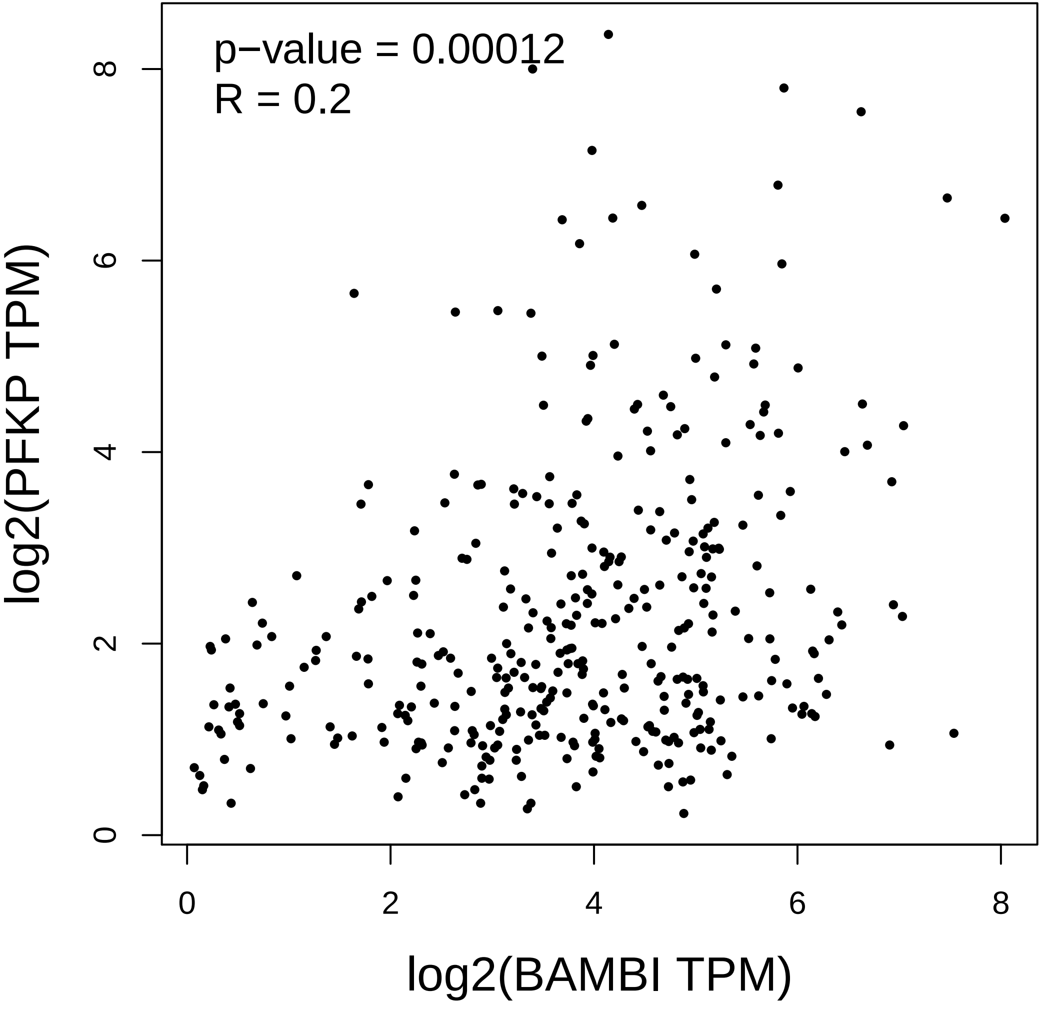
p−value = 0.00012
R = 0.2
log2(PFKP TPM)
log2(BAMBI TPM)
0
2
4
6
8
0
2
4
6
8

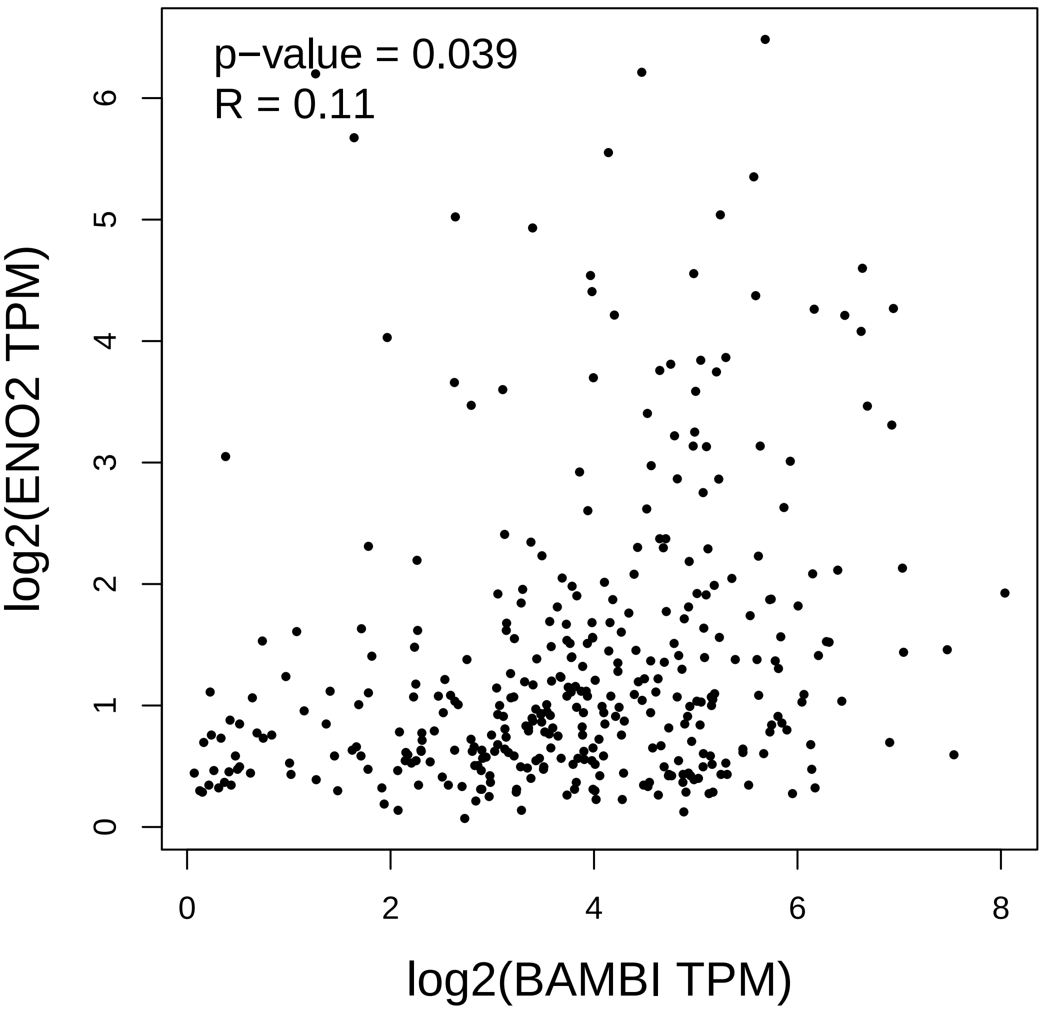

p−value = 0.039
R = 0.11
log2(ENO2 TPM)
0
1
2
3
4
5
6
log2(BAMBI TPM)
0
2
4
6
8

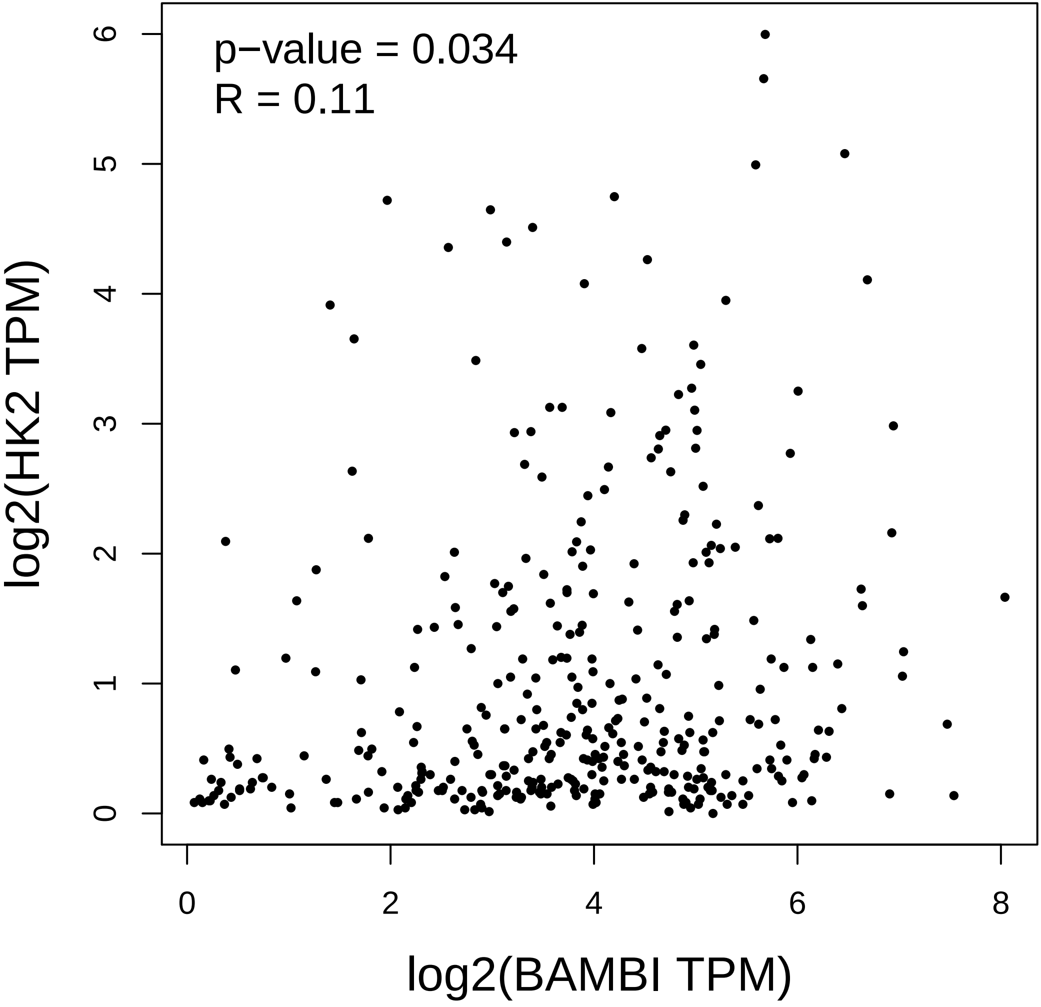
p−value = 0.034
R = 0.11
log2(HK2 TPM)
0
1
2
3
4
5
6
log2(BAMBI TPM)
0
2
4
6
8

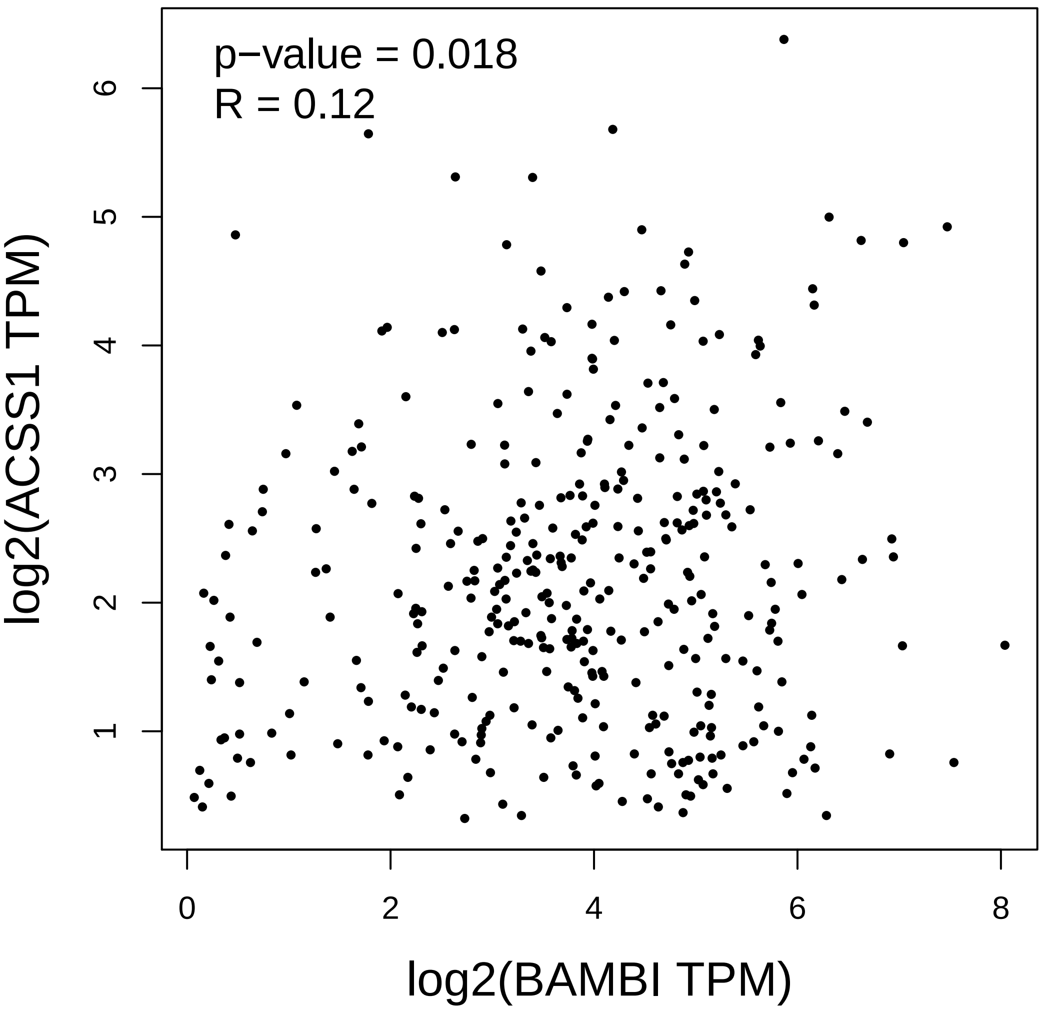
p−value = 0.018
R = 0.12
log2(ACSS1 TPM)
log2(BAMBI TPM)
0
2
4
6
8
1
2
3
4
5
6

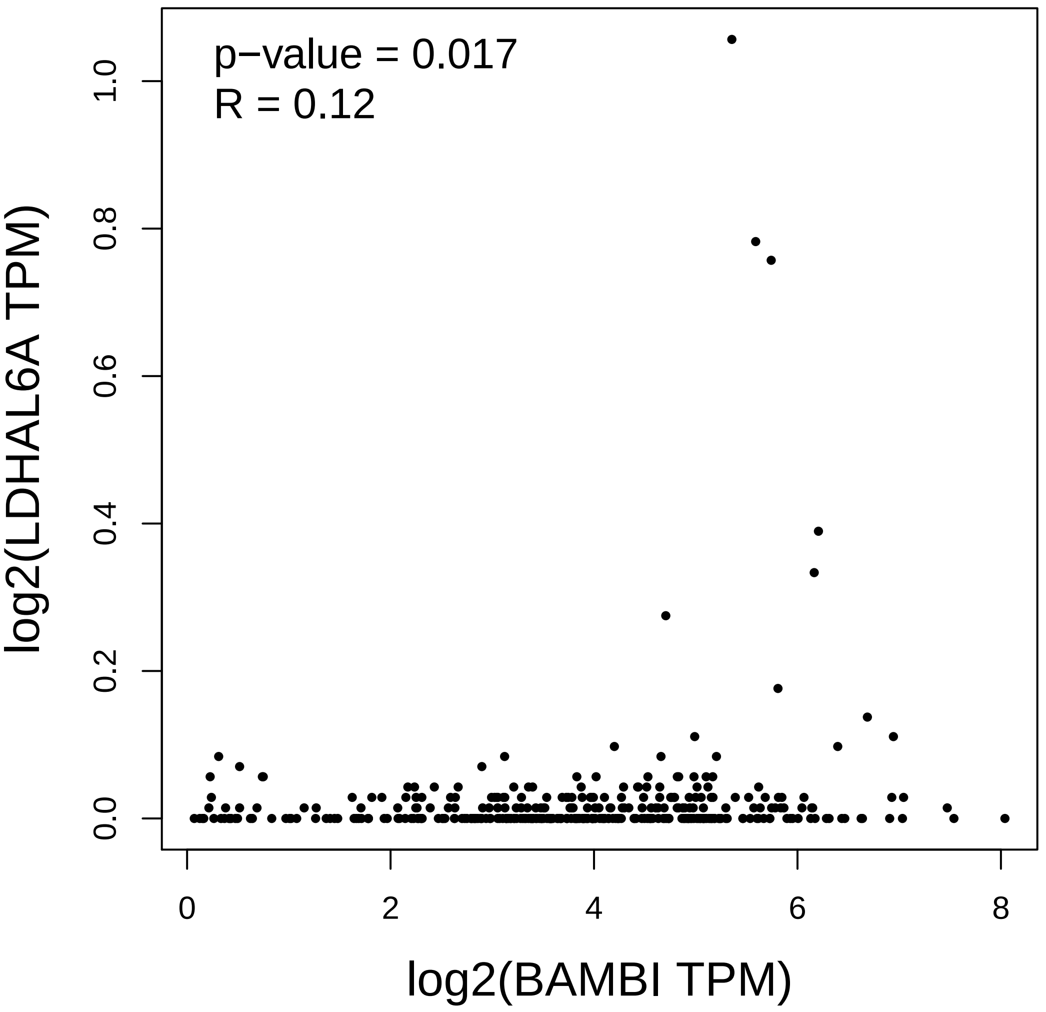
p−value = 0.017
R = 0.12
log2(LDHAL6A TPM)
log2(BAMBI TPM)
0.0
0.2
0.4
0.6
0.8
1.0
0
2
4
6
8

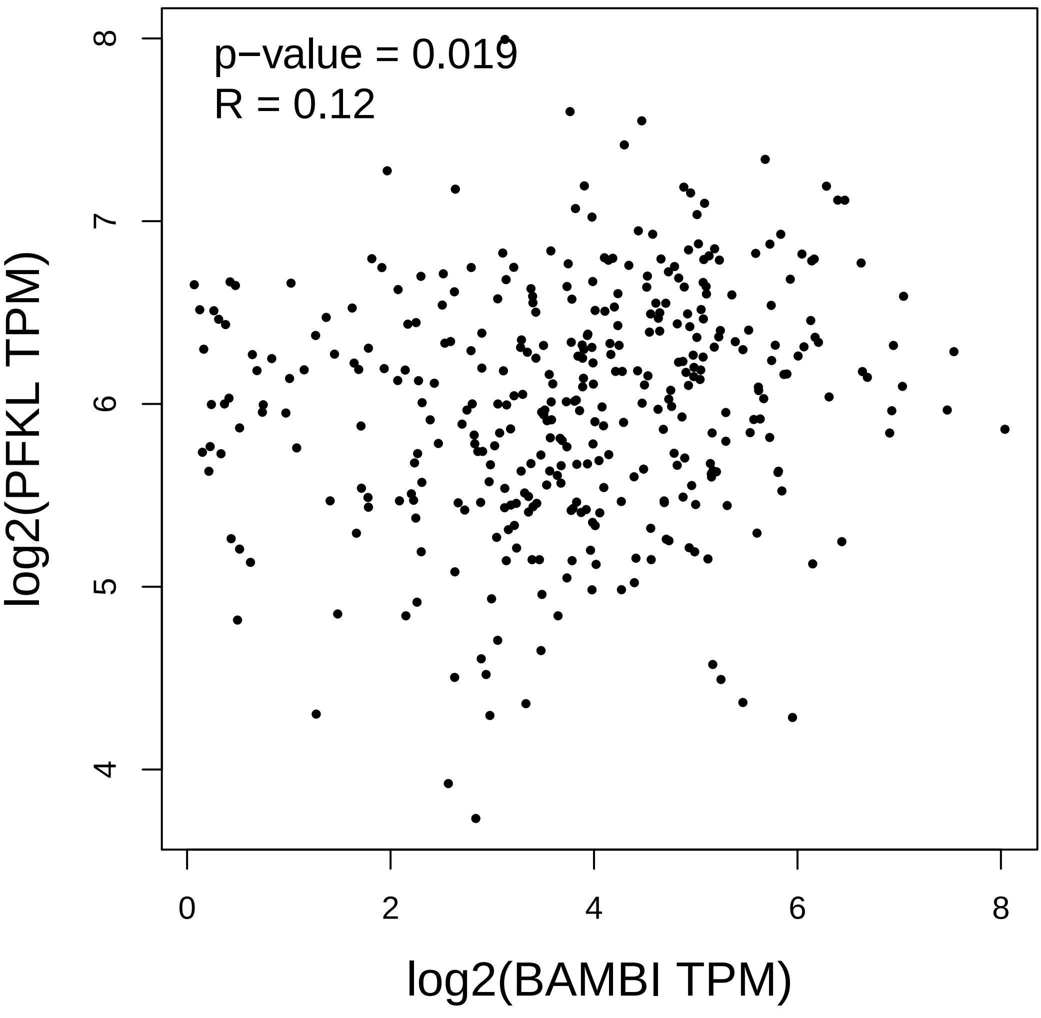
p−value = 0.019
R = 0.12
log2(PFKL TPM)
log2(BAMBI TPM)
4
5
6
7
8
0
2
4
6
8

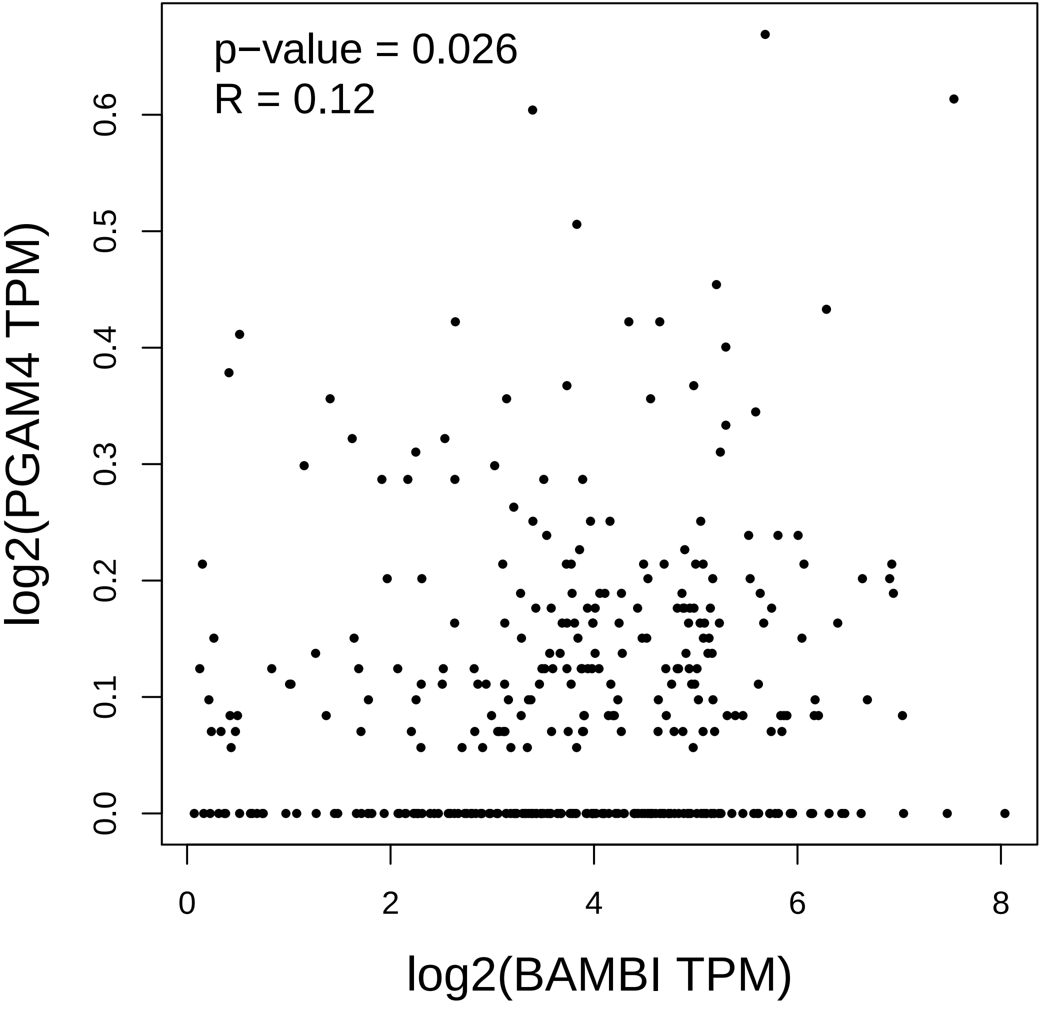
p−value = 0.026
R = 0.12
log2(PGAM4 TPM)
log2(BAMBI TPM)
0.0
0.1
0.2
0.3
0.4
0.5
0.6
0
2
4
6
8

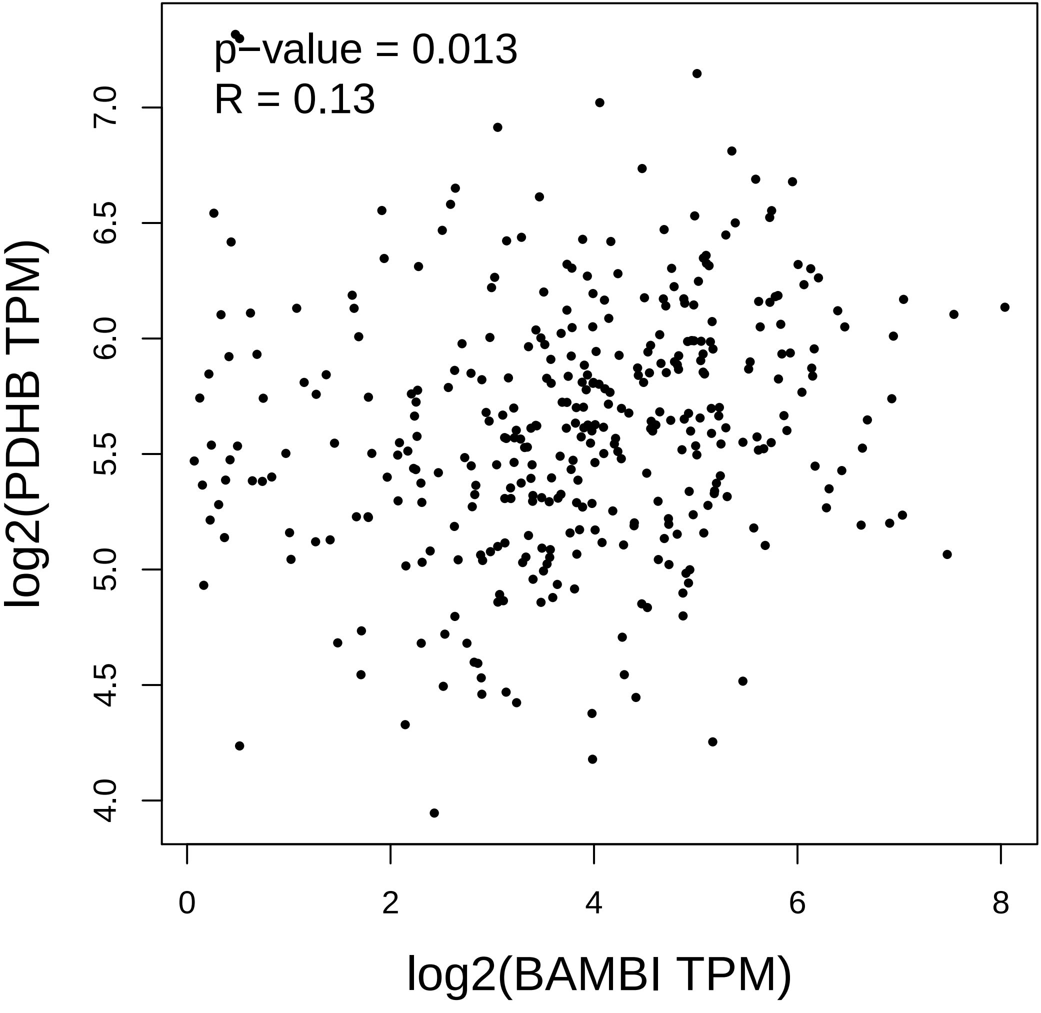
p−value = 0.013
R = 0.13
log2(PDHB TPM)
4.0
4.5
5.0
5.5
6.0
6.5
7.0
0
2
4
6
8
log2(BAMBI TPM)

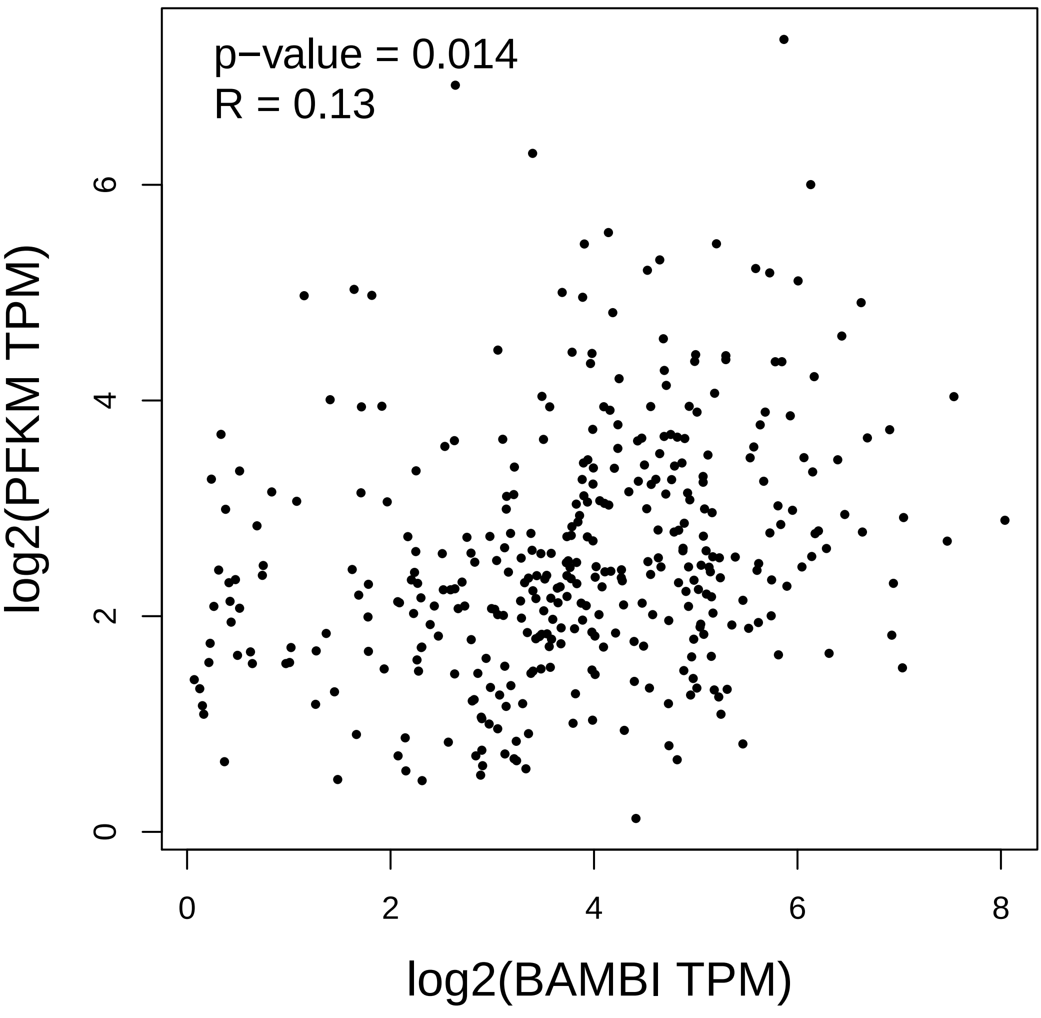
p−value = 0.014
R = 0.13
log2(PFKM TPM)
0
2
4
6
0
2
4
6
8
log2(BAMBI TPM)

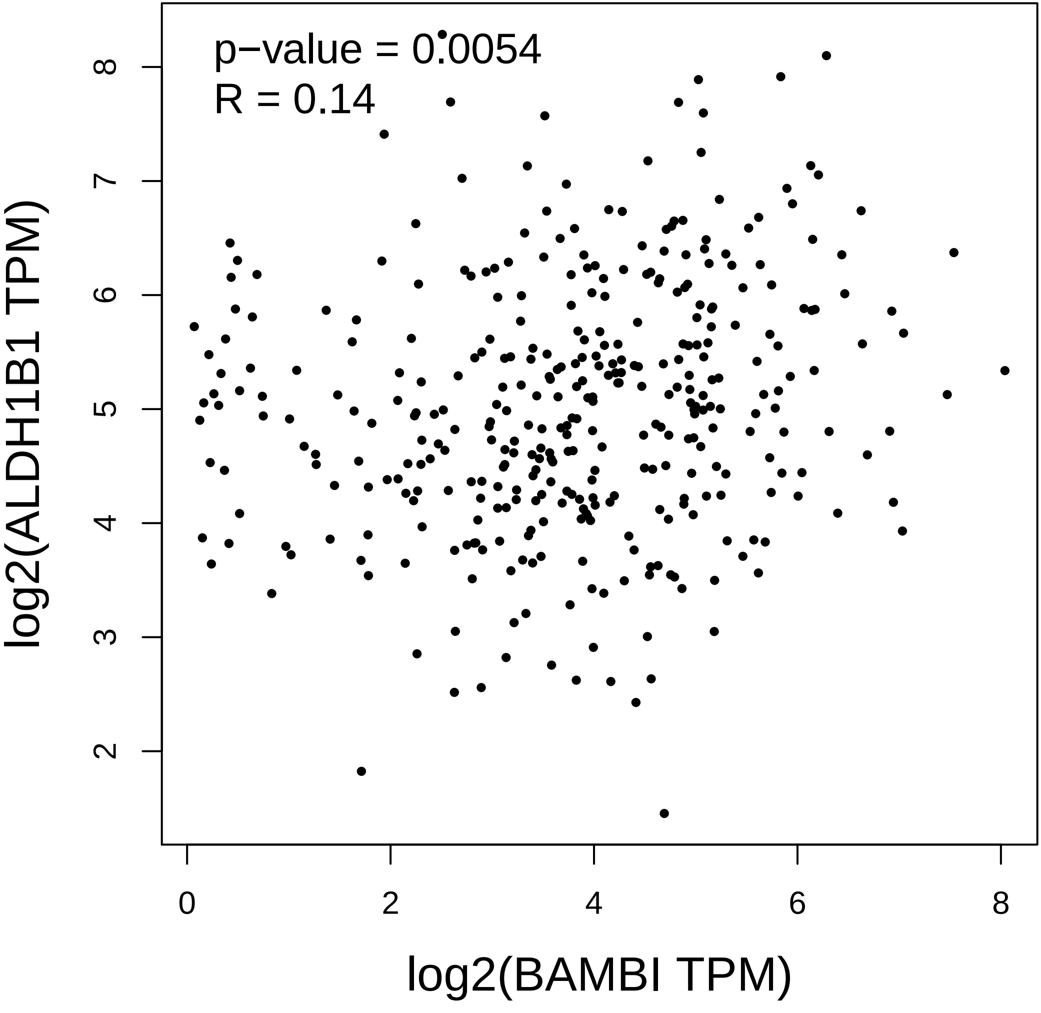

p−value = 0.0054
R = 0.14
log2(ALDH1B1 TPM)
2
3
4
5
6
7
8
0
2
4
6
8
log2(BAMBI TPM)

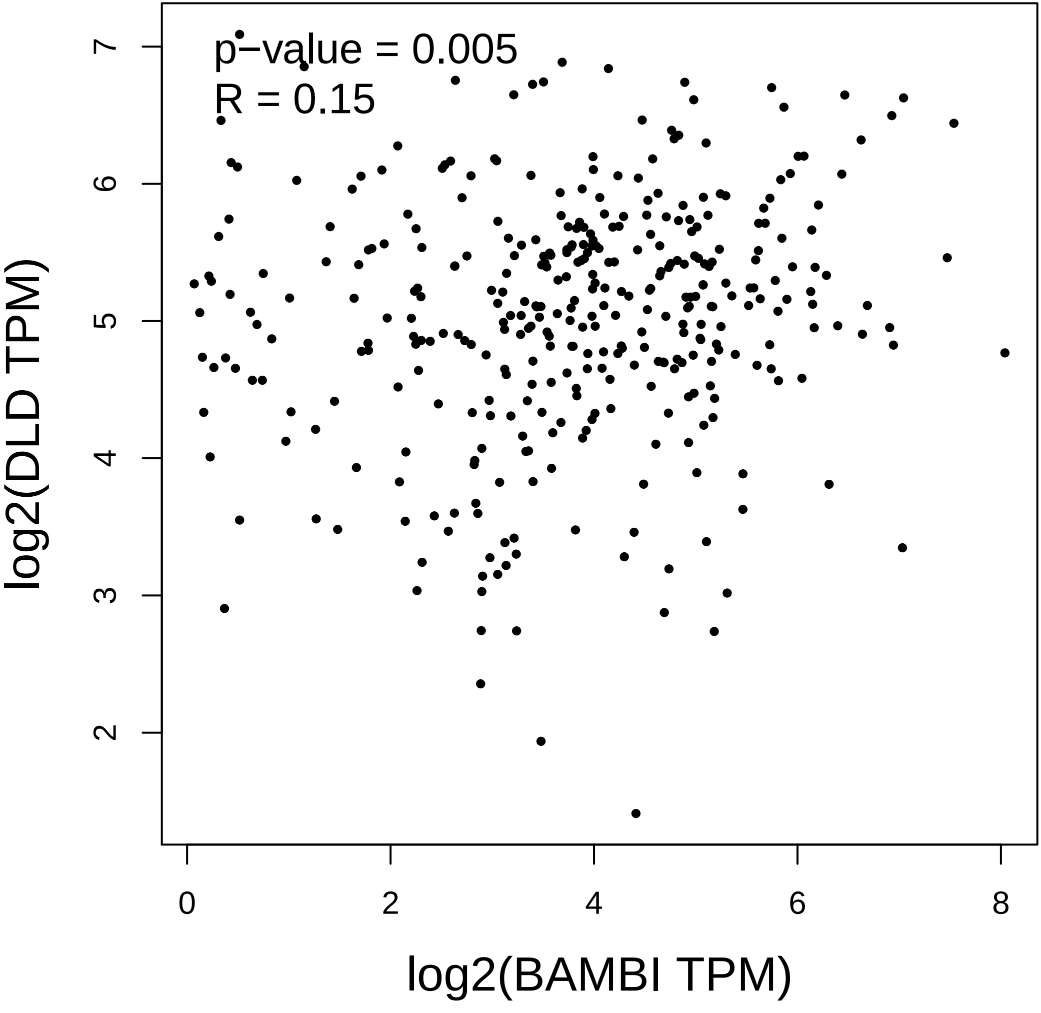
p−value = 0.005
R = 0.15
log2(DLD TPM)
2
3
4
5
6
7
0
2
4
6
8
log2(BAMBI TPM)

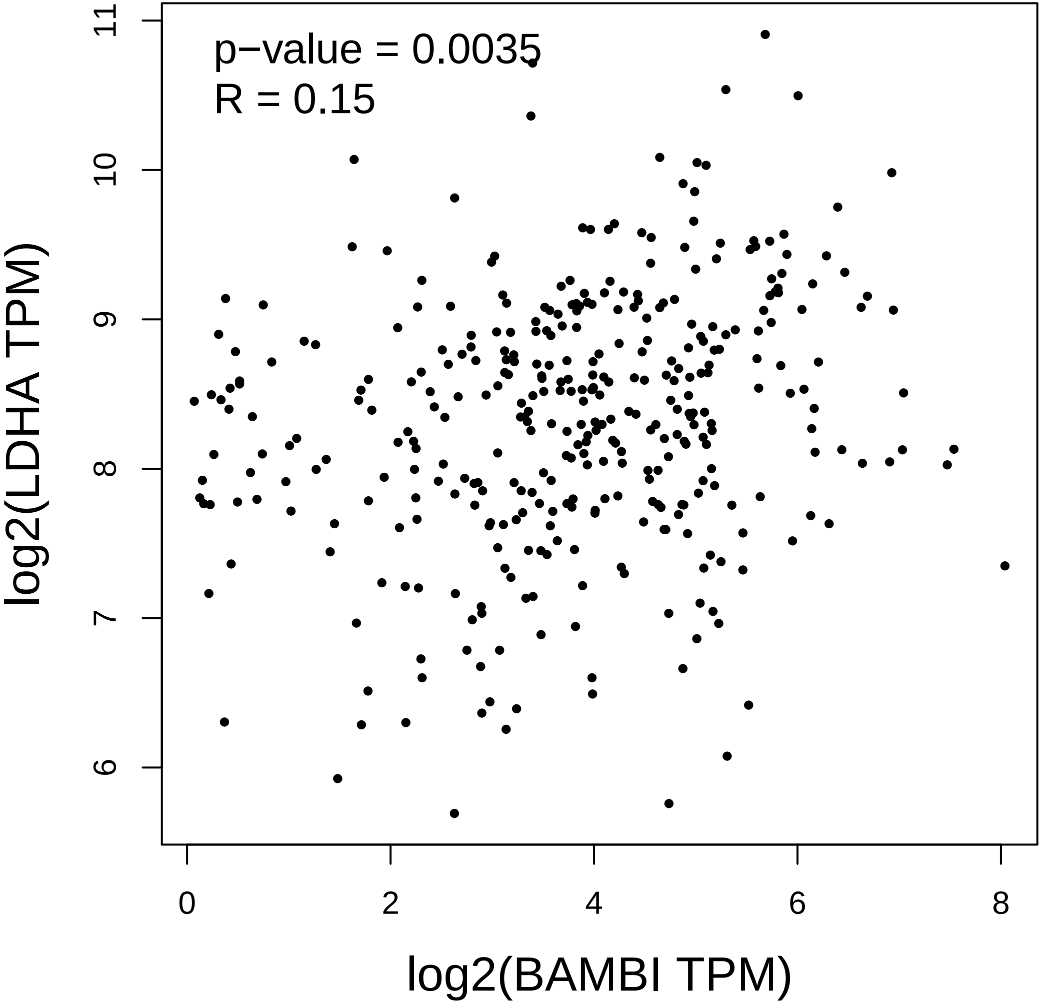

p−value = 0.0035
R = 0.15
log2(LDHA TPM)
log2(BAMBI TPM)
6
7
8
9
10
11
0
2
4
6
8

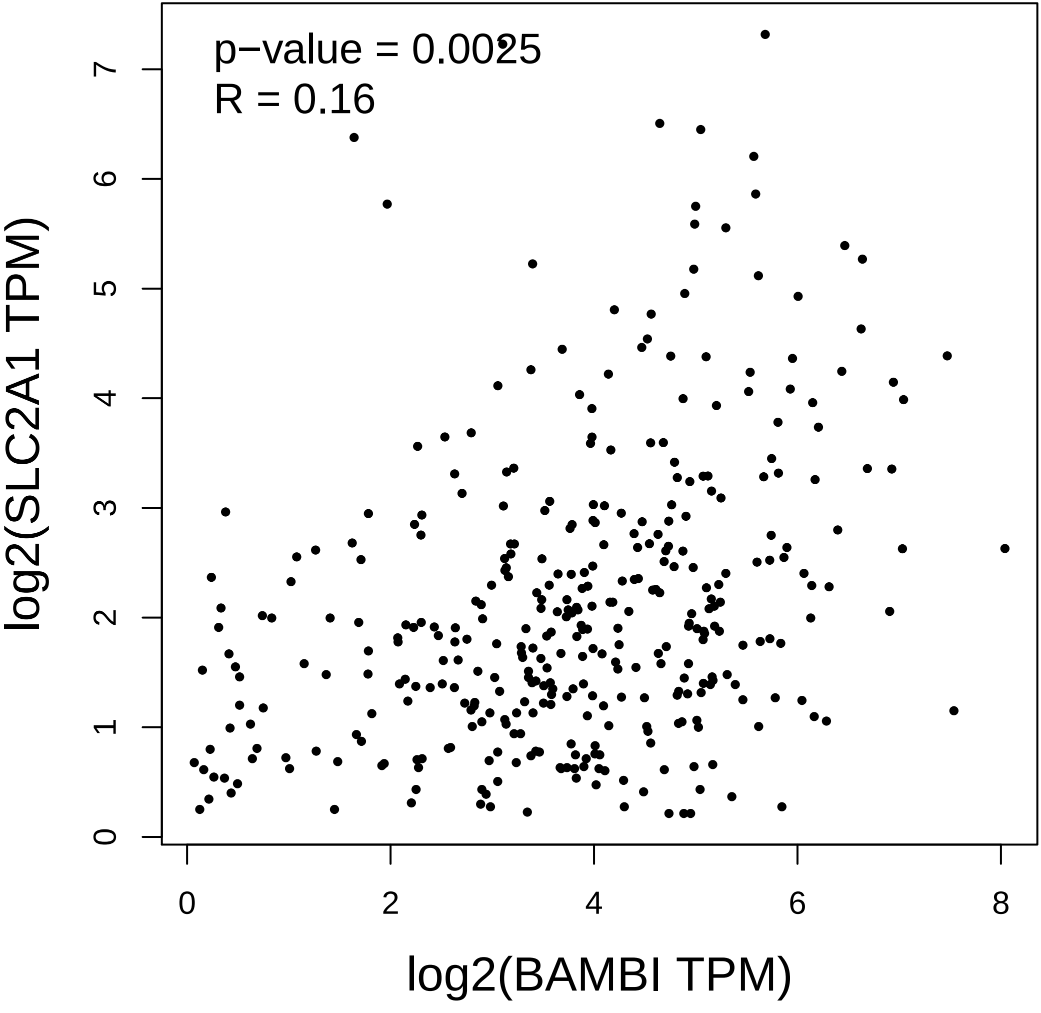
p−value = 0.0025
R = 0.16
log2(SLC2A1 TPM)
0
1
2
3
4
5
6
7
0
2
4
6
8
log2(BAMBI TPM)

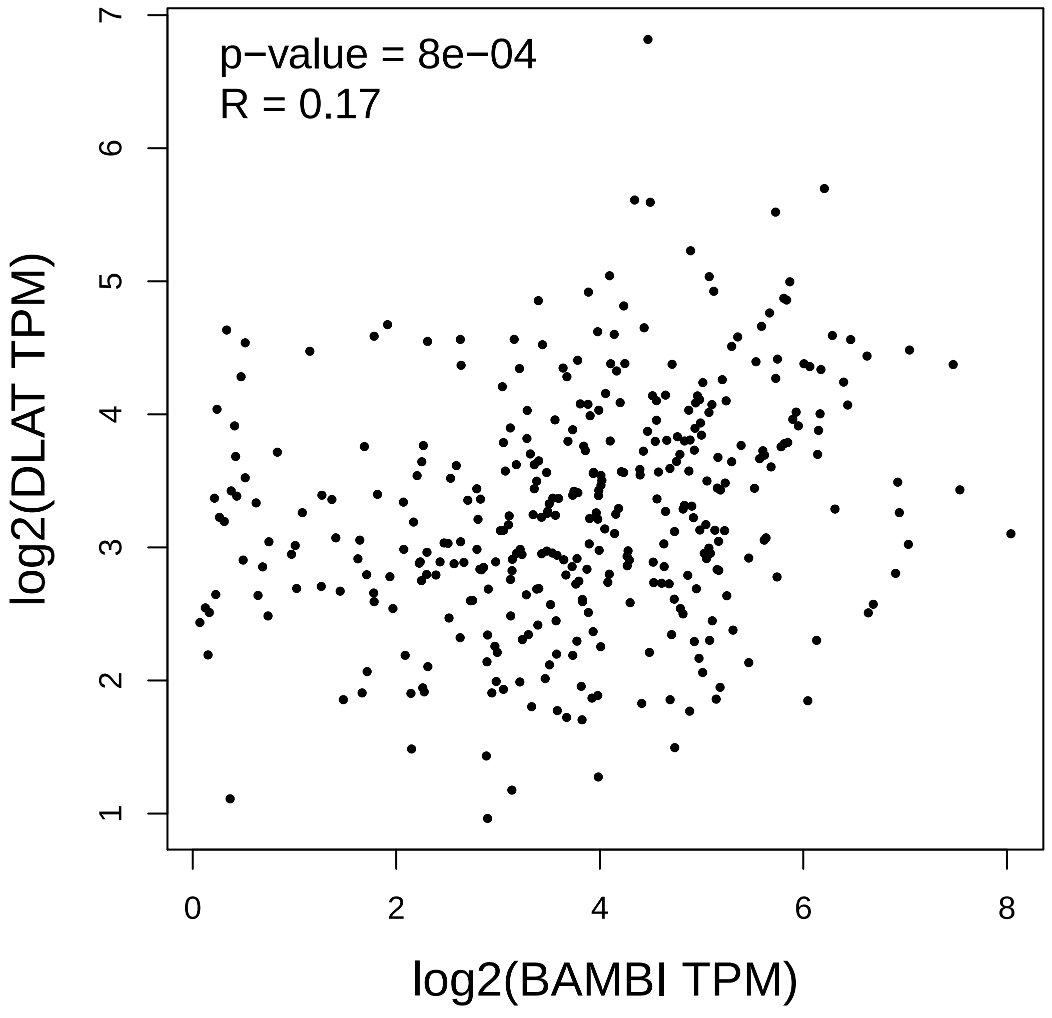
p−value = 8e−04
R = 0.17
log2(DLAT TPM)
log2(BAMBI TPM)
1
2
3
4
5
6
7
0
2
4
6
8

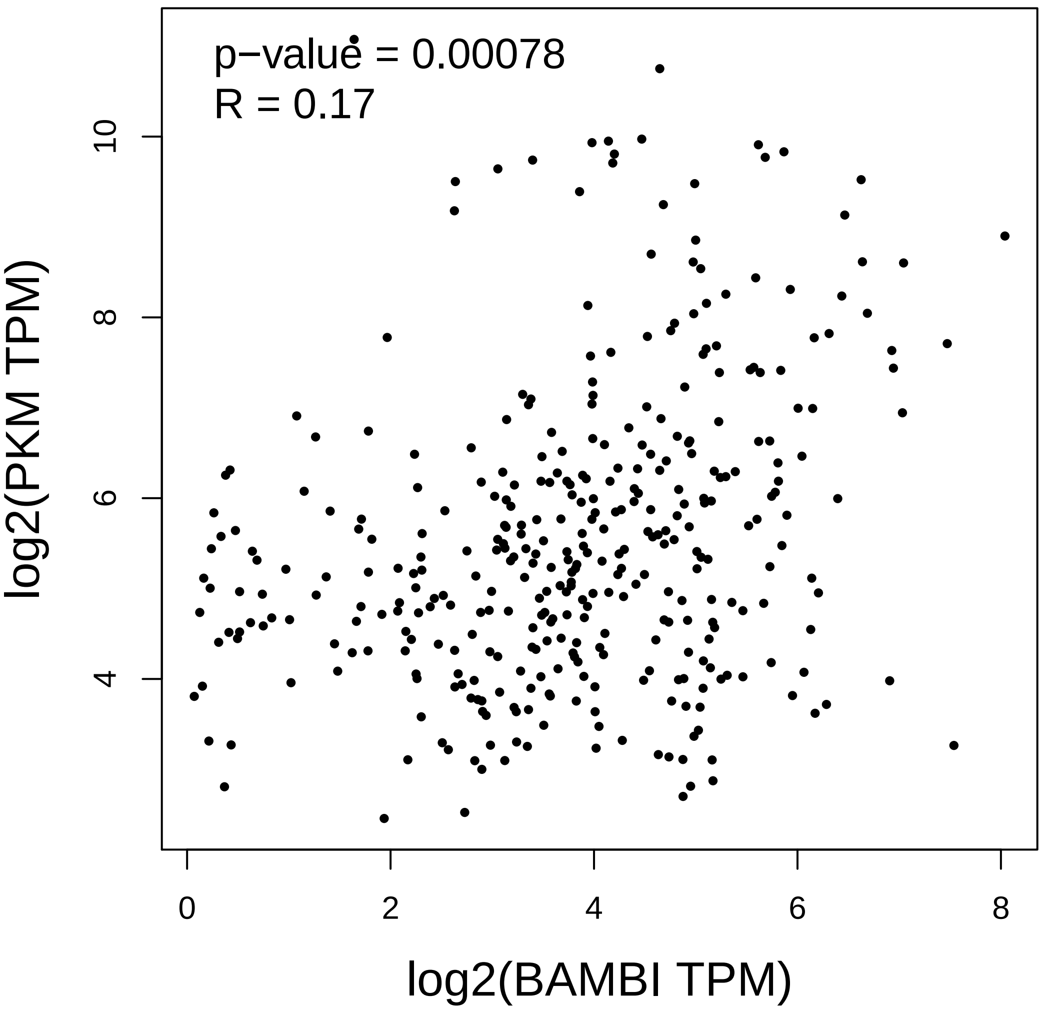
p−value = 0.00078
R = 0.17
log2(PKM TPM)
10
8
6
4
0
2
4
6
8
log2(BAMBI TPM)

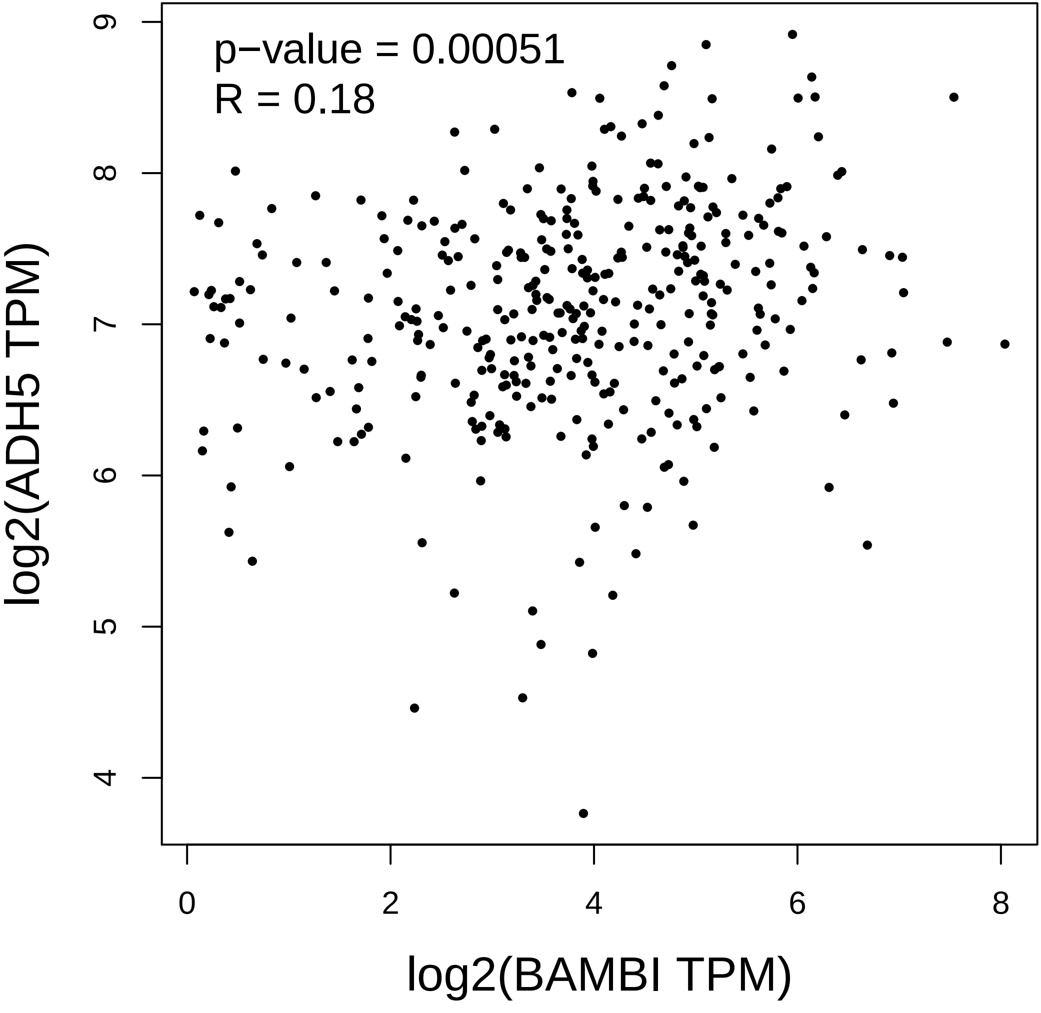
p−value = 0.00051
R = 0.18
log2(ADH5 TPM)
log2(BAMBI TPM)
4
5
6
7
8
9
0
2
4
6
8

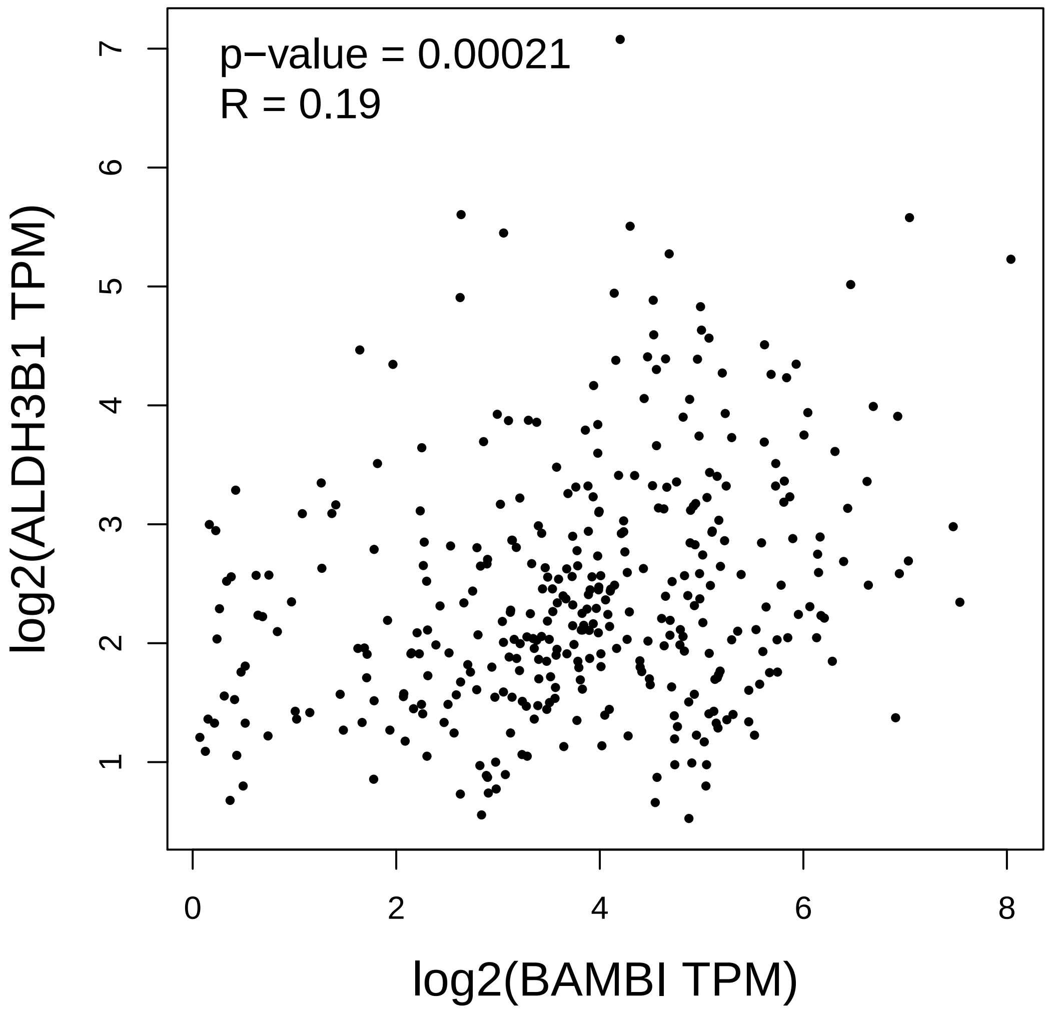

p−value = 0.00021
R = 0.19
log2(ALDH3B1 TPM)
log2(BAMBI TPM)
1
2
3
4
5
6
7
0
2
4
6
8

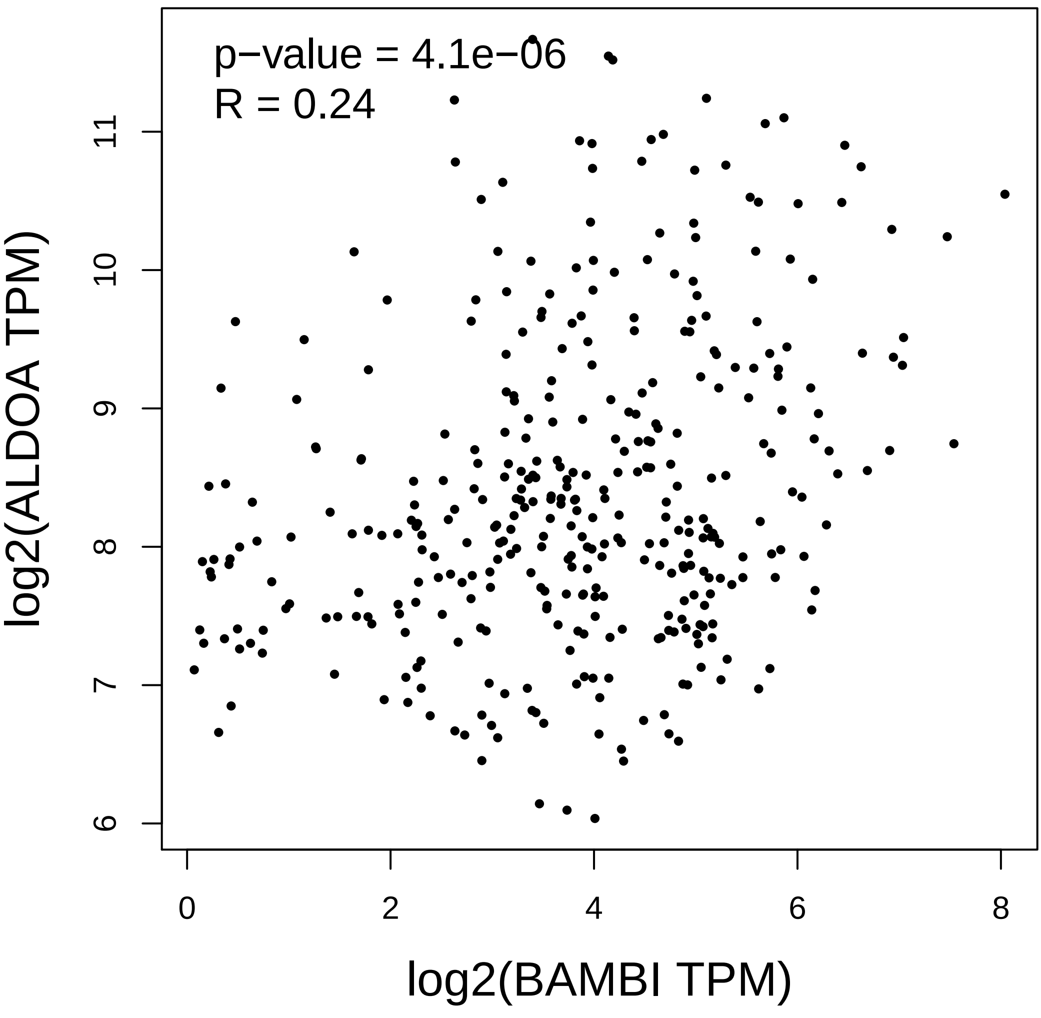
p−value = 4.1e−06
R = 0.24
log2(ALDOA TPM)
log2(BAMBI TPM)
6
7
8
9
10
11
0
2
4
6
8

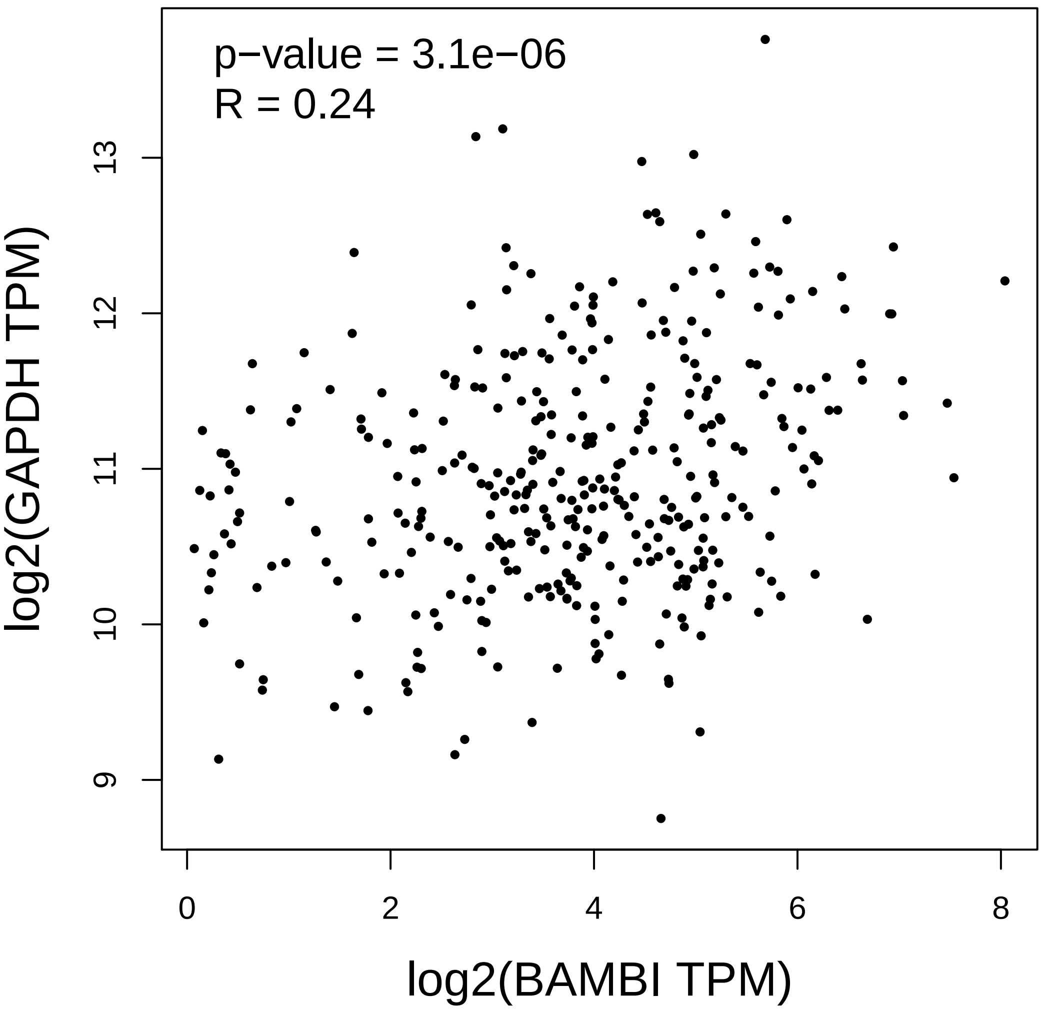

p−value = 3.1e−06
R = 0.24
log2(GAPDH TPM)
9
10
11
12
13
0
2
4
6
8
log2(BAMBI TPM)

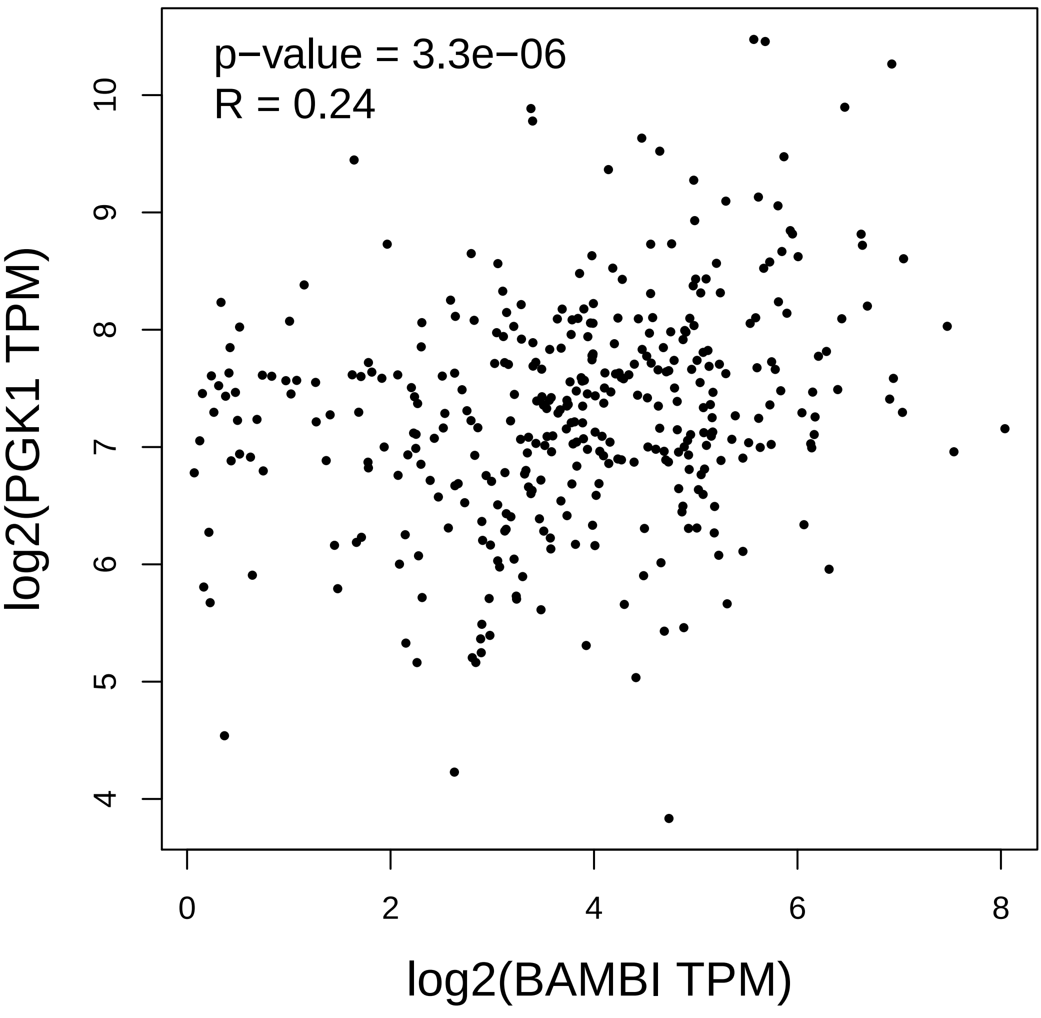

p−value = 3.3e−06
R = 0.24
log2(PGK1 TPM)
4
5
6
7
8
9
10
0
2
4
6
8
log2(BAMBI TPM)

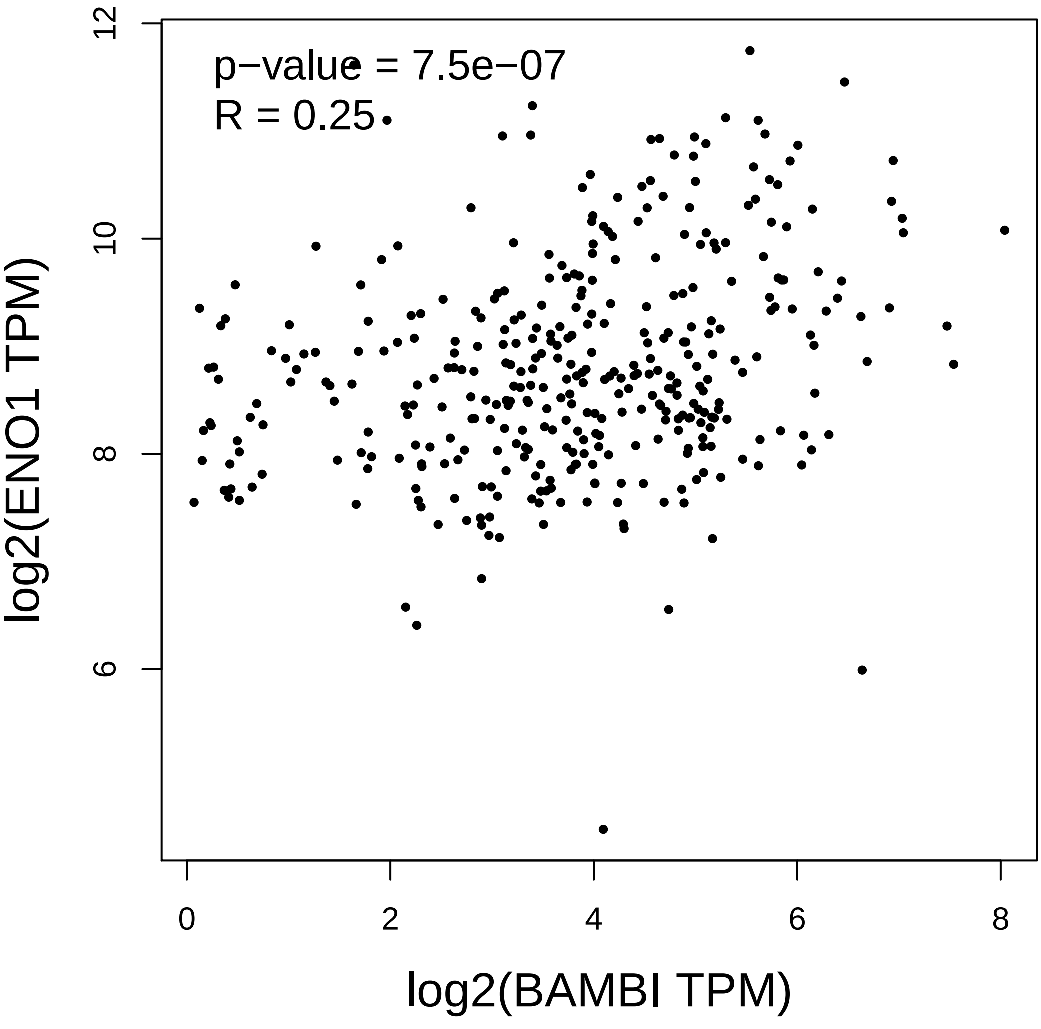
p−value = 7.5e−07
R = 0.25
log2(ENO1 TPM)
log2(BAMBI TPM)
12
10
8
6
0
2
4
6
8

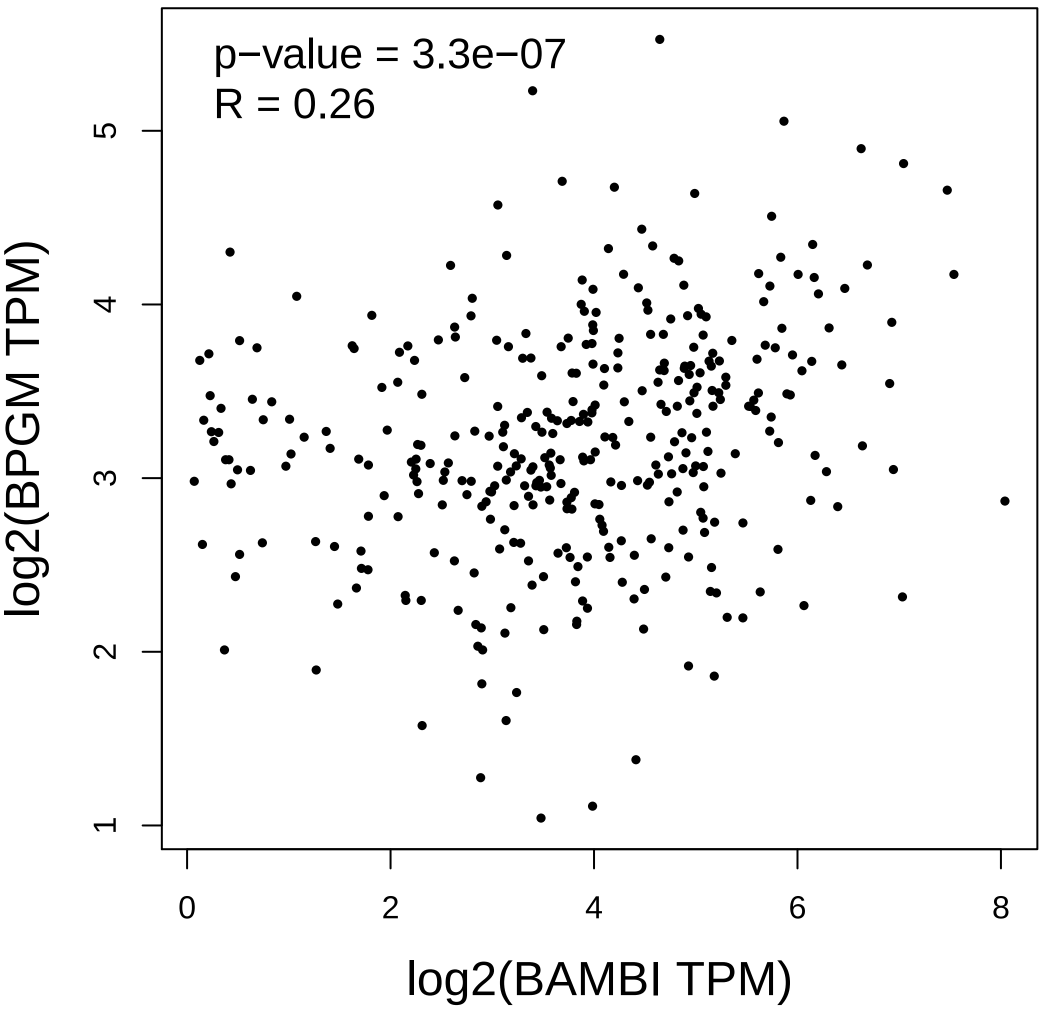
p−value = 3.3e−07
R = 0.26
log2(BPGM TPM)
log2(BAMBI TPM)
1
2
3
4
5
0
2
4
6
8

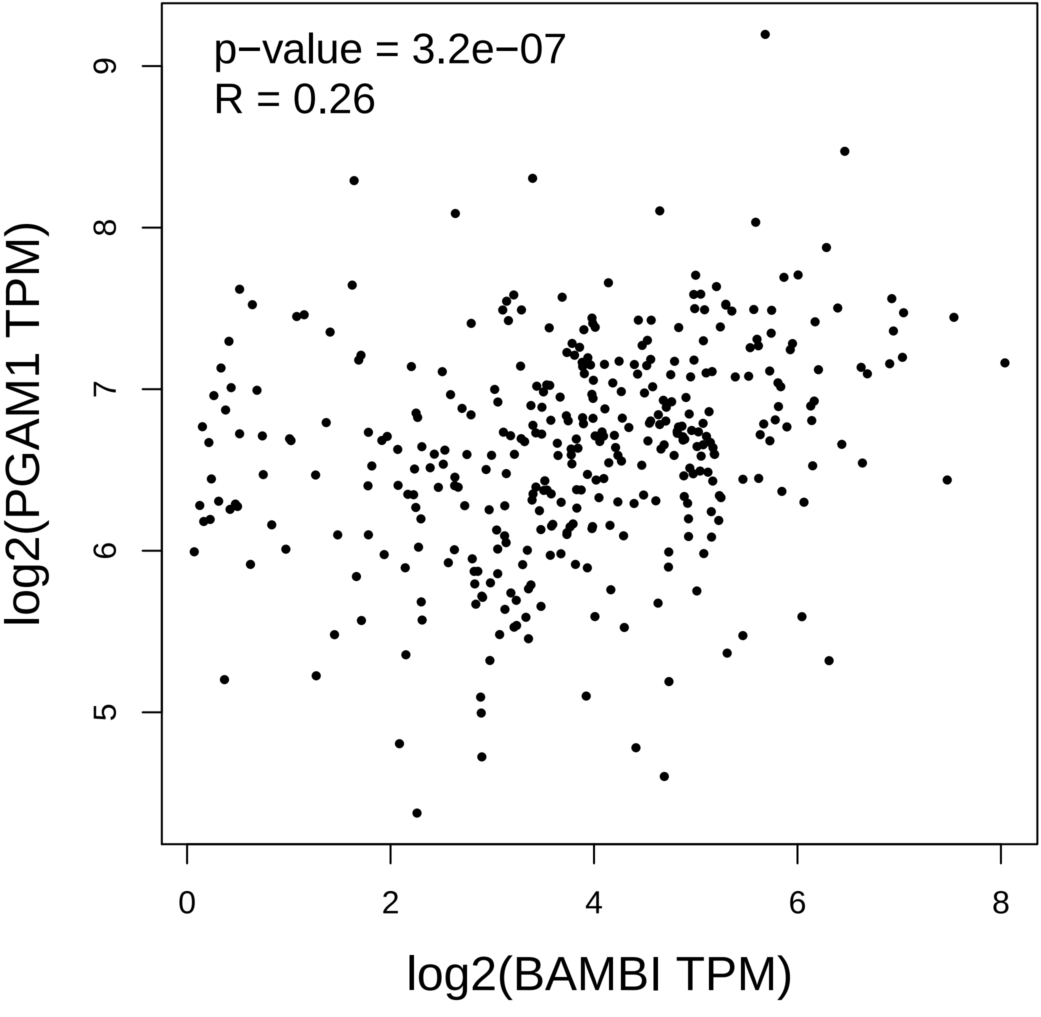

p−value = 3.2e−07
R = 0.26
log2(PGAM1 TPM)
log2(BAMBI TPM)
5
6
7
8
9
0
2
4
6
8

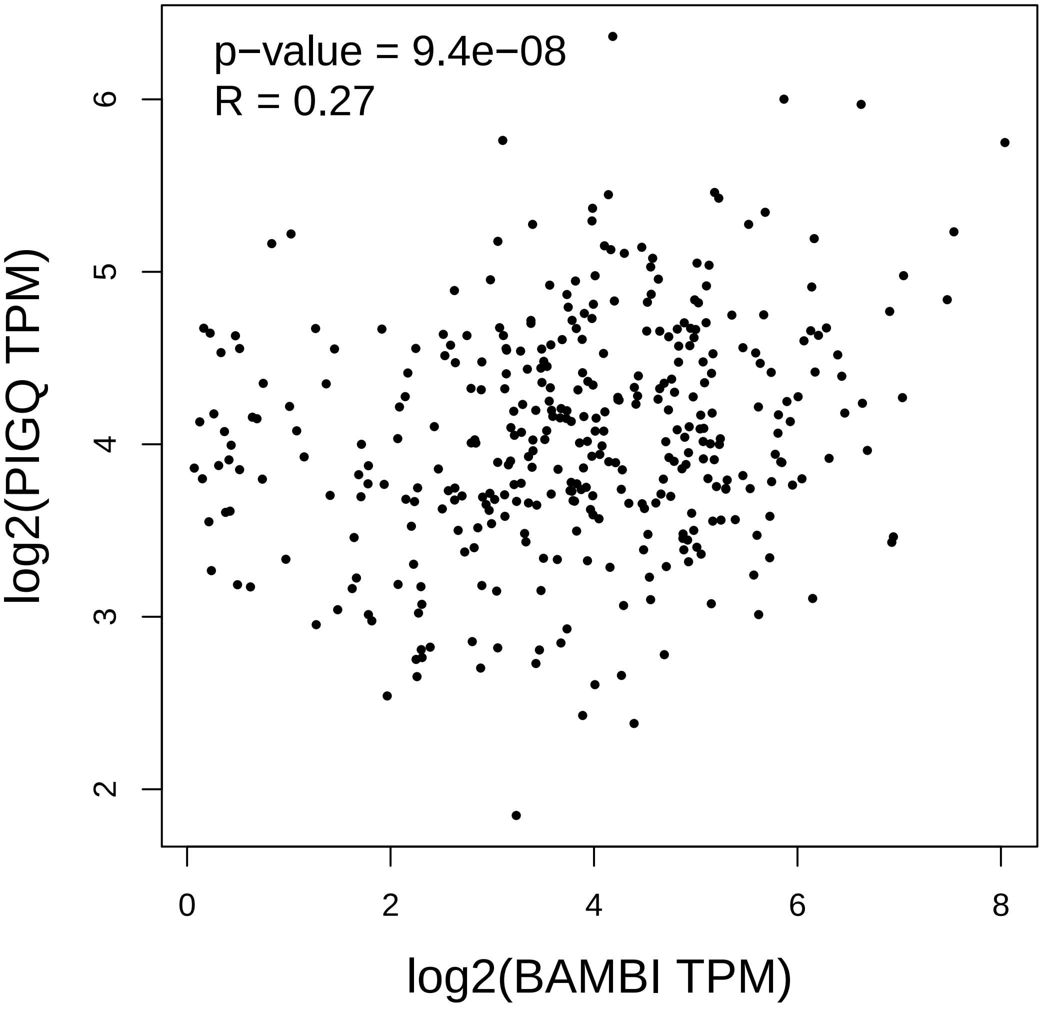
p−value = 9.4e−08
R = 0.27
log2(PIGQ TPM)
log2(BAMBI TPM)
2
3
4
5
6
0
2
4
6
8

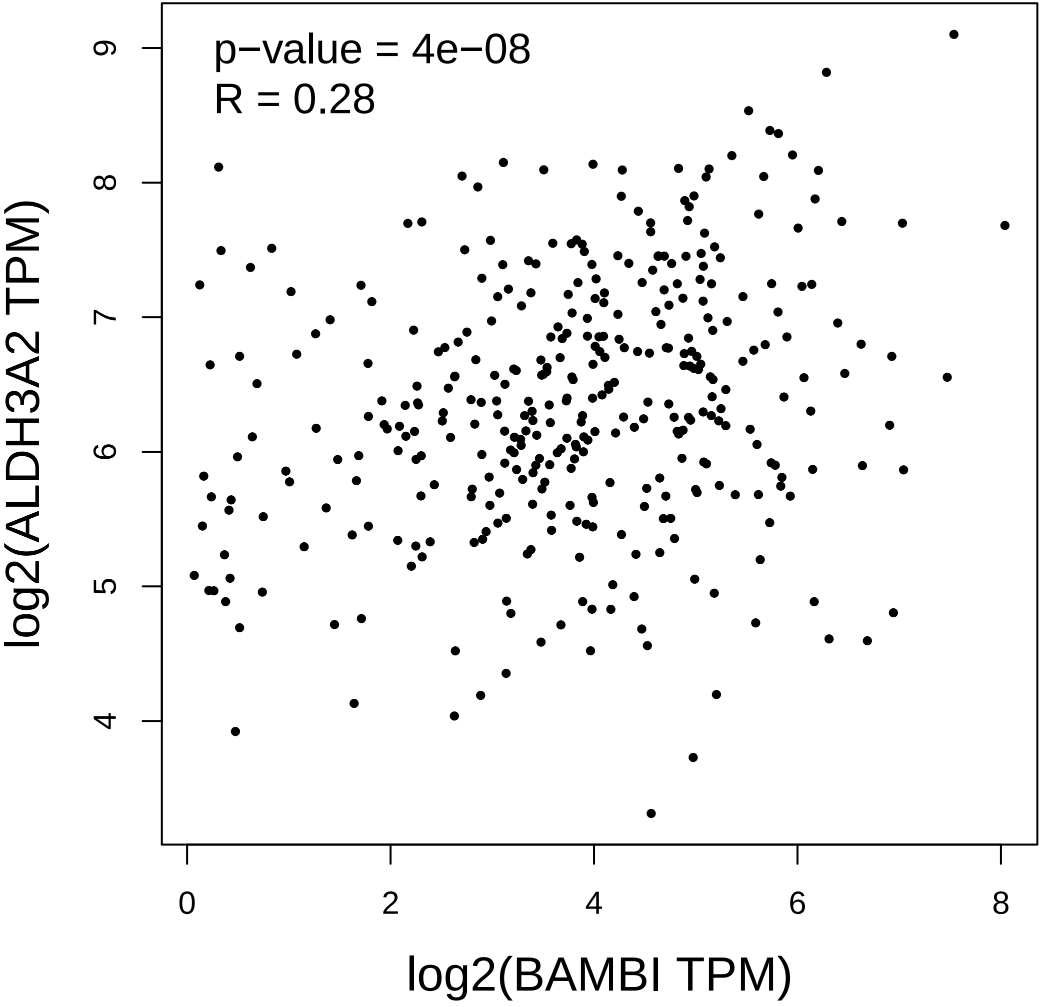

p−value = 4e−08
R = 0.28
log2(ALDH3A2 TPM)
log2(BAMBI TPM)
4
5
6
7
8
9
0
2
4
6
8

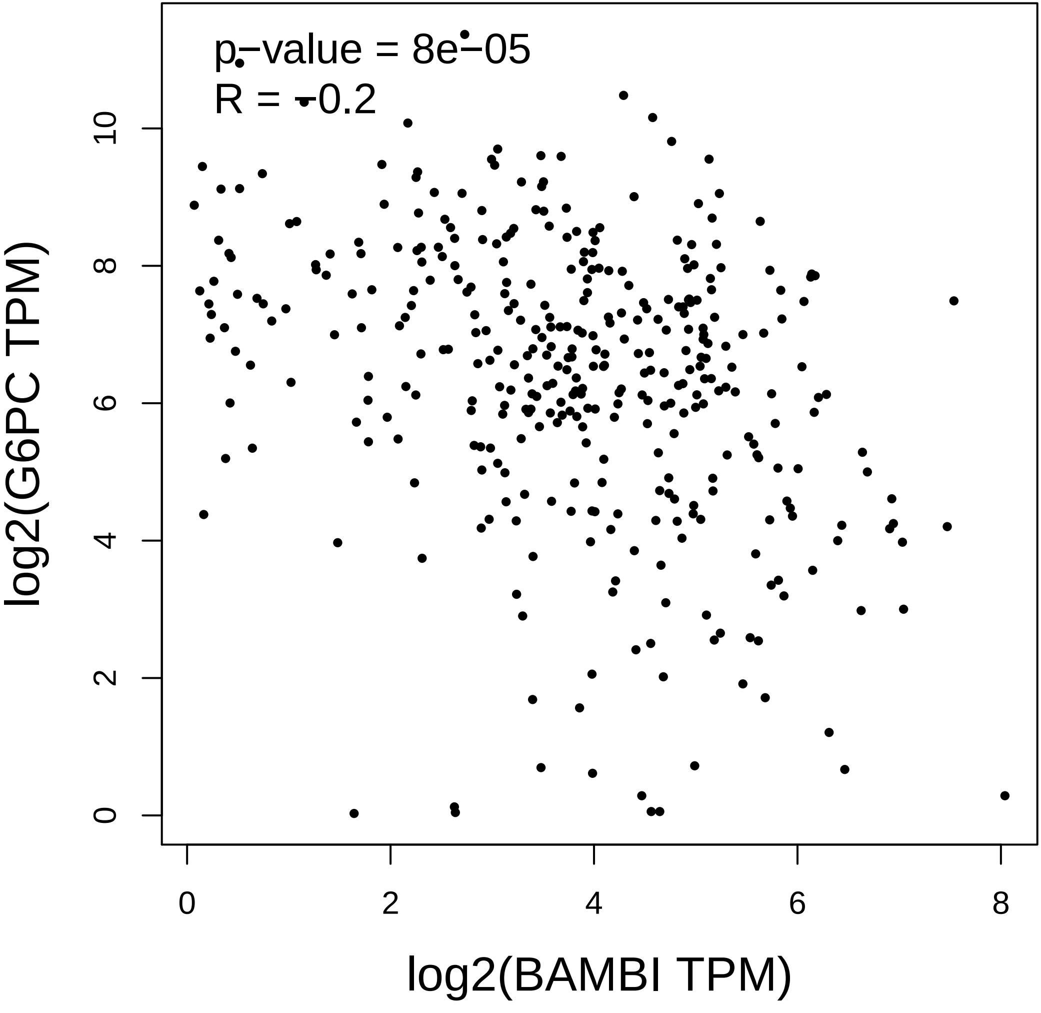

p−value = 8e−05
R = −0.2
log2(G6PC TPM)
0
2
4
6
8
10
log2(BAMBI TPM)
0
2
4
6
8

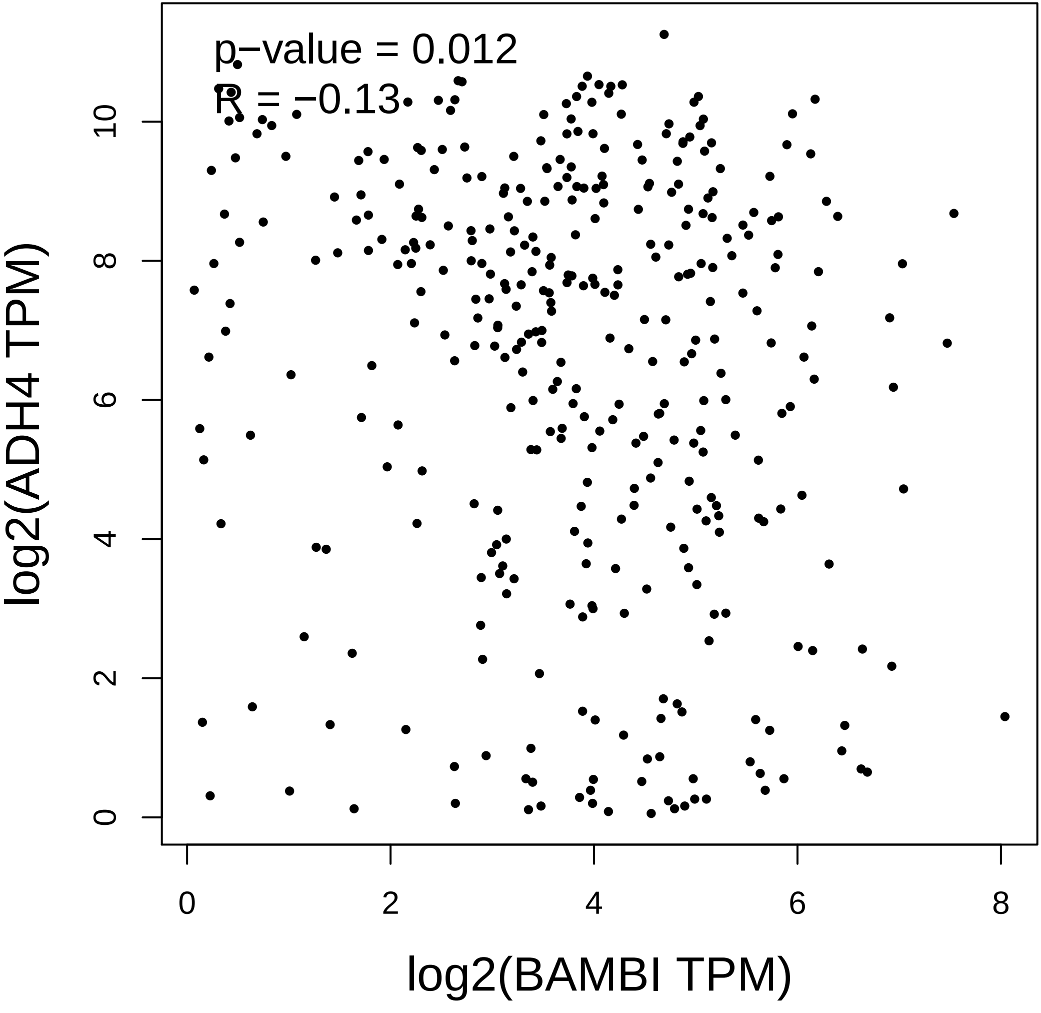

p−value = 0.012
R = −0.13
log2(ADH4 TPM)
0
2
4
6
8
10
0
2
4
6
8
log2(BAMBI TPM)

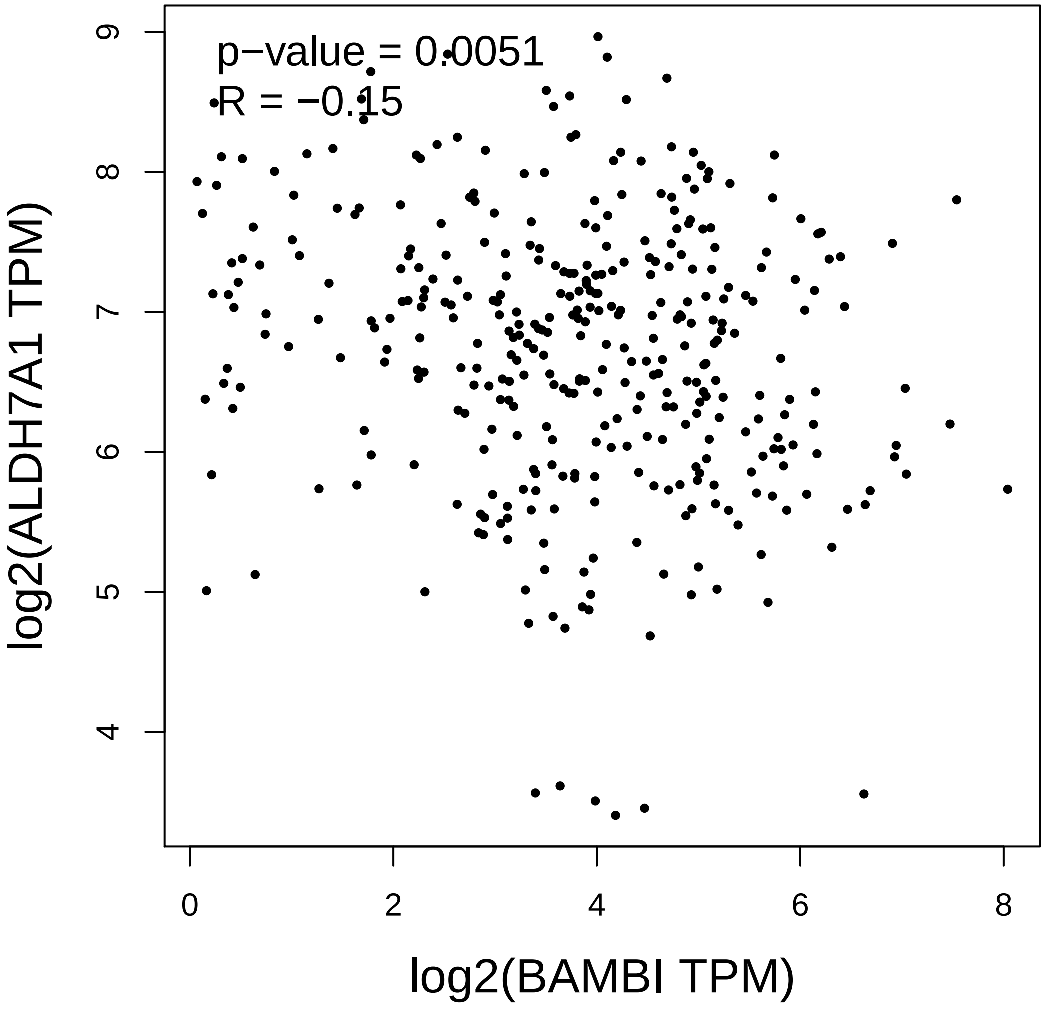
p−value = 0.0051
R = −0.15
log2(ALDH7A1 TPM)
log2(BAMBI TPM)
9
8
7
6
5
4
0
2
4
6
8

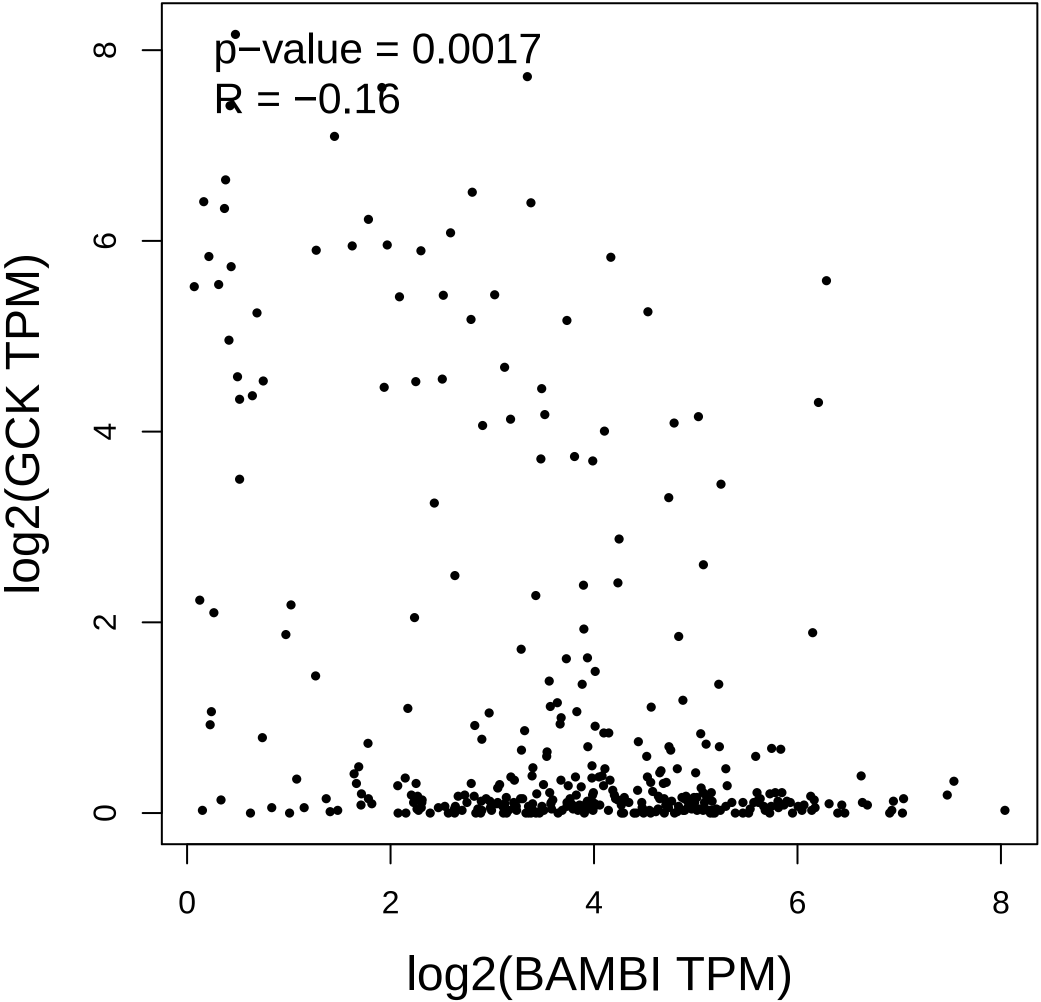

p−value = 0.0017
R = −0.16
log2(GCK TPM)
log2(BAMBI TPM)
0
2
4
6
8
0
2
4
6
8

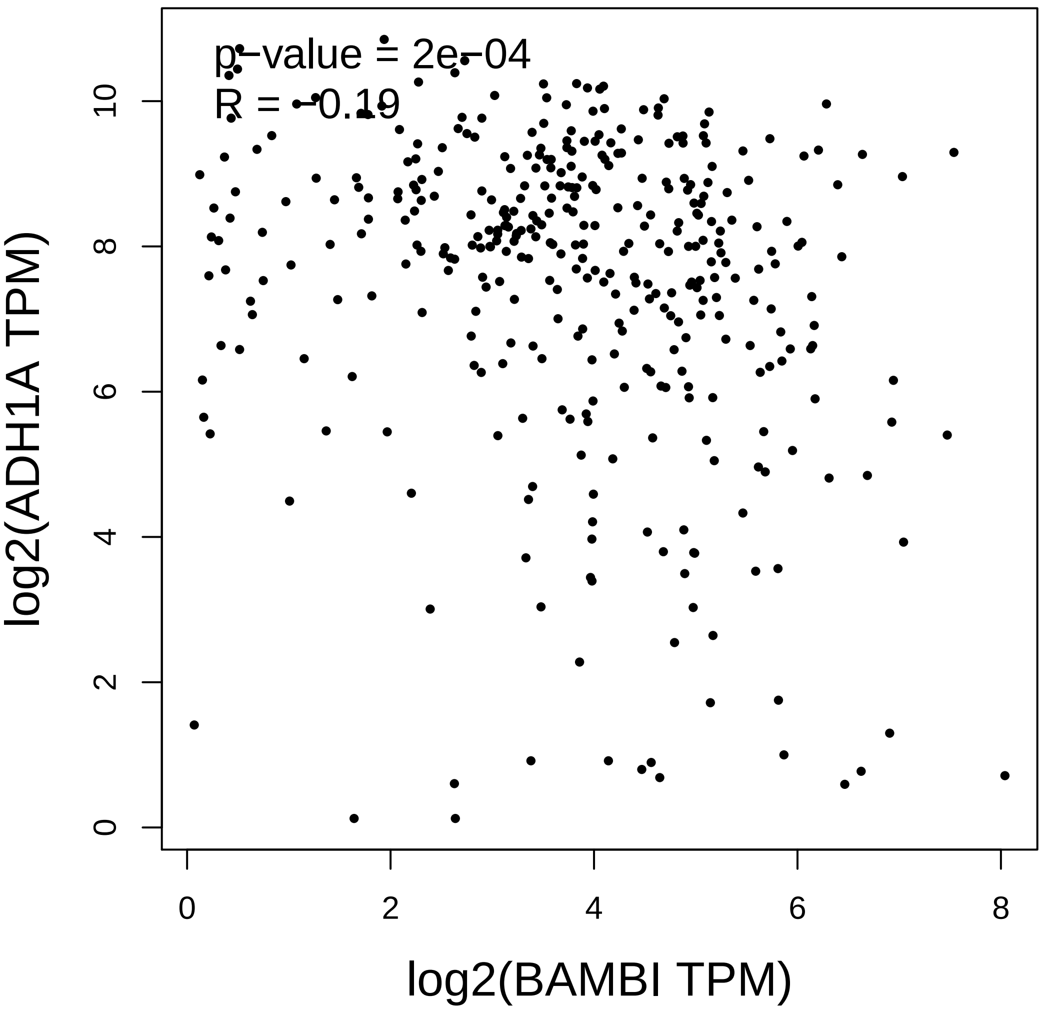

p−value = 2e−04
R = −0.19
log2(ADH1A TPM)
0
2
4
6
8
10
0
2
4
6
8
log2(BAMBI TPM)

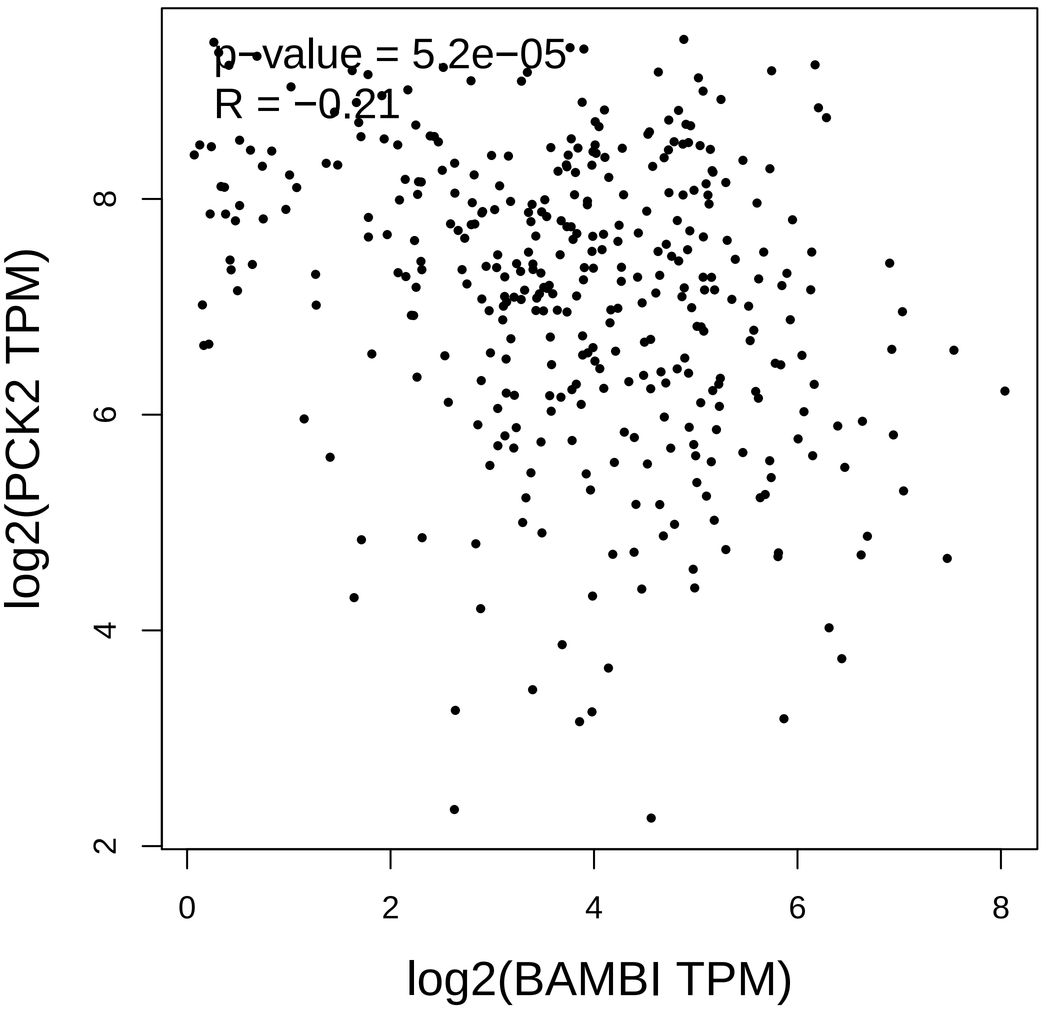
p−value = 5.2e−05
R = −0.21
log2(PCK2 TPM)
log2(BAMBI TPM)
2
4
6
8
0
2
4
6
8

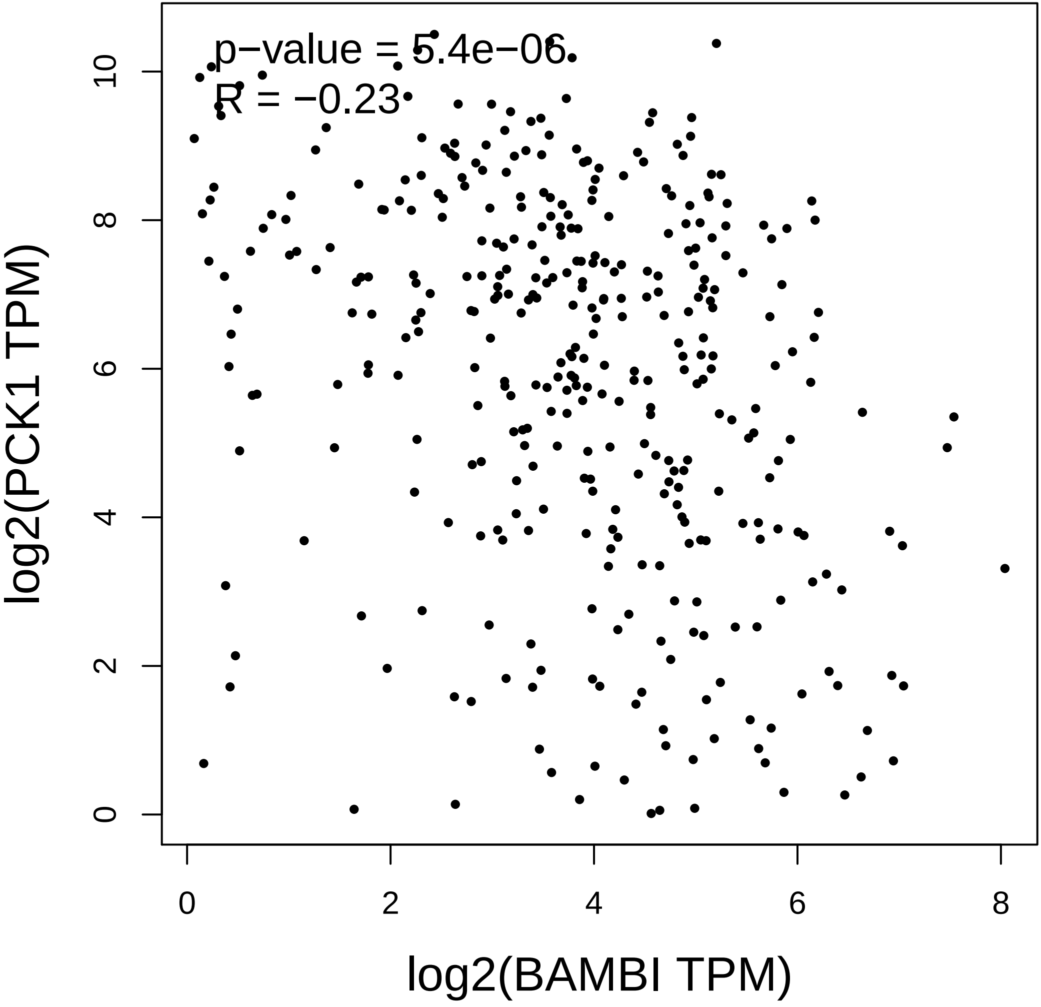

p−value = 5.4e−06
R = −0.23
log2(PCK1 TPM)
0
2
4
6
8
10
log2(BAMBI TPM)
0
2
4
6
8

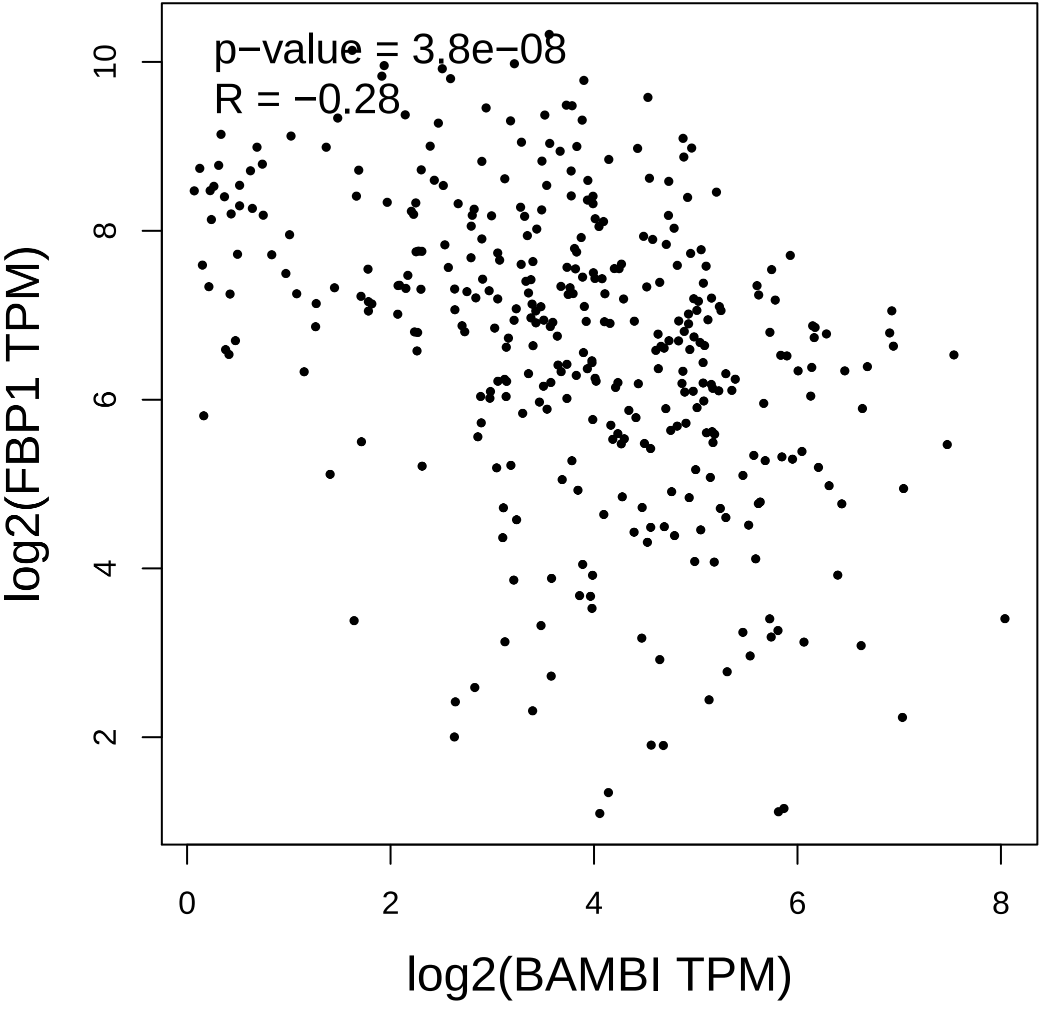
p−value = 3.8e−08
R = −0.28
log2(FBP1 TPM)
log2(BAMBI TPM)
2
4
6
8
10
0
2
4
6
8

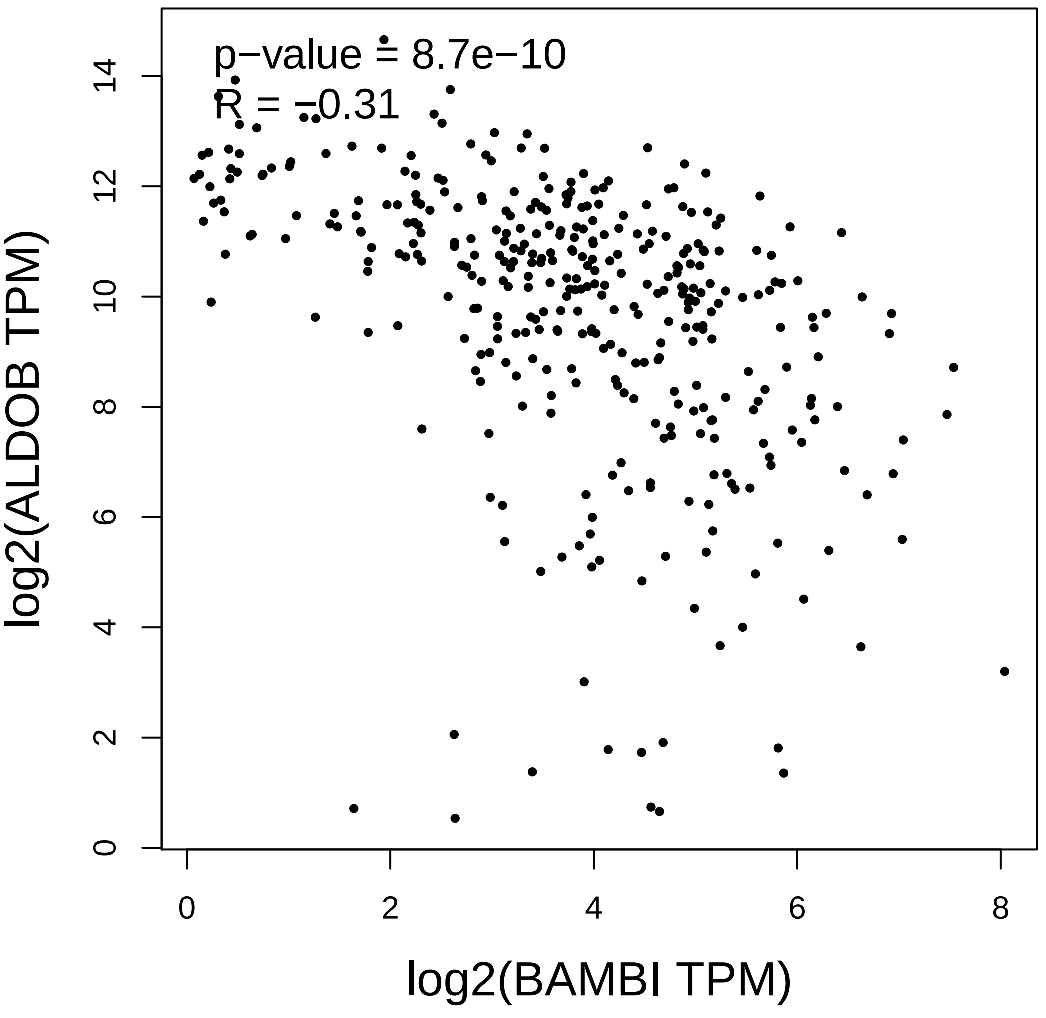
p−value = 8.7e−10
R = −0.31
log2(ALDOB TPM)
log2(BAMBI TPM)
0
2
4
6
8
10
12
14
0
2
4
6
8

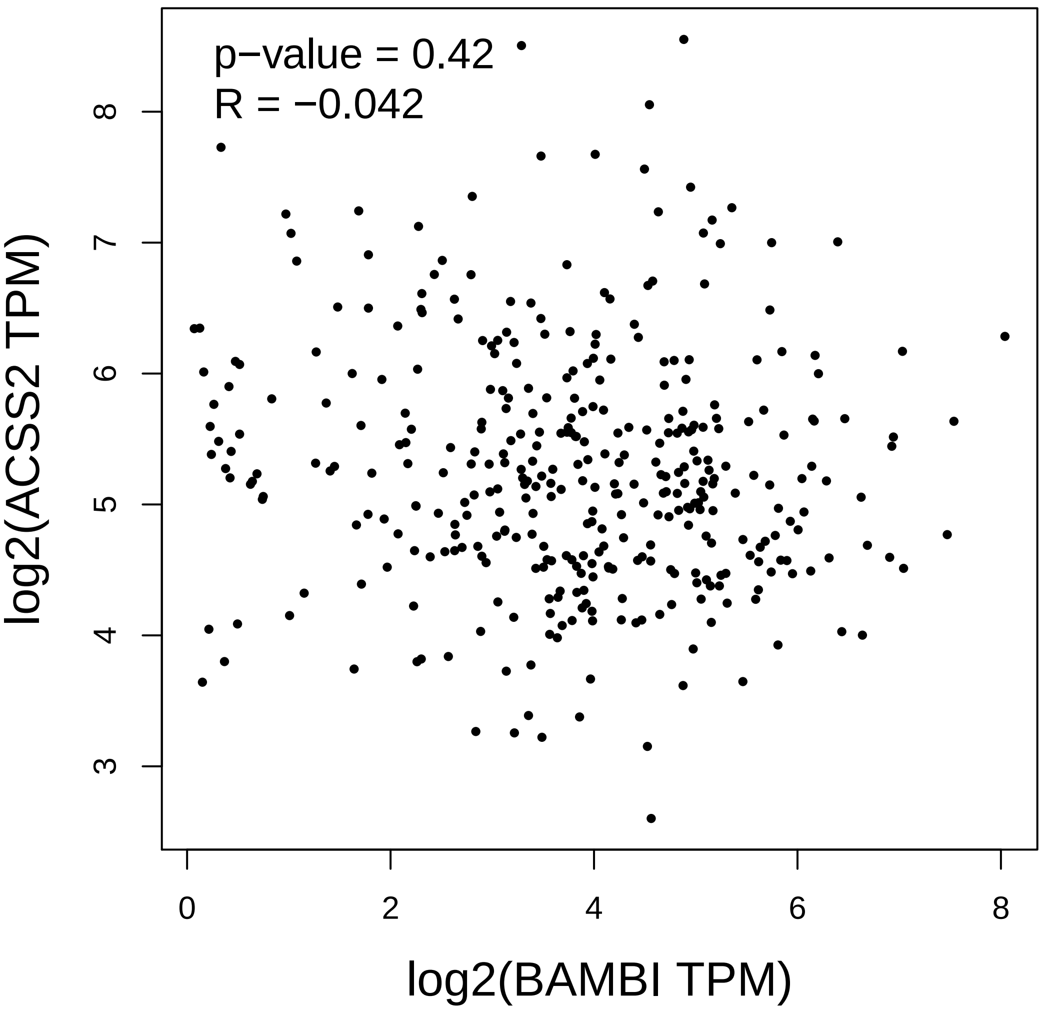
p−value = 0.42
R = −0.042
log2(ACSS2 TPM)
3
4
5
6
7
8
0
2
4
6
8
log2(BAMBI TPM)

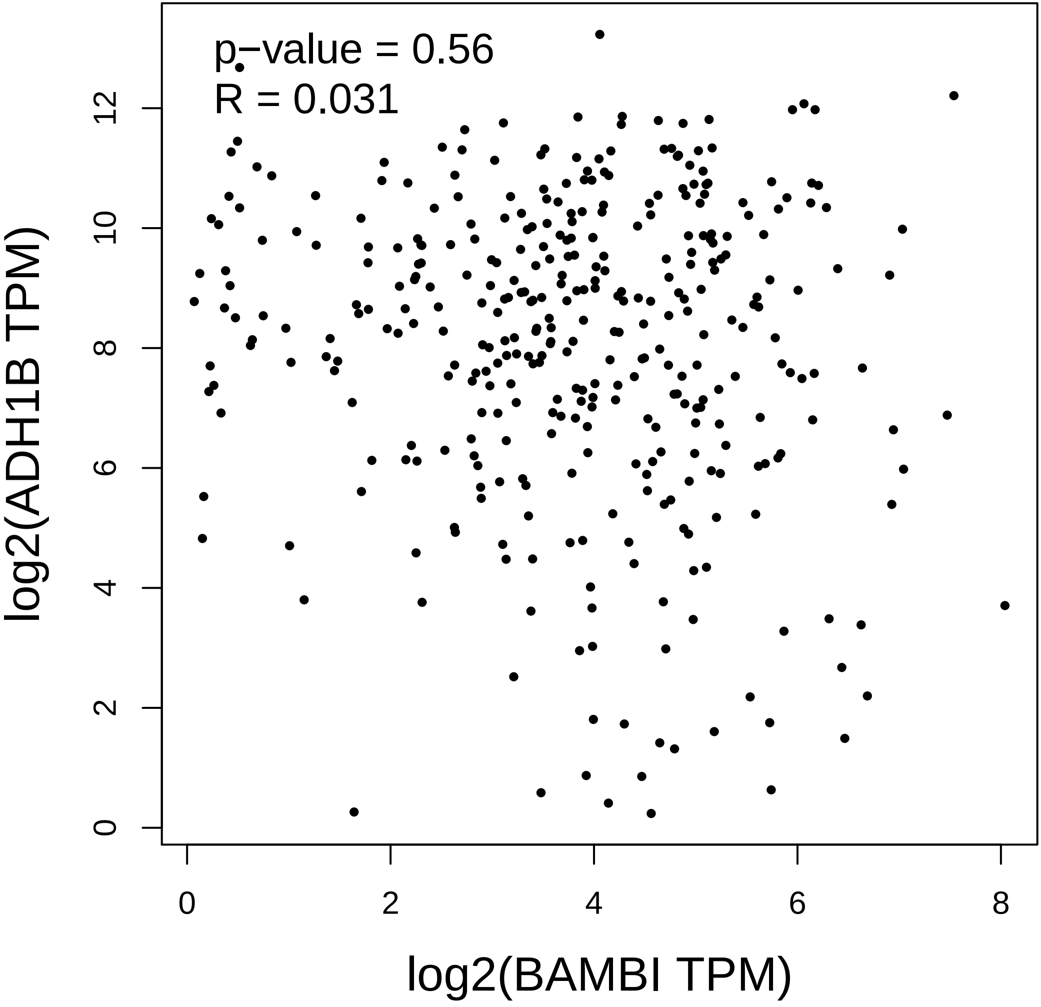

p−value = 0.56
R = 0.031
log2(ADH1B TPM)
log2(BAMBI TPM)
0
2
4
6
8
10
12
0
2
4
6
8

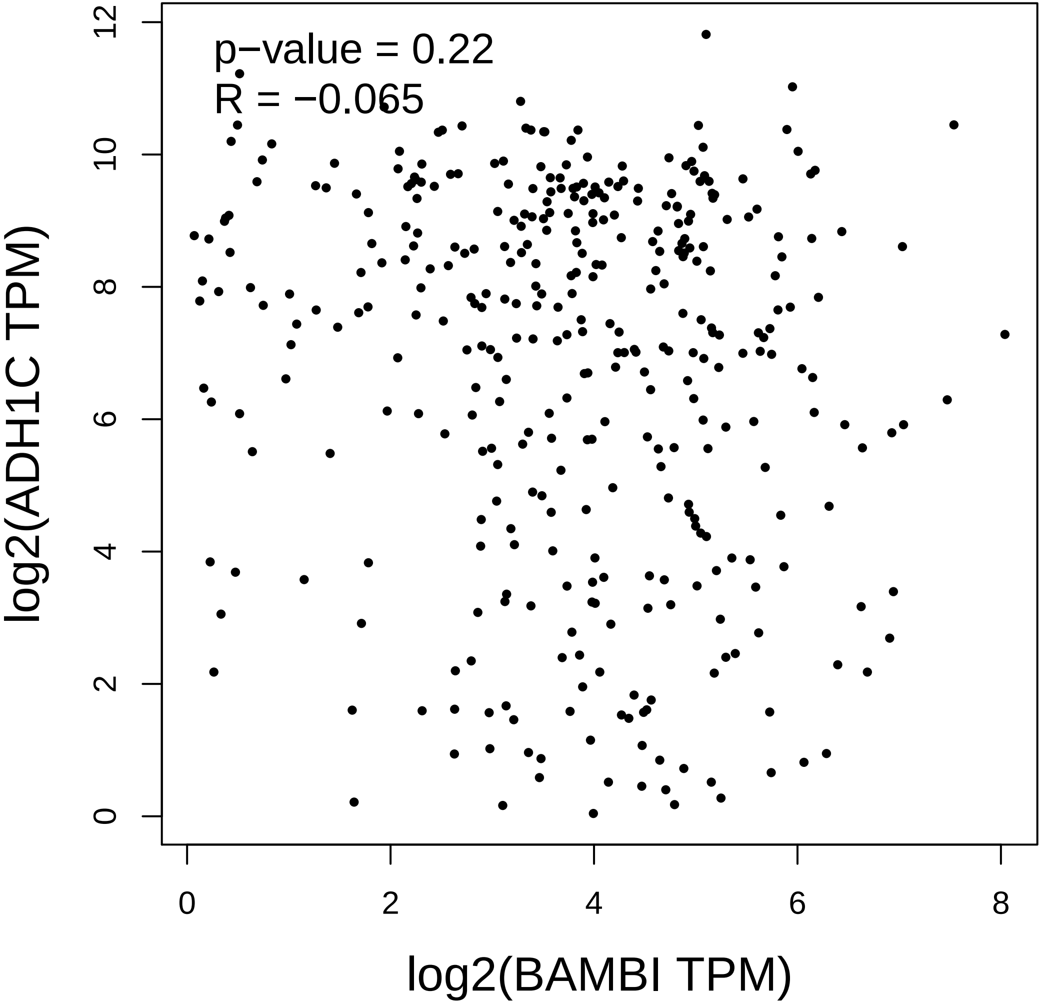
p−value = 0.22
R = −0.065
log2(ADH1C TPM)
log2(BAMBI TPM)
0
2
4
6
8
10
12
0
2
4
6
8

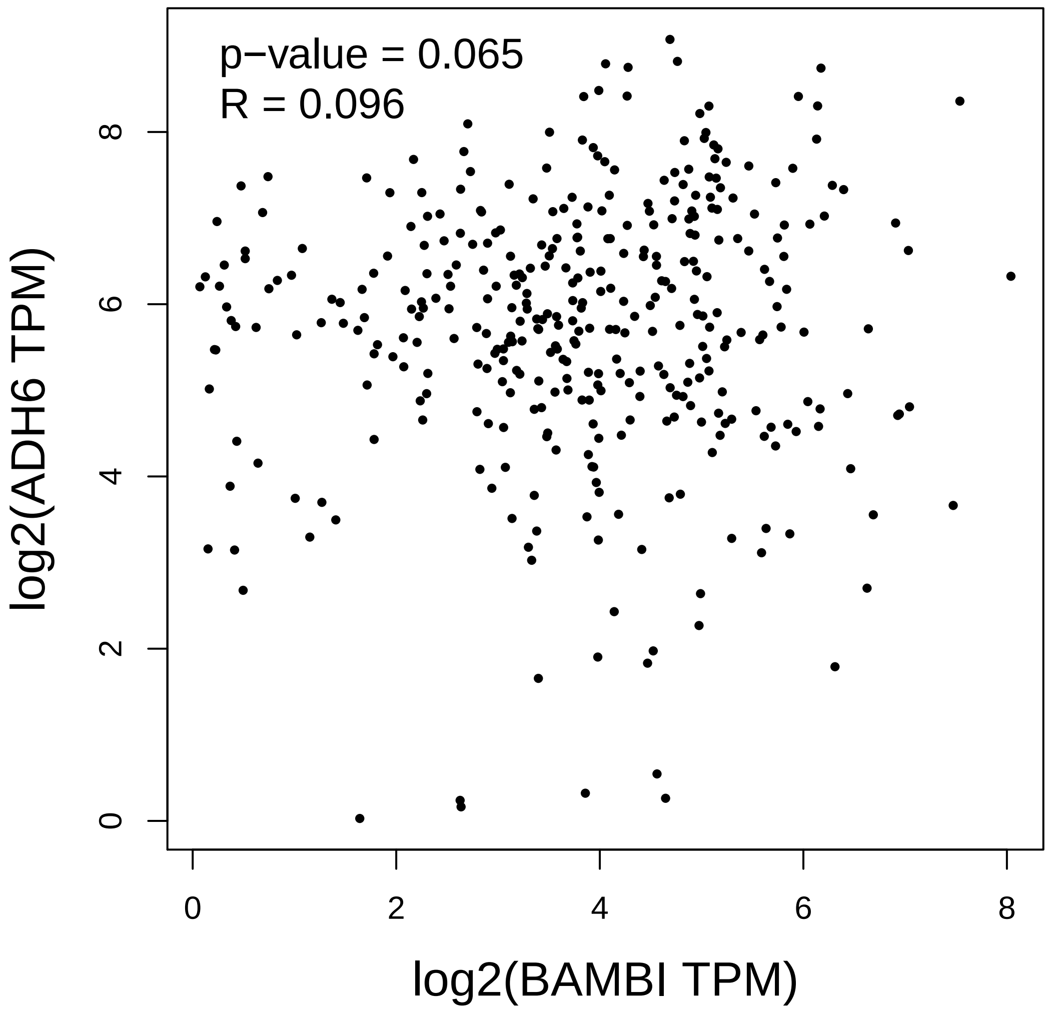
p−value = 0.065
R = 0.096
log2(ADH6 TPM)
log2(BAMBI TPM)
0
2
4
6
8
0
2
4
6
8

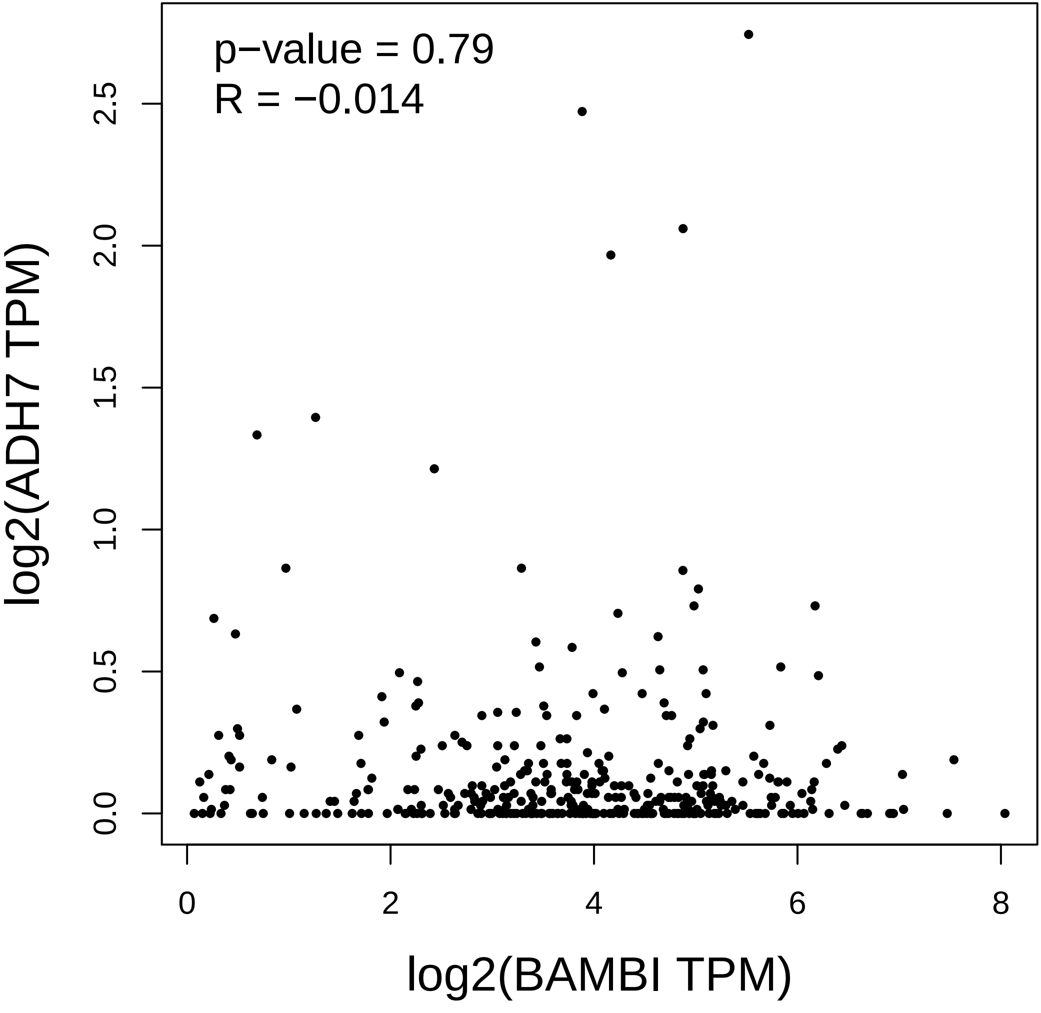
p−value = 0.79
R = −0.014
log2(ADH7 TPM)
0.0
0.5
1.0
1.5
2.0
2.5
0
2
4
6
8
log2(BAMBI TPM)

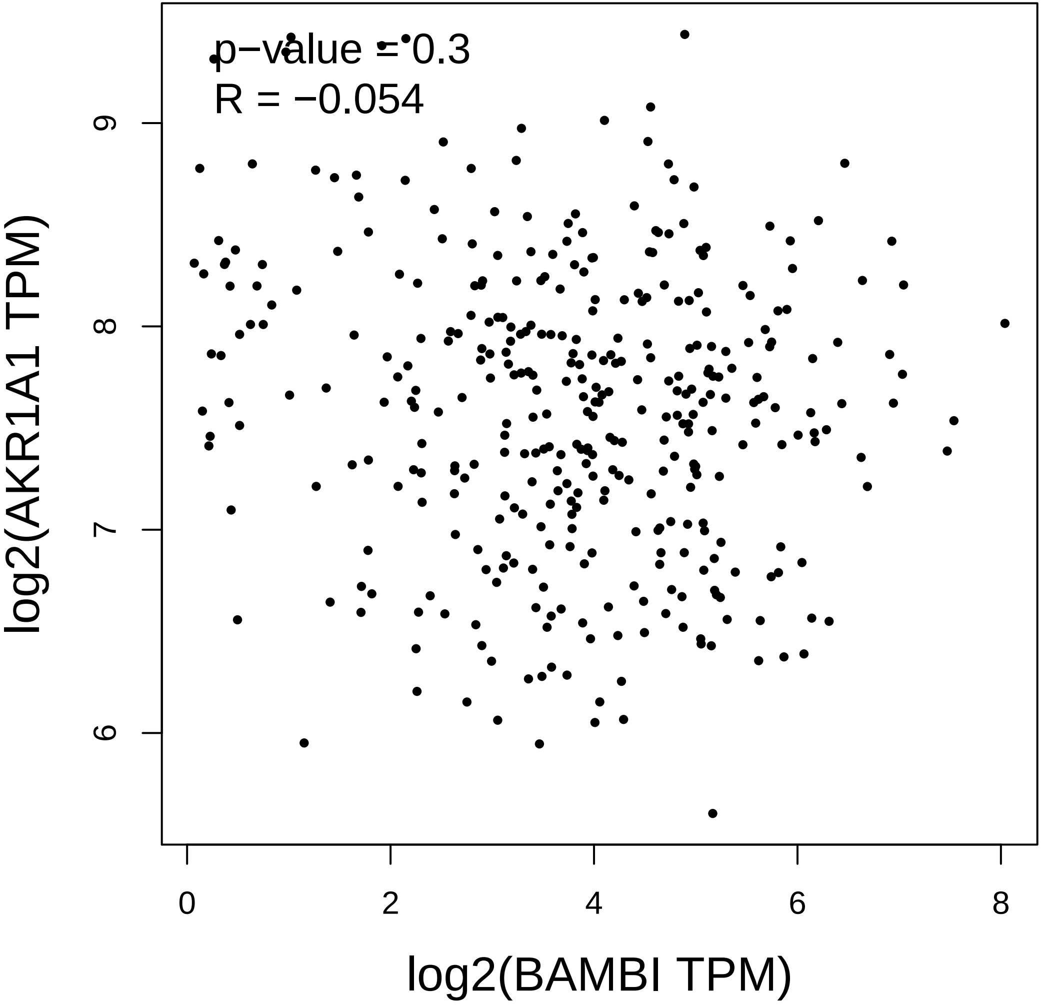
p−value = 0.3
R = −0.054
log2(AKR1A1 TPM)
6
7
8
9
0
2
4
6
8
log2(BAMBI TPM)

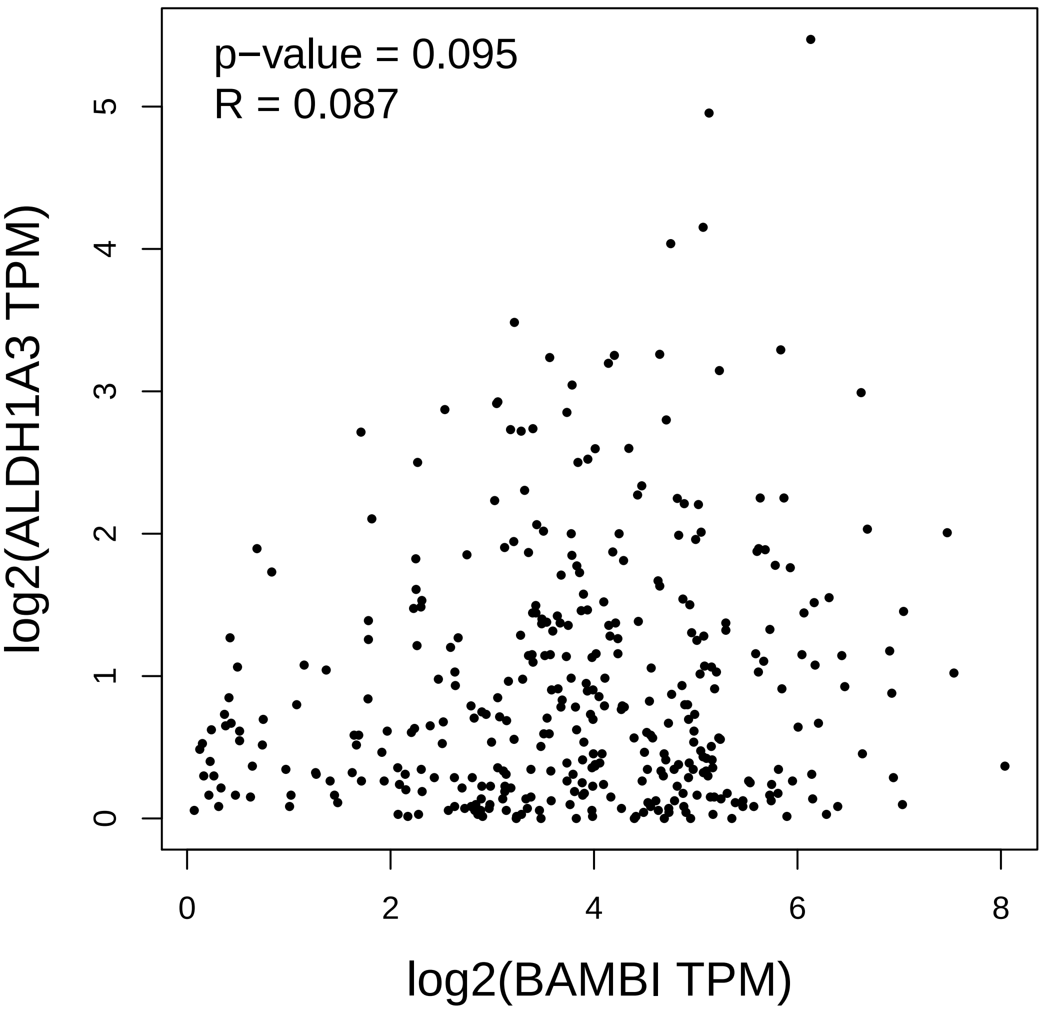
p−value = 0.095
R = 0.087
log2(ALDH1A3 TPM)
log2(BAMBI TPM)
0
1
2
3
4
5
0
2
4
6
8

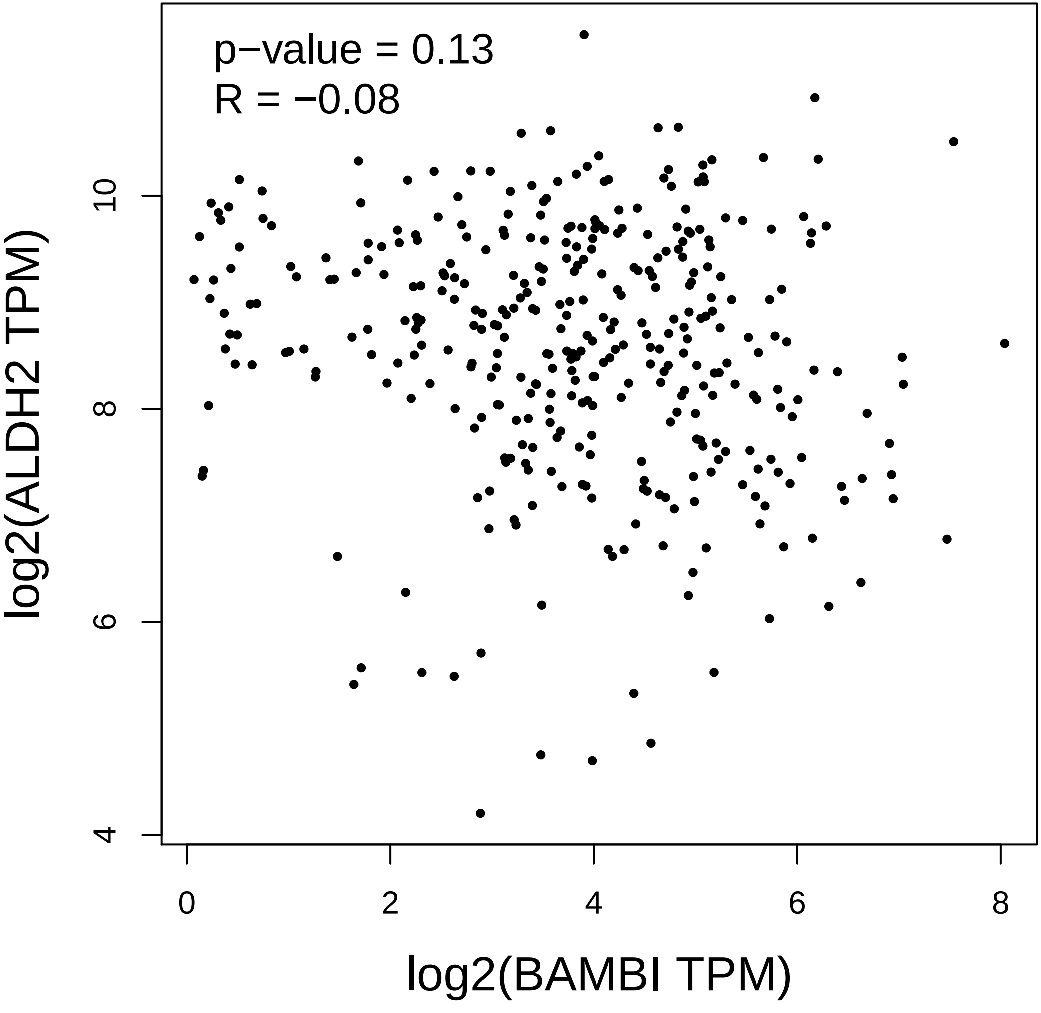
p−value = 0.13
R = −0.08
log2(ALDH2 TPM)
log2(BAMBI TPM)
4
6
8
10
0
2
4
6
8

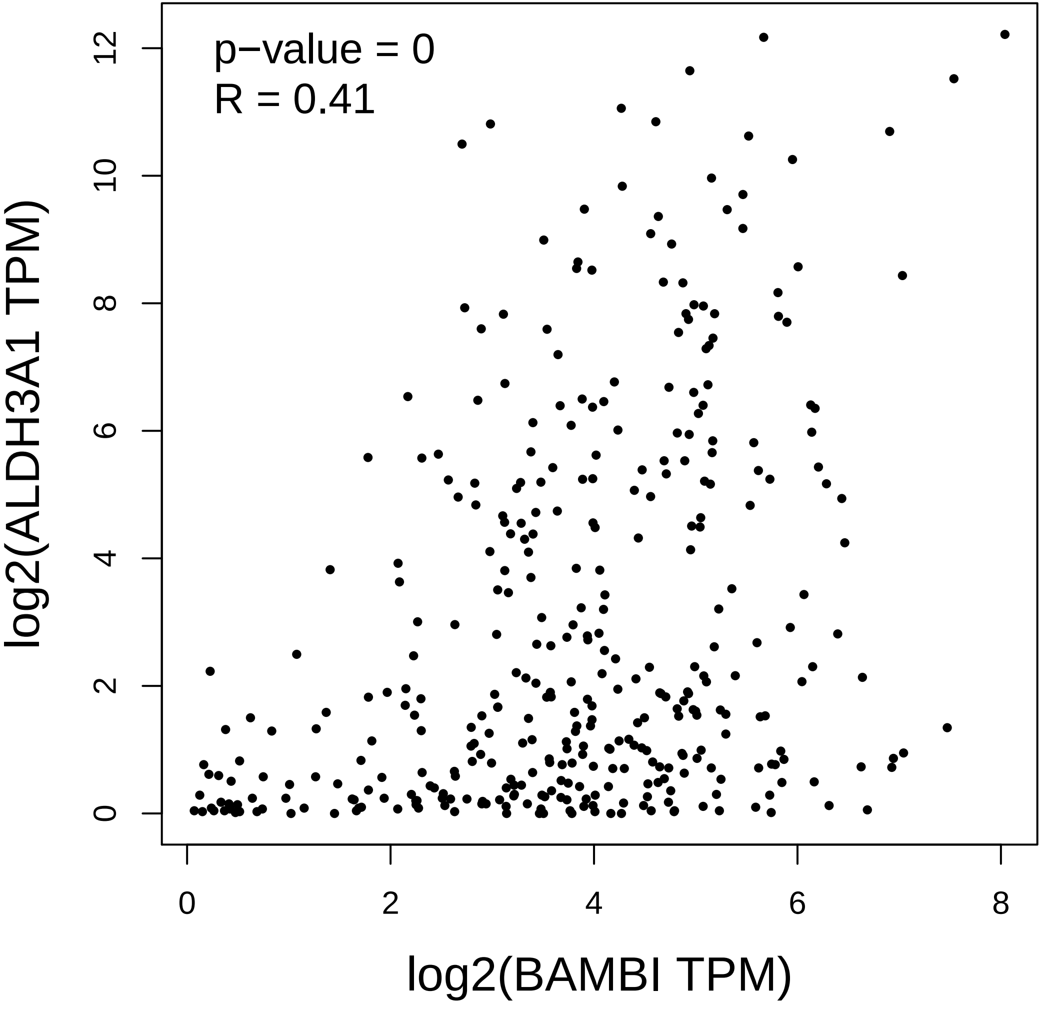

p−value = 0
R = 0.41
log2(ALDH3A1 TPM)
log2(BAMBI TPM)
0
2
4
6
8
10
12

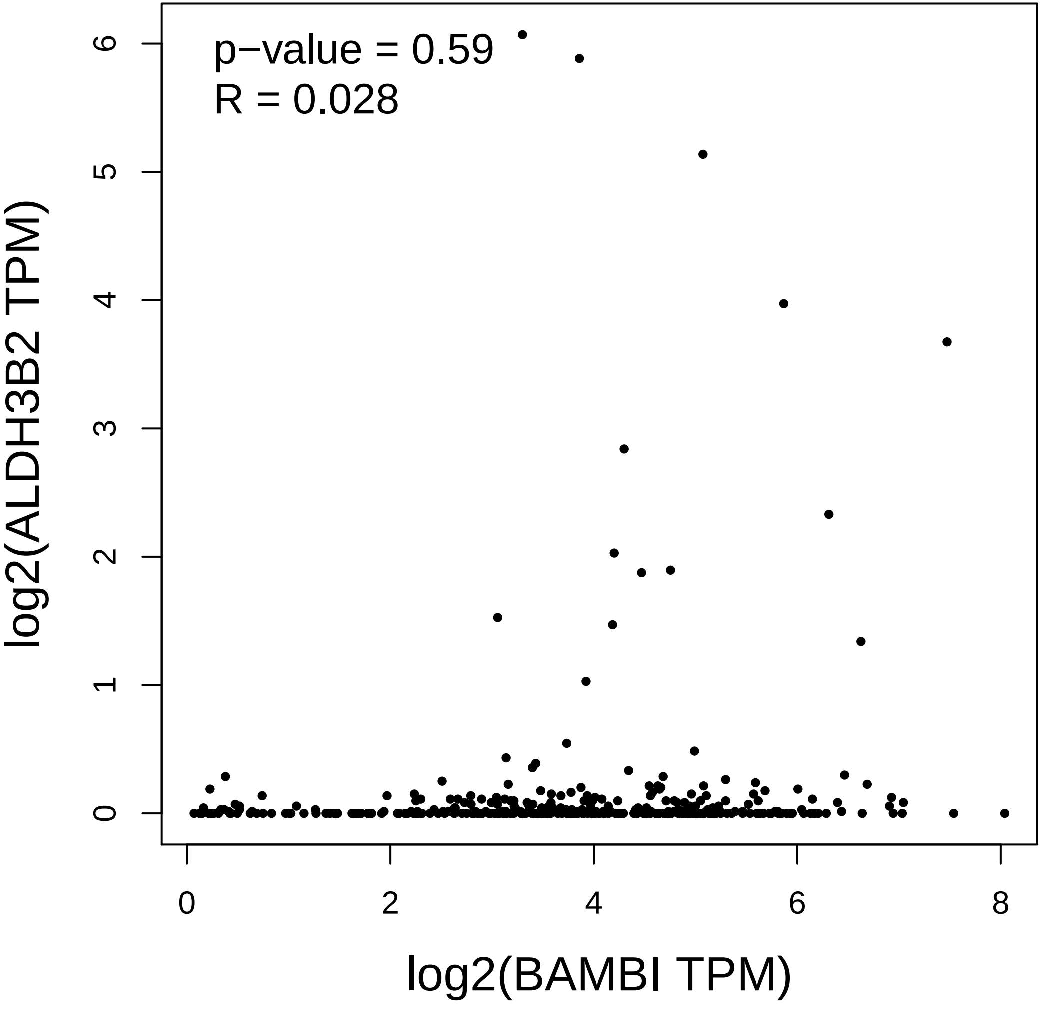
p−value = 0.59
R = 0.028
log2(ALDH3B2 TPM)
log2(BAMBI TPM)
0
1
2
3
4
5
6
0
2
4
6
8

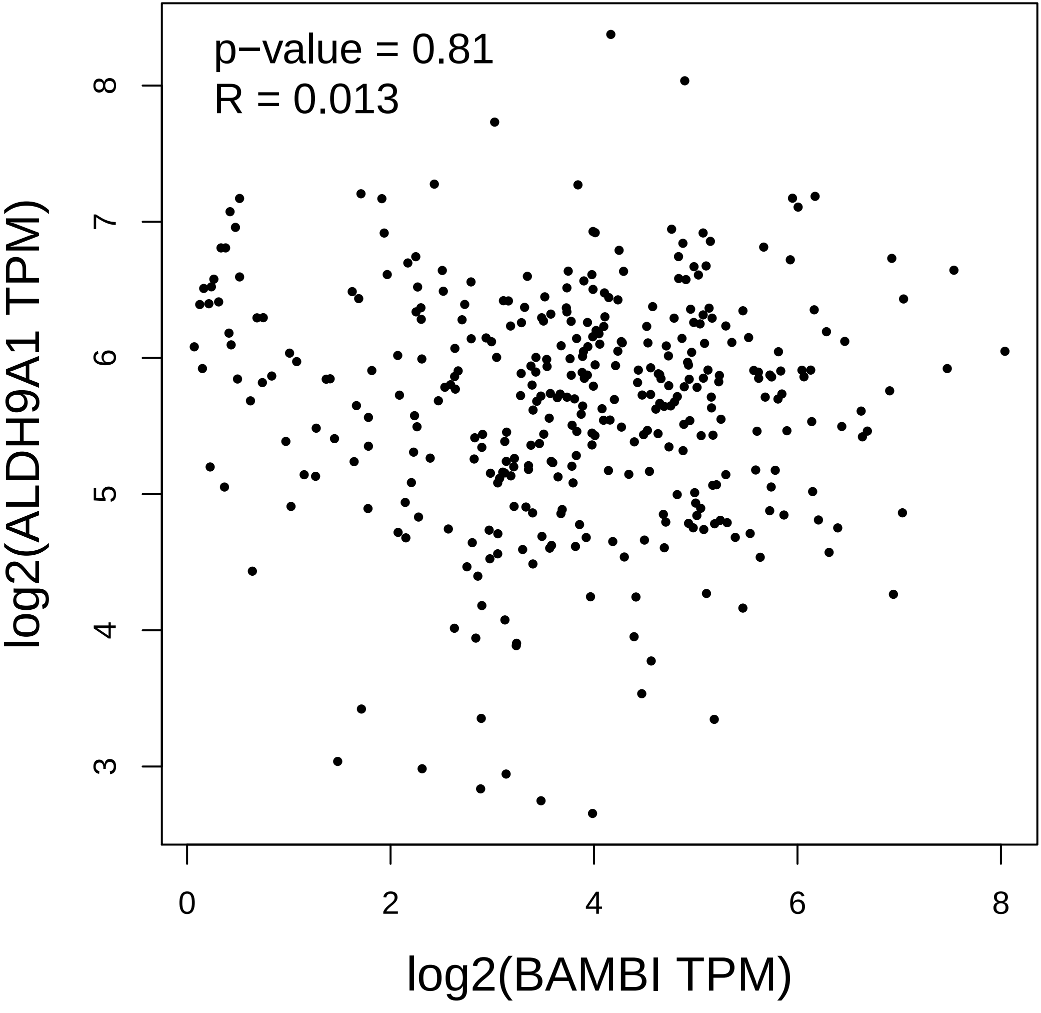
p−value = 0.81
R = 0.013
log2(ALDH9A1 TPM)
log2(BAMBI TPM)
3
4
5
6
7
8
0
2
4
6
8

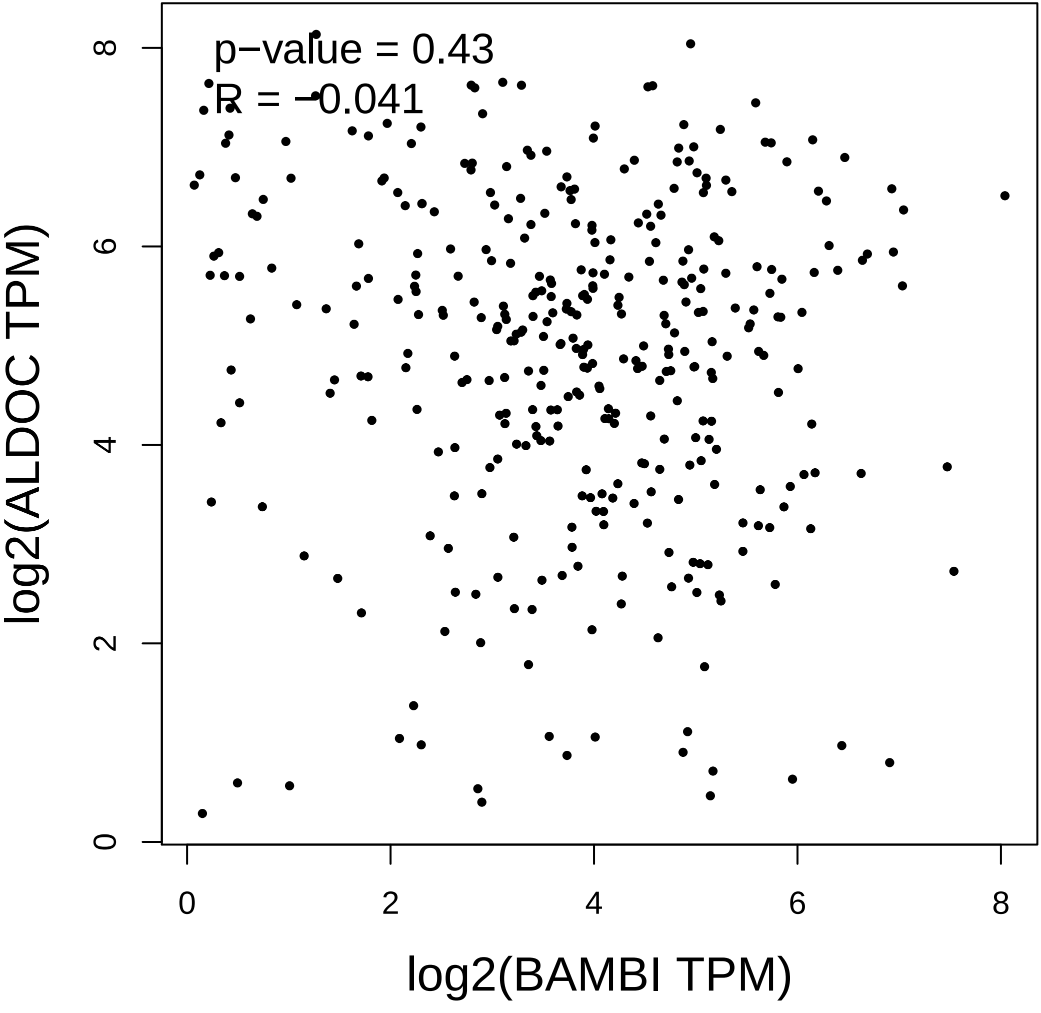
p−value = 0.43
R = −0.041
log2(ALDOC TPM)
0
2
4
6
8
log2(BAMBI TPM)
0
2
4
6
8

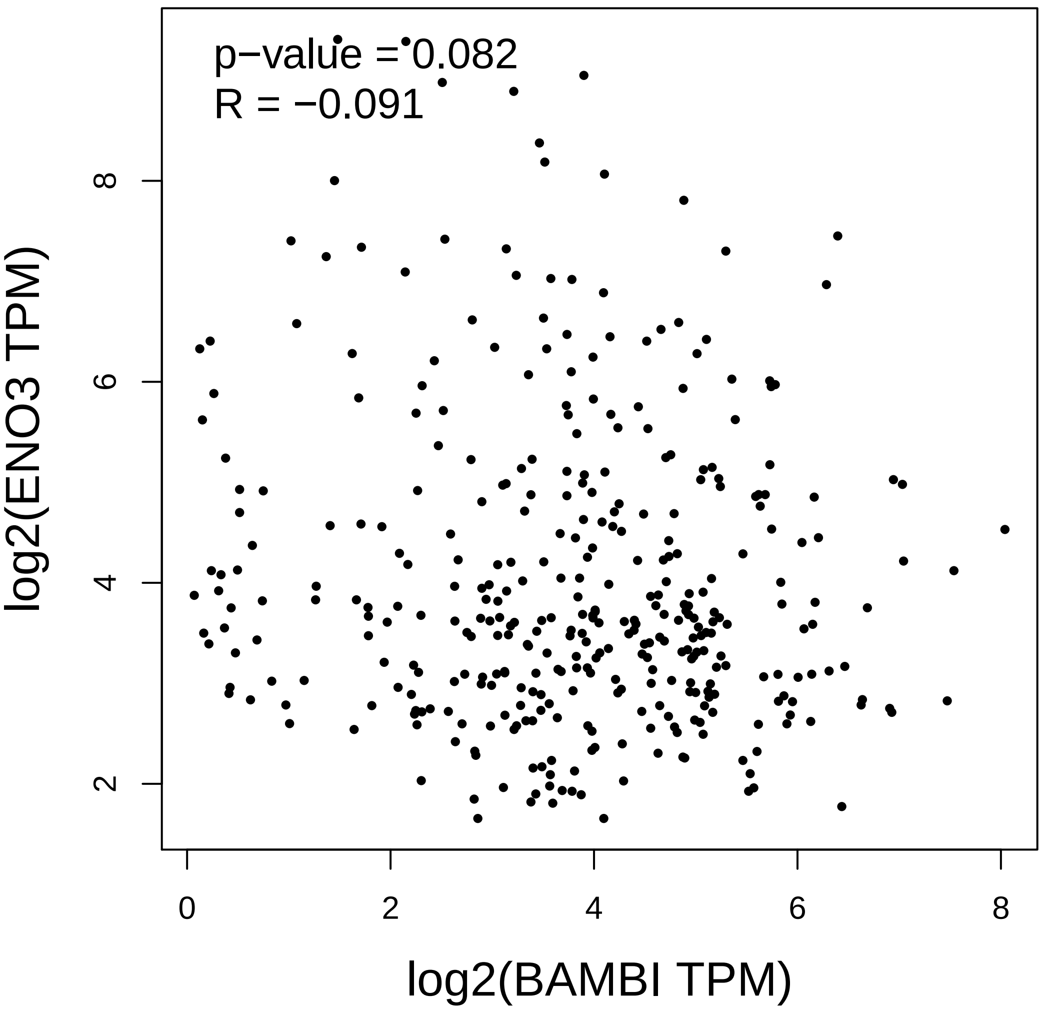
p−value = 0.082
R = −0.091
log2(ENO3 TPM)
2
4
6
8
0
2
4
6
8
log2(BAMBI TPM)

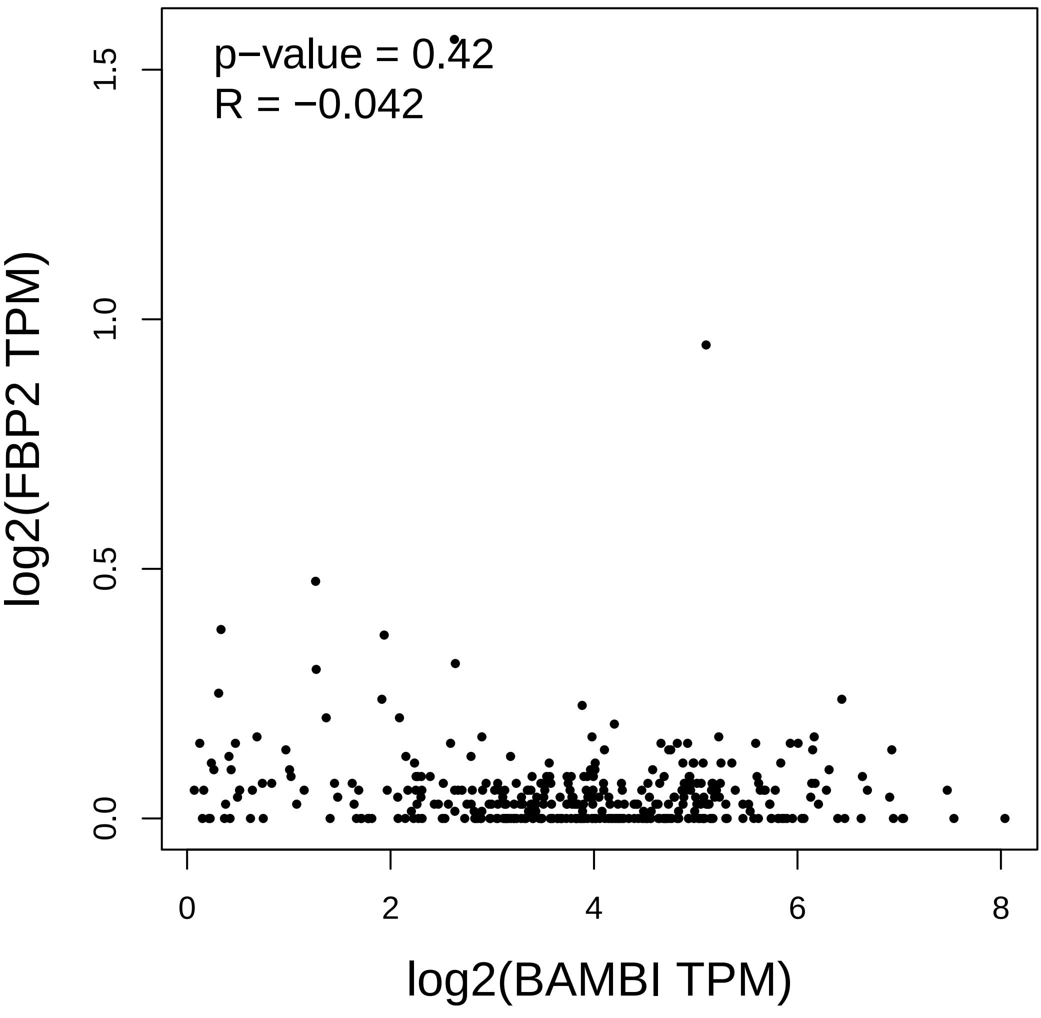
p−value = 0.42
R = −0.042
log2(FBP2 TPM)
log2(BAMBI TPM)
0.0
0.5
1.0
1.5
0
2
4
6
8

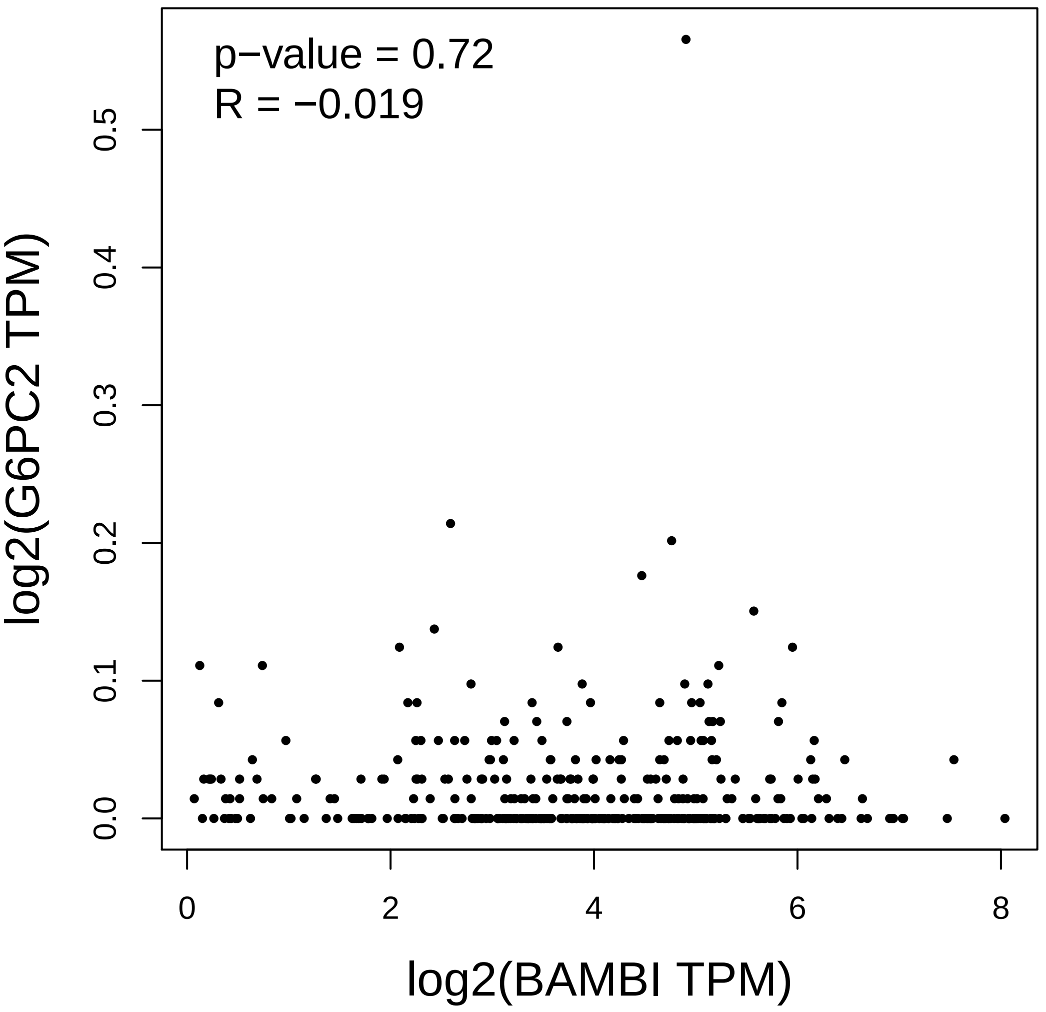
p−value = 0.72
R = −0.019
log2(G6PC2 TPM)
log2(BAMBI TPM)
0.0
0.1
0.2
0.3
0.4
0.5
0
2
4
6
8

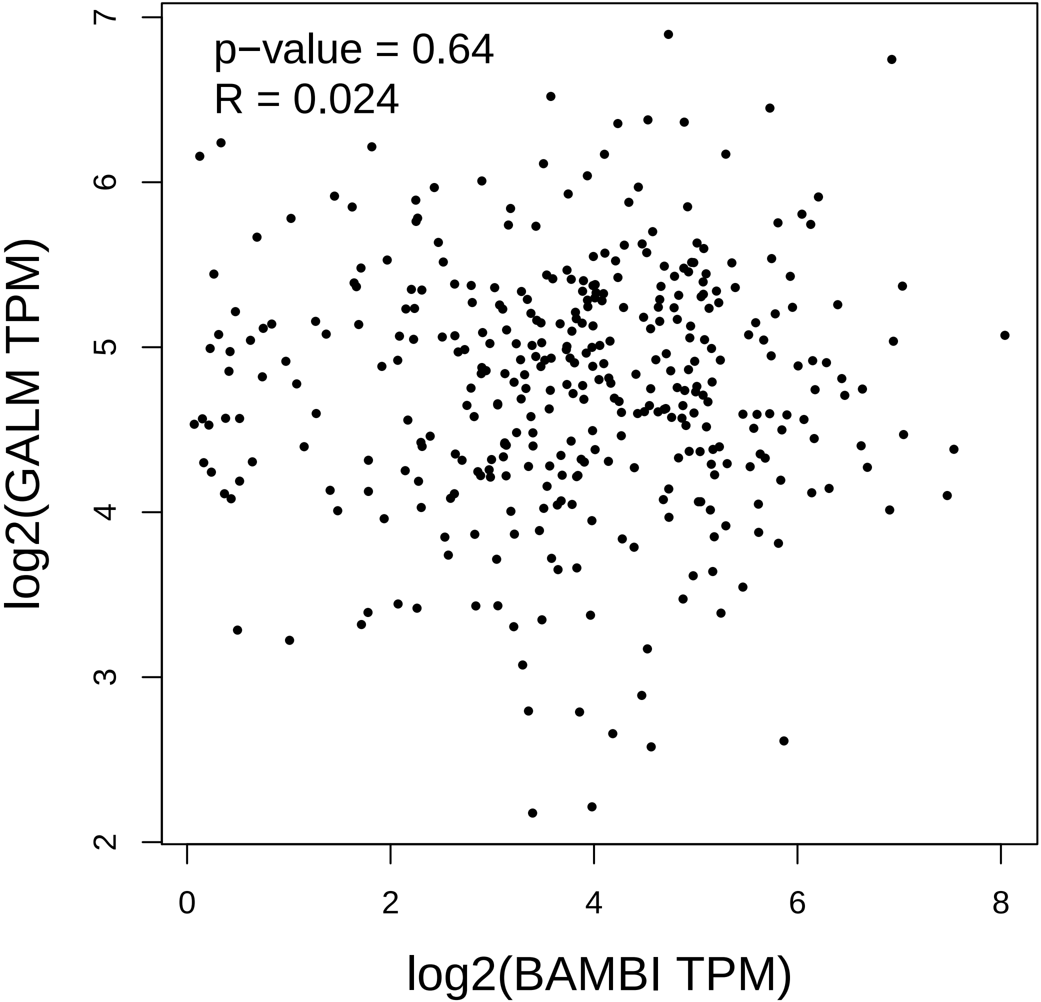

p−value = 0.64
R = 0.024
log2(GALM TPM)
log2(BAMBI TPM)
2
3
4
5
6
7
0
2
4
6
8

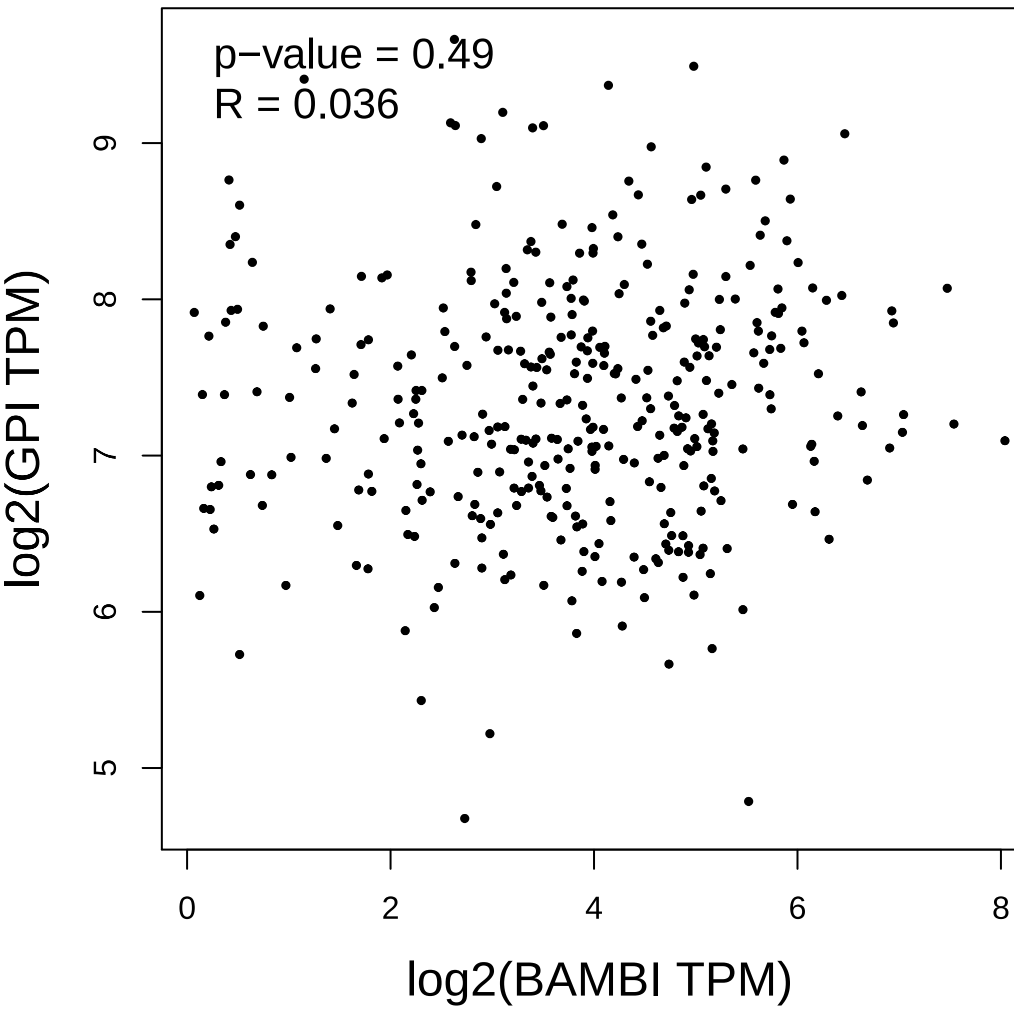
p−value = 0.49
R = 0.036
log2(GPI TPM)
5
6
7
8
9
0
2
4
6
8
log2(BAMBI TPM)

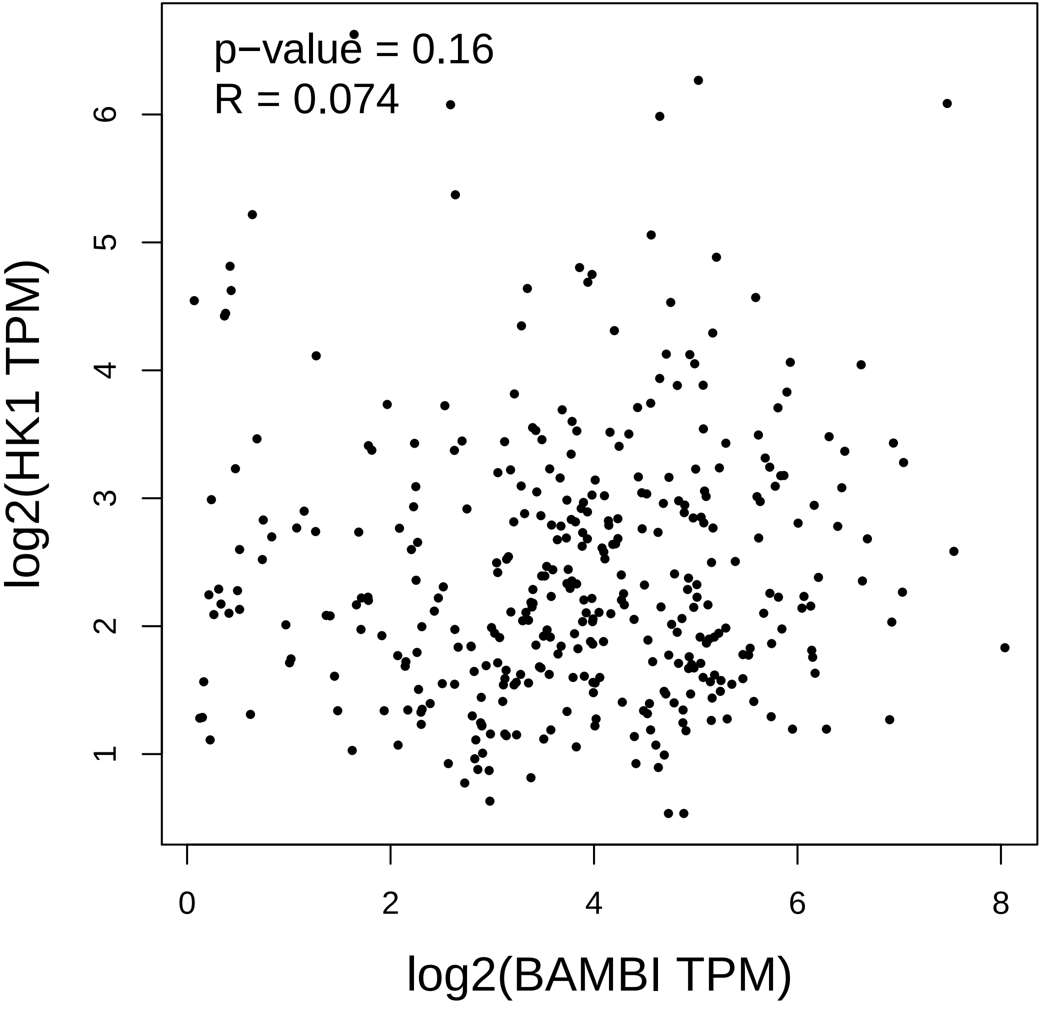
p−value = 0.16
R = 0.074
log2(HK1 TPM)
1
2
3
4
5
6
0
2
4
6
8
log2(BAMBI TPM)

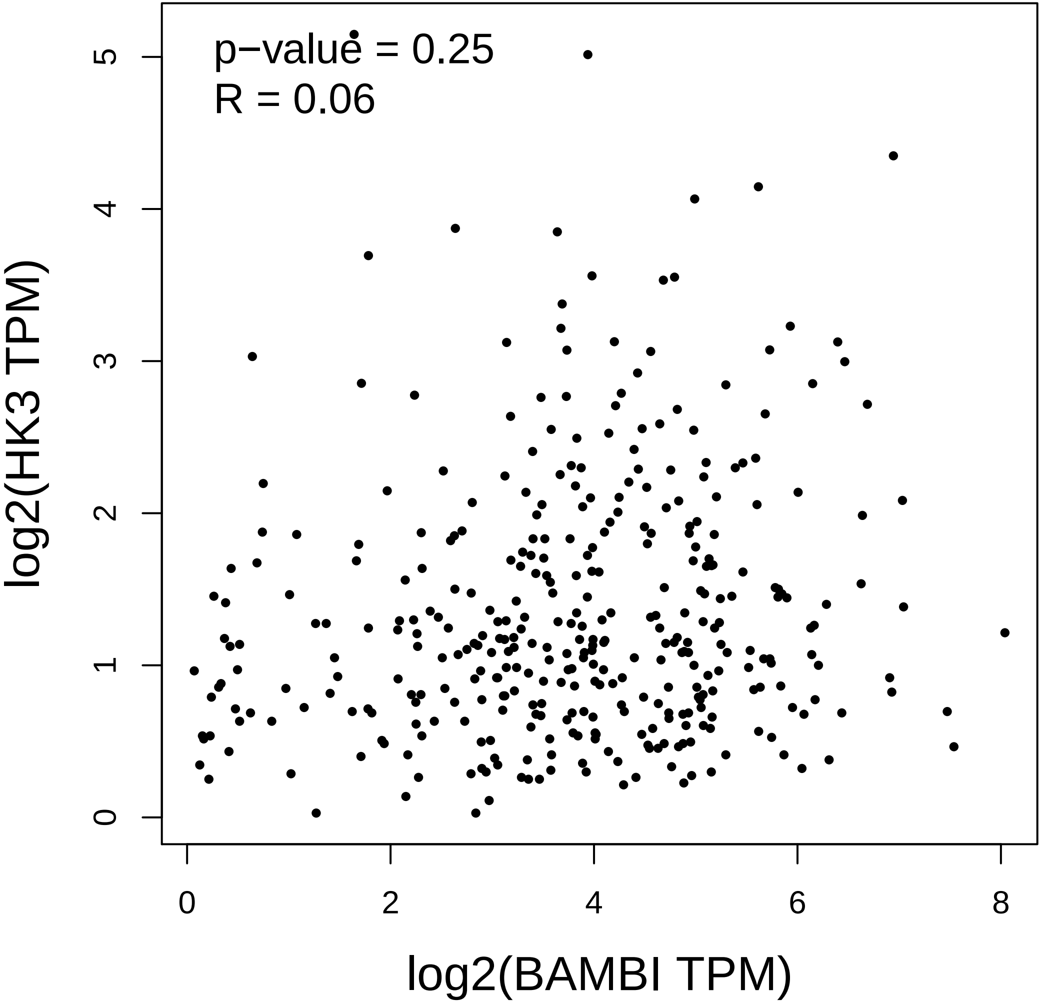
p−value = 0.25
R = 0.06
log2(HK3 TPM)
log2(BAMBI TPM)
0
1
2
3
4
5
0
2
4
6
8

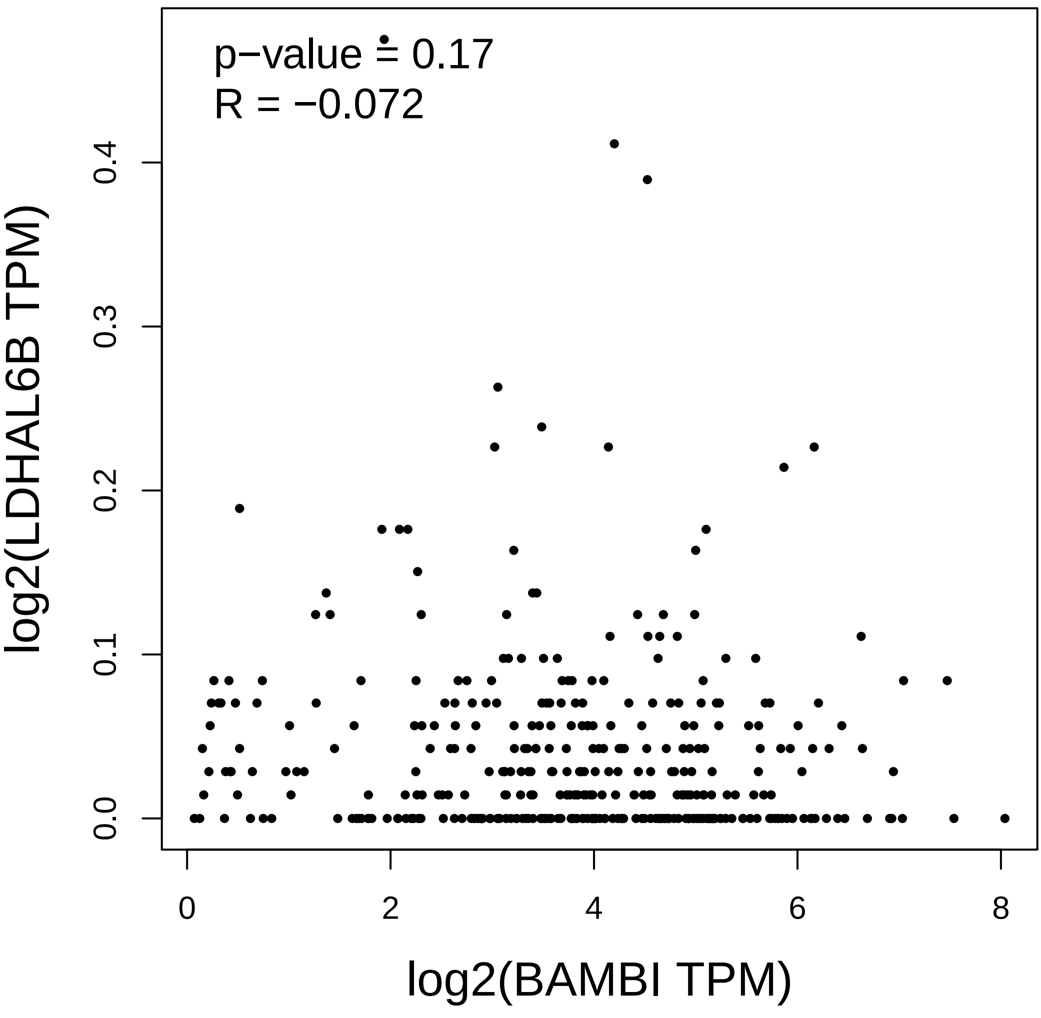
p−value = 0.17
R = −0.072
log2(LDHAL6B TPM)
0.0
0.1
0.2
0.3
0.4
0
2
4
6
8
log2(BAMBI TPM)

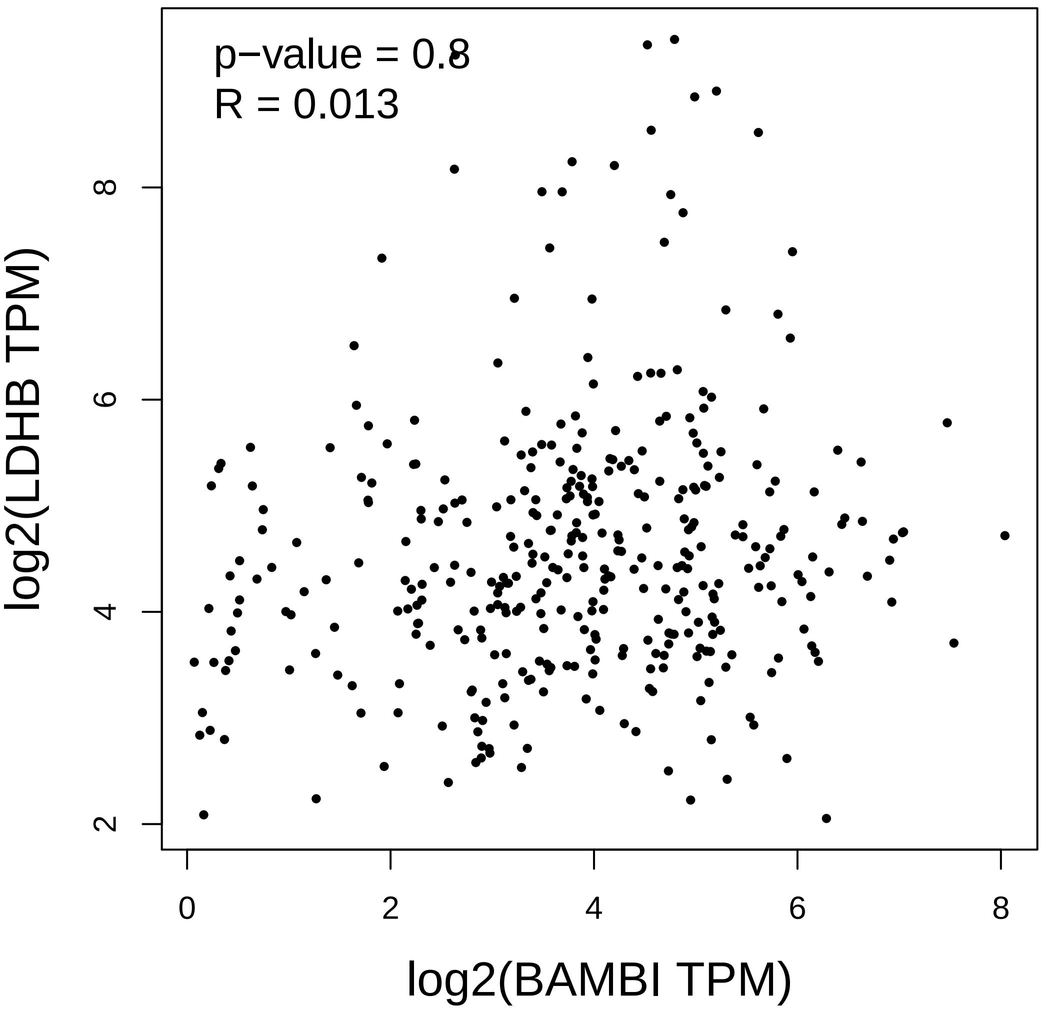
p−value = 0.8
R = 0.013
log2(LDHB TPM)
2
4
6
8
0
2
4
6
8
log2(BAMBI TPM)

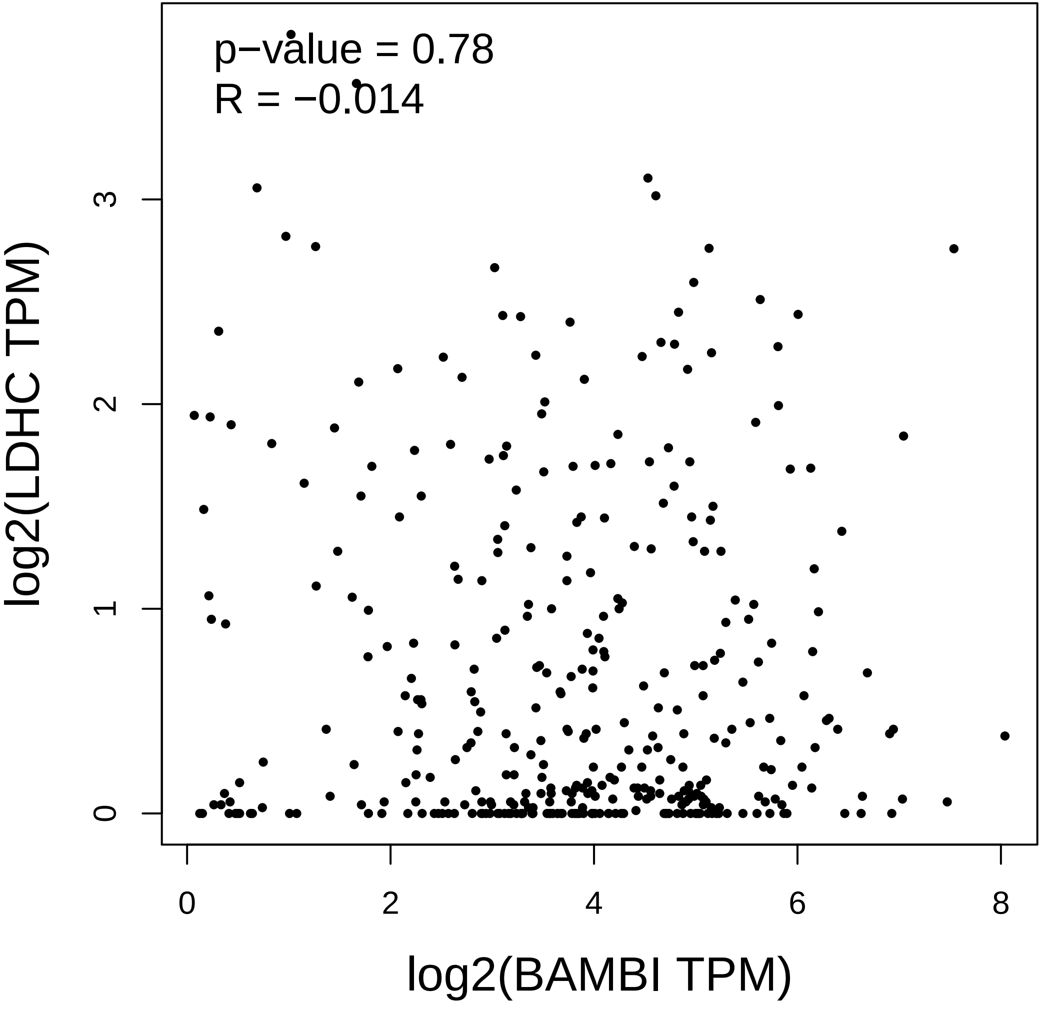

p−value = 0.78
R = −0.014
log2(LDHC TPM)
0
1
2
3
0
2
4
6
8
log2(BAMBI TPM)

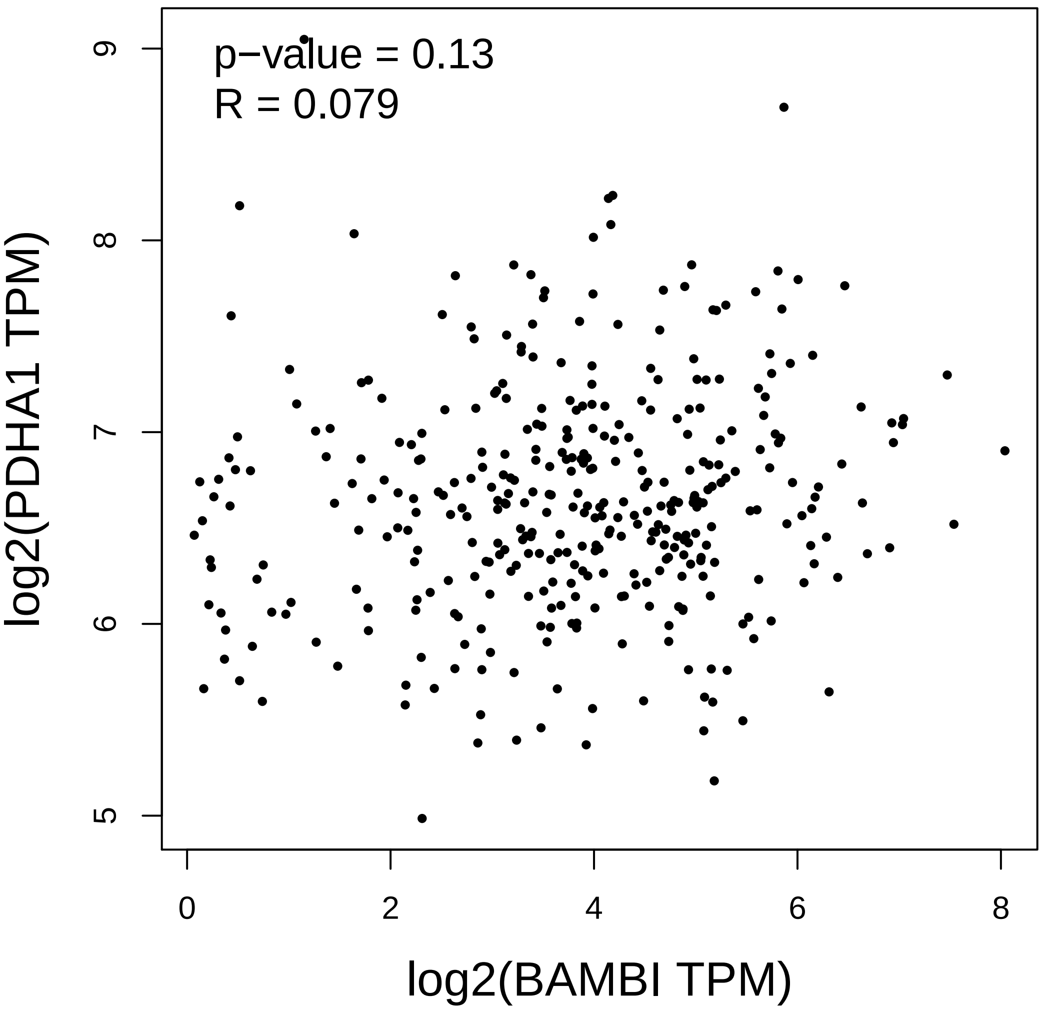

p−value = 0.13
R = 0.079
log2(PDHA1 TPM)
log2(BAMBI TPM)
5
6
7
8
9
0
2
4
6
8

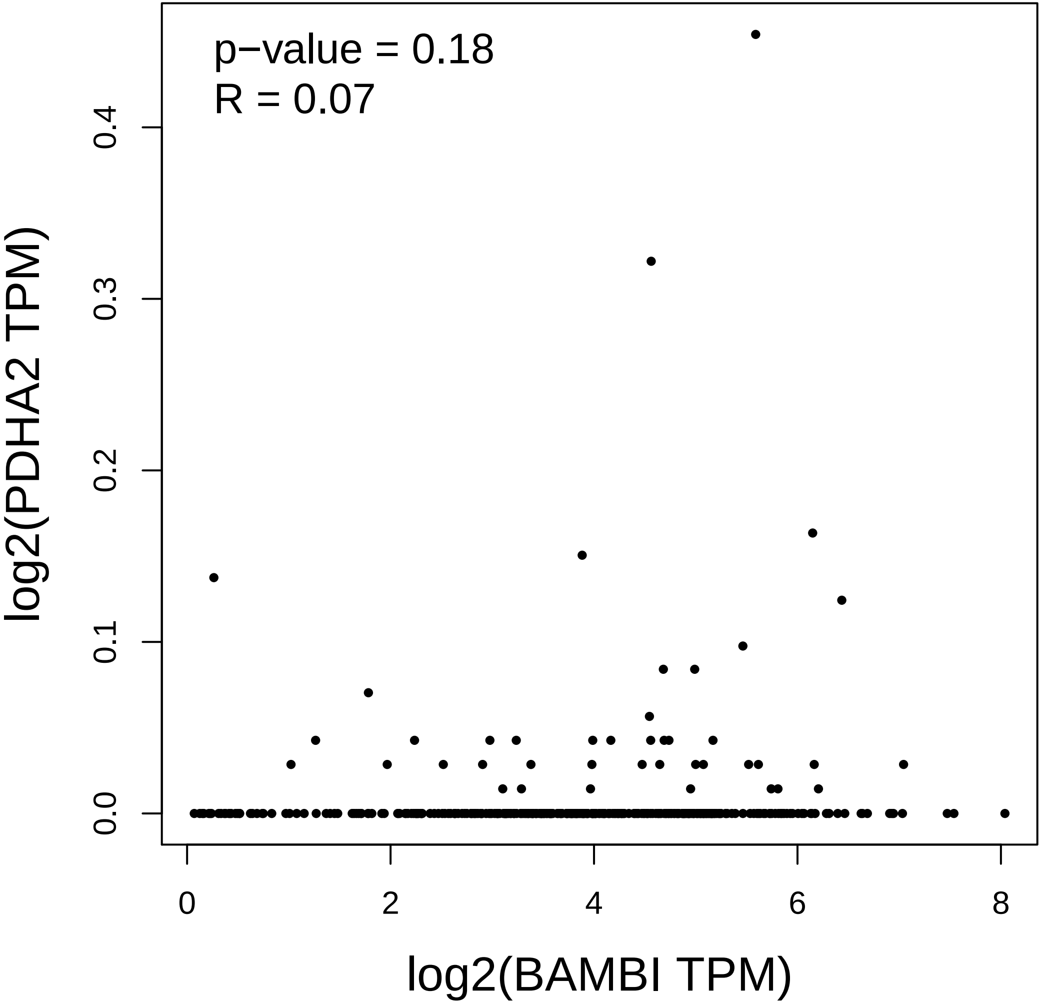
p−value = 0.18
R = 0.07
0.0
0.1
0.2
0.3
0.4
0
2
4
6
8
log2(PDHA2 TPM)
log2(BAMBI TPM)

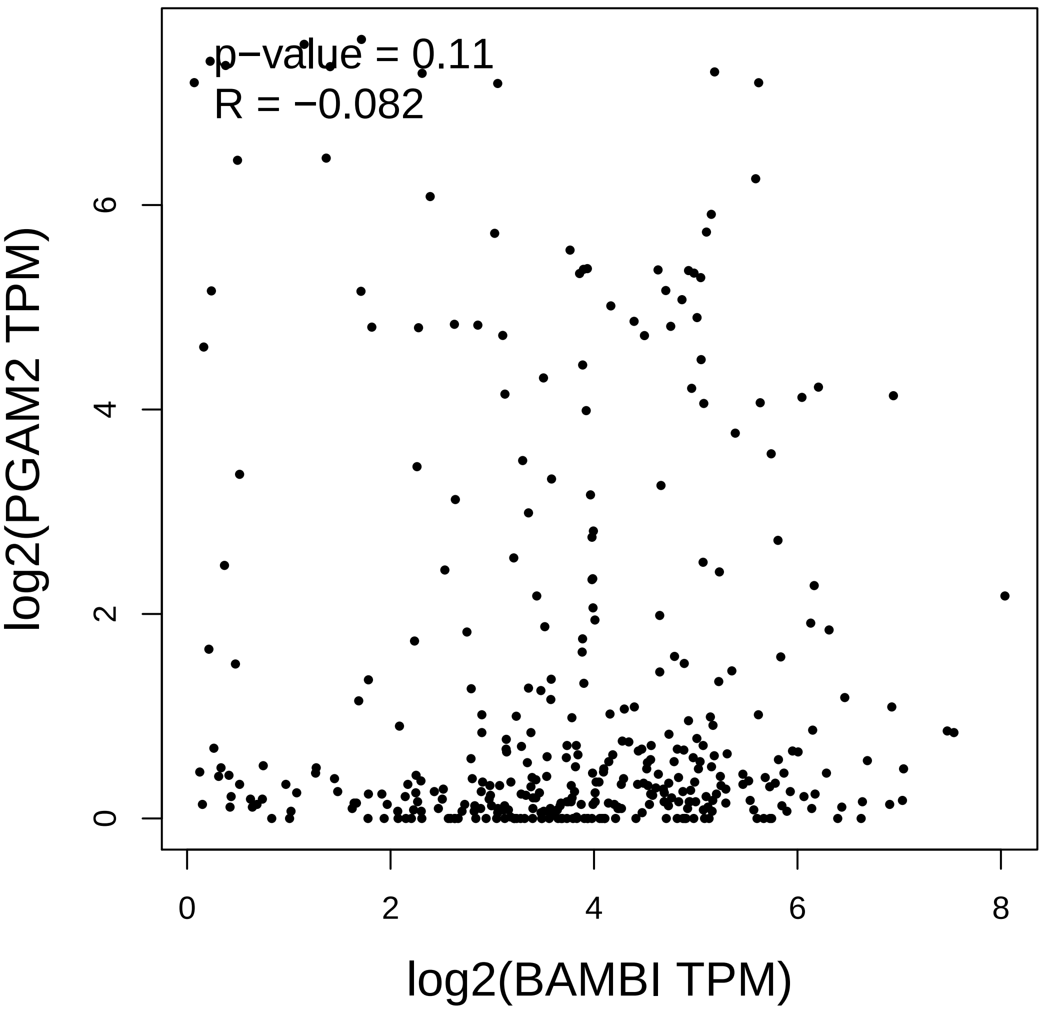
p−value = 0.11
R = −0.082
log2(PGAM2 TPM)
log2(BAMBI TPM)
0
2
4
6
0
2
4
6
8

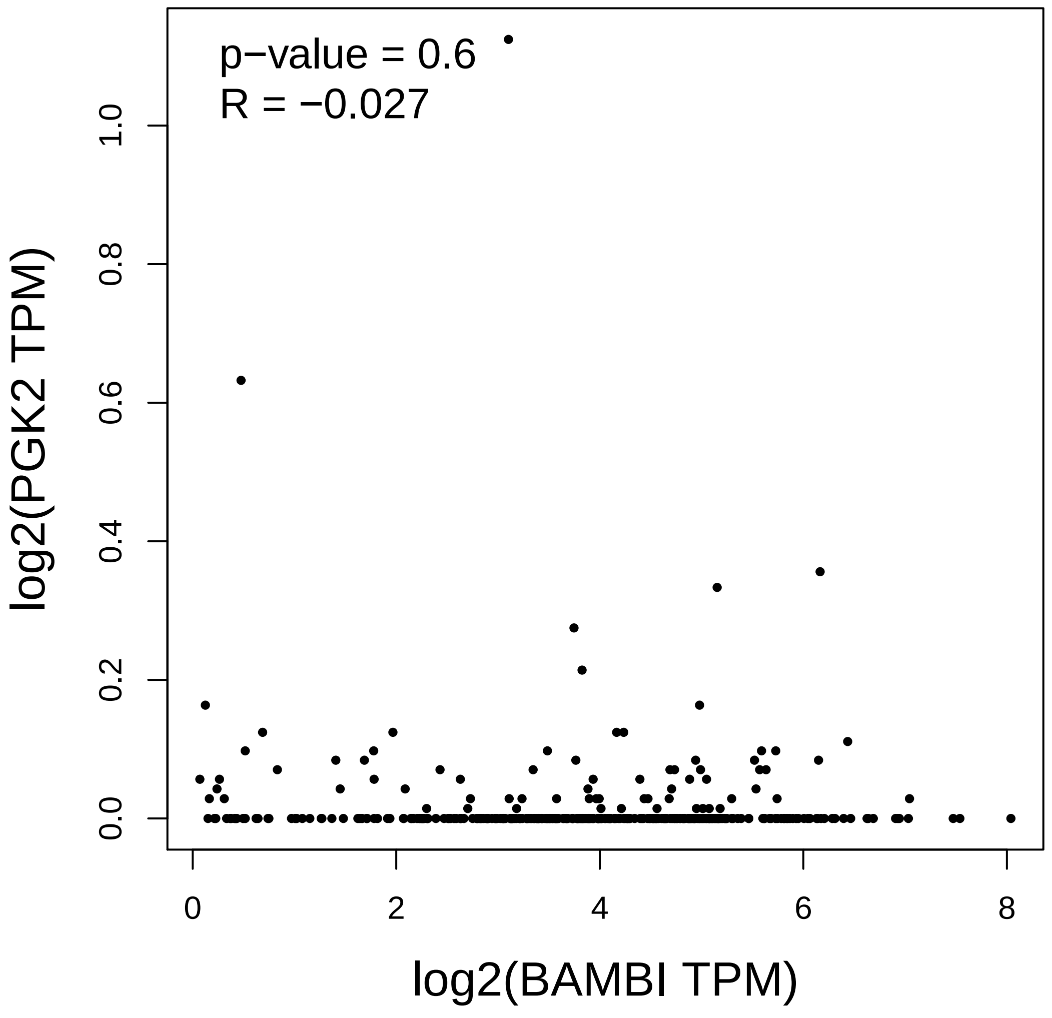
p−value = 0.6
R = −0.027
log2(PGK2 TPM)
log2(BAMBI TPM)
0.0
0.2
0.4
0.6
0.8
1.0
0
2
4
6
8

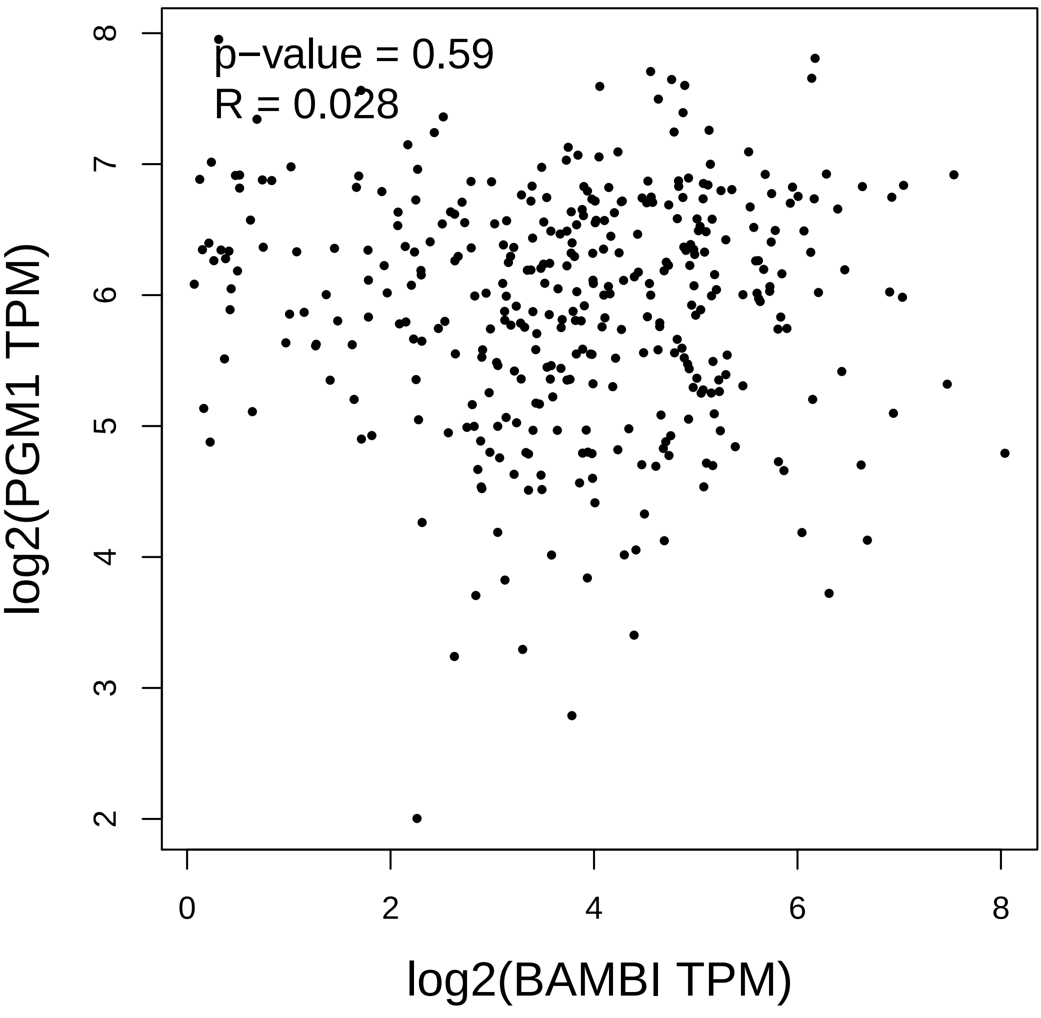
p−value = 0.59
R = 0.028
log2(PGM1 TPM)
log2(BAMBI TPM)
0
2
4
6
8
2
3
4
5
6
7
8

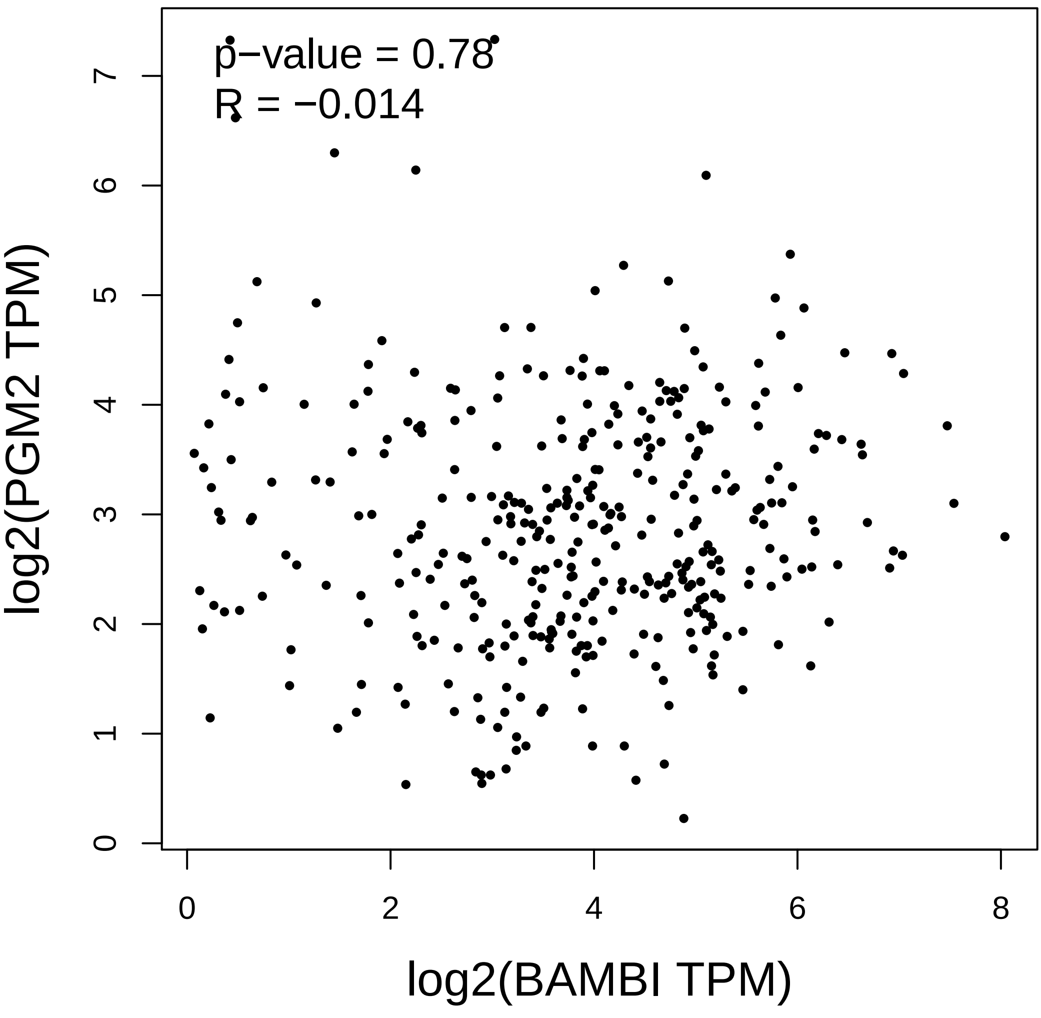
p−value = 0.78
R = −0.014
log2(PGM2 TPM)
log2(BAMBI TPM)
0
1
2
3
4
5
6
7
0
2
4
6
8

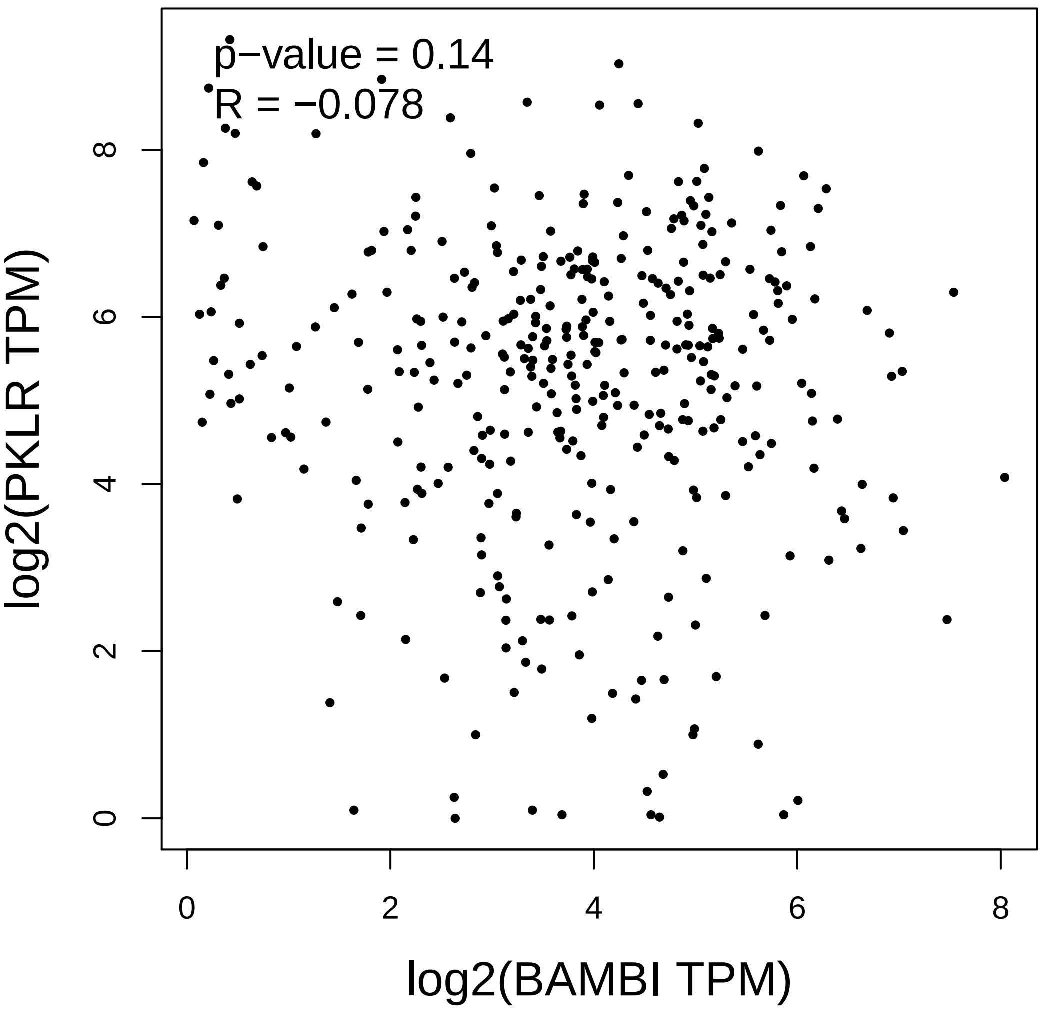

p−value = 0.14
R = −0.078
log2(PKLR TPM)
0
2
4
6
8
log2(BAMBI TPM)
0
2
4
6
8
