# Supplementary material for: BAMBI Is a Prognostic Biomarker Associated with Macrophage Polarization, Glycolysis, and Lipid Metabolism in Hepatocellular Carcinoma
Source: Int J Mol Sci. 2024 Nov 26;25(23):12713. doi: 10.3390/ijms252312713 (PMC11640931; doi:10.3390/ijms252312713)
Supplement: Supplementary file 1 [file ijms-25-12713-s001.zip › Supplementary material 241125.pdf]

Supplementary Material

Table S1. Sequences of primers used for reverse transcription-quantitative PCR.

Target	Sequence (5'-3')
BAMBI	F: TCACTGGGGCATGTACAGTG<br>R: TGGTGACAGTGTGTACAAAG
Human CD11c	F: AGAGCTGTGATAAGCCAGTTCC<br>R: AATTCCTCGAAAGTGAAGTGTGT
Human Il-12	F: CCTTGCACTTCTGAAGAGATTGA<br>R: ACAGGGCCATCATAAAAGAGGT
Human CD206	F: GGGTTGCTATCACTCTCTATGC<br>R: TTTCTTGTCTGTTGCCGTAGTT
Human ARG-1	F: CCAGTCCGTCAACATCAAAACT<br>R: CCAGTCCGTCAACATCAAAACT
Human GAPDH	F: GGAGCGAGATCCCTCCAAAAT<br>R: GGCTGTTGTCATACTTCTCATGG
Human SLC2A1	F: GGCCAAGAGTGTGCTAAAGAA<br>R: ACAGCGTTGATGCCAGACAG
Human PIGQ	F: GCAGTGACCGCTTTGATGAG<br>R: GTACTGAAGATTAGCGTGAGGTG
Human PKM	F: ATGTCGAAGCCCCATAGTGAA<br>R: TGGGTGGTGAATCAATGTCCA
Human LDHA	F: ATGGCAACTCTAAAGGATCAGC<br>R: CCAACCCCAACAACTGTAATCT
Human SIRT1	F: TAGCCTTGTCAGATAAGGAAGGA<br>R: ACAGCTTCACAGTCAACTTTGT
Human ACACA	F: ATGTCTGGCTTGCACCTAGTA<br>R: CCCCAAAGCGAGTAACAAATTCT
Human ACTIN	F: CGTGACATTAAGGAGAAGCTG<br>R: CTAGAAGCATTTGCGGTGGAC
Mouse CD11c	F: CTGGATAGCCTTTCTTCTGCTG<br>R: GCACACTGTGTCCGAACTCA
Mouse Il-12	F: CAATCACGCTACCTCCTCTTTT<br>R: CAGCAGTGCAGGAATAATGTTTC
Mouse CD206	F: CTCTGTTCAGCTATTGGACGC<br>R: TGGCACTCCCAAACATAATTTGA
Mouse ARG-1	F: CTCCAAGCCAAAGTCCTTAGAG<br>R: GGAGCTGTCATTAGGGACATCA
Mouse GAPDH	F: AGGTCGGTGTGAACGGATTTG<br>R: GGGGTCGTTGATGGCAACA
Mouse SLC2A1	F: GCAGTTCGGCTATAACACTGG<br>R: GCGGTGGTTCCATGTTTGATTG
Mouse PIGQ	F: CAGTGGCCTGTTAGTGGGAC<br>R: CAGGGGTCATGGGAGAAGATG

Mouse PKM	F: CGCCTGGACATTGACTCTG<br>R: GAAATTCAGCCGAGCCACATT
Mouse LDHA	F: CAAAGACTACTGTGTAACTGCGA<br>R: TGGACTGTACTTGACAATGTTGG
Mouse SIRT1	F: TGATTGGCACCGATCCTCG<br>R: CCACAGCGTCATATCATCCAG
Mouse ACACA	F: CTCCCGATTCATAATTGGGTCTG<br>R: TCGACCTTGTTTTACTAGGTGC
Mouse ACTIN	F: GTGACGTTGACATCCGTAAAGA<br>R: GCCGGACTCATCGTACTCC

F (forward); R (reverse); BAMBI (bone morphogenic protein and activin membrane-bound inhibitor homolog); CD11c (integrin subunit alpha X, ITGAX); IL-12A (Interleukin 12A); CD206 (mannose receptor C-type 1, MRC1); ARG-1 (arginase 1).

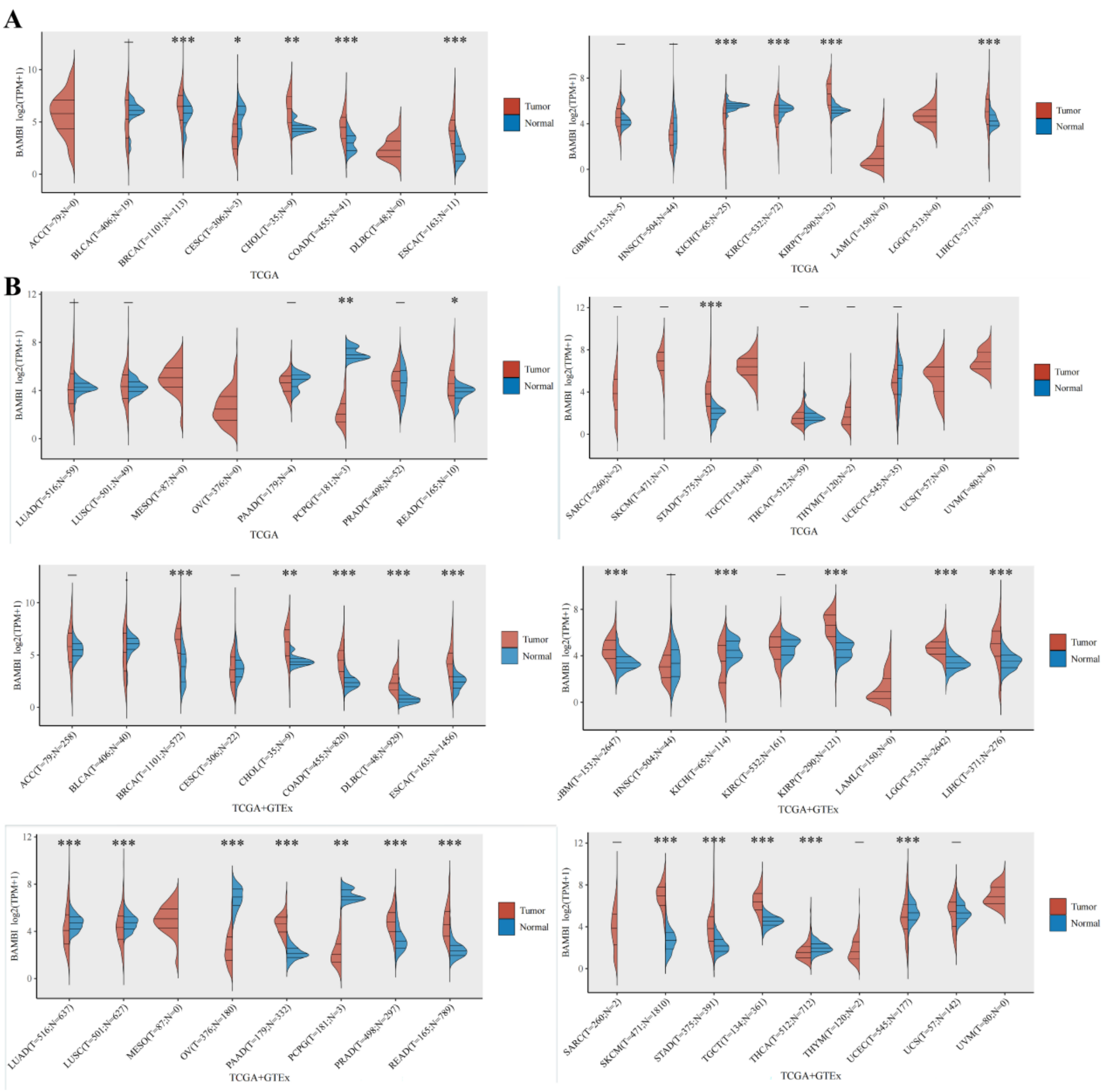

Figure S1. Analysis of BAMBI expression in various cancers. (A) Expression of BAMBI in 24 types of human cancers based on TCGA data for cancer and normal tissues. BAMBI expression was increased in 8 types of cancer tissues and decreased in 4 types of cancer tissues compared with the corresponding normal tissues. (B) Expression of BAMBI in 24 types of human cancers based on TCGA data for cancer and normal tissues, as well as GTEx data for normal tissues. BAMBI expression was higher in 15 types of cancer tissues and lower in 7 types of cancer tissues compared with the corresponding normal tissues. *P<0.05; **P<0.01; ***P<0.001. Adrenocortical carcinoma (ACC), bladder urothelial carcinoma (BLCA), breast invasive carcinoma (BRCA), cervical squamous

cell carcinoma and endocervical adenocarcinoma (CESC), cholangiocarcinoma (CHOL), colon adenocarcinoma (COAD), lymphoid neoplasm diffuse large B-cell lymphoma (DLBC), esophageal carcinoma (ESCA), glioblastoma multiforme (GBM), head and neck squamous cell carcinoma (HNSC), kidney chromophobe (KICH), kidney renal clear cell carcinoma (KIRC), kidney renal papillary cell carcinoma (KIRP), acute myeloid leukemia (LAML), brain lower grade glioma (LGG), liver hepatocellular carcinoma (LIHC, also known as HCC), lung adenocarcinoma (LUAD), lung squamous cell carcinoma (LUSC), mesothelioma (MESO), ovarian serous cystadenocarcinoma (OV), pancreatic adenocarcinoma (PAAD), pheochromocytoma and paraganglioma (PCPG), prostate adenocarcinoma (PRAD), rectal adenocarcinoma (READ), sarcoma (SARC), skin cutaneous melanoma (SKCM), stomach adenocarcinoma (STAD), testicular germ cell tumors (TGCT), thyroid carcinoma (THCA), thymoma (THYM), uterine corpus endometrial carcinoma (UCEC), uterine carcinosarcoma (UCS), and uveal melanoma (UVM).

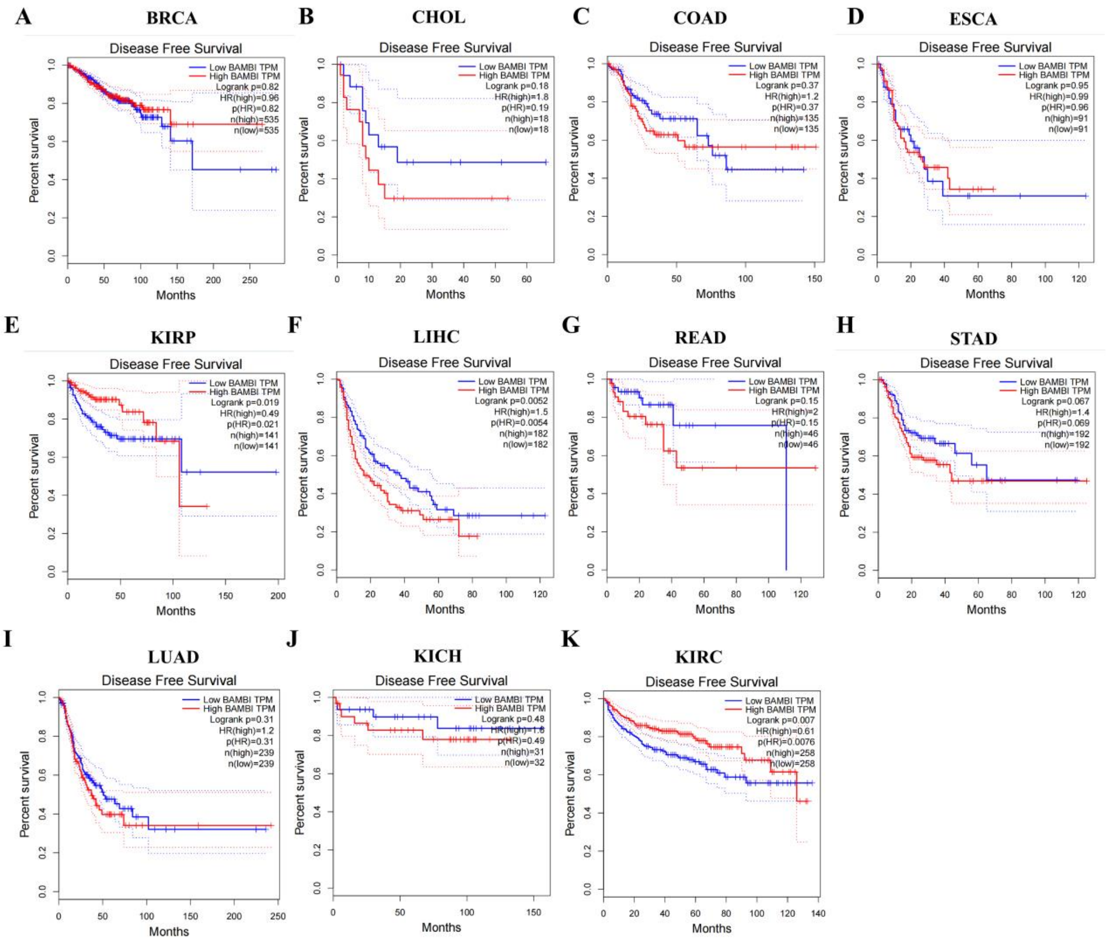

Figure S2. Analysis of disease-free survival according to BAMBI expression in 11 types of cancers, as determined using the GEPIA database. (A-K) Plots of disease-free survival according to BAMBI expression in BRCA (A), CHOL (B), COAD (C), ESCA (D), KIRP (E), HCC (F), READ (G), STAD (H), CESC (I), KICH (J), and KIRC (K). P-values <0.05 were considered statistically significant.

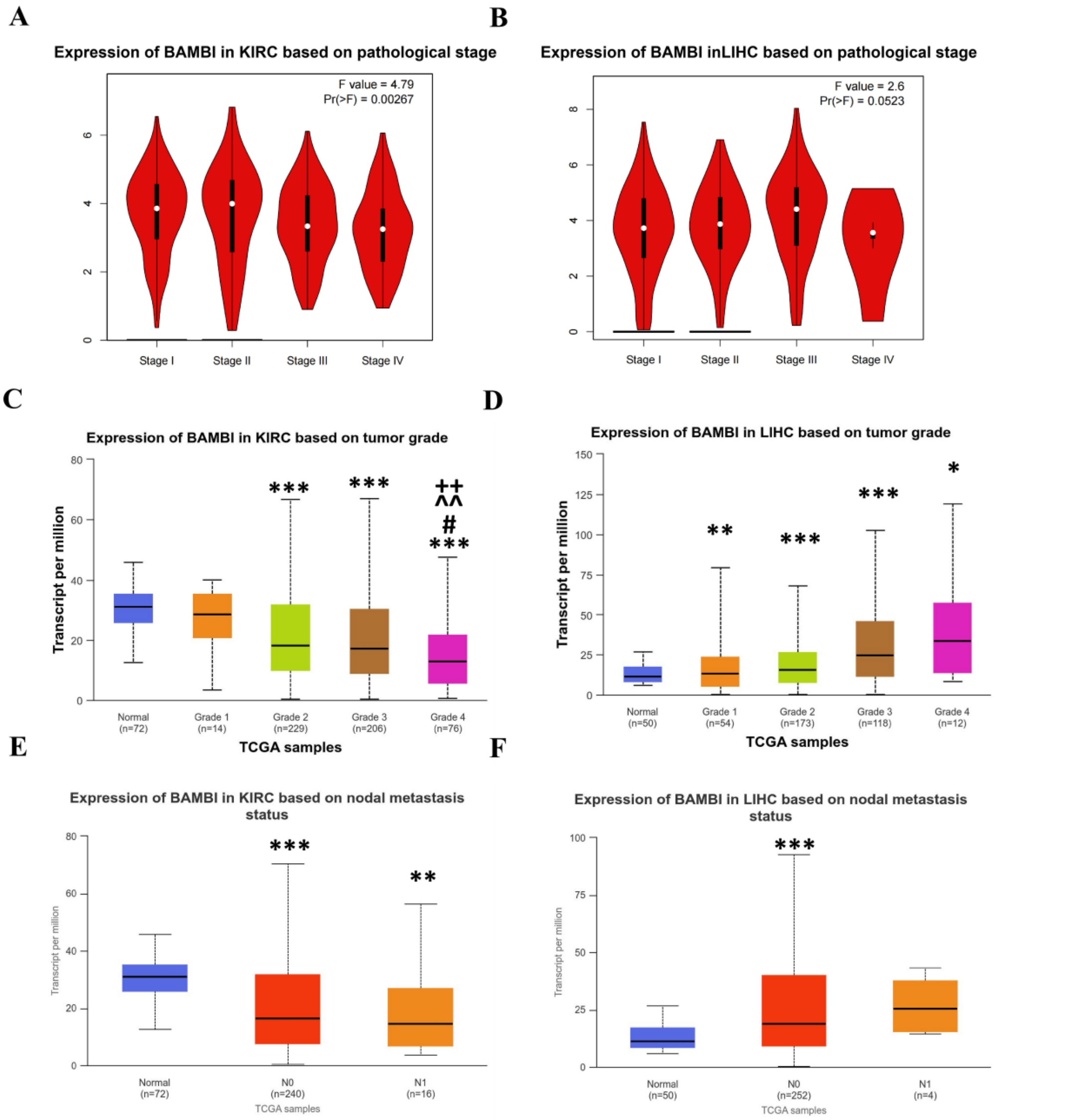

Figure S3. Analyses of pathological stage, tumor grade, and lymph node metastasis according to BAMBI expression in HCC and KIRC, as determined using the GEPIA and UALCAN databases. (A, B) Plots of pathological stage according to BAMBI expression in HCC (A) and KIRC (B). (C, D) Plots of tumor grade according to BAMBI expression in HCC (C) and KIRC (D). (E, F) Plots of lymph node metastasis according to BAMBI expression in HCC (E) and KIRC (F). * vs. normal; # vs. grade 1; ^ vs. grade 2; + vs. grade 3; *P< 0.05; **P<0.01; ***P<0.001.

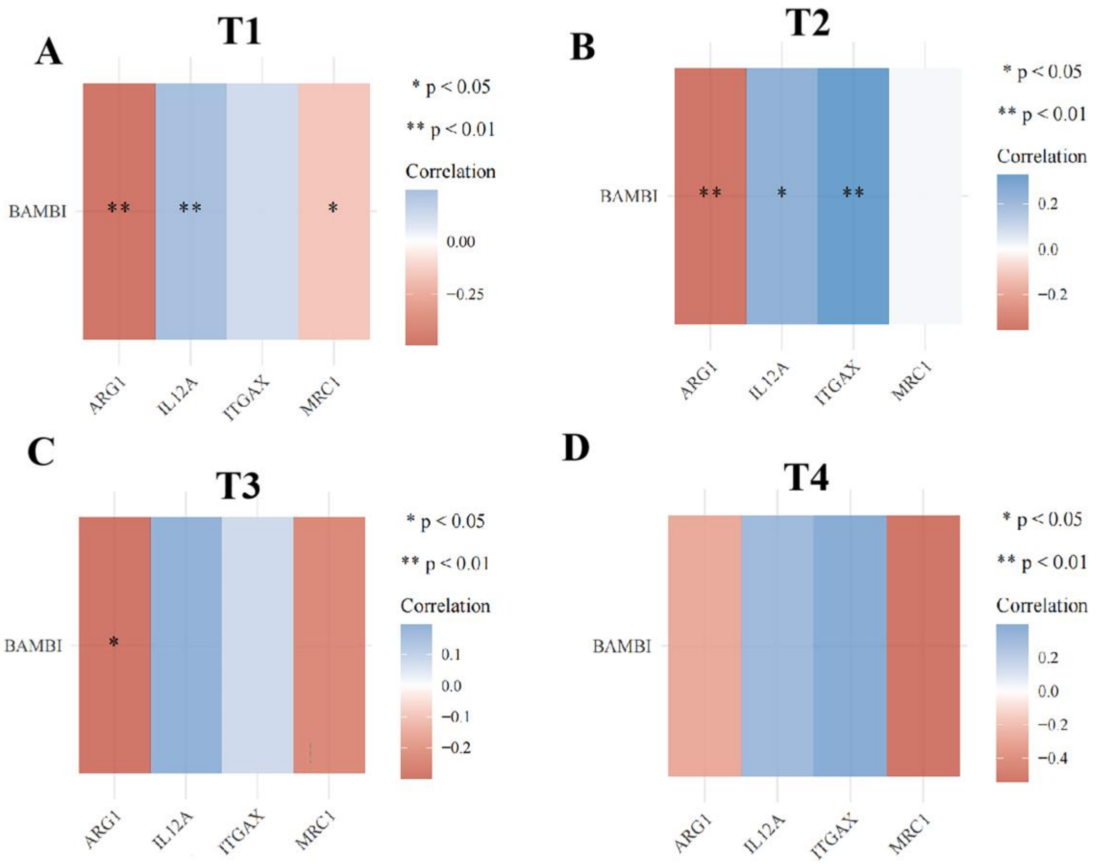

Figure S4. Correlation analysis between BAMBI expression and biomarkers for M1 and M2 macrophages in HCC samples with different stages. The correlation of BAMBI expression and biomarkers for M1 and M2 macrophages in HCC samples with T1 (A), T2 (B), T3 (C) and T4 (D) stages. *P<0.05; **P<0.01.

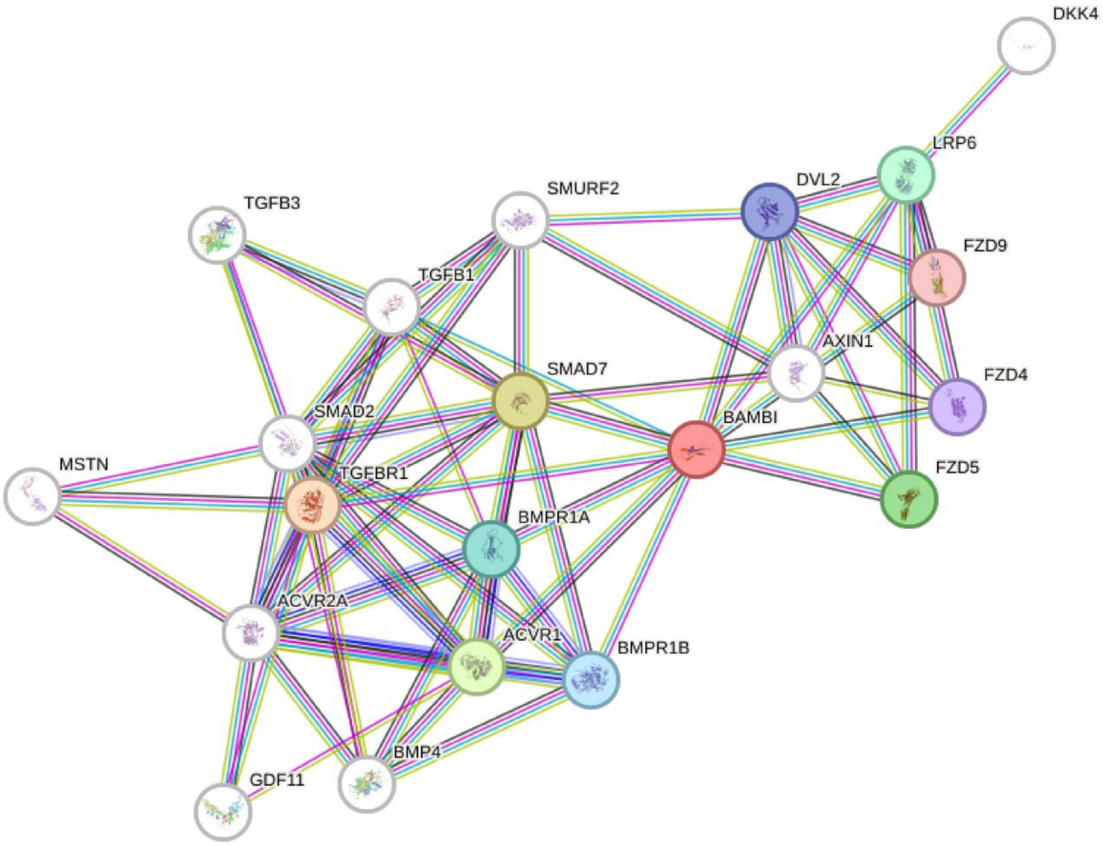

Figure S5. Protein-protein interaction network for BAMBI established using the STRING database. The protein-protein interaction network for BAMBI consists of 21 nodes and 70 edges with high-confidence interactions (interaction score >0.7). Edges in cyan and purple-red represent known interactions; edges in dark green, red, and blue represent predicted interactions; and edges in light green, black, and purple represent text mining, co-expression, and protein homology.

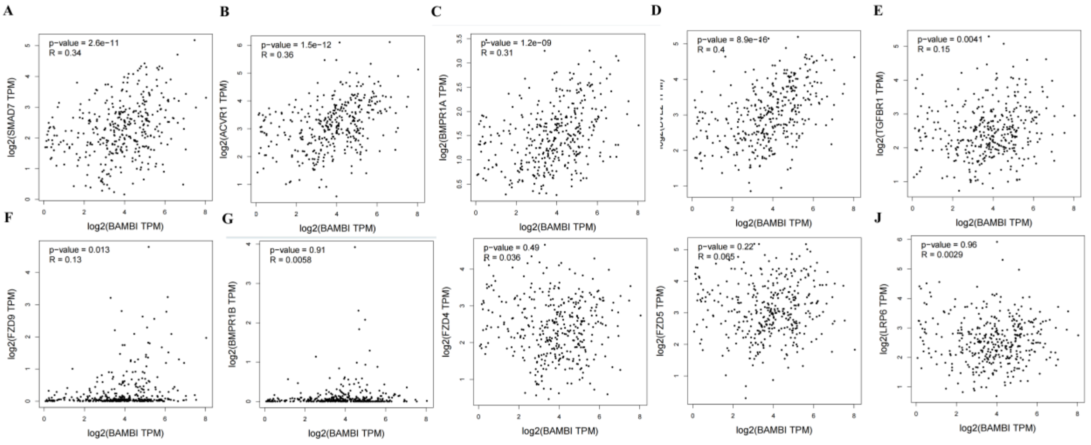

Figure S6. Associations of BAMBI with predicted interacting genes. (A-K) Correlations of BAMBI expression with the expression levels of SMAD7 (A), TGFBR1 (B), BMPR1A (C), BMPR1B (D), ACVR1 (E), FZD4 (F), FZD5 (G), FZD9 (H), DVL2 (I), and LRP6 (J) in HCC. P-values <0.05 were considered statistically significant.
